# Enantioselective Rhodium-Catalyzed Cycloisomerization of 1,6-Allenynes to access 5/6-Fused Bicycle[4.3.0]nonadienes

**DOI:** 10.1038/s41467-019-08900-z

**Published:** 2019-02-27

**Authors:** Xu Deng, Li-Yang Shi, Jialing Lan, Yu-Qing Guan, Xiaoyong Zhang, Hui Lv, Lung Wa Chung, Xumu Zhang

**Affiliations:** 1 Shenzhen Grubbs Institute and Department of Chemistry, Southern University of Science and Technology, Shenzhen, 518055 China; 2 College of Xiangya Pharmaceutical Sciences, Central South University, Changsha, 410013 China; 3 Key Laboratory of Biomedical Polymers of Ministry of Education & College of Chemistry and Molecular Sciences, Engineering Research Center of Organosilicon Compounds & Materials, Ministry of Education, Wuhan University, Wuhan, Hubei 430072 China; 4 School of Chemistry and Chemical Engineering, Harbin Institute of Technology, Harbin, 150001 China

## Abstract

Transition-metal-catalyzed cycloisomerization of 1,n-allenynes represents a powerful synthetic tool to rapidly assemble complex polycyclic skeletons from simple linear substrates. Nevertheless, there are no reports of the asymmetric version of these reactions. Moreover, most of these reactions proceed through a *6-endo-dig* cyclization pathway, which preferentially delivers the distal product (via 5/5 rhodacyclic intermediate) rather than the proximal one (via 6/5 rhodacyclic intermediate). Herein, we report an enantioselective rhodium(I)-catalyzed cycloisomerization of 1,6-allenynes to provide the proximal product 5/6-fused bicycle[4.3.0]nonadienes in good yields and with excellent enantioselectivities. Remarkably, this chemistry works perfectly for 1,6-allenynes having a cyclic substituent within the allene component, thereby affording synthetically formidable tricyclic products with excellent enantioselectivities. Moreover, extensive DFT calculations suggest an uncommon pathway involving 5-*exo*-*dig* cycloisomerization, ring-expansion, rate-determining alkene isomerization involving C_sp3_-H activation, C-C activation of the cyclobutene moiety and finally reductive elimination. Deuterium labeling experiments support the rate-determining step involving the C–H bond activation in this transformation.

## Abstract (web summary)

Cycloisomerization of 1,n-allenynes allows to efficiently assembly polycyclic skeletons, however its asymmetric variant remains elusive. Here, the authors report the enantioselective Rh(I)-catalyzed cycloisomerization of 1,6-allenynes and disclose an uncommon 5-exo-dig pathway via DFT calculations.

## Introduction

**Fig. 1:**
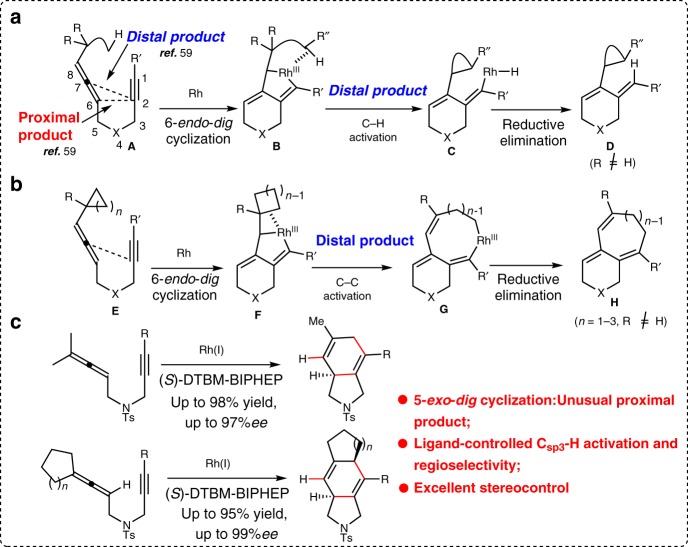
Transition metal-catalyzed cycloisomerization of 1,6-allenynes. **a** Transition metal-catalyzed cycloisomerization of 1,6-allenynes involving C–H activation. **b** Transition metal-catalyzed cycloisomerization of 1,6-allenynes involving C-C activation. **c** This work: enantioselective rhodium(I)-catalyzed cycloisomerization of 1,6-allenynes to form proximal product 5/6-fused bicycle[4.3.0]nonadienes

A pre-eminent goal of synthetic chemistry is the highly-selective construction of targeted structural complexity in an atom- and step-economy fashion^1^. Transition metal-catalyzed cycloisomerization reactions of 1,n-allenynes provide a powerful synthetic approach in this regard, as they facilitate the rapid assembly of complex polycyclic skeletons from simple linear substrates^2^. Since Malacria’s seminal work on the cobalt-catalyzed cycloisomerization of 1,n-allenynes^3^, a variety of metals, such as Rh, Au, Pt, Ru, Pd, Co, Ti, Ag, Ga and Mo, have been reported to catalyze the reaction, which proceeds through the common metallacyclic or carbocation intermediate. Among these contributions, rhodium is of particular interest with respect to the metallacycle pathway. To this end, Brummond reported the first rhodium(I)-catalyzed cycloisomerization of 1,6-allenynes during the screening of the effect of transition metals on the allenic Pauson-Khand reaction^4^, which afforded the commonly observed Alder-ene type product, namely a cross-conjugated triene through the formation of a rhodabicyclic intermediate followed by β-hydride elimination^5–10^. Recently, a diverse set of rhodium(I)-catalyzed cyclizations of 1,6-allenynes involving either site-selective C–H or C-C activation processes have been reported. For example, Sato described a rhodium(I)-catalyzed cyclopropanation/cyclization sequence of *tert*-butylallene-alkynes, which involves C_sp3_–H activation directed by the formation of metallacycles, thus giving cyclopropanes in moderate to good yields^11^. Mukai reported a similar rhodium(I)-catalyzed cycloisomerization of 1,6-allenynes **A** through C_sp3_–H^12^ and C_sp2_–H^13–15^ activation to furnish polycyclic products **D** efficiently (Fig. 1a). In addition, Mukai also reported a series of rhodium(I)-catalyzed cycloisomerization reactions of allenylcycloalkane-alkynes **E** (*n* = 1–3) through a metallacycle directed β-carbon elimination to access a variety of bicyclic products **H** (Fig. 1b)^12,16–20^. These reactions can be tentatively rationalized by the initial formation of the rhodabicyclo[4,3,0] intermediate **B/F**, followed by the activation of the C–H and/or C-C bond proximal to the rhodium(I) center, which presumably collapses to the final products through reductive elimination^12,20–23^. Interestingly, C–H activation and C-C activation compete in some cases, which can be finely tuned by the careful selection of the rhodium(I) catalyst^12,14^. Although a diverse set of cycloisomerization reactions of 1,n-allenynes have been disclosed, to the best of our knowledge, there are no reports on the related asymmetric reactions. Additionally, all the examples involving rhodium(I)-catalyzed C–H and/or C-C activation of allenynes require no α-H at the allenic terminal of the substrate to prevent β-hydride elimination of the possible rhodacyclic intermediate **B**/ **F**^13,15–20,24,25^.

As part of our continuing interests in asymmetric transition-metal-catalyzed cyclizations^26–31^, we now disclose an enantioselective rhodium(I)-catalyzed cycloisomerization of 1,6-allenynes through an unusual 5-*exo*-*dig* cyclization followed by an alkene isomerization via a C_sp3_–H activation process, which enables the synthesis of 5/6-fused bicycle[4.3.0]nonadienes in good yields and excellent enantioselectivities by careful selection of the chiral bisphosphine ligand (Fig. 1c). This work also represents a rare example of a reaction with substrates with α-H at the allenic terminal that favor C_sp3_–H activation rather than β-hydride elimination.

## Results

### Reaction optimization

Previous reports revealed that the intramolecular cycloisomerization of *N*-tethered 1,6-allenynes catalyzed by a rhodium(I) complex modified with a bidentate ligand provided the exclusive formation of the commonly observed Alder-ene product^4–7,9–11,32–34^. Since multiple factors, including the ancillary ligands, the counter-ions and the solvent can dramatically impact the course of rhodium(I)-catalyzed reactions^35^, there are significant opportunities to expand the synthetic possibilities for these highly reactive π components, namely, 1,6-allenynes, towards the rhodium(I) catalyst by careful selection of reaction conditions^2^. We envisaged that rhodium(I) species with a single vacant coordination site for the substrate would exhibit significantly different reactivity and selectivity^36–38^. Therefore, we further investigated the influence of the rhodium(I) catalyst and solvent effects on the reaction. Treatment of **1a** with the chiral rhodium(I) complex generated in situ from [Rh(CH_2_CH_2_)_2_Cl]_2_ (5 mol%) and various chiral diphosphines (10 mol%) and PPh_3_ (10 mol%) in the presence of silver salt (20 mol%) in degassed 1,2-dicholoroethane at room temperature indicates that the product distribution is highly ligand-dependent (Table 1 entries 1–4). For instance, **L1**-**L3** furnished the commonly observed Alder-ene type product **3a** exclusively (entries 1, 2, 3), whereas the highly strained and electron-rich *C2*-symmetric biarylphosphane ligand **L4** furnished the unexpected 5/6-fused cycloadduct **2a**, along with **3a** in moderate yield, albeit with poor enantio-induction (entry 4). This result motivated further investigations to improve regio- and enantioselectivity of the reaction. Interestingly, replacing the pre-catalyst [Rh(CH_2_CH_2_)_2_Cl]_2_ to a cationic (entry 5) and neutral rhodium(I) precursor (entry 6) were inefficient, either leading to no conversion or the exclusive formation of **3a**. Gratifyingly, the examination of the solvent on product formation and distribution led to significant improvements. For instance, 1,4-dioxane gave encouraging regio- and enantio-induction (entries 10 and 11), whereas other solvents, such as CHCl_3_, toluene, THF, were inferior (entries 7–9). Similar solvent effects were also observed by Sato and coworkers in their investigation of the ruthenium(I)-catalyzed cyclization of 1,6-allenynes^39^. Moreover, closer examination of the influence of silver salts revealed that counter-ions exerted negligible effects on the reaction outcomes. Subsequent screening of monodentate ligands demonstrated that PCy_3_ delivered the desired** 2a** in good yield, along with satisfactory regioselectivity and excellent enantioselectivity (entry 12). Additionally, the reaction proceeds smoothly in the absence of an additional monodentate ligand, albeit with modest regio-selection. This suggests that additional ligand is not necessary for the reaction, whereas it improves the regio- and enantiocontrol. Finally, control experiments demonstrated that AgBF_4_ did not catalyze the cyclization in the absence of rhodium, thereby indicating a rhodium-specific reaction.

**Table 1:** Reaction development and optimizations

NBD, norbornadiene; DCE, 1,2-dicholoroethane; THF, tetrahydrofuran

^a^All the reactions were conducted with **1a** (0.1 mmol) with an in situ generated ligand (10 mol%)-PPh_3_ (10 mol%)-Rh(I) (5 mol%) complex in the presence of Ag(I) salt (20 mol%) in degassed solvent at 40 °C unless otherwise noted

^b^The combined yield of **2a** and **3a**, isolated yields were reported

^c^Determined by ^1^H-NMR of the mixture

^d^Determined by HPLC using a chiral stationary phase, See Supplementary Figure 41

^e^1,4-Dioxane

^f^No reaction

^g^PCy_3_ was used as the additional ligand

^h^Without use of the additional ligand

The bold values means the results under the optimal conditions

### Substrate scope

In light of the promising preliminary results with the optimal catalyst system (Table 1, entry 12), we selected to examine the scope and limitations of this rhodium(I)-catalyzed cycloisomerization of 1,6-allenynes (Table 2). For *N*-tethered substrates, the reactions are sensitive to the substituent on the alkyne terminus. All *ortho*-, *meta*- and *para*-substituted aryl groups are generally well tolerated (entries 1–8), affording the desired 5/6-fused cycloadducts in moderate to good yields with good to excellent enantioselectivities. Additionally, the aryl substitution pattern has an interesting impact on the reaction outcome. For example, electron-withdrawing groups proved to be beneficial to the yields, regio- and enantioselectivities (entries 1, 3, 4, 7 and 8), whereas electron-donating groups are detrimental (entries 2, 5 and 6). In addition, the 3-cyano aryl substituent led to low level of enantioinduction (entry 4), which may be attributed to the chelation to the rhodium center. Hetero-aryl groups on the alkyne terminus are also well tolerated (entry 10); however, *N*-tethered 1,6-allenynes with terminal alkynes (R = H, Supplementary Table 1, entry 3), or containing alkyl groups or other functional groups on the alkyne terminus (Supplementary Table 1, entries 1, 2 and 4), including *O*-tethered congeners (Supplementary Table 1, entries 5 and 6) afforded the exclusive formation of the Alder-ene type product, without any detectable amount of the corresponding 5/6-fused bicyclic product. These results indicate that the reaction is sensitive to the subtle changes in the nature of the tether and substituent. X-ray crystallographic analysis of** 2g** unambiguously established its structure possessing a bicycle[4.3.0]nonadiene framework with the absolute configuration of C8 having an *S* configuration.

**Table 2:** Cycloisomerization of 1,6-allenynes

^a^ The reactions were conducted with **1** (0.1 mmol) with in situ generated complex with (*S*)-DTBM-BIPHEP (10 mol%), PCy_3_ (10 mol%), [{Rh(CH_2_CH_2_)_2_Cl}_2_] (5 mol%) in the presence of AgBF_4_ (20 mol%) in 1,4-dioxane at 40 °C unless otherwise noted; ^b^ The combined yield of **2** and **3**, isolated yields were reported; ^c^ Determined by ^1^H-NMR of the mixture; ^d^ Determined by HPLC using a chiral stationary phase, see Supplementary Figures 41–51.

Encouraged by aforementioned results, we wondered if the current catalytic system was also applicable to substrates with cyclic subunits within the allene component. Thus, several cyclic 1,6-allenynes were examined (Table 3). Gratifyingly, 1,6-allenynes both having a five-membered or a six-membered cyclic substituent within the allene proceed smoothly and provide improved results, thereby delivering the synthetically challenging tricyclic adducts **5** in high yields with excellent regio- and stereoselectivities (entries 1–4). In particular, the reactions displayed excellent diastereoselectivities (entries 1–4). For each example examined, only one diastereoisomer was detected by HPLC in addition to ^1^H-NMR (400 MHz). The proposed relative stereochemical assignment of **5c** was assigned based on the NOESY analysis (See Supplementary Figure 16).

**Table 3:** Cycloisomerization of cyclic 1,6-allenynes

^a^ The reactions were conducted with **4** (0.1 mmol) with in situ generated complex with (*S*)-DTBM-BIPHEP (10 mol%), PCy_3_ (10 mol%), [{Rh(CH_2_CH_2_)_2_Cl}_2_] (5 mol%) in the presence of AgBF_4_ (20 mol%) in 1,4-dioxane at 40 °C unless otherwise noted

^b^The combined yield of **5** and **6**, isolated yields were reported

^c,d^Determined by ^1^H-NMR of the mixture

^e^Determined by HPLC using a chiral stationary phase, see Supplementary Figures 52–55

### Mechanistic studies

**Fig. 2:**
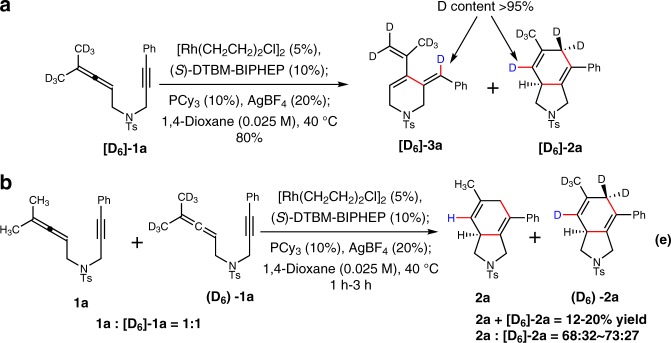
Mechanistic investigations. **a** Cycloisomerization of [D_6_]-**1a** under the optimized reaction conditions; **b** The kinetic isotope competition experiment by using the mixture of **1a** and [D_6_]-**1a**

To gain some insight into the reaction mechanism, we investigated the cyclization of [D_6_]-**1a** under the optimal reaction conditions (Table 1, entry 12). As expected, the reaction gave a mixture of the desired 5/6-fused bicyclic product [D_6_]-**2a** and Alder-ene type product [D_6_]-**3a** with the ratio of 1:1, wherein deuterium atom was transferred to the expected position in high content (Fig. 2a). In addition, a kinetic isotope competition experiment using an equimolar mixture of **1a** and [D_6_]-**1a** by quenching at early stages (1–3 h) revealed that the reaction gave a mixture of **2a** and [D_6_]-**2a** in 12~20% yield with the ratio of 68:32~73:27. The corresponding KIE was estimated to be ~2.5 (Fig. 2b). These results suggested that the rate-determining step probably involves C–H bond activation or formation.

To further understand the reaction mechanism of this unusual rhodium(I)-catalyzed cycloisomerization of 1,6-allenynes, an extensive DFT (M06-L/6–31 G* was used for the geometry optimization) study was conducted on seven possible pathways (**i**–**vii**) using the ligand **L4**, with PMe_3_ and substrate **1a** (with 249 atoms in total). In addition, for the most possible pathway (**i-Ph**), PPh_3_ ligand was employed as opposed to PMe_3_ in order to draw a better comparison with the actual experimental results.

**Fig. 3:**
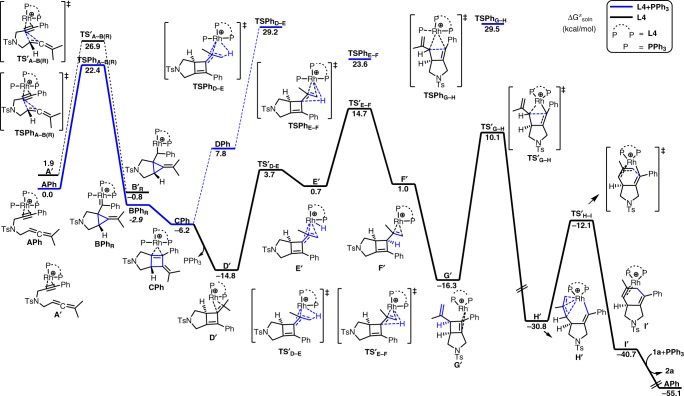
DFT Results. The computed free energy profiles for the most possible pathway (**i-Ph**) of the Rh(I)-catalyzed cycloisomerization of 1,6-allenyne (**1a**) in solution by SMD M06-L//M06-L method

**Fig. 4:**
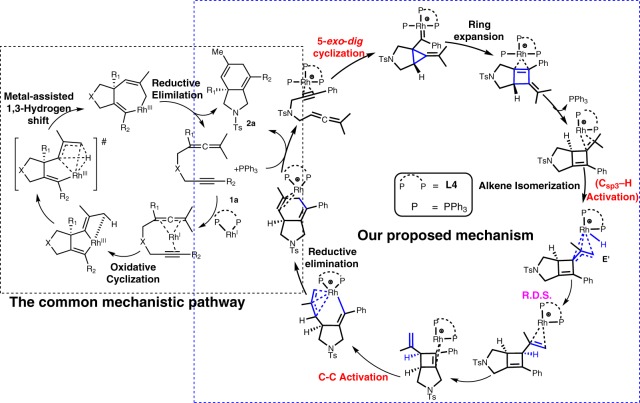
The mechanistic pathways. The common pathway and our proposed pathway for the uncommon formation of 5/6-Fused Bicycle[4.3.0]nonadienes

**Fig. 5:**
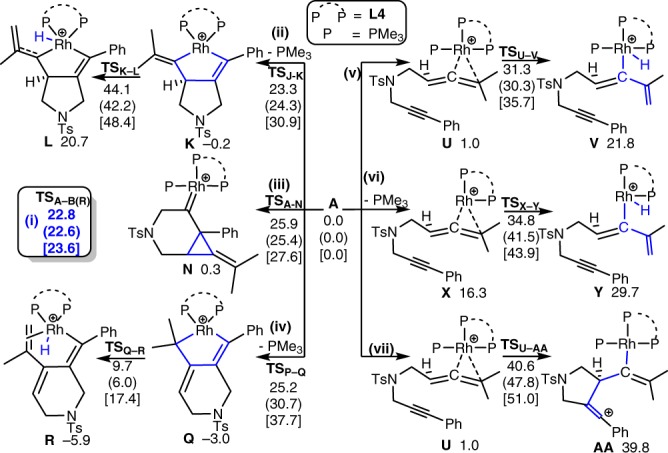
Computational studies on several pathways. The free energy profiles for different reaction pathways of the cationic Rh(I)-catalyzed cycloisomerization of 1,6-allenyne (**1a**) using a PMe_3_ ligand in solution by the SMD M06-L//M06-L method. The relative free energy for the key transition states by SMD PBE0-D3//M06-L and SMD B3LYP-D3//M06-L methods are also given in parenthesis and bracket, respectively

Although the formation of the 5/6-fused bicycle[4.3.0]nonadienes and Alder-ene type products could be rationalized by the common initial oxidative cyclization pathways to form the rhoda(III)bicyclic intermediates (Fig. 4), these common pathways were not supported by different DFT methods (M06-L, M06, PW6B95-D3, B3LYP-D3, B3PW91-D3, BP86-D3, PBE0-D3 and ωB97XD methods for the energy calculations) due to their higher barriers (Figs. 3–5). As shown in Figs. 3–5, Supplementary Figures 56–62 and Table 4, our computational results emerged a uncommon pathway that the most favorable pathway starts from a stable rhodium(I) complex **APh** (Fig. 3 and Fig. 4)^40^, in which the two phosphine atoms of **L4** and a PPh_3_ ligand as well as the alkyne moiety of **1a** coordinate to the acidic rhodium(I) metal. Then, the cationic metal complex **APh** preferentially undergoes 5-*exo*-*dig* cyclization with two new C-C bond formations to give a stable rhodium(I)-carbenoid complex **BPh**_**R**_ (Δ*G* = –2.9 kcal/mol) with a barrier of about 22.4 kcal/mol in solution by the M06-L method. The similar computed barrier was also obtained from different DFT methods (~22.4–25.8 kcal/mol, Table 4). The 5-*exo*-*dig* cyclization is subsequently followed by a ring expansion to form a more stable bicyclic intermediate **CPh** (formal [2 + 2] cycloaddition product)^11,12,36,41–44^. Such transformation is (partly) analogous to Pt/Au-catalyzed cycloisomerization of allenynes^23,35,36,41–44^. After dissociation of the bulky PPh_3_ ligand from **CPh** and shift of another rhodium(I)-alkene coordination mode to form **D′** (Δ*G* = −14.8 kcal/mol), isomerization of this alkene part from the methylethylidene moiety proceed. This Rh(I)-catalyzed isomerization involves oxidative addition through **TS′**_**D-E**_ to give an unstable η^3^-allyl rhodium(III)-hydride intermediate **E′** (Δ*G* = 0.7 kcal/mol). Such Rh(I)-mediated allylic C_sp3_–H activation was also proposed to be involved in another Rh(I)-catalyzed cycloisomerization reactions^40,45,46^. Notably, this Rh(I)-catalyzed C_sp3_–H activation (oxidative addition) process is different from the common pathway involving β-hydride elimination from rhoda(III)bicyclic intermediates (see Fig. 1 and 4). Then, the rate-determining rhodium(III)-mediated allylic C_sp3_–H bond-forming reductive elimination via **TS′**_**E-F**_ takes place to give rhodium(I) intermediates **F′** and **G′** with an overall barrier of ~29.5 kcal/mol above **D′**. Such isomerization transforms intermediate **D′** with the tetrasubstituted alkene moieties into intermediate **F′** and **G′** with the disubstituted alkene moieties. The alkene isomerization step is followed by the rhodium(I)-catalyzed C-C bond activation^21,47–49^ of the cyclobutene moiety of **G′** via **TS′**_**G-H**_ to give a stable rhodacycle intermediate **H′** by overcoming the barrier of roughly 26.4 kcal/mol above **G′**. Again, such C-C bond activation process involving rhodium(I)-catalyzed oxidative addition is also different from that in the common pathways involving the rhoda(III)bicyclic intermediates (Fig. 1 and 4). Finally, C-C forming reductive elimination completes the reaction to afford the desired 5/6-fused bicyclic product **I′** with regeneration of a cationic Rh(I) complex. The overall process was computed to be exergonic by about 55.1 kcal/mol in solution. In general, different DFT methods give a qualitatively similar mechanistic picture and suggest the highest overall barrier for the C_sp3_–H bond-forming reductive elimination, which is in qualitatively agreement with our KIE experiments (Fig. 2)^50^. This mechanism involving the uncommon 5-*exo*-*dig* cyclization, rhodium(I)-catalyzed C–H bond activation and C-C bond activation steps was proposed to be the key factor to form the unusual 5/6-fused bicycle[4.3.0]nonadienes.

**Table 4:** The relative free energies (in kcal/mol) of the most possible pathway (i-Ph) and the barrier for the lowest-energy side-reaction oxidative coupling pathway (via TS_P-Q_) for the Rh(I)-catalyzed cycloisomerization of a 1,6-allenyne (1a) in solution phase by different DFT methods (except PW6B95-D3 in gas phase). PPh_3_ was used as the additional ligand

	ΔG_B3LYP-D3_^a^	ΔG_B3PW91-D3_^a^	ΔG_PBE0-D3_^a^	ΔG_ωB97XD_^a^	ΔG_PW6B95-D3_^a^
**APh**	0.0	0.0	0.0	0.0	0.0
**A′**	15.6	21.2	11.7	14.7	20.6
**TSPh**_**A-B(R)**_	25.6	22.4	24.0	25.8	24.5
**TS′**_**A-B(R)**_	39.4	43.4	34.4	41.4	45.1
**BPh**_**(R)**_	6.3	−1.9	−3.1	−2.7	0.8
**B′**_**R**_	16.2	16.2	4.1	10.3	19.3
**CPh**	5.1	−6.2	−7.3	−7.6	−4.5
**DPh**	23.1	10.5	7.9	9.5	10.9
**D′**	5.7	1.1	−11.9	−7.3	3.1
**TSPh**_**D-E**_	41.1	30.3	26.0	32.0	33.6
**TS′**_**D-E**_	21.8	17.3	3.7	9.4	17.9
**E′**	16.7	14.8	1.4	7.6	17.2
**TS′**_**E-F**_	32.7	28.0	14.8	21.7	28.4
**TSPh**_**E-F**_	35.2	22.7	18.8	22.7	24.4
**F′**	19.8	18.1	3.0	8.6	17.8
**G′**	1.4	−2.7	−15.1	−11.3	−2.0
**TS′**_**G-H**_	24.6	23.1	13.3	20.2	24.7
**TSPh**_**G-H**_	40.5	31.9	31.2	30.9	31.7
**H′**	−15.3	−21.5	−30.5	−25.8	−19.9
**TS′**_**H-I**_	2.0	−3.8	−14.4	−9.7	−2.7
**I′**	−24.6	−29.3	−42.2	−38.5	−30.9
**TS**_**P-Q**_	36.1	33.1	24.6	31.4	33.2

In comparison, different DFT methods also show that the lowest-energy side-reaction oxidative coupling pathway (via **TS**_**P-Q**_) generally has a larger barrier than that for the above-mentioned 5-*exo*-*dig* cyclization via **TSPh**_**A-B(R)**_. These computational results demonstrated that the second PPh_3_ ligand play a critical role in directing the reaction towards the initial 5-*exo*-*dig* cyclization step and suppressing the common initial oxidative coupling pathway. In the absence of the PPh_3_ ligand, 1,4-dioxane molecule might coordinate to the rhodium(I) metal center and thus disfavor the oxidative coupling pathway to form the Alder-ene type product.

The overall reaction mechanism for the most favorable pathway is slightly different, when a smaller PMe_3_ ligand was used to replace PPh_3_ as the monodentate ligand. Notably, the computed barrier for the 5-*exo*-*dig* cyclization with the PMe_3_ ligand is quite similar (22.8 kcal/mol); however, the bulky PPh_3_ ligand is only involved in the first stage of the cycloisomerization and then dissociates from the metal, presumably due to steric repulsion with the bulky **L4** ligand and substrate. In contrast, PMe_3_ can re-coordinate to the metal center in the key C_sp3_–H bond-forming reductive elimination step. In comparison, our DFT results show that the common pathways (such as initial 6-*endo*-*dig* cyclization (pathway **iii**), initial oxidative coupling between the alkyne and allene parts (pathways **ii** and **iv**), initial C_sp3_–H activation (pathways **v** and **vi**) and initial single C-C bond forming (pathway **vii**), see Fig. 5) were computed to have higher barriers than the 5-*exo*-*dig* cyclization via **TS**_**A–B(R)**_ in the most favorable pathway (**i**), presumably due to a stronger binding affinity of PMe_3_ than the alkene and a more bulky ligand **L4** disfavoring the above-mentioned common pathways. Our proposed most favorable pathway was also supported by different DFT methods (Supplementary Tables 2–27). Moreover, our DFT calculations also suggest that the alkene isomerization step with the new C–H bond formation is the possible rate-determining step.

## Discussion

In summary, this study developed an enantioselective ligand-controlled rhodium(I)-catalyzed cycloisomerization of 1,6-allenynes for the synthesis of 5/6-fused bicycle[4.3.0]nonadiene skeletons through a 5-*exo*-*dig* cyclization pathway. Notably, this synthetic method also works for cyclic 1,6-allenynes to afford the diastereoselective construction of tricyclic products, which are challenging for classical synthetic methods and will be important for applications in drug and natural products synthesis. Although both 5-*exo-dig* and 6-*endo-dig* pathways are not surprising in metal-catalyzed alkyne closure^51–53^ and it is quite common for those with gold catalysts to provide 5-*exo-dig* products^51,54–58^, it is very unusual for rhodium catalysts to favor a 5-*exo-dig* pathway^58^, thereby preferentially forming the proximal product (via 5/5 rhodacyclic intermediate) rather than the distal product (via 6/5 rhodacyclic intermediate)^59^. Our systematic DFT study provides an insight over the classical mechanistic pathways (such as initial 6-*endo*-*dig* cyclization, initial oxidative coupling between the alkyne and allene parts and C_sp3_–H activation). Hence, the calculations suggest a uncommon pathway initiated with a rare 5-*exo*-*dig* cyclization followed by ring expansion, alkene isomerization via rate-determining rhodium(I)-catalyzed C–H bond activation, rhodium(I)-catalyzed C-C bond activation of the cyclobutene moiety and final C-C bond-forming reductive elimination was the most favorable pathway. Deuterium labeling experiments further support the rate-determining step involving the C–H bond activation in this transformation. The present chemical transformation provides a highly regio- and stereoselective transformation, which was attained by the screening of ligands, solvents and additives. Further studies on the application of this method to the synthesis of natural products are in progress.

## Methods

### Procedures for synthesis of 2 and 5

In an oven-dried Shlenk tube, [Rh(CH_2_CH_2_)_2_Cl]_2_ (0.005 mmol) and the chiral diphosphane ligand (0.01 mmol) were dissolved in freshly distilled 1,4-dioxane (1.0 mL). The mixture was stirred at 25 °C under argon for 0.5 h. Then PCy_3_ (0.01 mmol) was added to the solution and was stirred at 25 °C for additional 6 h. Then Ag(I) salt (0.02 mmol) was added to the mixture and was stirred for further 15 min, followed by adding 1,4-dioxane (3 mL) and the 1,6-allenyne **1** or **4**(0.1 mmol) successively. The resulting mixture was stirred at 40 °C under argon until no starting material was detected by TLC. Upon the completion of the reaction, the solvent was removed. The crude mixture was directly subjected to column Chromatography on silica gel using petrol ether/EtOAc (30:1–10:1) as eluent to give the desired product.

### Procedures for synthesis of 3 and 6

In a oven-dried Shlenk tube, [Rh(CH_2_CH_2_)_2_Cl]_2_ (0.0025 mmol) and ( ± )-BINAP (0.005 mmol) were dissolved in freshly distilled 1,4-dioxane (1.0 mL). The mixture was stirred at 25 °C under argon for 0.5 h. Then Ag^I^ salt (0.01 mmol) was added to the mixture and was stirred for further 15 min, followed by adding 1,4-dioxane (1 mL) and the 1,6-allenyne **1 **or **4** (0.1 mmol) successively. The resulting mixture was stirred at 25 °C under argon until no starting material was detected by TLC. Upon the completion of the reaction, the solvent was removed. The crude mixture was directly subjected to column Chromatography on silica gel using petrol ether/EtOAc (30:1–10:1) as eluent to give the desired product.

### Measurement of enantiomeric excess (*ee*)

Racemic 5/6 bicylic products were prepared with (±)-DTBM-BIPHEP as the chiral ligand according to the general procedure described above. Similarly, optically active 5/6 bicyclic products were prepared with (*S*)-DTBM-BIPHEP as the chiral ligand according to the procedure described above. The *ee* value was determined by chiral HPLC (CHIRALPAK AD-H, OD-H, AS-H and IB-H column).

### Computational Details

All DFT calculations were performed using the Gaussian 09 program^60,61^, except for the single-point gas-phase calculations by PW6B95D3 method, which were performed by the Gaussian 16 program. M06-L method was used to fully optimize the reactants, intermediates, products and transition states (TS) in gas phase. The 6–31 G* basis set was used for H, C, N, O, P, S and Cl atoms, while the SDD basis set and its effective-core potential were used for the Rh and Ag atom. Frequency calculations were then carried out to confirm transition states with one imaginary frequency and minima without imaginary frequency. Solvent effects (1,4-dioxane) were also included through implicit SMD solvent model^60^ by carrying single-point solvent calculations on the optimized geometries in gas phase. Single-point calculations in solution by several functionals (B3LYP-D3, B3PW91-D3, BP86-D3, M06, PBE0-D3 and ɷB97XD)^62–64^ were also performed to examine effect of the functionals on the energetic profiles. Moreover, single-point calculations in gas phase PW6B95D3 were also carried out.

## Supplementary information

Supplementary Information

Description of Additional Supplementary Files

Supplementary Data 1

## Data Availability

Crystallographic data for the structure **2g** reported in this paper have been deposited at the Cambridge Crystallographic Data Centre under deposition number CCDC 1586680. Copies of the data can be obtained free of charge via www.ccdc.cam.ac.uk. All other data supporting the findings of this study, including experimental procedures and compound characterization, are available within the paper and its Supplementary Information, or from the corresponding author upon reasonable request.
