# Supplementary material for: Enantioselective Rhodium-Catalyzed Cycloisomerization of 1,6-Allenynes to access 5/6-Fused Bicycle[4.3.0]nonadienes
Source: Nat Commun. 2019 Feb 27;10:949. doi: 10.1038/s41467-019-08900-z (PMC6393573; doi:10.1038/s41467-019-08900-z)
Supplement: Supplementary file 1 — Supplementary Information [file 41467_2019_8900_MOESM1_ESM.pdf]

Enantioselective Rhodium-Catalyzed Cycloisomerization of 1,6-Allenynes

Deng et al.

Supplementary Methods

General procedures. All reactions were carried out under argon atmosphere using flame-dried glassware unless otherwise noted. Methylene chloride (CH_2Cl_2), 1,2-Dichloride ethane ($ClCH_2CH_2Cl$, DCE) were dried over CaH_2. THF, 1,4-dioxane, toluene were distilled over sodium metal. All reagents were commercially available and used without further purification unless indicated otherwise. Reagents were purchased at the highest commercial quality and used without further purification, unless otherwise stated. Reactions were monitored by thin layer chromatorgraphy (TLC) carried out on GF254 plates (0.25 mm layer thickness) using UV light as visualizing agent and aqueous ammonium cerium nitrate/ammonium molybdate and sulfuric acid/ethanol solution (15%) as developing agents. Flash chromatography was performed with 300–400 mesh silica gels.

^{1}H and ^{13}C–NMR experiments were performed on a Bruker AM-400 spectrometer at ambient temperature. The residual solvent protons (^{1}H) or the solvent carbons (^{13}C) were used as internal standards. ^{1}H-NMR data are presented as follows: chemical shift in ppm downfield from tetramethylsilane (multiplicity, coupling constant, integration). The following abbreviations are used to designate multiplicities in reporting NMR data: s, singlet; br s, broad singlet; d, doublet; t, triplet; q, quartet; qt, quartet of triplets; dd, doublet of doublets; dt, doublet of triplets; AB, AB quartet; m, multiplet. High-resolution mass spectra (HRMS) was taken on Agilent ESITOF (time of flight) mass spectrometer at a 4000 V emitter voltage.

Typical procedure for Rh-catalyzed cycloisomerization of 1,6 allenyne. In a oven-dried Shlenk tube, $[Rh(CH_2CH_2)_2Cl]_2$ (0.005 mmol) and the chiral diphosphane ligand (0.01 mmol) were dissolved in freshly distilled 1,4-dioxane (1.0 mL). The mixture was stirred at 25 °C under argon for 0.5 hours. Then PCy_3 (0.01 mmol) was added to the solution and was stirred at 25 °C for another 6 hours. Then Ag(I) salt (0.02 mmol) was added to the mixture and was stirred for further 15 min, followed by adding 1,4-dioxane (3 mL) and the 1,6-allenyne (0.1 mmol) successively. The resulting mixture was stirred at 40 °C under argon until no starting material was

detected by TLC. Upon the completion of the reaction, the solvent was removed. The crude mixture was directly subjected to column Chromatography on silica gel using petrol ether/EtOAc (30:1-10:1) as eluent to give the desired product.

And the alder-ene type products were prepared with (±)-BINAP as the chiral ligand according to the general procedure described below: In a oven-dried shlenk tube, $[Rh(CH_2CH_2)_2Cl]_2$ (0.0025 mmol) and (±)-BINAP (0.005 mmol) were dissolved in freshly distilled 1,4-dioxane (1.0 mL). The mixture was stirred at 25 ℃ under argon for 0.5 hours. Then Ag^I salt (0.01 mmol) was added to the mixture and was stirred for further 15 min, followed by adding 1,4-dioxane (1 mL) and the 1,6-allenyne (0.1 mmol) successively. The resulting mixture was stirred at 25 °C under argon until no starting material was detected by TLC. Upon the completion of the reaction, the solvent was removed. The crude mixture was directly subjected to column Chromatography on silica gel using petrol ether/EtOAc (30:1-10:1) as eluent to give the desired product.

Measurement of enantiomeric excess (*ee*). Racemic 5/6 bicylic products were prepared with (±)-DTBM-BIPHEP as the chiral ligand according to the general procedure described above. Similarly, optically active 5/6 bicyclic products were prepared with (*S*)-DTBM-BIPHEP as the chiral ligand according to the procedure described above. The *ee* value was determined by chiral HPLC (CHIRALPAK AD-H, OD-H, AS-H and IB-H column).

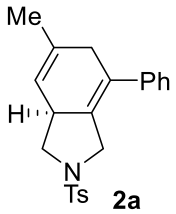

2a

2a, 25 mg, 69% yield, 90% *ee* (OD-H, Hexane:*i*-PrOH = 95:5, 0.5 mL/min, 254 nM, t1 = 24.0 min, t2 = 28.3 min), $[\alpha]_D^{25}$ = -16.0 in $CHCl_3$; ^{1}H-NMR (400 MHz, $CDCl_3$) δ 7.64 (d, *J* = 8.2 Hz, 2H), 7.35 – 7.31 (m, 2H), 7.27 (dd, *J* = 7.1, 3.0 Hz, 3H), 7.16 – 7.09 (m, 2H), 5.42 (s, 1H), 4.22 – 4.13 (m, 1H), 3.84 (t, *J* = 8.3 Hz, 1H), 3.67 (d, *J* = 13.9 Hz, 1H), 3.31-3.16 (m, 1H), 2.88 (dd, *J* = 15.5, 8.6 Hz, 2H), 2.63 (dd, *J* = 11.4, 8.8 Hz, 1H), 2.40 (s, 3H), 1.75 (s, 3H) ppm; ^{13}C-NMR (101 MHz, $CDCl_3$) δ 143.3, 139.9, 134.5, 133.9, 130.5, 129.7, 129.5, 128.4, 127.6, 127.4, 127.3, 117.4, 52.4, 49.9, 40.9, 36.4, 22.7, 21.5 ppm; HR-ESI-MS (*m/z*): calcd. for $C_{22}H_{24}NO_2S$ $[M+ H]^+$, 366.1528, found 366.1521.

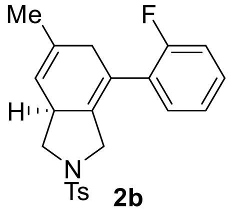

2b

2b, 26 mg, 68% yield, 89% *ee* (AD-H, Hexane:*i*-PrOH = 95:5, 0.5 mL/min, 254 nM, t1 = 46.0 min, t2 = 47.9 min), $[\alpha]_D^{25}$ = -2.9 in $CHCl_3$; ^{1}H-NMR (400 MHz, $CDCl_3$) δ 7.64 (d, *J* = 8.2 Hz, 2H), 7.35 – 7.26 (m, 3H), 7.16 – 7.09 (m, 3H), 5.42 (s, 1H), 4.62 (d, *J* = 2.1 Hz, 1H), 4.22 – 4.13 (m, 1H), 3.84 (t, *J* = 8.3 Hz, 1H), 3.67 (d, *J* = 13.9 Hz, 1H), 3.31-3.16 (m, 1H), 2.88 (dd, *J* = 15.5, 8.6 Hz, 2H), 2.63 (dd, *J* = 11.4, 8.8 Hz, 1H), 2.40 (s, 3H), 1.75 (s, 3H) ppm; ^{13}C-NMR (101 MHz, $CDCl_3$) δ^{13}C NMR (101 MHz, $CDCl_3$) δ = 158. 2, 143.4, 134.5, 133.7, 133.2, 129.8, 129.7, 129.7, 129.3, 129.2, 127.4, 124.2, 124.1, 123.9, 117.3, 116.0, 115.8, 52.8, 49.7, 40.4, 36.4, 22.7, 21.5 ppm; HR-ESI-MS (*m/z*): calcd. for $C_{22}H_{24}NO_2S$ $[M+ H]^+$, 366.1528, found 366.1521.

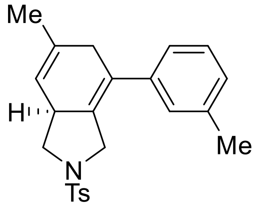

2c

2c, 19 mg, 50% yield, 80% *ee* (OD-H, Hexane:*i*-PrOH = 95:5, 0.5 mL/min, 254 nM, t1 = 24.0 min, t2 = 28.3 min), $[\alpha]_D^{25}$ = -15.9 in $CHCl_3$; ^{1}H-NMR (400 MHz, $CDCl_3$) δ 7.64 (d, *J* = 8.3 Hz, 2H), 7.32 – 7.22 (m, 3H), 7.11 (dd, *J* = 10.1, 6.6 Hz, 1H), 6.93 (dd, *J* = 13.9, 7.3 Hz, 2H), 5.41 (s, 1H), 4.16 (ddd, *J* = 18.6, 4.8, 3.2 Hz, 1H), 3.89 – 3.77 (m, 1H), 3.68 (d, *J* = 13.8 Hz, 1H), 3.28-3.16 (m, 1H), 2.91 – 2.79 (m, 2H), 2.62 (dd, *J* = 11.4, 8.8 Hz, 1H), 2.40 (s, 3H), 2.35 (s, 3H), 1.74 (s, 3H) ppm; ^{13}C-NMR (101 MHz, $CDCl_3$) δ 143.3, 139.9, 134.5, 133.9, 130.2, 129.8, 129.6, 128.3, 128.2, 127.9, 127.6, 127.5, 124.5, 117.4, 52.5, 49.9, 40.8, 36.5, 22.7, 21.5, 21.5 ppm; HR-ESI-MS (*m/z*): calcd. for $C_{23}H_{26}NO_2S$ $[M+H]^+$, 380.1684, found 380.1677.

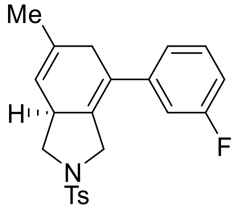

2d

2d, 29 mg, 75 % yield, 97% *ee* (OD-H, Hexane:*i*-PrOH = 95:5, 0.4 mL/min, 254 nM, t1 = 24.0 min, t2 = 28.3 min), $[\alpha]_D^{25}$ = -14.9 in $CHCl_3$; ^{1}H-NMR (400 MHz, $CDCl_3$) δ 7.64 (d, *J* = 8.3 Hz, 2H), 7.30 – 7.25 (m, 3H), 7.01 – 6.95 (m, 1H), 6.90 (d, *J* = 7.7 Hz, 1H), 6.86 – 6.76 (m, 1H), 5.41 (s, 1H), 4.25 – 4.09 (m, 1H), 3.84 (t, *J* = 7.8 Hz, 1H), 3.66 (dd, *J* = 14.0, 1.2 Hz, 1H), 3.21 (ddd, *J* = 11.4, 8.7, 4.8 Hz, 1H), 2.84 (d, *J* = 8.1 Hz, 2H), 2.63 (dd, *J* = 11.4, 8.8 Hz, 1H), 2.41 (s, 3H), 1.75 (s, 3H) ppm; ^{13}C-NMR (101 MHz, $CDCl_3$) δ 163.9, 161.5, 143.4, 134.3, 133.7, 131.6, 130.0, 129.9, 129.7, 128.5, 128.5, 127.4, 123.2, 123.2, 117.3, 114.5, 114.3, 114.1, 52.4, 49.8, 40.9, 36.3, 22.7, 21.5 ppm; HR-ESI-MS (*m/z*): calcd. for $C_{22}H_{23}NO_2SF$ $[M+H]^+$, 384.1434, found 384.1424.

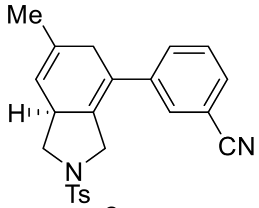

2e

2e, 25 mg, 64 % yield, 45% *ee* (OD-H, Hexane : *i*-PrOH = 95:5, 0.4 mL/min, 254 nM, t1 = 41.5 min, t2 = 41.7 min), ^{1}H NMR (400 MHz, $CDCl_3$) δ = 7.65 (d, *J*=8.2 Hz, 2H), 7.61 – 7.57 (m, 1H), 7.47 (t, *J*=7.7 Hz, 1H), 7.42 – 7.35 (m, 2H), 7.30 (d, *J*=8.0 Hz, 2H), 5.43 (s, 1H), 4.12 (dd, *J*=13.9, 1.6 Hz, 1H), 3.86 (t, *J*=8.4 Hz, 1H), 3.58 (d, *J*=13.0 Hz, 1H), 3.33 – 3.12 (m, 1H), 2.84 (d, *J*=8.3 Hz, 2H), 2.66 (dd, *J*=11.3, 8.9 Hz, 1H), 2.43 (s, 3H), 1.76 (s, 3H) ppm; ^{13}C NMR (101 MHz, $CDCl_3$) δ = 143.6, 141.2, 134.0, 133.6, 132.9, 132.0, 131.0, 130.7, 129.8, 129.4, 127.7, 127.4, 118.6, 117.4, 112.7, 52.4, 49.5, 40.9, 36.3, 22.6, 21.5 ppm; HR-ESI-MS (*m/z*): calcd. for $C_{23}H_{23}N_2O_2S$ $[M+H]^+$, 391.1480, found 391.1471.

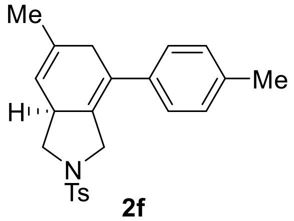

2f

2f, 6 mg, 15 % yield, 88% *ee* (AD-H, Hexane:*i*-PrOH = 90:10, 0.6 mL/min, 254 nM, t1 = 24.0 min, t2 = 28.3 min), $[\alpha]_D^{25}$ = -18.7 in $CHCl_3$; ^{1}H-NMR (400 MHz, $CDCl_3$) δ 7.64 (d, *J* = 8.3 Hz, 2H), 7.26 (d, *J* = 7.8 Hz, 2H), 7.14 (d, *J* = 7.9 Hz, 2H), 7.03 (d, *J* = 8.2 Hz, 2H), 5.41 (s, 1H), 4.20 (ddd, *J* = 13.9, 7.9, 1.4 Hz, 1H), 3.90 – 3.75 (m, 1H), 3.69 (d, *J* = 13.9 Hz, 1H), 3.30 – 3.14 (m, 1H), 2.93 – 2.78 (m, 2H), 2.62 (dd, *J* = 11.4, 8.8 Hz, 1H), 2.40 (s, 3H), 2.36 (s, 3H), 1.74 (s, 3H) ppm; ^{13}C-NMR (101 MHz, $CDCl_3$) δ 143.33, 136.92, 134.55, 130.06, 129.82, 129.69, 129.08, 127.85, 127.63, 127.48, 127.24, 117.47, 52.47, 50.04, 40.94, 36.41, 22.77, 21.55, 21.20 ppm; HR-ESI-MS (*m/z*): calcd. for $C_{23}H_{26}NO_2S$ $[M+H]^+$, 380.1684, found 380.1677.

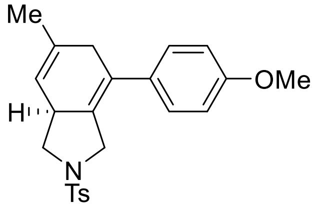

2g

2g, 25 mg, 64% yield, 78% *ee* (AS-H, Hexane:*i*-PrOH = 85:15, 0.8 mL/min, 254 nM, t1 = 24.0 min, t2 = 28.3 min), $[\alpha]_D^{25}$ = -15.9 in $CHCl_3$; ^{1}H-NMR (400 MHz, $CDCl_3$) δ 7.57 (d, *J* = 8.3 Hz, 2H), 7.19 (d, *J* = 8.1 Hz, 2H), 7.00 (d, *J* = 8.8 Hz, 2H), 6.80 (d, *J* = 8.8 Hz, 2H), 5.34 (s, 1H), 4.22 – 4.06 (m, 1H), 3.79 (s, 1H), 3.75 (s, 3H), 3.61 (d, *J* = 13.8 Hz, 1H), 3.25 – 3.04 (m, 1H), 2.78 (dd, *J* = 23.4, 8.5 Hz, 2H), 2.53 (dd, *J* = 11.4, 8.8 Hz, 1H), 2.33 (s, 3H), 1.67 (s, 3H) ppm; ^{13}C-NMR (101 MHz, $CDCl_3$) δ 157.7, 142.2, 133.4, 132.9, 131.1, 128.6, 128.5, 127.9, 127.4, 126.4, 116.4, 112.6, 54.2, 51.3, 49.0, 39.8, 35.3, 21.7, 20.4 ppm; HR-ESI-MS (*m/z*): calcd. for $C_{23}H_{26}NO_3S$ $[M+H]^+$, 396.1633, found 396.1626.

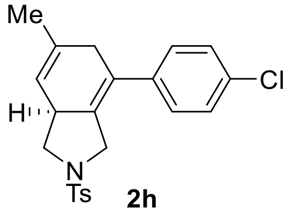

2h

2h, 17 mg, 43% yield, 90% *ee* (AD-H, Hexane:*i*-PrOH = 95:5, 0.5 mL/min, 254 nM, t1 = 24.0 min, t2 = 28.3 min), $[\alpha]_D^{25}$ = 1.9 in $CHCl_3$; ^{1}H NMR (400 MHz, $CDCl_3$) δ = 7.64 (d, *J*=8.2 Hz, 2H), 7.33 – 7.29 (m, 2H), 7.29 – 7.25 (m, 2H), 7.10 – 7.04 (m, 2H), 5.42 (s, 1H), 4.16 (dd, *J*=13.9, 1.4 Hz, 1H), 3.84 (t, *J*=8.3 Hz, 1H), 3.61 (d, *J*=13.8 Hz, 1H), 3.32 – 3.11 (m, 1H), 2.84 (t, *J*=6.6 Hz, 2H), 2.61 (dd, *J*=11.4, 8.8 Hz, 1H), 2.41 (s, 3H), 1.75 (s, 3H) ppm; ^{13}C NMR (101 MHz, $CDCl_3$) δ = 143.4, 138.2, 134.3, 133.8, 133.2, 131.3, 129.7, 128.7, 128.6, 128.4, 127.4, 117.4, 52.3, 49.8, 40.9, 36.3, 22.7, 21.5 ppm; HR-ESI-MS (*m/z*): calcd. for $C_{22}H_{23}NO_2SCl$ $[M+H]^+$, 400.1138, found 400.1127.

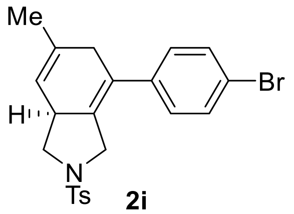

2i

2i, 27 mg, 60% yield, 94% *ee* (AD-H, Hexane:*i*-PrOH = 90:10, 0.4 mL/min, 254 nM, t1 = 24.0 min, t2 = 28.3 min), $[\alpha]_D^{25}$ = 18.4 in $CHCl_3$; ^{1}H NMR (400 MHz, $CDCl_3$) δ = 7.57 (d, *J*=8.2 Hz, 2H), 7.45 – 7.35 (m, 2H), 7.20 (d, *J*=8.7 Hz, 2H), 6.94 (d, *J*=8.4 Hz, 2H), 5.34 (s, 1H), 4.09 (d, *J*=13.9 Hz, 1H), 3.77 (t, *J*=8.3 Hz, 1H), 3.54 (d, *J*=13.7 Hz, 1H), 3.28 – 3.00 (m, 1H), 2.84 – 2.62 (m, 2H), 2.54 (dd, *J*=11.4, 8.8 Hz, 1H), 2.34 (s, 3H), 1.67 (s, 3H) ppm; ^{13}C NMR (101 MHz, $CDCl_3$) δ = 143.4, 138.7, 134.3, 133.7, 131.5, 131.4, 129.7, 129.0, 128.5, 127.4, 121.4, 117.4, 52.3, 49.8, 41.0, 36.2, 22.7, 21.5 ppm; HR-ESI-MS (*m/z*): calcd. for $C_{22}H_{23}NO_2SBr$ $[M+ H]^+$, 444.0633, found 444.0618.

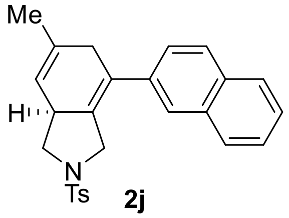

2j

2j, 10 mg, 23% yield, 55% *ee* (AD-H, Hexane:*i*-PrOH = 95:5, 0.5 mL/min, 254 nM, t1 = 68.2 min, t2 = 79.5 min), ^{1}H NMR (400 MHz, $CDCl_3$) δ = 7.88 – 7.77 (m, 2H), 7.64 – 7.57 (m, 3H), 7.51 (dt, *J*=5.5, 3.3 Hz, 2H), 7.30 – 7.19 (m, 4H), 5.46 (s, 1H), 4.36 – 4.23 (m, 1H), 3.87 (t, *J*=8.3 Hz, 1H), 3.66 (d, *J*=13.9 Hz, 1H), 3.31 (s, 1H), 3.04 (dd, *J*=21.4, 6.6 Hz, 1H), 2.91 (dd, *J*=21.5, 10.3 Hz, 1H), 2.63 (dd, *J*=11.4, 8.7 Hz, 1H), 2.39 (s, 3H), 1.78 (s, 3H) ppm; ^{13}C NMR (101 MHz, $CDCl_3$) δ = 143.3, 137.4, 134.5, 133.7, 133.1, 132.6, 131.1, 129.7, 129.5, 128.0, 128.0, 127.6, 127.5, 126.4, 126.3, 126.1, 125.4, 117.5, 52.5, 50.0, 41.0, 36.5, 25.3, 22.7, 21.5 ppm.

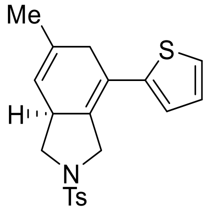

2k

2k, 10 mg, 27% yield, 92% *ee* (IB, Hexane:*i*-PrOH = 90:10, 0.5 mL/min, 254 nM, t1 = 24.0 min, t2 = 28.3 min), $[\alpha]_D^{25}$ = -15.8 in $CHCl_3$; ^{1}H-NMR (400 MHz, $CDCl_3$) δ 7.64 (d, *J* = 8.3 Hz, 2H), 7.24-7.18 (m, 3H), 6.99 (dd, *J* = 5.1, 3.7 Hz, 1H), 6.86 (d, *J* = 3.7 Hz, 1H), 5.36 (s, 1H), 4.43–4.16 (m, 1H), 3.91 (dd, *J* = 14.6, 1.7 Hz, 1H), 3.83–3.76 (m, 1H), 3.35–3.12 (m, 1H), 2.89 (d, *J* = 8.1 Hz, 2H), 2.47 (dd, *J* = 11.7, 8.6 Hz, 1H), 2.33 (s, 3H), 1.69 (s, 3H) ppm; ^{13}C-NMR (101 MHz, $CDCl_3$) δ 142.5, 141.2, 133.1, 132.2, 129.4, 128.8, 128.7, 126.7, 126.5, 126.2, 123.7, 123.6, 120.9, 116.3, 51.1, 49.7, 40.3, 35.1, 21.6, 20.4 ppm; HR-ESI-MS (*m/z*): calcd. for $C_{20}H_{22}NO_2S_2$ $[M+H]^+$, 372.1092, found 372.1078.

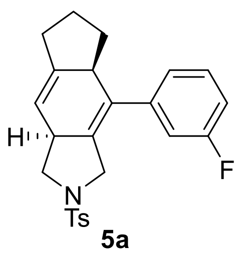

5a

5a, 31 mg, 72% yield, 99% *ee* (OD, Hexane:*i*-PrOH = 97:3, 0.5 mL/min, 230 nM, t1 = 45.7 min, t2 = 49.8 min), $[\alpha]_D^{25}$ = 0.3 in $CHCl_3$; ^{1}H NMR (400 MHz, $CDCl_3$) δ = 7.62 (d, *J*=8.2, 2H), 7.30 (dd, *J*=7.9, 2.0, 1H), 7.25 (d, *J*=7.5, 2H), 6.98 (dd, *J*=8.5, 2.6, 1H), 6.86 (d, *J*=7.7, 1H), 6.82 – 6.70 (m, 1H), 5.40 (s, 1H), 4.04 (d, *J*=13.2, 1H), 3.82 – 3.73 (m, 1H), 3.69 (d, *J*=13.2, 1H), 3.32 – 3.07 (m, 2H), 2.68 (d, *J*=8.6, 1H), 2.42 (s, 3H), 2.04 – 1.99 (m, 1H), 1.84 (td, *J*=11.4, 6.0, 1H), 1.67 (ddd, *J*=12.2, 8.2, 4.7, 2H), 1.16 – 1.03 (m, 1H), 0.86 (dt, *J*=11.7, 4.8, 1H); ^{13}C NMR (101 MHz, $CDCl_3$) δ = 145.1, 143.4, 134.7, 134.2, 131.9, 131.7, 129.8, 129.7, 129.7, 127.6, 127.3, 123.4, 114.5, 114.3, 114.1, 113.9, 111.9, 52.1, 49.6, 44.0, 42.6, 30.4, 29.0, 21.5, 20.8.

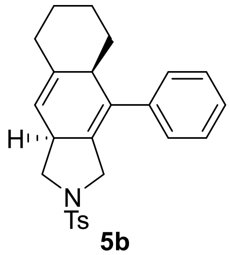

5b

5b, 19 mg, 48 % yield, 90% *ee* (AD-H, Hexane:*i*-PrOH = 90:10, 1.0 mL/min, 254 nM, t1 = 24.0 min, t2 = 28.3 min), $[\alpha]_D^{25}$ = +7.6 in $CHCl_3$; ^{1}H NMR (400 MHz, $CDCl_3$) δ = 7.61 (d, *J*=8.2 Hz, 2H), 7.40 – 7.22 (m, 6H), 7.11 – 7.04 (m, 2H), 5.37 (s, 1H), 4.11 (t, *J*=9.9 Hz, 1H), 3.79 (t, *J*=8.3 Hz, 1H), 3.55 (d, *J*=13.6 Hz, 1H), 3.32 – 3.13 (m, 1H), 2.92 (d, *J*=6.6 Hz, 1H), 2.61 (dd, *J*=11.4, 8.8 Hz, 1H), 2.41 (s, 3H), 2.27 (d, *J*=12.8 Hz, 1H), 2.01 (d, *J*=12.3 Hz, 1H), 1.91 – 1.78 (m, 1H), 1.69 (t, *J*=15.6 Hz, 2H), 1.32 – 1.35 (m, 2H), 1.12 – 0.93 (m, 1H); ^{13}C NMR (101 MHz, $CDCl_3$) δ = 143.2, 142.7, 139.1, 134.1, 134.0, 130.1, 129.7, 128.4, 127.7, 127.4, 127.4, 113.7, 52.6, 50.0, 44.2, 40.1, 35.8, 34.0, 28.9, 27.1, 21.5; HR-ESI-MS (*m/z*): calcd. for $C_{25}H_{28}NO_2S$ $[M+H]^+$, 406.1841, found 406.1834.

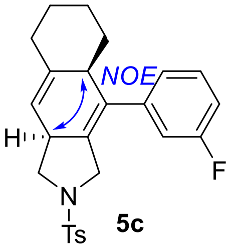

5c

5c, 40 mg, 89 % yield, 95% *ee* (AD-H, Hexane:*i*-PrOH = 95:5, 0.5 mL/min, 254 nM, t1 = 24.0 min, t2 = 28.3 min), $[\alpha]_D^{25}$ = 26.2 in $CHCl_3$; ^{1}H NMR (400 MHz, $CDCl_3$) δ = 7.61 (d, *J*=8.2 Hz, 2H), 7.28 (m, 3H), 6.98 (td, *J*=8.4, 1.9 Hz, 1H), 6.85 (d, *J*=7.7 Hz, 1H), 6.77 (dd, *J*=9.8, 1.8 Hz, 1H), 5.37 (s, 1H), 4.08 (d, *J*=13.7, 1H), 3.80 (t, *J*=8.3 Hz, 1H), 3.53 (d, *J*=13.7 Hz, 1H), 3.22 (d, *J*=8.3 Hz, 1H), 3.00 – 2.77 (m, 1H), 2.61 (dd, *J*=11.4, 8.8 Hz, 1H), 2.41 (s, 3H), 2.27 (d, *J*=12.9, 1H), 2.17 – 1.94 (m, 1H), 1.84 (d, *J*=11.7 Hz, 1H), 1.73 (d, *J*=12.1 Hz, 1H), 1.69 – 1.62 (m, 1H), 1.46 – 1.18 (m, 2H), 1.05 (tt, *J*=12.3, 6.0 Hz, 1H) ppm; ^{13}C NMR (101 MHz, $CDCl_3$) δ = 163.9, 161.5, 143.4, 142.5, 141.5, 141.4, 133.9, 133.0, 131.0, 129.9, 129.8, 129.7, 127.4, 123.7, 123.6, 114.5, 114.4, 114.2, 114.2, 113.5, 52.5, 49.8, 44.2, 40.1, 35.6, 33.9, 28.9, 27.0, 21.5 ppm; HR-ESI-MS (*m/z*): calcd. for $C_{25}H_{26}FO_2SNa$ $[M+Na]^+$, 446.0601, found 446.0602.

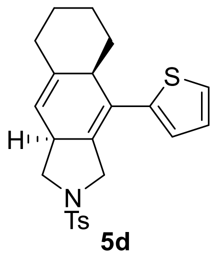

5d

5d, 35 mg, 86 % yield, 97% *ee* (OD-H, Hexane:*i*-PrOH = 95:5, 0.4 mL/min, 254 nM, t1 = 24.0 min, t2 = 28.3 min), $[\alpha]_D^{25}$ = 56.2 in $CHCl_3$; ^1H NMR (400 MHz, $CDCl_3$) δ = 7.67 (d, *J*=8.2 Hz, 2H), 7.34 – 7.22 (m, 3H), 7.04 (dd, *J*=5.1, 3.6 Hz, 1H), 6.85 (d, *J*=3.0 Hz, 1H), 5.37 (s, 1H), 4.26 (d, *J*=14.3 Hz, 1H), 3.87 (d, *J*=14.3 Hz, 1H), 3.82 (t, *J*=8.2 Hz, 1H), 3.27 (dd, *J*=11.3, 5.4 Hz, 1H), 2.95 – 2.84 (m, 1H), 2.54 (dd, *J*=11.6, 8.6 Hz, 1H), 2.40 (s, 3H), 2.27 (d, *J*=12.6 Hz, 1H), 2.03 – 1.94 (m, 2H), 1.87 (d, *J*=11.2 Hz, 1H), 1.79 (d, *J*=13.3 Hz, 1H),1.48 (ddd, *J*=13.0, 5.4, 2.5 Hz, 1H), 1.17 – 1.08 (m, 2H) ppm; ^{13}C NMR (101 MHz, $CDCl_3$) δ = 143.4, 142.6, 141.4, 133.7, 130.6, 129.7, 127.5, 127.2, 126.8, 125.9, 124.5, 113.4, 52.0, 50.5, 45.3, 40.4, 35.6, 34.4, 29.1, 27.1, 21.5 ppm; HR-ESI-MS (*m/z*): calcd. for $C_{23}H_{26}NO_2S_2$ $[M+ H]^+$, 412.1405, found 412.1397.

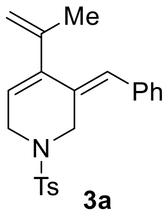

3a

3a, ^{1}H-NMR (400 MHz, $CDCl_3$) δ 7.58 – 7.53 (m, 2H), 7.42 – 7.35 (m, 2H), 7.32 – 7.28 (m, 1H), 7.27 – 7.22 (m, 2H), 7.21 – 7.16 (m, 2H), 6.41 (s, 1H), 5.53 (dd, *J* = 3.8, 3.2 Hz, 1H), 4.97 (dq, *J* = 2.9, 1.4 Hz, 1H), 4.65 (dd, *J* = 2.4, 0.8 Hz, 1H), 4.23 (d, *J* = 1.4 Hz, 2H), 3.94 (d, *J* = 3.4 H z, 2H), 2.41 (s, 3H), 1.84 – 1.71 (s, 3H) ppm; ^{13}C NMR (101 MHz, $CDCl_3$) δ = 143.6, 143.4, 142.1, 136.2, 134.3, 129.4, 128.9, 128.9, 128.6, 128.5, 127.7, 127.3, 120.7, 115.6, 45.3, 44.7, 23.0, 21.5 ppm.

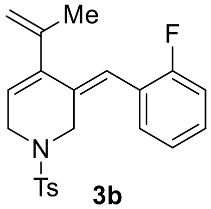

3b

3b, ^{1}H NMR (400 MHz, $CDCl_3$) δ = 7.56 (d, *J*=8.3, 2H), 7.33 – 7.28 (m, 1H), 7.30 (m, 1H), 7.24 (s, 1H), 7.18 (dd, *J*=4.5, 3.5, 2H), 7.14 – 7.05 (m, 1H), 6.39 (s, 1H), 5.55 (t, *J*=3.6, 1H), 4.98 (dd, *J*=2.2, 1.5, 1H), 4.64 (d, *J*=1.4, 1H), 4.12 (s, 2H), 3.95 (d, *J*=3.6, 2H), 2.41 (s, 3H), 1.77 (s, 3H) ppm; ^{13}C NMR (101 MHz, $CDCl_3$) δ = 161.3, 158.8, 143.5, 143.3, 141.8, 134.3, 130.6, 130.5, 130.5, 129.5, 129.4, 129.3, 127.6, 124.1, 124.0, 124.0, 123.8, 121.4, 121.4, 121.3, 115.8, 115.8, 115.5, 45.3, 45.0, 44.9, 22.9, 21.5 ppm.

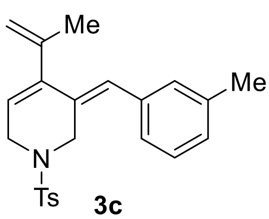

3c

3c, ^{1}H-NMR (400 MHz, $CDCl_3$) δ 7.59 (d, *J* = 8.3Hz, 2H), 7.38 – 7.21 (m, 3H), 7.13 (d, *J* = 7.6 Hz, 1H), 7.03 (d, *J* = 7.5 Hz, 2H), 6.41 (s, 1H), 5.55 (t, *J* = 3.6 Hz, 1H), 4.99 (dd, *J* = 2.4, 1.5 Hz, 1H), 4.78 – 4.59 (m, 1H), 4.26 (d, *J* = 1.3 Hz, 2H), 3.97 (d, *J* = 3.6 Hz, 2H), 2.45 (s, 3H), 2.42 (s, 3H), 1.80 (s, 3H) ppm; ^{13}C-NMR (101 MHz,

$CDCl_3$) δ 143.6, 143.4, 142.2, 138.0, 136.1, 134.4, 129.7, 129.4, 128.7, 128.4, 128.1, 127.7, 125.9, 120.5, 115.5, 45.3, 44.8, 23.0, 21.5, 21.5 ppm.

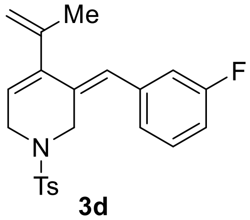

3d

3d, ^{1}H-NMR (400 MHz, $CDCl_3$) δ 7.59 (d, *J* = 8.3 Hz, 2H), 7.38 (td, *J* = 8.0, 6.1 Hz, 1H), 7.33 – 7.23 (m, 2H), 7.11 – 6.96 (m, 2H), 6.88 (dd, *J* = 10.0, 1.7 Hz, 1H), 6.39 (s, 1H), 5.61 (t, *J* = 3.6 Hz, 1H), 5.01 (dd, *J* = 2.2, 1.5 Hz, 1H), 4.68 (d, *J* = 1.4 Hz, 1H), 4.22 (d, *J* = 1.3 Hz, 2H), 3.97 (d, *J* = 3.6 Hz, 2H), 2.44 (s, 3H), 1.80 (s, 3H) ppm; ^{13}C-NMR (101 MHz, $CDCl_3$) δ 163.9, 161.5, 143.6, 143.3, 141.8, 138.4, 138.3, 134.2, 130.1, 130.0, 130.0, 129.4, 127.7, 127.2, 127.2, 124.6, 124.6, 121.5, 115.8, 115.8, 115.5, 114.3, 114.1, 45.2, 44.6, 22.9, 21.5 ppm.

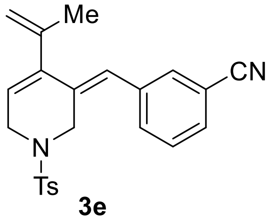

3e

3e, ^{1}H NMR (400 MHz, $CDCl_3$) δ = 7.49 (d, *J*=8.3 Hz, 3H), 7.44 (t, *J*=7.7 Hz, 1H), 7.40 – 7.32 (m, 2H), 7.25 – 7.19 (m, 2H), 6.31 (s, 1H), 5.57 (t, *J*=3.6 Hz, 1H), 4.95 (dd, *J*=2.1, 1.6 Hz, 1H), 4.63 (d, *J*=1.3 Hz, 1H), 4.04 (d, *J*=1.2 Hz, 2H), 3.86 (d, *J*=3.6 Hz, 2H), 2.36 (s, 3H), 1.71 (s, 3H) ppm; ^{13}C NMR (101 MHz, $CDCl_3$) δ = 142.7, 141.9, 140.4, 136.4, 132.9, 132.0, 131.3, 130.4, 129.6, 128.5, 128.4, 126.6, 124.8, 121.3, 117.5, 115.1, 111.7, 44.2, 43.4, 21.9, 20.5 ppm.

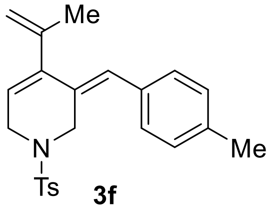

3f

3f, ^{1}H-NMR (400 MHz, $CDCl_3$) δ 7.58 (d, *J* = 8.3 Hz, 2H), 7.34 – 7.25 (m, 2H), 7.23 (d, *J* = 7.9 Hz, 2H), 7.11 (d, *J* = 8.0 Hz, 2H), 6.40 (s, 1H), 5.53 (t, *J* = 3.6 Hz, 1H), 4.99 (dd, *J* = 2.4 Hz, 1.5, 1H), 4.66 (d, *J* = 1.5 Hz, 1H), 4.27 (d, *J* = 1.3 Hz, 2H), 3.96

(d, J = 3.6 Hz, 2H), 2.44 (s, 3H), 2.41 (s, 3H), 1.79 (s, 3H) ppm; ^{13}C-NMR (101 MHz, $CDCl_3$) δ 143.7, 143.4, 142.2, 137.2, 134.4, 133.3, 129.3, 129.2, 128.8, 128.6, 128.2, 127.7, 120.2, 115.5, 45.3, 44.8, 23.0, 21.5, 21.2 ppm.

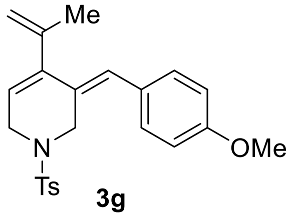

3g

3g, ^{1}H-NMR (400 MHz, $CDCl_3$) δ 7.59 (d, J = 8.3 Hz, 2H), 7.34 – 7.23 (m, 2H), 7.16 (d, J = 8.6 Hz, 2H), 7.00 – 6.91 (m, 2H), 6.37 (s, 1H), 5.51 (t, J = 3.6 Hz, 1H), 4.98 (dd, J = 2.4, 1.5 Hz, 1H), 4.65 (d, J = 1.6 Hz, 1H), 4.26 (d, J = 1.2 Hz, 2H), 3.96 (d, J = 3.6 Hz, 2H), 3.88 (s, 3H), 2.44 (s, 3H), 1.79 (s, 3H) ppm; ^{13}C-NMR (101 MHz, $CDCl_3$) δ 158.8, 143.7, 143.4, 142.2, 134.4, 130.3, 129.3, 128.7, 128.2, 127.7, 127.5, 119.9, 115.4, 114.0, 55.3, 45.2, 44.7, 23.0, 21.5 ppm.

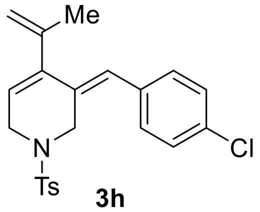

3h

3h, ^{1}H NMR (400 MHz, $CDCl_3$) δ = 7.56 (d, J=8.3 Hz, 2H), 7.42 – 7.31 (m, 2H), 7.33 – 7.22 (m, 2H), 7.12 (d, J=8.4 Hz, 2H), 6.36 (s, 1H), 5.56 (t, J=3.6 Hz, 1H), 4.98 (dd, J=2.2, 1.5 Hz, 1H), 4.66 (d, J=1.4 Hz, 1H), 4.16 (d, J=1.2 Hz, 2H), 3.92 (d, J=3.6 Hz, 2H), 2.41 (s, 3H), 1.77 (s, 3H) ppm; ^{13}C NMR (101 MHz, $CDCl_3$) δ = 143.5, 143.3, 141.9, 134.6, 134.2, 133.1, 130.2, 129.7, 129.4, 128.7, 127.7, 127.2, 121.2, 115.8, 45.2, 44.5, 23.0, 21.5 ppm.

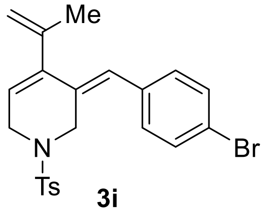

3i

3i, ^{1}H NMR (400 MHz, $CDCl_3$) δ = 7.56 (d, J=8.2 Hz, 2H), 7.51 (d, J=8.4 Hz, 2H), 7.25 (d, J=8.6 Hz, 2H), 7.05 (d, J=8.3 Hz, 2H), 6.33 (s, 1H), 5.57 (s, 1H), 4.98 (s, 1H), 4.66 (d, J=1.3 Hz, 1H), 4.15 (d, J=1.0 Hz, 2H), 3.92 (d, J=3.6 Hz, 2H), 2.42 (s, 3H),

1.77 (s, 3H) ppm; ^{13}C NMR (101 MHz, $CDCl_3$) δ = 143.6, 143.3, 141.9, 135.1, 134.2, 131.6, 130.5, 129.7, 129.4, 127.7, 127.2, 121.3, 121.2, 115.8, 45.3, 44.5, 23.0, 21.5 ppm;

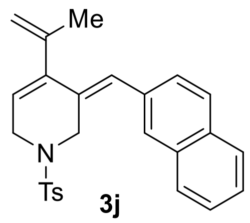

3j

3j, ^{1}H NMR (400 MHz, $CDCl_3$) δ = 7.77 (dd, *J*=8.9, 3.3 Hz, 3H), 7.57 (s, 1H), 7.47 (d, *J*=8.2 Hz, 2H), 7.45 – 7.40 (m, 2H), 7.24 (dd, *J*=8.5, 1.5 Hz, 1H), 7.20 – 7.09 (m, 3H), 6.48 (s, 1H), 5.49 (t, *J*=3.6 Hz, 1H), 4.93 (dd, *J*=2.3, 1.5 Hz, 1H), 4.62 (d, *J*=1.5 Hz, 1H), 4.24 (d, *J*=1.1 Hz, 2H), 3.89 (d, *J*=3.6 Hz, 2H), 2.33 (s, 3H), 1.74 (s, 3H) ppm; ^{13}C NMR (101 MHz, $CDCl_3$) δ = 143.6, 143.4, 142.2, 134.3, 133.7, 133.3, 132.4, 129.4, 129.3, 128.6, 128.1, 128.0, 128.0, 127.7, 127.7, 126.9, 126.4, 126.2, 120.9, 115.7, 45.4, 44.9, 23.0, 21.5 ppm;

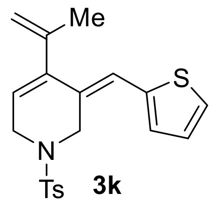

3k

3k, ^{1}H-NMR (400 MHz, $CDCl_3$) δ 7.67 (d, *J* = 8.3 Hz, 2H), 7.40 (dd, *J* = 5.1, 0.7 Hz, 1H), 7.35 – 7.24 (m, 2H), 7.10 (dd, *J* = 5.1, 3.6 Hz, 1H), 7.03 (d, *J* = 3.6 Hz, 1H), 6.51 (s, 1H), 5.57 (t, *J* = 3.9 Hz, 1H), 5.01 (dd, *J* = 2.2 Hz, 1.5, 1H), 4.66 (d, *J* = 1.4 Hz, 1H), 4.39 (d, *J* = 1.4 Hz, 2H), 3.95 (d, *J* = 3.8 Hz, 2H), 2.43 (s, 3H), 1.78 (s, 3H) ppm; ^{13}C-NMR (101 MHz, $CDCl_3$) δ 143.5, 143.4, 141.9, 139.2, 134.1, 129.5, 128.5, 127.7, 127.5, 127.3, 126.6, 120.8, 120.3, 115.7, 45.1, 44.8, 23.2, 21.5 ppm.

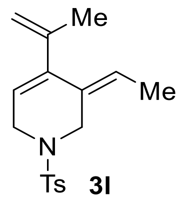

3l

3l, ^{1}H-NMR (400 MHz, $CDCl_3$) δ 7.74 – 7.51 (m, 2H), 7.21 (d, *J* = 8.5 Hz, 2H), 5.55 – 5.34 (m, 1H), 5.28 (t, *J* = 3.7 Hz, 1H), 4.82 (dq, *J* = 2.9 Hz, 1.4, 1H), 4.53 (dd, *J* =

2.4, 0.8 Hz, 1H), 3.85 (s, 2H), 3.73 (d, *J* = 3.7 Hz, 2H), 2.34 (s, 3H), 1.67 (dd, *J* = 7.2, 0.5 Hz, 3H), 1.63 (dd, *J* = 1.3, 0.9 Hz, 3H) ppm; ^{13}C-NMR (101 MHz, $CDCl_3$) δ 143.7, 143.4, 141.8, 134.0, 129.4, 128.3, 127.7, 123.6, 118.0, 114.9, 45.1, 43.8, 29.7, 22.9, 21.5, 13.3 ppm.

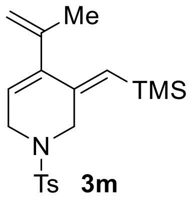

3m

3m,^{1}H-NMR (400 MHz, $CDCl_3$) δ 7.69 (d, *J* = 8.2 Hz, 2H), 7.32 (d, *J* = 8.1 Hz, 2H), 5.58 (s, 1H), 5.54 (t, *J* = 3.7 Hz, 1H), 4.97 (dd, *J* = 2.1 Hz, 1.5, 1H), 4.68 (d, *J* = 1.5 Hz, 1H), 3.94 (s, 2H), 3.81 (d, *J* = 3.7 Hz, 2H), 2.44 (s, 3H), 1.73 (s, 3H), 0.31 – 0.16 (m, 9H) ppm.^{13}C-NMR (101 MHz, $CDCl_3$) δ = 143.67, 143.59, 142.97, 142.72, 133.67, 129.59, 128.52, 127.80, 120.74, 115.39, 77.38, 77.06, 76.74, 48.22, 45.18, 22.93, 21.52, 0.02, -1.18 ppm.

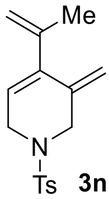

3n

3n, ^{1}H-NMR (400 MHz, $CDCl_3$) δ 7.70 (d, *J* = 8.3 Hz, 2H), 7.40 – 7.26 (m, 2H), 5.53 (dd, *J* = 3.4 Hz, 2.5, 1H), 5.06 (d, *J* = 6.3 Hz, 2H), 5.04 – 4.88 (m, 1H), 4.72 (d, *J* = 1.2 Hz, 1H), 3.87 (s, 2H), 3.84 (d, *J* = 3.7 Hz, 2H), 2.44 (s, 3H), 1.78 (s, 3H) ppm; ^{13}C-NMR (101 MHz, $CDCl_3$) δ 143.5, 142.7, 140.6, 135.6, 133.6, 129.5, 127.8, 120.9, 115.2, 113.6, 49.5, 45.4, 22.7, 21.5 ppm.

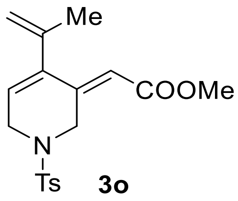

3o

3o,^{1}H-NMR (400 MHz, $CDCl_3$) δ 7.71 (d, *J* = 8.3 Hz, 2H), 7.42 – 7.06 (m, 2H), 5.90 (t, *J* = 3.9 Hz, 1H), 5.75 (s, 1H), 5.14 – 4.94 (m, 1H), 4.69 (d, *J* = 0.9 Hz, 1H), 4.56 (d, *J* = 1.6 Hz, 2H), 3.90 (d, *J* = 3.9 Hz, 2H), 3.77 (s, 3H), 2.43 (s, 3H), 1.75 (s, 3H) ppm;

^{13}C-NMR (101 MHz, $CDCl_3$) δ 166.7, 145.1, 143.7, 142.2, 141.0, 133.7, 129.7, 127.8, 127.7, 127.6, 116.5, 115.6, 51.2, 44.8, 44.7, 22.9, 21.5 ppm.

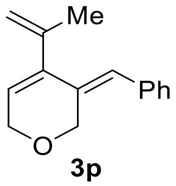

3p

3p, ^{1}H-NMR (400 MHz, $CDCl_3$) δ 7.38 (dd, *J* = 10.4, 4.6 Hz, 2H), 7.34 – 7.23 (m, 1H), 7.18 (d, *J* = 7.3 Hz, 2H), 6.57 (s, 1H), 5.80 (t, *J* = 3.1 Hz, 1H), 5.14 (dd, *J* = 2.3, 1.5 Hz, 1H), 5.03 (d, *J* = 1.5 Hz, 1H), 4.63 (d, *J* = 1.3 Hz, 2H), 4.34 (d, *J* = 3.1 Hz, 2H), 2.00 (s, 3H) ppm; ^{13}C-NMR (101 MHz, $CDCl_3$) δ 143.7, 140.6, 136.6, 132.0, 129.1, 128.2, 126.9, 126.2, 124.1, 115.4, 65.8, 65.2, 23.1 ppm.

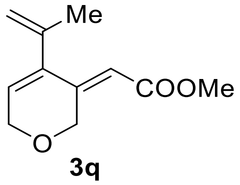

3q

3q, ^{1}H-NMR (400 MHz, $CDCl_3$) δ 6.07 (s, 1H), 5.76 (d, *J* = 0.6 Hz, 1H), 5.13 – 5.00 (m, 1H), 4.92 (d, *J* = 1.6 Hz, 2H), 4.90 – 4.85 (m, 1H), 4.26 (d, *J* = 3.4 Hz, 2H), 1.88 (d, *J* = 0.7 Hz, 3H) ppm; ^{13}C-NMR (101 MHz, $CDCl_3$) δ 166.9, 148.3, 142.3, 139.6, 131.1, 116.2, 113.0, 65.7, 64.9, 51.3, 23.1 ppm.

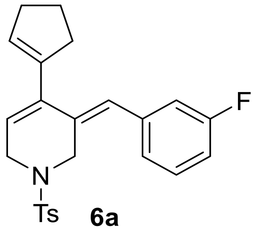

6a

6a, ^{1}H NMR (400 MHz, $CDCl_3$) δ = 7.48 (d, *J*=8.2 Hz, 2H), 7.31 (t, *J*=7.5 Hz, 2H), 7.24 – 7.14 (m, 3H), 7.11 (d, *J*=7.5 Hz, 2H), 6.50 (s, 1H), 5.53 (s, 1H), 5.48 (t, *J*=3.7 Hz, 1H), 5.22 (s, 1H), 4.10 (s, 2H), 3.86 (d, *J*=3.6 Hz, 2H), 2.33 (s, 3H), 2.32 – 2.24 (m, 4H), 1.86 – 1.76 (m, 2H) ppm; ^{13}C NMR (101 MHz, $CDCl_3$) δ = 143.4, 141.0, 136.5, 136.3, 134.1, 129.9, 129.6, 129.5, 129.4, 128.9, 128.8, 128.4, 128.1, 127.7, 127.2, 120.6, 45.4, 44.6, 35.5, 33.1, 23.3, 21.5 ppm.

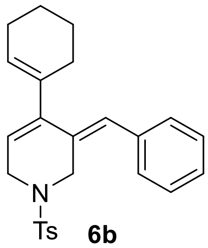

6b

6b, ^{1}H-NMR (400 MHz, $CDCl_3$) δ 7.59 (d, *J* = 8.2 Hz, 2H), 7.41 (t, *J* = 7.5 Hz, 2H), 7.29 (t, *J* = 7.1 Hz, 3H), 7.21 (d, *J* = 7.3 Hz, 2H), 6.41 (s, 1H), 5.50 (t, *J* = 3.7 Hz, 1H), 5.45 – 5.27 (m, 1H), 4.23 (d, *J* = 1.0 Hz, 2H), 3.94 (d, *J* = 3.7 Hz, 2H), 2.45 (s, 3H), 2.08 (s, 2H), 1.96 (d, *J* = 1.5 Hz, 2H), 1.71 – 1.50 (m, 4H) ppm; ^{13}C-NMR (101 MHz, $CDCl_3$) δ 143.4, 142.5, 136.7, 136.4, 134.3, 129.6, 129.4, 128.9, 128.5, 128.2, 127.7, 127.2, 126.7, 120.5, 45.3, 44.8, 28.9, 25.2, 22.8, 22.1, 21.5 ppm.

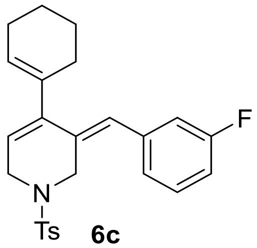

6c

6c, ^{1}H NMR (400 MHz, $CDCl_3$) δ = 7.49 (d, *J*=8.2 Hz, 2H), 7.27 (td, *J*=7.9, 6.2 Hz, 1H), 7.21 – 7.12 (m, 2H), 6.96 – 6.84 (m, 2H), 6.83 – 6.72 (m, 1H), 6.25 (s, 1H), 5.44 (t, *J*=3.6 Hz, 1H), 5.35 – 5.19 (m, 1H), 4.08 (d, *J*=1.1 Hz, 2H), 3.84 (d, *J*=3.6 Hz, 2H), 2.34 (s, 3H), 1.97 (s, 2H), 1.84 (d, *J*=1.4 Hz, 2H), 1.59 – 1.42 (m, 4H) ppm; ^{13}C NMR (101 MHz, $CDCl_3$) δ = 163.9, 161.5, 143.5, 142.2, 138.6, 138.5, 136.4, 134.2, 130.7, 130.0, 129.9, 129.7, 129.4, 127.7, 127.4, 126.9, 126.8, 126.8, 124.7, 124.6, 121.3, 115.8, 115.6, 114.2, 114.0, 45.3, 44.7, 28.9, 25.2, 22.8, 22.0, 21.5 ppm.

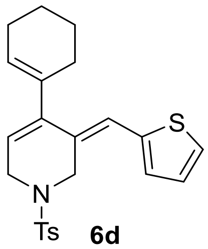

6d

6d, ^{1}H NMR (400 MHz, $CDCl_3$) δ = 7.64 (d, *J*=8.3 Hz, 2H), 7.35 (d, *J*=4.6 Hz, 1H), 7.30 – 7.23 (m, 2H), 7.07 (dd, *J*=5.1, 3.6 Hz, 1H), 6.98 (d, *J*=3.5 Hz, 1H), 6.44 (s, 1H), 5.48 (t, *J*=3.8 Hz, 1H), 5.38 – 5.15 (m, 1H), 4.33 (d, *J*=1.3 Hz, 2H), 3.89 (d, *J*=3.8 Hz,

2H), 2.41 (s, 3H), 2.05 (s, 2H), 1.88 (d, *J*=1.6 Hz, 2H), 1.75 – 1.52 (m, 4H) ppm; ^{13}C NMR (101 MHz, $CDCl_3$) δ = 143.5, 142.3, 139.4, 136.5, 134.0, 129.5, 128.4, 128.2, 127.8, 127.5, 126.7, 126.4, 120.7, 119.9, 45.2, 44.9, 29.0, 25.2, 22.8, 22.0, 21.5 ppm.

Supplementary Table 1. Rh[I]-catalyzed cycloaddition of 1,6-allenynes[a]

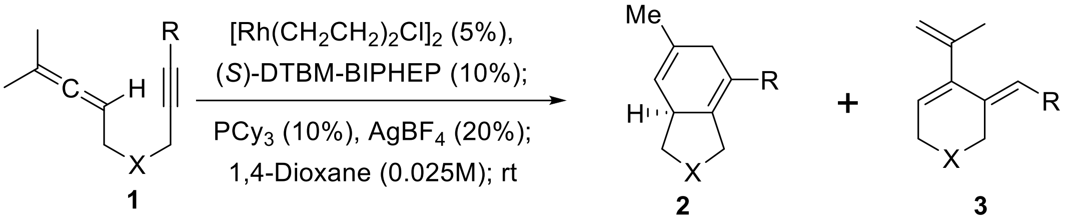

Entry	Substituents	Yield (%)	Ratio[c]	ee (%)
1	R = Me, X = NTs	76	**2l : 3l** < 5 : 95	-
2	R = TMS, X = NTs	94	**2m : 3m** < 5: 95	-
3	R = H, X= NTs	91	**2n : 3n** < 5 :95	-
4	R = COOMe, X = NTs	98	**2o : 3o** < 5 :95	-
5	R = Ph, X = O	95	**2p : 3p** < 5: 95	-
6	R = COOMe, X = O	97	**2q : 3q** < 5: 95	-

[a]All the reactions were conducted with **1** (0.1 mmol) in the presence of an *in situ* generated (*S*)-DTBM-BIPHEP (10 mol%)-PCy_3 (10 mol%)-$[Rh(CH_2CH_2)_2Cl]_2$ (5 mol%) complex in the presence of $AgBF_4$ (20 mol%) in degassed 1,4-dioxane at 25°C; [b]The combined yield of **2** and **3**, isolated yields were reported; [c]Determined by ^{1}H-NMR of the mixture;

Supplementary Table 2. The absolute electronic and free energies (in Hartrees) of the pathway **i** for the Rh(I)-catalyzed cycloisomerization of a 1,6-allenyne (**1a**) in gas (by the M06-L method) and solution (by the SMD M06-L//M06-L method) phases. PMe_3 ligand was used as the additional ligand.

	**E**	**E+ZPE**	**G**	**E_{soln}**
**A**	-6042.956113	-6040.798142	-6040.950265	-6043.007810
**PMe_3**	-461.048358	-460.934334	-460.963470	-461.048578
**$TS_{A\text{-}B(R)}$**	-6042.916951	-6040.760496	-6040.914399	-6042.968219
**B_R**	-6042.971120	-6040.810220	-6040.961916	-6043.022135
**C**	-6042.977350	-6040.812509	-6040.960157	-6043.029413
**D**	-6042.969247	-6040.805111	-6040.952403	-6043.021647
**$TS_{D\text{-}E}$**	-6042.931173	-6040.771497	-6040.918810	-6042.982903
**E**	-6042.955798	-6040.793693	-6040.940406	-6043.008494
**$TS_{E\text{-}F}$**	-6042.926092	-6040.766399	-6040.914103	-6042.979454
**F**	-6042.947082	-6040.781427	-6040.928272	-6043.003968
**G**	-6042.983925	-6040.819356	-6040.965471	-6043.032532
**$TS_{G\text{-}H}$**	-6042.936884	-6040.773415	-6040.919879	-6042.987267
**H**	-6042.991575	-6040.826883	-6040.973438	-6043.043349
**A_S**	-6042.948417	-6040.789171	-6040.942429	-6042.999223
**$TS_{A\text{-}B(S)}$**	-6042.913561	-6040.753828	-6040.904121	-6042.963671
**A′**	-5581.862421	-5579.821380	-5579.967703	-5581.917068
**$TS'_{A\text{-}B(R)}$**	-5581.820582	-5579.781699	-5579.926978	-5581.876106
**B'_R**	-5581.867907	-5579.825909	-5579.970111	-5581.924369
**D′**	-5581.889871	-5579.844895	-5579.992089	-5581.946750
**$TS'_{D\text{-}E}$**	-5581.857841	-5579.818811	-5579.964819	-5581.912539
**E′**	-5581.862503	-5579.821003	-5579.965838	-5581.920948
**$TS'_{E\text{-}F}$**	-5581.840976	-5579.802685	-5579.946903	-5581.895908
**F′**	-5581.867524	-5579.821914	-5579.966918	-5581.924305
**G′**	-5581.899719	-5579.854646	-5579.995825	-5581.955154
**$TS'_{G\text{-}H}$**	-5581.853583	-5579.812306	-5579.956461	-5581.906334
**H′**	-5581.923039	-5579.878034	-5580.018170	-5581.979263
**$TS'_{H\text{-}I}$**	-5581.892328	-5579.849116	-5579.990733	-5581.946244
**I′**	-5581.939492	-5579.892563	-5580.035404	-5581.994312
**1a**	-1455.253876	-1454.855481	-1454.914268	-1455.270955
**2a**	-1455.357854	-1454.951468	-1455.001769	-1455.375238

Supplementary Table 3. The relative electronic and free energies (in kcal/mol) of the pathway **i** for the Rh(I)-catalyzed cycloisomerization of a 1,6-allenyne (**1a**) in gas (by the M06-L method) and solution (by the SMD M06-L//M06-L method) phases. PMe_3 ligand was used as the additional ligand.

	△E	△E+ZPE	△G	△E_{soln}	△G_{soln}[a]
**A**	0.0	0.0	0.0	0.0	0.0
**$TS_{A\text{-}B(R)}$**	24.6	23.6	22.5	24.8	22.8
**B_R**	-9.4	-7.6	-7.3	-9.0	-6.9
**C**	-13.3	-9.0	-6.2	-13.6	-6.4
**D**	-8.2	-4.4	-1.3	-8.7	-1.8
**$TS_{D\text{-}E}$**	15.7	16.7	19.7	15.6	19.7
**E**	0.2	2.8	6.2	-0.4	5.6
**$TS_{E\text{-}F}$**	18.8	19.9	22.7	17.8	21.6
**F**	5.7	10.5	13.8	2.4	10.5
**G**	-17.5	-13.3	-9.5	-15.5	-7.6
**$TS_{G\text{-}H}$**	12.1	15.5	19.1	12.9	19.9
**H**	-22.3	-18.0	-14.5	-22.3	-14.6
**A_S**	4.8	5.6	4.9	5.4	5.5
**$TS_{A\text{-}B(S)}$**	26.7	27.8	29.0	27.7	30.0
**A′**	28.4	26.6	12.0	26.5	10.0
**$TS'_{A\text{-}B(R)}$**	54.7	51.5	37.5	52.2	35.0
**B'_R**	25.0	23.8	10.5	21.9	7.3
**D′**	11.2	11.9	-3.3	7.8	-6.7
**$TS'_{D\text{-}E}$**	31.3	28.2	13.8	29.3	11.8
**E′**	28.4	26.9	13.2	24.0	8.8
**$TS'_{E\text{-}F}$**	41.9	38.4	25.0	39.7	22.9
**F′**	25.2	26.3	12.5	21.9	9.1
**G′**	5.0	5.7	-5.7	2.6	-8.2
**$TS'_{G\text{-}H}$**	34.0	32.3	19.0	33.2	18.2
**H′**	-9.6	-8.9	-19.7	-12.6	-22.7
**$TS'_{H\text{-}I}$**	9.7	9.2	-2.5	8.2	-4.0
**I′**	-19.9	-18.0	-30.5	-22.0	-32.6

a. The free-energy corrections were taken from those obtained by the M06-L method in gas phase.

Supplementary Table 4. The absolute electronic energies (in Hartrees) of the pathway **i** for the Rh(I)-catalyzed cycloisomerization of a 1,6-allenyne (**1a**) in solution phase by different DFT methods. PMe_3 ligand was used as the additional ligand.

	$E_{B3LYP\text{-}D3}$	$E_{B3PW91\text{-}D3}$	$E_{BP86\text{-}D3}$	E_{M06}	$E_{PBE0\text{-}D3}$	$E_{\omega B97XD}$
**A**	-6043.998673	-6042.242667	-6044.104192	-6040.206378	-6037.950604	-6042.294861
**PMe_3**	-461.103768	-461.015242	-461.110726	-460.955627	-460.798574	-461.041035
**$TS_{A\text{-}B(R)}$**	-6043.957799	-6042.204917	-6044.072452	-6040.166690	-6037.911273	-6042.249730
**B_R**	-6044.002023	-6042.257790	-6044.117478	-6040.221961	-6037.968423	-6042.308480
**C**	-6044.002247	-6042.261824	-6044.119514	-6040.224960	-6037.973186	-6042.314457
**D**	-6043.992760	-6042.257071	-6044.115721	-6040.215772	-6037.968774	-6042.307937
**$TS_{D\text{-}E}$**	-6043.960147	-6042.225640	-6044.088515	-6040.172423	-6037.938918	-6042.271837
**E**	-6043.984509	-6042.248210	-6044.109198	-6040.195813	-6037.960715	-6042.296532
**$TS_{E\text{-}F}$**	-6043.953826	-6042.217731	-6044.079277	-6040.169998	-6037.932156	-6042.266876
**F**	-6043.977108	-6042.235141	-6044.093518	-6040.195358	-6037.951979	-6042.288051
**G**	-6044.012832	-6042.273455	-6044.130413	-6040.227322	-6037.984077	-6042.325921
**$TS_{G\text{-}H}$**	-6043.967842	-6042.227592	-6044.089051	-6040.180679	-6037.934963	-6042.276006
**H**	-6044.028636	-6042.285761	-6044.148007	-6040.229979	-6037.992518	-6042.328878
**A_S**	-6043.991955	-6042.236452	-6044.096651	-6040.197940	-6037.945070	-6042.286974
**$TS_{A\text{-}B(S)}$**	-6043.949612	-6042.197838	-6044.067124	-6040.158326	-6037.905116	-6042.239700
**A′**	-5582.841404	-5581.166334	-5582.931592	-5579.203988	-5577.097370	-5581.196061
**$TS'_{A\text{-}B(R)}$**	-5582.802321	-5581.129875	-5582.900582	-5579.162837	-5577.060137	-5581.152340
**B'_R**	-5582.843542	-5581.177416	-5582.939073	-5579.211196	-5577.112573	-5581.206142
**D′**	-5582.860226	-5581.201424	-5582.958384	-5579.234108	-5577.138034	-5581.234209
**$TS'_{D\text{-}E}$**	-5582.829784	-5581.170880	-5582.931601	-5579.202440	-5577.108481	-5581.202721
**E′**	-5582.841538	-5581.178474	-5582.938770	-5579.204408	-5577.115792	-5581.209341
**$TS'_{E\text{-}F}$**	-5582.813397	-5581.154949	-5582.916774	-5579.183809	-5577.091898	-5581.184217
**F′**	-5582.840504	-5581.177187	-5582.933301	-5579.215076	-5577.117180	-5581.211684
**G′**	-5582.873238	-5581.213626	-5582.970434	-5579.246194	-5577.149381	-5581.246663
**$TS'_{G\text{-}H}$**	-5582.829475	-5581.165684	-5582.933560	-5579.193995	-5577.097276	-5581.189715
**H′**	-5582.900697	-5581.244574	-5583.006886	-5579.259923	-5577.174855	-5581.270782
**$TS'_{H\text{-}I}$**	-5582.869849	-5581.213068	-5582.975907	-5579.235569	-5577.145820	-5581.241741
**I′**	-5582.914743	-5581.256310	-5583.011566	-5579.285938	-5577.192708	-5581.290178

Supplementary Table 5. The relative electronic energies (in kcal/mol) of the pathway **i** for the Rh(I)-catalyzed cycloisomerization of a 1,6-allenyne (**1a**) in solution phase by different DFT methods. PMe_3 ligand was used as the additional ligand.

	$\triangle E_{B3LYP-D3}$	$\triangle E_{B3PW91-D3}$	$\triangle E_{BP86-D3}$	$\triangle E_{M06}$	$\triangle E_{PBE0-D3}$	$\triangle E_{\omega B97XD}$
**A**	0.0	0.0	0.0	0.0	0.0	0.0
**TS$_{A-B(R)}$**	25.6	23.7	19.9	24.9	24.7	28.3
**B$_R$**	-2.1	-9.5	-8.3	-9.8	-11.2	-8.5
**C**	-2.2	-12.0	-9.6	-11.7	-14.2	-12.3
**D**	3.7	-9.0	-7.2	-5.9	-11.4	-8.2
**TS$_{D-E}$**	24.2	10.7	9.8	21.3	7.3	14.4
**E**	8.9	-3.5	-3.1	6.6	-6.3	-1.0
**TS$_{E-F}$**	28.1	15.6	15.6	22.8	11.6	17.6
**F**	13.5	4.7	6.7	6.9	-0.9	4.3
**G**	-8.9	-19.3	-16.5	-13.1	-21.0	-19.5
**TS$_{G-H}$**	19.3	9.5	9.5	16.1	9.8	11.8
**H**	-18.8	-27.0	-27.5	-14.8	-26.3	-21.3
**A$_S$**	4.2	3.9	4.7	5.3	3.5	4.9
**TS$_{A-B(S)}$**	30.8	28.1	23.3	30.2	28.5	34.6
**A′**	33.6	38.3	38.8	29.3	34.3	36.2
**TS′$_{A-B(R)}$**	58.1	61.2	58.3	55.2	57.7	63.7
**B′$_R$**	32.2	31.4	34.1	24.8	24.8	29.9
**D′**	21.8	16.3	22.0	10.4	8.8	12.3
**TS′$_{D-E}$**	40.9	35.5	38.8	30.3	27.3	32.1
**E′**	33.5	30.7	34.3	29.1	22.7	27.9
**TS′$_{E-F}$**	51.1	45.5	48.1	42.0	37.7	43.7
**F′**	34.1	31.5	37.8	22.4	21.9	26.4
**G′**	13.6	8.7	14.5	2.9	1.7	4.5
**TS′$_{G-H}$**	41.1	38.7	37.6	35.6	34.4	40.2
**H′**	-3.6	-10.8	-8.4	-5.8	-14.3	-10.6
**TS′$_{H-I}$**	15.7	9.0	11.0	9.5	3.9	7.6
**I′**	-12.4	-18.1	-11.4	-22.1	-25.5	-22.8

Supplementary Table 6. The relative Gibbs free energies (in kcal/mol) of the pathway **i** for the Rh(I)-catalyzed cycloisomerization of a 1,6-allenyne (**1a**) in solution phase by different DFT methods. PMe_3 ligand was used as the additional ligand.

	$\triangle G_{B3LYP-D3}$[a]	$\triangle G_{B3PW91-D3}$[a]	$\triangle G_{BP86-D3}$[a]	$\triangle G_{M06}$[a]	$\triangle G_{PBE0-D3}$[a]	$\triangle G_{\omega B97XD}$[a]
**A**	0.0	0.0	0.0	0.0	0.0	0.0
**TS$_{A-B(R)}$**	23.6	21.6	17.8	22.8	22.6	26.3
**B$_R$**	0.0	-7.4	-6.2	-7.7	-9.1	-6.4
**C**	4.9	-4.9	-2.5	-4.5	-7.1	-5.2
**D**	10.6	-2.1	-0.3	1.0	-4.5	-1.3
**TS$_{D-E}$**	28.3	14.8	13.9	25.4	11.4	18.5
**E**	14.9	2.5	2.8	12.6	-0.4	4.9
**TS$_{E-F}$**	32.0	19.5	19.5	26.7	15.4	21.4
**F**	21.7	12.9	14.8	15.0	7.3	12.4
**G**	-0.9	-11.3	-8.5	-5.1	-13.0	-11.5
**TS$_{G-H}$**	26.3	16.5	16.5	23.1	16.8	18.8
**H**	-11.1	-19.3	-19.8	-7.1	-18.6	-13.6
**A$_S$**	4.3	4.0	4.8	5.4	3.6	5.0
**TS$_{A-B(S)}$**	33.0	30.4	25.5	32.4	30.8	36.9
**A′**	17.1	21.9	22.4	12.9	17.8	19.8
**TS′$_{A-B(R)}$**	40.9	44.0	41.1	38.0	40.5	46.5
**B′$_R$**	17.7	16.8	19.6	10.3	10.2	15.4
**D′**	7.2	1.8	7.5	-4.1	-5.8	-2.2
**TS′$_{D-E}$**	23.3	18.0	21.3	12.8	9.8	14.5
**E′**	18.2	15.5	19.1	13.8	7.5	12.7
**TS′$_{E-F}$**	34.3	28.6	31.3	25.1	20.9	26.8
**F′**	21.4	18.8	25.0	9.6	9.1	13.7
**G′**	2.9	-2.1	3.7	-7.8	-9.0	-6.2
**TS′$_{G-H}$**	26.1	23.8	22.6	20.7	19.4	25.3
**H′**	-13.7	-20.9	-18.5	-15.9	-24.4	-20.7
**TS′$_{H-I}$**	3.6	-3.1	-1.1	-2.6	-8.3	-4.6
**I′**	-23.0	-28.7	-21.9	-32.7	-36.1	-33.4

a. The free-energy corrections were taken from those obtained by the M06-L method in gas phase.

Supplementary Table 7. The absolute (in Hartrees), relative electronic and free energies (in kcal/mol) of the pathway **i** for the Rh(I)-catalyzed cycloisomerization of a 1,6-allenyne (**1a**) in gas phase by the PW6B95-D3 method. PMe_3 ligand was used as the additional ligand.

	$E_{PW6B95\text{-}D3}$	$\triangle E_{PW6B95\text{-}D3}$	$\triangle G_{PW6B95\text{-}D3}{}^{a}$
**A**	-6050.769783	0.0	0.0
**PMe_3**	-461.521402	—	—
**$TS_{A\text{-}B(R)}$**	-6050.727997	26.2	24.2
**B_R**	-6050.779192	-5.9	-3.8
**C**	-6050.786654	-10.6	-3.5
**D**	-6050.782838	-8.2	-1.3
**$TS_{D\text{-}E}$**	-6050.748983	13.1	17.1
**E**	-6050.775757	-3.7	2.2
**$TS_{E\text{-}F}$**	-6050.743963	16.2	20.1
**F**	-6050.756089	8.6	16.7
**G**	-6050.803695	-21.3	-13.4
**$TS_{G\text{-}H}$**	-6050.757533	7.7	14.7
**H**	-6050.808931	-24.6	-16.9
**A_S**	-6050.766341	2.2	2.2
**$TS_{A\text{-}B(S)}$**	-6050.723731	28.9	31.2
**A′**	-5589.188229	37.7	21.3
**$TS'_{A\text{-}B(R)}$**	-5589.147955	63.0	45.9
**B'_R**	-5589.193389	34.5	20.0
**D′**	-5589.219122	18.4	3.8
**$TS'_{D\text{-}E}$**	-5589.190733	36.2	18.6
**E′**	-5589.195494	33.2	17.9
**$TS'_{E\text{-}F}$**	-5589.175096	46.0	29.1
**F′**	-5589.198517	31.3	18.5
**G′**	-5589.233329	9.4	-1.3
**$TS'_{G\text{-}H}$**	-5589.183982	40.4	25.5
**H′**	-5589.262795	-9.0	-19.1
**$TS'_{H\text{-}I}$**	-5589.232240	10.1	-2.0
**I′**	-5589.279565	-19.6	-30.2

a. The free-energy corrections were taken from those obtained by the M06-L method in gas phase.

Supplementary Table 8. The absolute electronic and free energies (in Hartrees) of the pathway **ii** for the Rh(I)-catalyzed cycloisomerization of a 1,6-allenyne (**1a**) in gas (by the M06-L method) and solution (by the SMD M06-L//M06-L method) phases.

	E	E+ZPE	G	E_{soln}
**J**	-5581.869676	-5579.826307	-5579.967603	-5581.922663
**TS_{J-K}**	-5581.853026	-5579.808952	-5579.947774	-5581.906113
**K**	-5581.883871	-5579.840164	-5579.981584	-5581.940591
**TS_{K-L}**	-5581.801629	-5579.766086	-5579.911063	-5581.858370
**L**	-5581.843047	-5579.804623	-5579.948952	-5581.899198
**TS_{L-M}**	-5581.828356	-5579.793575	-5579.937685	-5581.884370
**M**	-5581.866472	-5579.826146	-5579.971519	-5581.919327

Supplementary Table 9. The relative electronic and free energies (in kcal/mol) of the pathway **ii** for the Rh(I)-catalyzed cycloisomerization of a 1,6-allenyne (**1a**) in gas (by the M06-L method) and solution (by the SMD M06-L//M06-L method) phases.

	△E	△E+ZPE	△G	△E_{soln}	△G_{soln}[a]
**J**	23.9	23.5	11.9	23.0	11.0
**TS_{J-K}**	34.4	34.4	24.4	33.3	23.3
**K**	15.0	14.8	3.1	11.7	-0.2
**TS_{K-L}**	66.6	61.3	47.4	63.3	44.1
**L**	40.6	37.1	23.6	37.7	20.7
**TS_{L-M}**	49.8	44.1	30.7	47.0	27.8
**M**	25.9	23.6	9.5	25.1	8.6

a. The free-energy corrections were taken from those obtained by the M06-L method in gas phase.

Supplementary Table 10. The absolute electronic energies (in Hartrees) of the pathway **ii** for the Rh(I)-catalyzed cycloisomerization of a 1,6-allenyne (**1a**) in solution phase by different DFT methods.

	$E_{B3LYP-D3}$	$E_{B3PW91-D3}$	$E_{BP86-D3}$	E_{M06}	$E_{PBE0-D3}$	$E_{\omega B97XD}$
**J**	-5582.845742	-5581.185955	-5582.958939	-5579.201822	-5577.109938	-5581.206634
**TS_{J-K}**	-5582.829677	-5581.170801	-5582.941286	-5579.187498	-5577.097370	-5581.193403
**K**	-5582.865843	-5581.203962	-5582.971433	-5579.216763	-5577.132692	-5581.227883
**TS_{K-L}**	-5582.787134	-5581.125090	-5582.899895	-5579.140079	-5577.054081	-5581.143700
**L**	-5582.832495	-5581.166233	-5582.935016	-5579.182348	-5577.096406	-5581.192686
**TS_{L-M}**	-5582.815515	-5581.149885	-5582.916748	-5579.171537	-5577.081601	-5581.176760
**M**	-5582.841926	-5581.180778	-5582.948079	-5579.201698	-5577.110369	-5581.205788

Supplementary Table 11. The relative electronic and free energies (in kcal/mol) of the pathway **ii** for the Rh(I)-catalyzed cycloisomerization of a 1,6-allenyne (**1a**) in solution phase by different DFT methods.

	$\triangle E_{B3LYP-D3}$	$\triangle E_{B3PW91-D3}$	$\triangle E_{BP86-D3}$	$\triangle E_{M06}$	$\triangle E_{PBE0-D3}$	$\triangle E_{\omega B97XD}$
**J**	30.9	26.0	21.7	30.7	26.4	29.6
**TS_{J-K}**	40.9	35.5	32.7	39.7	34.3	37.9
**K**	18.2	14.7	13.8	21.3	12.1	16.3
**TS_{K-L}**	67.6	64.2	58.7	69.4	61.5	69.1
**L**	39.2	38.4	36.7	42.9	34.9	38.4
**TS_{L-M}**	49.8	48.7	48.1	49.7	44.2	48.4
**M**	33.2	29.3	28.5	30.8	26.1	30.1
	$\triangle G_{B3LYP-D3}{}^{a}$	$\triangle G_{B3PW91-D3}{}^{a}$	$\triangle G_{BP86-D3}{}^{a}$	$\triangle G_{M06}{}^{a}$	$\triangle G_{PBE0-D3}{}^{a}$	$\triangle G_{\omega B97XD}{}^{a}$
**J**	18.8	14.0	9.7	18.7	14.4	17.6
**TS_{J-K}**	30.9	25.5	22.7	29.7	24.3	27.9
**K**	6.4	2.9	2.0	9.5	0.3	4.4
**TS_{K-L}**	48.4	45.0	39.5	50.2	42.2	49.9
**L**	22.2	21.4	19.7	25.9	17.9	21.4
**TS_{L-M}**	30.7	29.5	29.0	30.6	25.0	29.2
**M**	16.8	12.8	12.0	14.3	9.7	13.7

a. The free-energy corrections were taken from those obtained by the M06-L method in gas phase.

Supplementary Table 12. The absolute electronic (in Hartrees), relative electronic and free energies (in kcal/mol) of the pathway **ii** for the Rh(I)-catalyzed cycloisomerization of a 1,6-allenyne (**1a**) in gas phase by PW6B95-D3 method.

	$E_{PW6B95-D3}$	$\triangle E_{PW6B95-D3}$	$\triangle G_{PW6B95-D3}$[a]
**TS_{J-K}**	-5589.189191	37.1	27.1
**TS_{K-L}**	-5589.137829	69.4	50.1
**TS_{L-M}**	-5589.166292	51.5	32.4

a. The free-energy corrections were taken from those obtained by the M06-L method in gas phase.

Supplementary Table 13. The absolute electronic and free energies (in Hartrees) for the pathways **iii** and **iv** of Rh(I)-catalyzed cycloisomerization of 1,6-allenynes (**1a**) in gas (by the M06-L method) and solution (by the SMD M06-L//M06-L method) phases.

	**E**	**E+ZPE**	**G**	**E_{soln}**
**N1**	-6042.917982	-6040.755863	-6040.903979	-6042.968247
**TS_{A-N}**	-6042.918983	-6040.760025	-6040.909763	-6042.969961
**N**	-6042.962624	-6040.800864	-6040.949837	-6043.014229
**O**	-6043.002696	-6040.839744	-6040.991791	-6043.055605
**P**	-5581.850269	-5579.805922	-5579.945333	-5581.906054
**TS_{P-Q}**	-5581.845599	-5579.802642	-5579.942916	-5581.900835
**Q**	-5581.888438	-5579.844171	-5579.987582	-5581.943901
**TS_{Q-R}**	-5581.869216	-5579.827235	-5579.966318	-5581.925664
**R**	-5581.889977	-5579.849171	-5579.991719	-5581.945946
**TS_{R-S}**	-5581.887094	-5579.846391	-5579.987792	-5581.94214
**S**	-5581.932508	-5579.887903	-5580.031586	-5581.984052
**T**	-5581.924925	-5579.879399	-5580.021163	-5581.978615

Supplementary Table 14. The relative electronic and free energies (in kcal/mol) of the pathways **iii** and **iv** for the Rh(I)-catalyzed cycloisomerization of a 1,6-allenyne (**1a**) in gas (by the M06-L method) and solution (by the SMD M06-L//M06-L method) phases.

	△E	△E+ZPE	△G	$△E_{soln}$	$△G_{soln}$[a]
**N1**	23.9	26.5	29.0	24.8	29.9
**TS_{A-N}**	23.3	23.9	25.4	23.8	25.9
**N**	-4.1	-1.7	0.3	-4.0	0.3
**O**	-29.2	-26.1	-26.1	-30.0	-26.8
**P**	36.1	36.3	26.0	33.4	23.3
**TS_{P-Q}**	39.0	38.4	27.5	36.6	25.2
**Q**	12.1	12.3	-0.5	9.6	-3.0
**TS_{Q-R}**	24.2	22.9	12.8	21.1	9.7
**R**	11.2	9.2	-3.1	8.3	-5.9
**TS_{R-S}**	13.0	10.9	-0.6	10.7	-2.9
**S**	-15.5	-15.1	-28.1	-15.6	-28.1
**T**	-10.8	-9.8	-21.6	-12.2	-23.0

a. The free-energy corrections were taken from those obtained by the M06-L method in gas phase.

Supplementary Table 15. The absolute electronic energies (in Hartrees) of the pathways **iii** and **iv** for the Rh(I)-catalyzed cycloisomerization of a 1,6-allenyne (**1a**) in solution phase by different DFT methods.

	$E_{B3LYP-D3}$	$E_{B3PW91-D3}$	$E_{BP86-D3}$	E_{M06}	$E_{PBE0-D3}$	$E_{\omega B97XD}$
**N1**	-6043.946199	-6042.200974	-6044.065345	-6040.166145	-6037.913055	-6042.249432
**TS_{A-N}**	-6043.957961	-6042.205389	-6044.073775	-6040.168052	-6037.913468	-6042.249327
**N**	-6043.997564	-6042.252135	-6044.111134	-6040.216026	-6037.964207	-6042.304388
**O**	-6044.040035	-6042.285272	-6044.140279	-6040.246893	-6037.996036	-6042.336815
**P**	-5582.826108	-5581.15975	-5582.929936	-5579.187324	-5577.085614	-5581.18615
**TS_{P-Q}**	-5582.816608	-5581.155378	-5582.92469	-5579.177482	-5577.084863	-5581.177356
**Q**	-5582.861082	-5581.197247	-5582.965497	-5579.221708	-5577.129025	-5581.219518
**TS_{Q-R}**	-5582.849174	-5581.191515	-5582.958697	-5579.212456	-5577.124432	-5581.214351
**R**	-5582.869521	-5581.208238	-5582.975188	-5579.223708	-5577.137248	-5581.231542
**TS_{R-S}**	-5582.866359	-5581.20395	-5582.972131	-5579.223925	-5577.132973	-5581.228544
**S**	-5582.90312	-5581.240945	-5583.001453	-5579.272527	-5577.172152	-5581.272726
**T**	-5582.89725	-5581.232936	-5582.995454	-5579.26237	-5577.163417	-5581.260854

Supplementary Table 16. The relative electronic and free energies (in kcal/mol) of the pathways **iii** and **iv** for the Rh(I)-catalyzed cycloisomerization of a 1,6-allenyne (**1a**) in solution phase by different DFT methods.

	$\triangle E_{B3LYP-D3}$	$\triangle E_{B3PW91-D3}$	$\triangle E_{BP86-D3}$	$\triangle E_{M06}$	$\triangle E_{PBE0-D3}$	$\triangle E_{\omega B97XD}$
**N1**	32.9	26.2	24.4	25.3	23.6	28.5
**TS$_{A-N}$**	25.5	23.4	19.1	24.0	23.3	28.6
**N**	0.7	-5.9	-4.4	-6.1	-8.5	-6.0
**O**	-26.0	-26.7	-22.6	-25.4	-28.5	-26.3
**P**	43.2	42.5	39.9	39.8	41.7	42.5
**TS$_{P-Q}$**	49.1	45.2	43.2	46.0	42.1	48.0
**Q**	21.2	18.9	17.6	18.2	14.4	21.5
**TS$_{Q-R}$**	28.7	22.5	21.8	24.0	17.3	24.8
**R**	15.9	12.0	11.5	17.0	9.3	14.0
**TS$_{R-S}$**	17.9	14.7	13.4	16.8	12.0	15.9
**S**	-5.2	-8.5	-5.0	-13.7	-12.6	-11.9
**T**	-1.5	-3.5	-1.2	-7.3	-7.1	-4.4
	$\triangle G_{B3LYP-D3}{}^{a}$	$\triangle G_{B3PW91-D3}{}^{a}$	$\triangle G_{BP86-D3}{}^{a}$	$\triangle G_{M06}{}^{a}$	$\triangle G_{PBE0-D3}{}^{a}$	$\triangle G_{\omega B97XD}{}^{a}$
**N1**	38.0	31.3	29.5	30.4	28.7	33.6
**TS$_{A-N}$**	27.6	25.5	21.2	26.1	25.4	30.7
**N**	5.1	-1.5	0.0	-1.7	-4.1	-1.6
**O**	-22.8	-23.6	-19.5	-22.3	-25.3	-23.2
**P**	33.1	32.4	29.8	29.7	31.6	32.4
**TS$_{P-Q}$**	37.7	33.7	31.7	34.5	30.7	36.5
**Q**	8.6	6.3	4.9	5.6	1.8	8.9
**TS$_{Q-R}$**	17.4	11.2	10.5	12.7	6.0	13.4
**R**	1.7	-2.2	-2.8	2.7	-5.0	-0.3
**TS$_{R-S}$**	4.3	1.1	-0.2	3.2	-1.6	2.3
**S**	-17.7	-21.1	-17.6	-26.2	-25.2	-24.4
**T**	-12.3	-14.3	-12.0	-18.1	-17.9	-15.2

a. The free-energy corrections were taken from those obtained by the M06-L method in gas phase.

Supplementary Table 17. The absolute electronic (in Hartrees), relative electronic and free energies (in kcal/mol) of the pathways **iii** and **iv** for the Rh(I)-catalyzed cycloisomerization of a 1,6-allenyne (**1a**) in gas phase by PW6B95-D3 method.

	$E_{PW6B95-D3}$	$\triangle E_{PW6B95-D3}$	$\triangle G_{PW6B95-D3}{}^{a}$
**TS$_{A-N}$**	-6050.730039	24.9	27.0
**TS$_{P-Q}$**	-5589.176107	45.4	33.9
**TS$_{Q-R}$**	-5589.206272	26.4	15.1
**TS$_{R-S}$**	-5589.224349	15.1	1.5

a. The free-energy corrections were taken from those obtained by the M06-L method in gas phase.

Supplementary Table 18. The absolute electronic and free energies (in Hartrees) for the pathways **v** and **vi** of Rh(I)-catalyzed cycloisomerization of 1,6-allenynes (**1a**) in gas (by the M06-L method) and solution (by the SMD M06-L//M06-L method) phases.

	**E**	**E+ZPE**	**G**	**E_{soln}**
**U**	-6042.955818	-6040.796577	-6040.946741	-6043.009378
**TS_{U-V}**	-6042.905726	-6040.749797	-6040.898529	-6042.959248
**V**	-6042.922477	-6040.764802	-6040.912887	-6042.976760
**TSV_{-W}**	-6042.895950	-6040.740501	-6040.889929	-6042.950230
**W**	-6042.959466	-6040.801200	-6040.953670	-6043.009471
**X**	-5581.852969	-5579.810947	-5579.955926	-5581.909363
**TS_{X-Y}**	-5581.819865	-5579.782820	-5579.929179	-5581.873561
**Y**	-5581.829197	-5579.789918	-5579.935525	-5581.884623
**TS_{Y-Z}**	-5581.820653	-5579.785324	-5579.934217	-5581.874990
**Z**	-5581.876219	-5579.834254	-5579.982311	-5581.928195

Supplementary Table 19. The relative electronic and free energies (in kcal/mol) of the pathways **v** and **vi** for the Rh(I)-catalyzed cycloisomerization of a 1,6-allenyne (**1a**) in gas (by the M06-L method) and solution (by the SMD M06-L//M06-L method) phases.

	**△E**	**△E+ZPE**	**△G**	**$△E_{soln}$**	**$△G_{soln}^{a}$**
**U**	0.2	1.0	2.2	-1.0	1.0
**TS_{U-V}**	31.6	30.3	32.5	30.5	31.3
**V**	21.1	20.9	23.5	19.5	21.8
**TSV_{-W}**	37.8	36.2	37.9	36.1	36.2
**W**	-2.1	-1.9	-2.1	-1.0	-1.1
**X**	34.4	33.2	19.4	31.3	16.3
**TS_{X-Y}**	55.2	50.8	36.2	53.8	34.8
**Y**	49.3	46.4	32.2	46.8	29.7
**TS_{Y-Z}**	54.7	49.2	33.0	52.9	31.2
**Z**	19.8	18.5	2.8	19.5	2.5

a. The free-energy corrections were taken from those obtained by the M06-L method in gas phase.

Supplementary Table 20. The absolute electronic energies (in Hartrees) of the pathways **v** and **vi** for the Rh(I)-catalyzed cycloisomerization of a 1,6-allenyne (**1a**) in solution phase by different DFT methods.

	$E_{B3LYP-D3}$	$E_{B3PW91-D3}$	$E_{BP86-D3}$	E_{M06}	$E_{PBE0-D3}$	$E_{ωB97XD}$
**U**	-6043.991466	-6042.237777	-6044.099223	-6040.202651	-6037.948054	-6042.288847
**TS$_{U-V}$**	-6043.943103	-6042.195059	-6044.061872	-6040.146976	-6037.903603	-6042.241550
**V**	-6043.960252	-6042.208502	-6044.073358	-6040.162741	-6037.916444	-6042.256306
**TSV$_{-W}$**	-6043.933376	-6042.179902	-6044.046413	-6040.140935	-6037.888266	-6042.226770
**W**	-6043.997547	-6042.238480	-6044.098176	-6040.205250	-6037.947522	-6042.291533
**X**	-5582.825914	-5581.152963	-5582.915883	-5579.192971	-5577.084165	-5581.185398
**TS$_{X-Y}$**	-5582.794738	-5581.124647	-5582.891995	-5579.161590	-5577.055580	-5581.153854
**Y**	-5582.810033	-5581.137740	-5582.903684	-5579.165997	-5577.069178	-5581.166723
**TS$_{Y-Z}$**	-5582.804264	-5581.128379	-5582.893139	-5579.162249	-5577.064476	-5581.159247
**Z**	-5582.853408	-5581.181381	-5582.940046	-5579.220328	-5577.116560	-5581.215102

Supplementary Table 21. The relative electronic and free energies (in kcal/mol) of the pathways **v** and **vi** for the Rh(I)-catalyzed cycloisomerization of a 1,6-allenyne (**1a**) in solution phase by different DFT methods.

	$\triangle E_{B3LYP-D3}$	$\triangle E_{B3PW91-D3}$	$\triangle E_{BP86-D3}$	$\triangle E_{M06}$	$\triangle E_{PBE0-D3}$	$\triangle E_{ωB97XD}$
**U**	4.5	3.1	3.1	2.3	1.6	3.8
**TS$_{U-V}$**	34.9	29.9	26.6	37.3	29.5	33.5
**V**	24.1	21.4	19.3	27.4	21.4	24.2
**TSV$_{-W}$**	41.0	39.4	36.3	41.1	39.1	42.7
**W**	0.7	2.6	3.8	0.7	1.9	2.1
**X**	43.3	46.7	48.7	36.3	42.6	42.9
**TS$_{X-Y}$**	62.9	64.5	63.7	55.9	60.5	62.7
**Y**	53.3	56.3	56.3	53.2	52.0	54.7
**TS$_{Y-Z}$**	56.9	62.2	63.0	55.5	54.9	59.3
**Z**	26.0	28.9	33.5	19.1	22.3	24.3
	$\triangle G_{B3LYP-D3}$[a]	$\triangle G_{B3PW91-D3}$[a]	$\triangle G_{BP86-D3}$[a]	$\triangle G_{M06}$[a]	$\triangle G_{PBE0-D3}$[a]	$\triangle G_{ωB97XD}$[a]
**U**	6.5	5.1	5.1	4.4	3.6	5.8
**TS$_{U-V}$**	35.7	30.7	27.4	38.1	30.3	34.3
**V**	26.5	23.8	21.7	29.7	23.8	26.5
**TSV$_{-W}$**	41.1	39.5	36.4	41.2	39.2	42.8
**W**	0.7	2.6	3.7	0.7	1.9	2.1
**X**	28.3	31.7	33.7	21.2	27.6	27.9
**TS$_{X-Y}$**	43.9	45.5	44.7	37.0	41.5	43.7
**Y**	36.1	39.2	39.2	36.1	34.9	37.5
**TS$_{Y-Z}$**	35.2	40.5	41.3	33.9	33.3	37.7
**Z**	9.1	11.9	16.5	2.1	5.3	7.3

a. The free-energy corrections were taken from those obtained by the M06-L method in gas phase.

Supplementary Table 22. The absolute electronic (in Hartrees), relative electronic and free energies (in kcal/mol) of the pathways **v** and **vi** for the Rh(I)-catalyzed cycloisomerization of a 1,6-allenyne (**1a**) in gas phase by PW6B95-D3 method.

	$E_{PW6B95-D3}$	$\triangle E_{PW6B95-D3}$	$\triangle G_{PW6B95-D3}$[a]
TS_{U-V}	-6050.719645	31.5	32.3
TS_{V-W}	-6050.704532	40.9	41.1
TS_{X-Y}	-5589.150020	61.7	42.7
TS_{Y-Z}	-5589.152801	60.0	38.3

a. The free-energy corrections were taken from those obtained by the M06-L method in gas phase.

Supplementary Table 23. The absolute electronic and free energies (in Hartrees) for the pathway **vii** of Rh(I)-catalyzed cycloisomerization of 1,6-allenynes (**1a**) in gas (by the M06-L method) and solution (by the SMD M06-L//M06-L method) phases.

	E	E+ZPE	G	E_{soln}
TS_{U-AA}	-6042.897357	-6040.738588	-6040.886691	-6042.947963
**AA**	-6042.898070	-6040.739050	-6040.888223	-6042.948373

Supplementary Table 24. The relative electronic and free energies (in kcal/mol) of the pathway **vii** for the Rh(I)-catalyzed cycloisomerization of a 1,6-allenyne (**1a**) in gas (by the M06-L method) and solution (by the SMD M06-L//M06-L method) phases.

	$\triangle E$	$\triangle E+ZPE$	$\triangle G$	$\triangle E_{soln}$	$\triangle G_{soln}$[a]
TS_{U-AA}	36.9	37.4	39.9	37.6	40.6
**AA**	36.4	37.1	38.9	37.3	39.8

a. The free-energy corrections were taken from those obtained by the M06-L method in gas phase.

Supplementary Table 25. The absolute electronic energies (in Hartrees) of the pathway **vii** for the Rh(I)-catalyzed cycloisomerization of a 1,6-allenyne (**1a**) in solution phase by different DFT methods.

	$E_{B3LYP-D3}$	$E_{B3PW91-D3}$	$E_{BP86-D3}$	E_{M06}	$E_{PBE0-D3}$	$E_{\omega B97XD}$
TS_{U-AA}	-6043.922192	-6042.174510	-6044.051172	-6040.133771	-6037.879184	-6042.206250
**AA**	-6043.922877	-6042.174231	-6044.051987	-6040.133530	-6037.879304	-6042.205542

Supplementary Table 26. The relative electronic and free energies (in kcal/mol) of the pathway **vii** for the Rh(I)-catalyzed cycloisomerization of a 1,6-allenyne (**1a**) in solution phase by different DFT methods.

	△E$_{B3LYP-D3}$	△E$_{B3PW91-D3}$	△E$_{BP86-D3}$	△E$_{M06}$	△E$_{PBE0-D3}$	△E$_{ωB97XD}$
**TS$_{U-AA}$**	48.0	42.8	33.3	45.6	44.8	55.6
**AA**	47.6	42.9	32.8	45.7	44.7	56.0
	**△G$_{B3LYP-D3}$[a]**	**△G$_{B3PW91-D3}$[a]**	**△G$_{BP86-D3}$[a]**	**△G$_{M06}$[a]**	**△G$_{PBE0-D3}$[a]**	**△G$_{ωB97XD}$[a]**
**TS$_{U-AA}$**	51.0	45.8	36.3	48.6	47.8	58.6
**AA**	50.1	45.4	35.3	48.2	47.2	58.5

a. The free-energy corrections were taken from those obtained by the M06-L method in gas phase.

Supplementary Table 27. The absolute electronic (in Hartrees), relative electronic and free energies (in kcal/mol) of the pathway **vii** for the Rh(I)-catalyzed cycloisomerization of a 1,6-allenyne (**1a**) in gas phase by PW6B95-D3 method.

	E$_{PW6B95-D3}$	△E$_{PW6B95-D3}$	△G$_{PW6B95-D3}$[a]
**TS$_{U-AA}$**	-6050.698341	44.8	47.9
**AA**	-6050.696657	45.9	48.4

a. The free-energy corrections were taken from those obtained by the M06-L method in gas phase.

Supplementary Table 28. The absolute electronic and free energies (in Hartrees) of the pathway **i-Ph** for the Rh(I)-catalyzed cycloisomerization of a 1,6-allenyne (**1a**) in gas (by the M06-L method) and solution (by the SMD M06-L//M06-L method) phases. PPh_3 was used as the additional ligand.

	E	E+ZPE	G	E$_{soln}$
**APh**	-6618.093207	-6615.772240	-6615.932374	-6618.147344
**PPh$_3$**	-1036.179190	-1035.905167	-1035.951486	-1036.188877
**TSPh$_{A-B(R)}$**	-6618.055964	-6615.736276	-6615.896098	-6618.110745
**BPh$_{(R)}$**	-6618.104156	-6615.779416	-6615.935719	-6618.159594
**CPh**	-6618.107374	-6615.783176	-6615.940985	-6618.162804
**DPh**	-6618.088334	-6615.761826	-6615.917202	-6618.145213
**TSPh$_{D-E}$**	-6618.044965	-6615.724211	-6615.880753	-6618.104131
**TSPh$_{E-F}$**	-6618.054891	-6615.735201	-6615.891919	-6618.111844
**GPh**	-6618.088839	-6615.764769	-6615.921502	-6618.140299
**TSPh$_{G-H}$**	-6618.050096	-6615.728044	-6615.883386	-6618.106200

Supplementary Table 29. The relative electronic and free energies (in kcal/mol) of the pathway **i-Ph** and the lowest-energy side-reaction oxidative coupling pathway (via **TS$_{P-Q}$**) for the Rh(I)-catalyzed cycloisomerization of a 1,6-allenyne (**1a**) in gas (by the M06-L method) and solution (by the SMD M06-L//M06-L method) phases. PPh_3 was used as the additional ligand.

	△E	△E+ZPE	△G	△E$_{soln}$	△G$_{soln}$[a]
**APh**	0.0	0.0	0.0	0.0	0.0
**TSPh$_{A-B(R)}$**	23.4	22.6	22.8	23.0	22.4
**BPh$_{(R)}$**	-6.9	-4.5	-2.1	-7.7	-2.9
**CPh**	-8.9	-6.9	-5.4	-9.7	-6.2
**DPh**	3.1	6.5	9.5	1.3	7.8
**TSPh$_{D-E}$**	30.3	30.1	32.4	27.1	29.2
**TSPh$_{E-F}$**	24.0	23.2	25.4	22.3	23.6
**GPh**	2.7	4.7	6.8	4.4	8.5
**TSPh$_{G-H}$**	27.1	27.7	30.7	25.8	29.5
**A′**	32.4	28.7	8.3	26.0	1.9
**TS′$_{A-B(R)}$**	58.6	53.6	33.8	51.7	26.9
**B′$_{R}$**	28.9	25.8	6.8	21.4	-0.8
**D′**	15.2	13.9	-7.0	7.4	-14.8
**TS′$_{D-E}$**	35.3	30.3	10.1	28.8	3.7
**E′**	32.3	28.9	9.4	23.5	0.7
**TS′$_{E-F}$**	45.8	40.4	21.3	39.3	14.7
**F′**	29.2	28.3	8.8	21.4	1.0
**G′**	9.0	7.8	-9.4	2.1	-16.3
**TS′$_{G-H}$**	37.9	34.4	15.3	32.7	10.1
**H′**	-5.7	-6.9	-23.4	-13.0	-30.8
**TS′$_{H-I}$**	13.6	11.3	-6.2	7.7	-12.1
**I′**	-16.0	-16.0	-34.2	-22.5	-40.7
**TS$_{P-Q}$**	42.9	40.4	23.8	36.2	17.1

a. The free-energy corrections were taken from those obtained by the M06-L method in gas phase.

Supplementary Table 30. The absolute electronic energies (in Hartrees) of the pathway **i-Ph** for the Rh(I)-catalyzed cycloisomerization of a 1,6-allenyne (**1a**) in solution phase by different DFT methods. PPh_3 was used as the additional ligand.

	$E_{B3LYP\text{-}D3}$	$E_{B3PW91\text{-}D3}$	$E_{PBE0\text{-}D3}$	$E_{\omega B97XD}$
**APh**	-6619.243083	-6617.269049	-6612.512052	-6617.313318
**PPh_3**	-1036.338441	-1036.030467	-1035.357573	-1036.055440
**$TSPh_{A\text{-}B(R)}$**	-6619.201325	-6617.232312	-6612.472898	-6617.271296
**$BPh_{(R)}$**	-6619.240620	-6617.279627	-6612.524656	-6617.325199
**CPh**	-6619.240534	-6617.284525	-6612.529314	-6617.331038
**DPh**	-6619.216570	-6617.262644	-6612.509816	-6617.308432
**$TSPh_{D\text{-}E}$**	-6619.180905	-6617.224184	-6612.473937	-6617.265718
**$TSPh_{E\text{-}F}$**	-6619.189139	-6617.235034	-6612.484225	-6617.279312
**GPh**	-6619.220905	-6617.266832	-6612.511332	-6617.312164
**$TSPh_{G\text{-}H}$**	-6619.184311	-6617.223885	-6612.468083	-6617.269814

Supplementary Table 31. The relative electronic energies (in kcal/mol) of the pathway **i-Ph** and the lowest-energy side-reaction oxidative coupling pathway (via **$TS_{P\text{-}Q}$**) for the Rh(I)-catalyzed cycloisomerization of a 1,6-allenyne (**1a**) in solution phase by different DFT methods. PPh_3 was used as the additional ligand.

	$\Delta E_{B3LYP\text{-}D3}$	$\Delta E_{B3PW91\text{-}D3}$	$\Delta E_{PBE0\text{-}D3}$	$\Delta E_{\omega B97XD}$
**APh**	0.0	0.0	0.0	0.0
**$TSPh_{A\text{-}B(R)}$**	26.2	23.1	24.6	26.4
**$BPh_{(R)}$**	1.5	-6.6	-7.9	-7.5
**CPh**	1.6	-9.7	-10.8	-11.1
**DPh**	16.6	4.0	1.4	3.1
**$TSPh_{D\text{-}E}$**	39.0	28.2	23.9	29.9
**$TSPh_{E\text{-}F}$**	33.9	21.3	17.5	21.3
**GPh**	13.9	1.4	0.5	0.7
**$TSPh_{G\text{-}H}$**	36.9	28.3	27.6	27.3
**A′**	39.7	45.3	35.8	38.8
**$TS'_{A\text{-}B(R)}$**	64.2	68.2	59.2	66.2
**B'_R**	38.3	38.4	26.3	32.5
**D′**	27.9	23.3	10.3	14.9
**$TS'_{D\text{-}E}$**	47.0	42.5	28.9	34.6
**E′**	39.6	37.7	24.3	30.5
**$TS'_{E\text{-}F}$**	57.3	52.5	39.3	46.2
**F′**	40.2	38.5	23.4	29.0
**G′**	19.7	15.7	3.2	7.0
**$TS'_{G\text{-}H}$**	47.2	45.7	35.9	42.8
**H′**	2.5	-3.8	-12.8	-8.1
**$TS'_{H\text{-}I}$**	21.8	16.0	5.4	10.1
**I′**	-6.3	-11.1	-24.0	-20.3
**$TS_{P\text{-}Q}$**	55.2	52.2	43.7	50.5

a. The free-energy corrections were taken from those obtained by the M06-L method in gas phase.

Supplementary Table 32. The relative free energies (in kcal/mol) of the pathway **i-Ph** and the lowest-energy side-reaction oxidative coupling pathway (via **TS$_{P-Q}$**) for the Rh(I)-catalyzed cycloisomerization of a 1,6-allenyne (**1a**) in solution phase by different DFT methods. PPh_3 was used as the additional ligand.

	$\Delta G_{B3LYP-D3}$[a]	$\Delta G_{B3PW91-D3}$[a]	$\Delta G_{PBE0-D3}$[a]	$\Delta G_{\omega B97XD}$[a]
**APh**	—	—	—	—
**PPh_3**	—	—	—	—
**TSPh$_{A-B(R)}$**	25.6	22.4	24.0	25.8
**BPh$_{(R)}$**	6.3	-1.9	-3.1	-2.7
**CPh**	5.1	-6.2	-7.3	-7.6
**DPh**	23.1	10.5	7.9	9.5
**TSPh$_{D-E}$**	41.1	30.3	26.0	32.0
**TSPh$_{E-F}$**	35.2	22.7	18.8	22.7
**GPh**	18.0	5.5	4.5	4.8
**TSPh$_{G-H}$**	40.5	31.9	31.2	30.9
**A′**	15.6	21.2	11.7	14.7
**TS′$_{A-B(R)}$**	39.4	43.4	34.4	41.4
**B′$_R$**	16.2	16.2	4.1	10.3
**D′**	5.7	1.1	-11.9	-7.3
**TS′$_{D-E}$**	21.8	17.3	3.7	9.4
**E′**	16.7	14.8	1.4	7.6
**TS′$_{E-F}$**	32.7	28.0	14.8	21.7
**F′**	19.8	18.1	3.0	8.6
**G′**	1.4	-2.7	-15.1	-11.3
**TS′$_{G-H}$**	24.6	23.1	13.3	20.2
**H′**	-15.3	-21.5	-30.5	-25.8
**TS′$_{H-I}$**	2.0	-3.8	-14.4	-9.7
**I′**	-24.6	-29.3	-42.2	-38.5
**TS$_{P-Q}$**	36.1	33.1	24.6	31.4

Supplementary Table 33. The absolute electronic (in Hartrees), relative electronic and free energies (in kcal/mol) of the pathway **i-Ph** and the lowest-energy side-reaction oxidative coupling pathway (via **TS**$_{P-Q}$) for the Rh(I)-catalyzed cycloisomerization of a 1,6-allenyne (**1a**) in gas phase by PW6B95-D3 method. PPh_3 was used as the additional ligand.

	$E_{PW6B95-D3}$	$\triangle E_{PW6B95-D3}$	$\triangle G_{PW6B95-D3}$[a]
**APh**	-6626.786224	—	—
**PPh$_3$**	-1037.526824	—	—
**TSPh$_{A-B(R)}$**	-6626.746264	25.1	24.5
**BPh$_{(R)}$**	-6626.792592	-4.0	0.8
**CPh**	-6626.798945	-8.0	-4.5
**DPh**	-6626.779089	4.5	10.9
**TSPh$_{D-E}$**	-6626.736099	31.5	33.6
**TSPh$_{E-F}$**	-6626.749481	23.1	24.4
**GPh**	-6626.787333	-0.7	3.4
**TSPh$_{G-H}$**	-6626.741461	28.1	31.7
**A′**	-5589.188229	44.7	20.6
**TS′$_{A-B(R)}$**	-5589.147955	69.9	45.1
**B′$_R$**	-5589.193389	41.4	19.3
**D′**	-5589.219122	25.3	3.1
**TS′$_{D-E}$**	-5589.190733	43.1	17.9
**E′**	-5589.195494	40.1	17.2
**TS′$_{E-F}$**	-5589.175096	52.9	28.4
**F′**	-5589.198517	38.2	17.8
**G′**	-5589.233329	16.4	-2.0
**TS′$_{G-H}$**	-5589.183982	47.3	24.7
**H′**	-5589.262795	-2.1	-19.9
**TS′$_{H-I}$**	-5589.23224	17.0	-2.7
**I′**	-5589.279565	-12.7	-30.9
**TS$_{P-Q}$**	-5589.176107	52.3	33.2

$$[\{Rh(CH_2CH_2)_2Cl\}_2] \xrightarrow{\Delta G_{soln}=4.2\ Kcal/mol} 2Rh(CH_2CH_2)_2Cl$$

Supplementary Table 34. The absolute electronic and free energies (in Hartrees) of $[\{Rh(CH_2CH_2)_2Cl\}_2]$, $\{Rh(CH_2CH_2)_2Cl\}$, Ag^+, **L4**, AgCl and CH_2CH_2 in gas (by the M06-L method) and solution (by the SMD M06-L//M06-L method) phases.

	E	E+ZPE	G	E_{soln}
$[\{Rh(CH_2CH_2)_2Cl\}_2]$[a]	-1456.038140	-1455.819845	-1455.866279	-1456.053875
$\{Rh(CH_2CH_2)_2Cl\}$	-728.001211	-727.892571	-727.926463	-728.012389
Ag^+	-146.761079	-146.761079	-146.777685	-146.847030
**L4**	-4016.016356	-4014.380009	-4014.500044	-4016.035323
AgCl	-607.265717	-607.265006	-607.289300	-607.281626
CH_2CH_2	-78.565782	-78.514787	-78.536318	-78.565208

a: **$[\{Rh(CH_2CH_2)_2Cl\}_2]$** has a very small imaginary frequency (-30.2), but we cannot eliminate that by several ways.

Supplementary Table 35. The relative electronic and free energies (in kcal/mol) of dissociation from $[\{Rh(CH_2CH_2)_2Cl\}_2]$ to form $\{Rh(CH_2CH_2)_2Cl\}$ in gas (by the M06-L method) and solution (by the SMD M06-L//M06-L method) phases.

	△E	△E+ZPE	△G	△E_{soln}	△G_{soln}[a]
$[\{Rh(CH_2CH_2)_2Cl\}_2]$	—	—	—	—	—
$\{Rh(CH_2CH_2)_2Cl\}$	22.4	21.8	8.4	18.3	4.2

a. The free-energy corrections were taken from those obtained by the M06-L method in gas phase.

$$Rh(CH_2CH_2)_2Cl + Ag^+ + L4 + PPh_3 + 1a \xrightarrow{\Delta G_{soln}=-94.4\ Kcal/mol} APh + AgCl + CH_2CH_2$$

Supplementary Table 36. The relative electronic and free energies (in kcal/mol) for the formation of **APh** from the above exchange reaction in gas (by the M06-L method) and solution (by the SMD M06-L//M06-L method) phases. PPh_3 was used as the additional ligand.

	△E	△E+ZPE	△G	△E_{soln}	△G_{soln}[a]
**APh**	-174.9	-171.0	-140.8	-128.5	-94.4

Supplementary Figure 1. ^{1}H and ^{13}C-NMR of **2a**

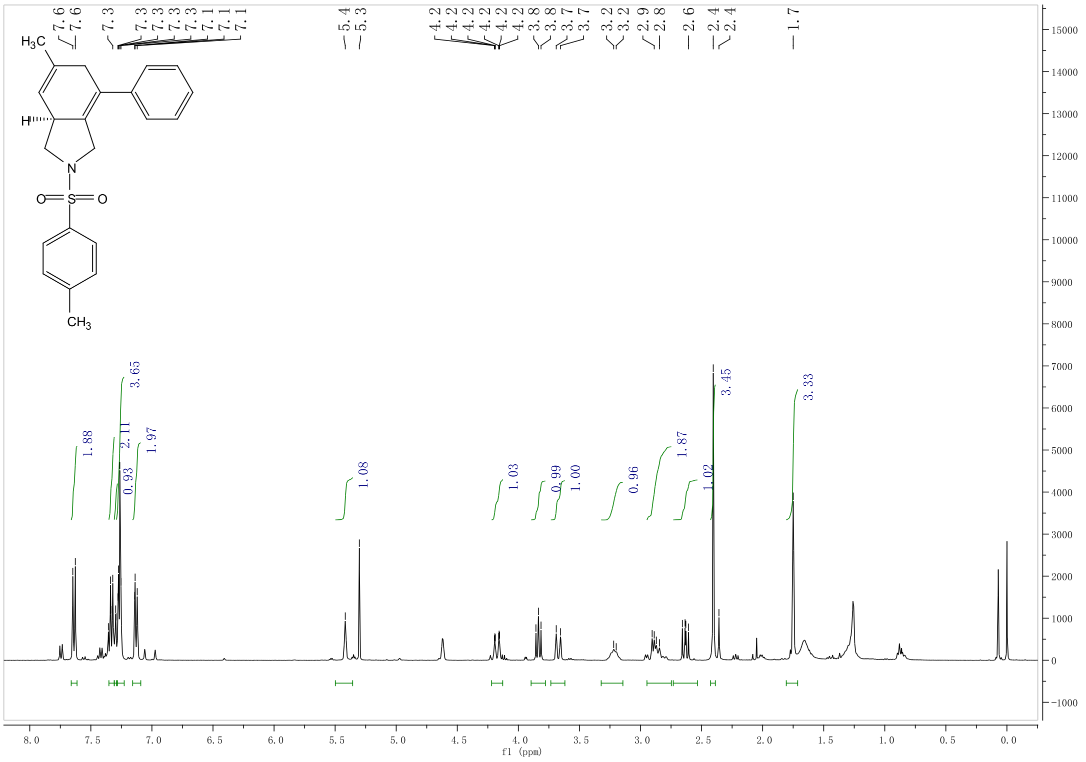

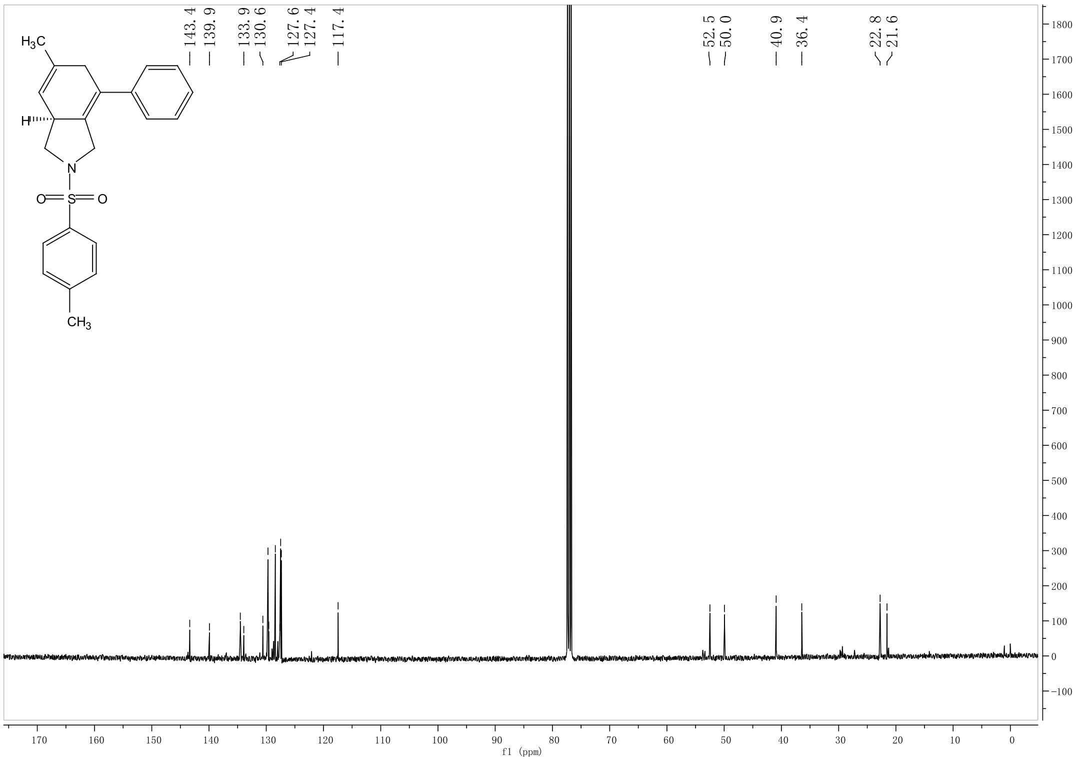

Supplementary Figure 2. ^{1}H and ^{13}C-NMR of **[D6]-2a**

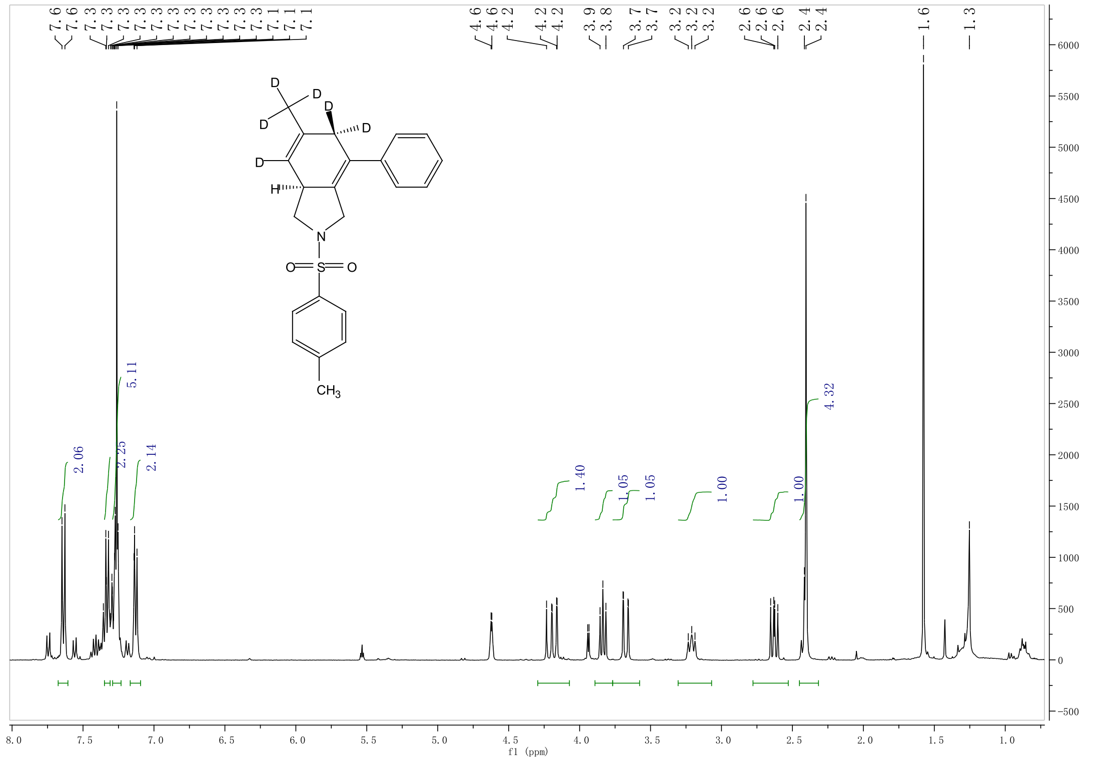

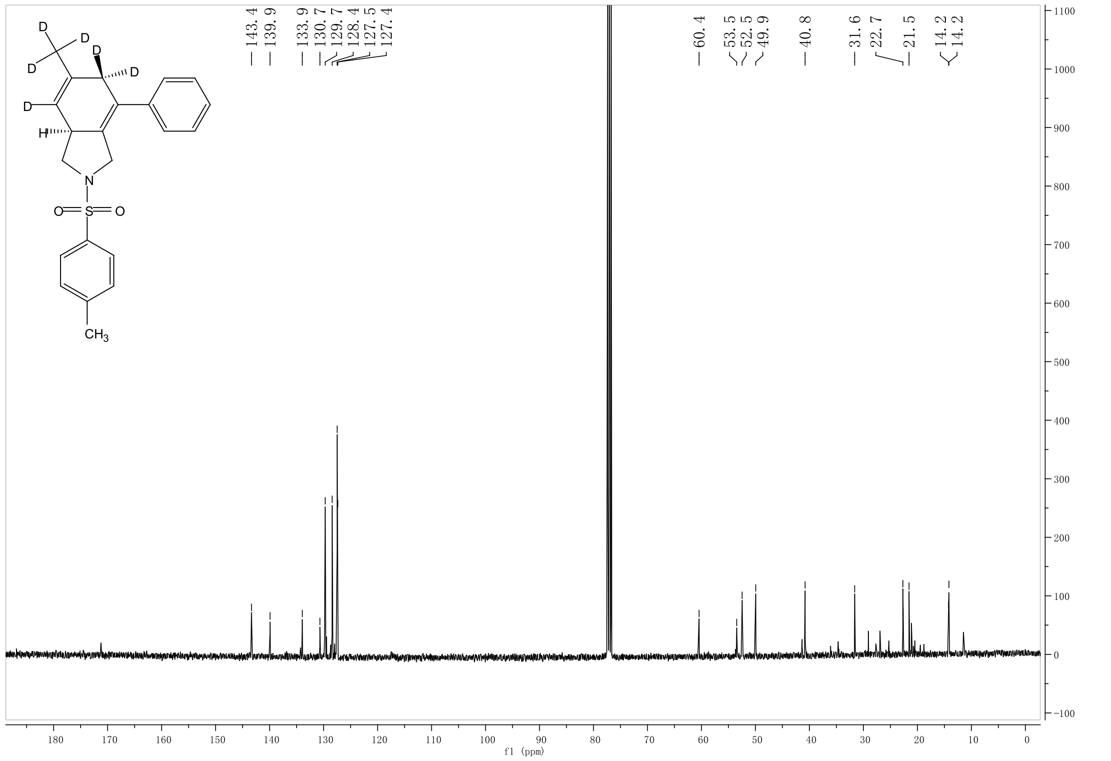

Supplementary Figure 3. ^{1}H and ^{13}C-NMR of **2b**

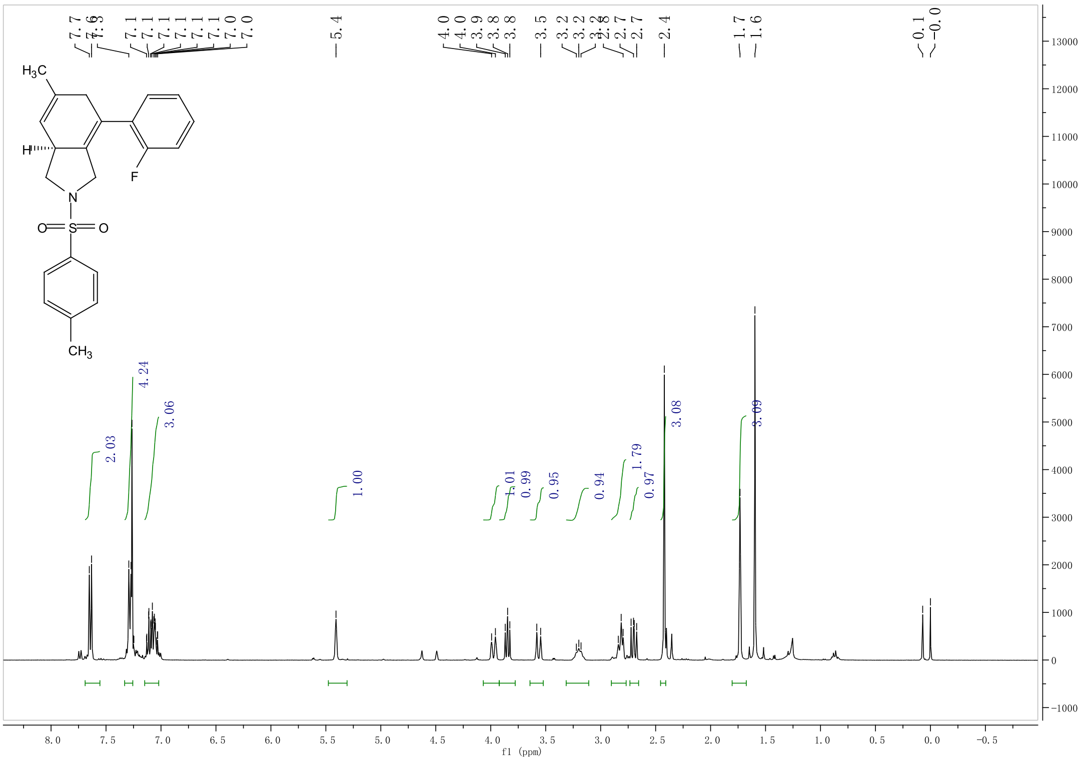

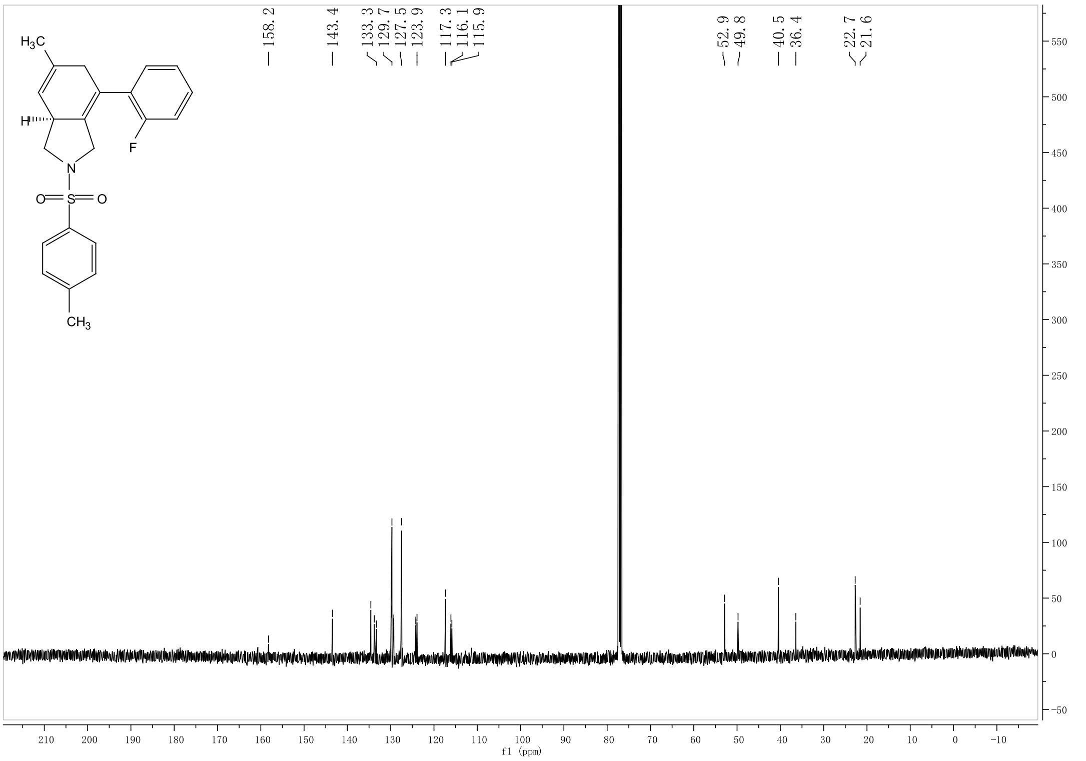

Supplementary Figure 4. ^1H and ^{13}C-NMR of **2c**

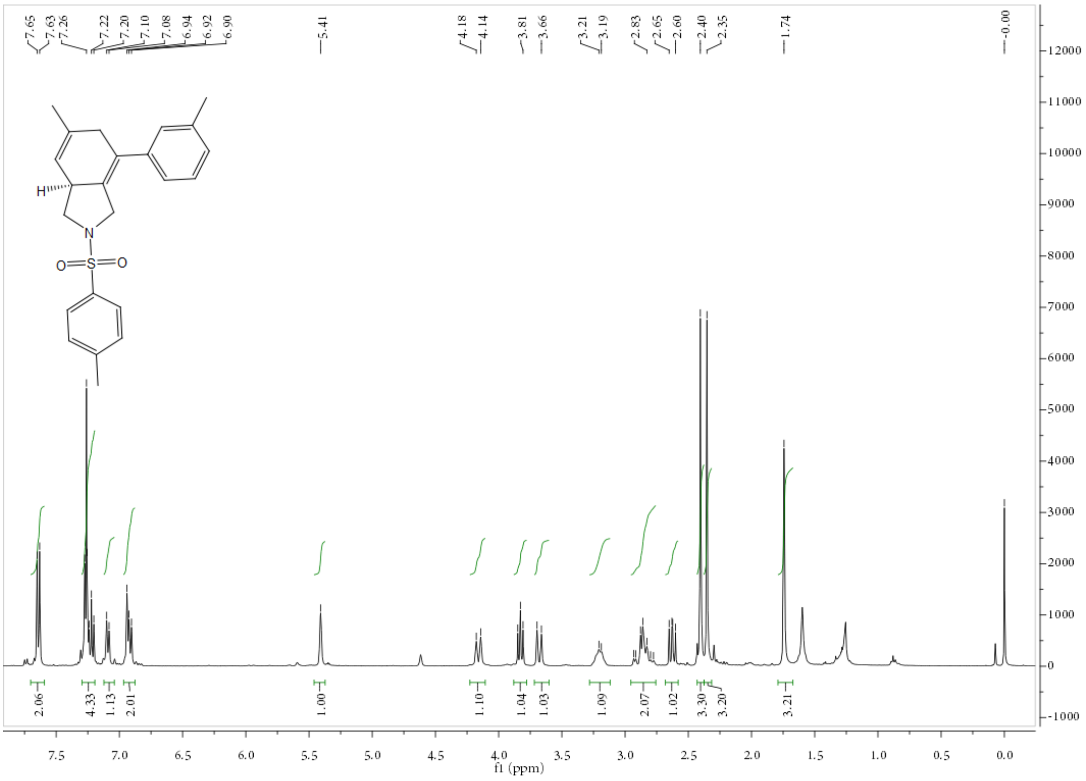

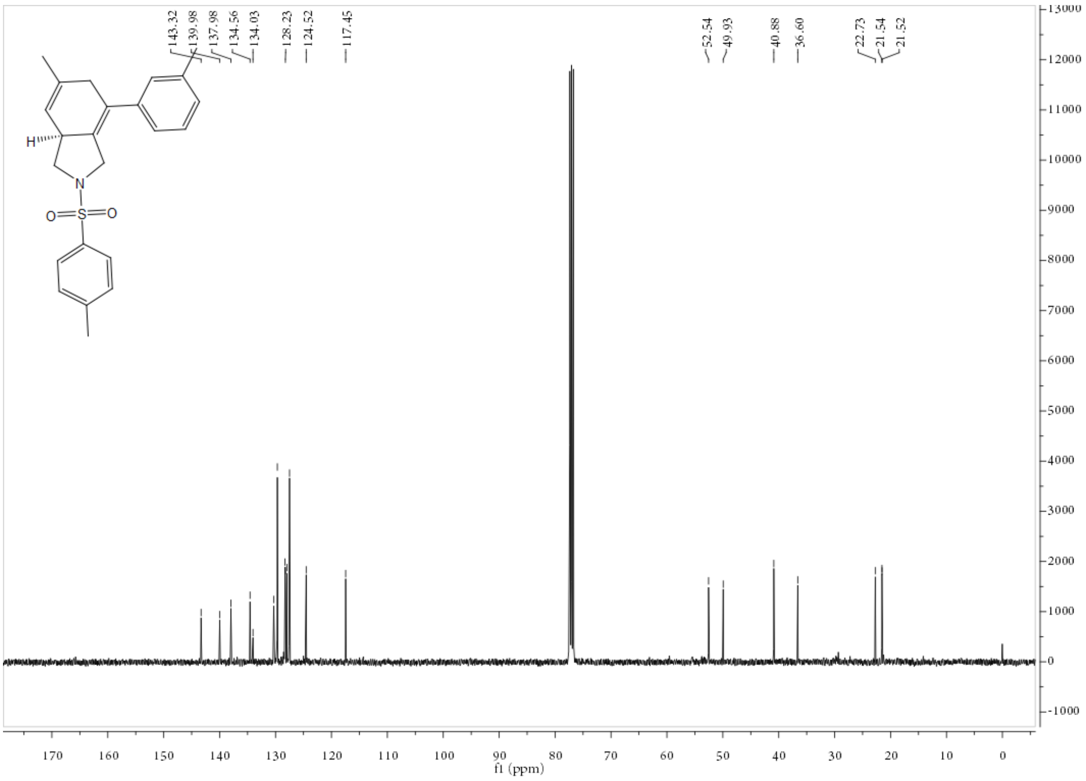

Supplementary Figure 5. ^{1}H and ^{13}C-NMR of **2d**

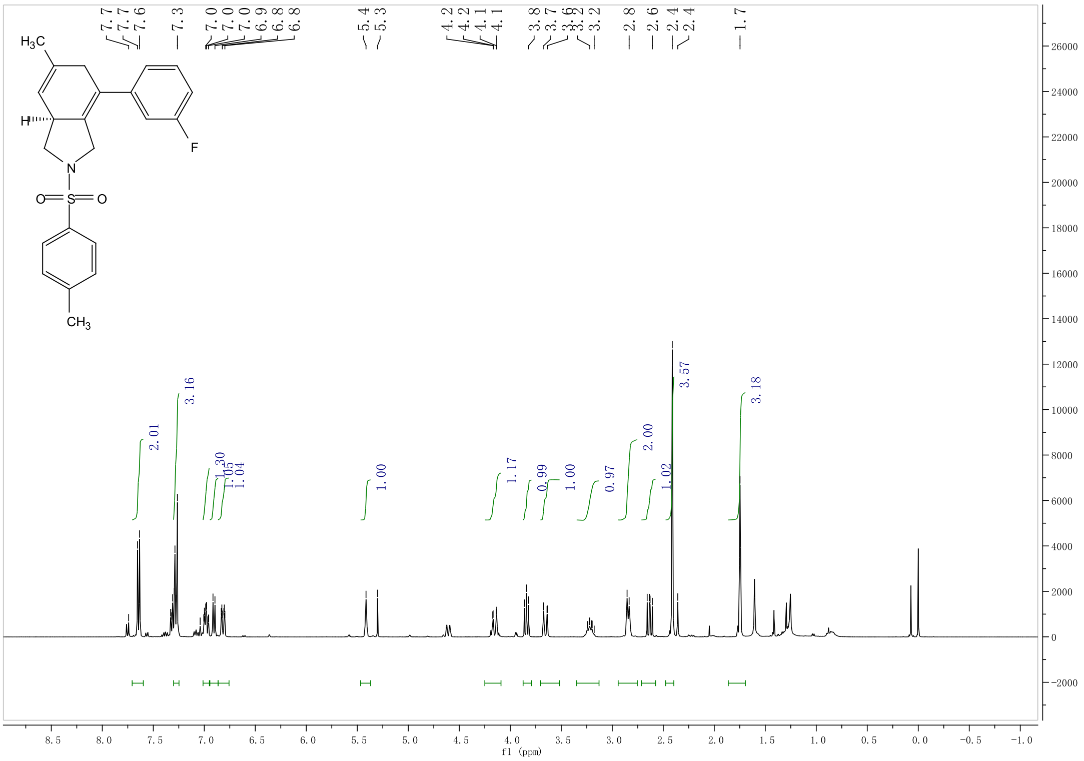

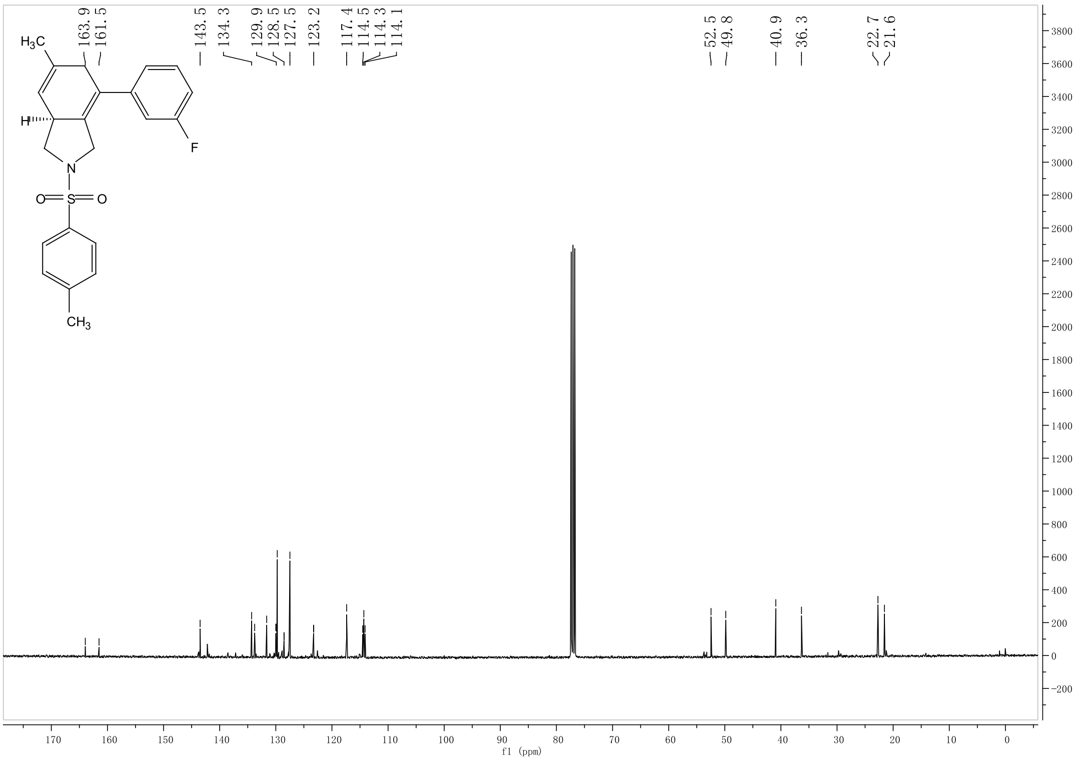

Supplementary Figure 6. ^{1}H and ^{13}C-NMR of **2e**

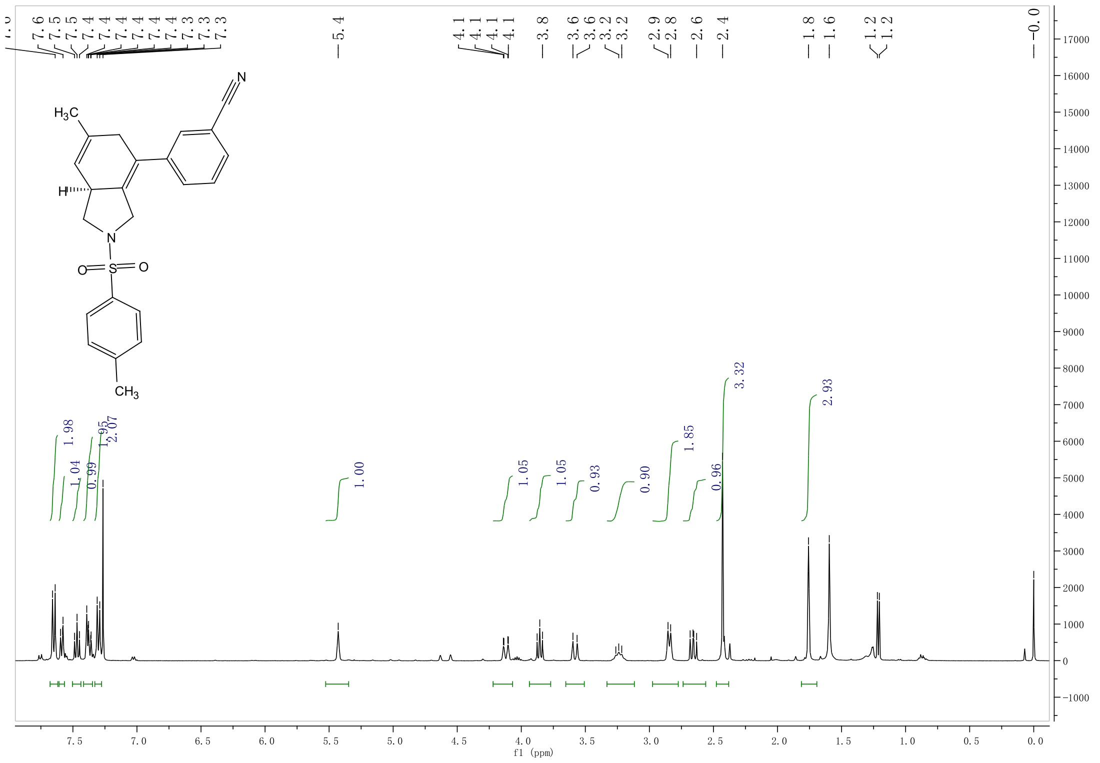

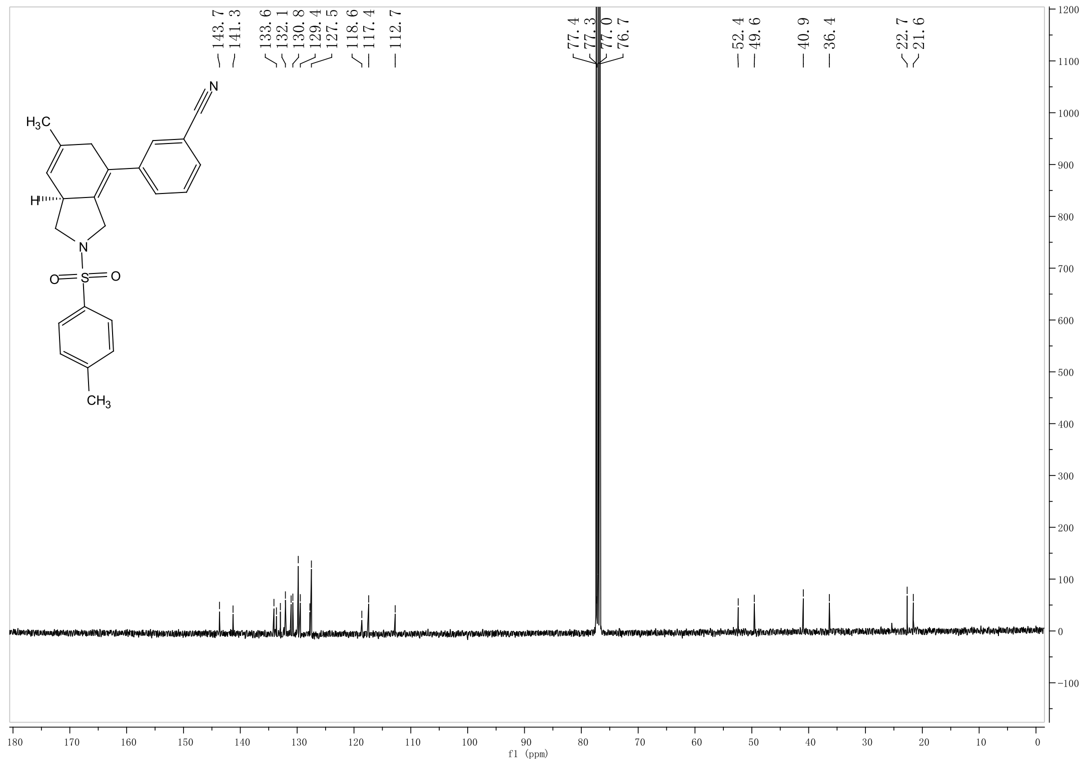

Supplementary Figure 7. ^{1}H and ^{13}C-NMR of **2f**

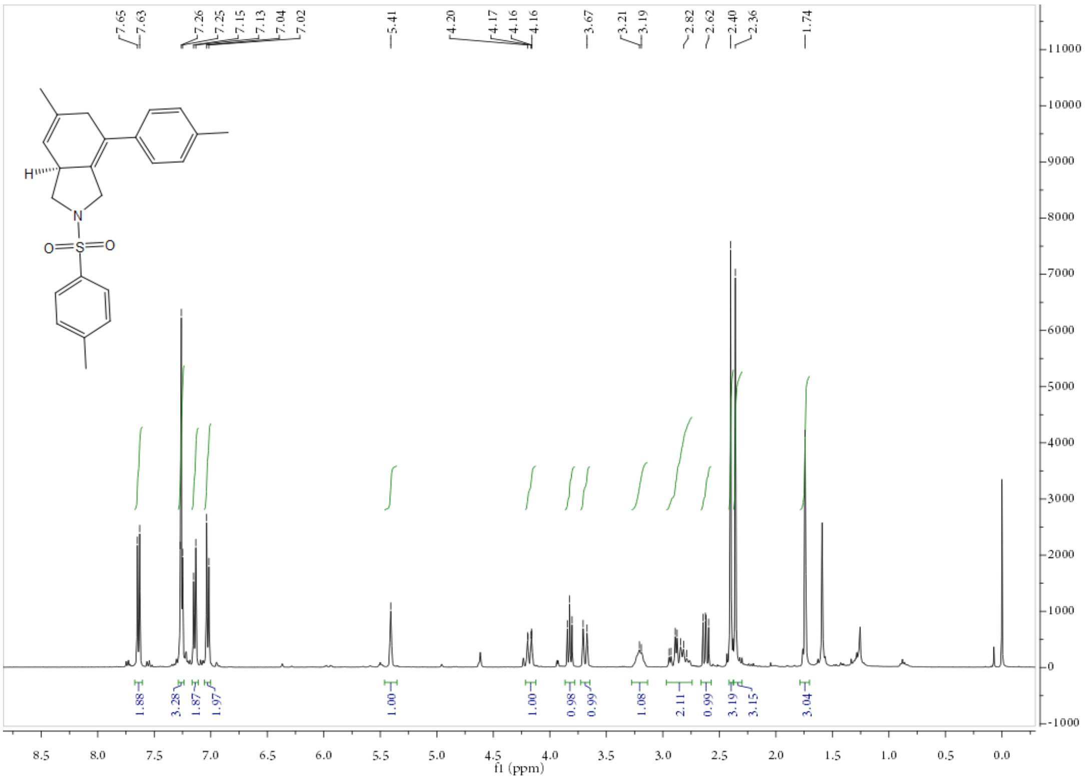

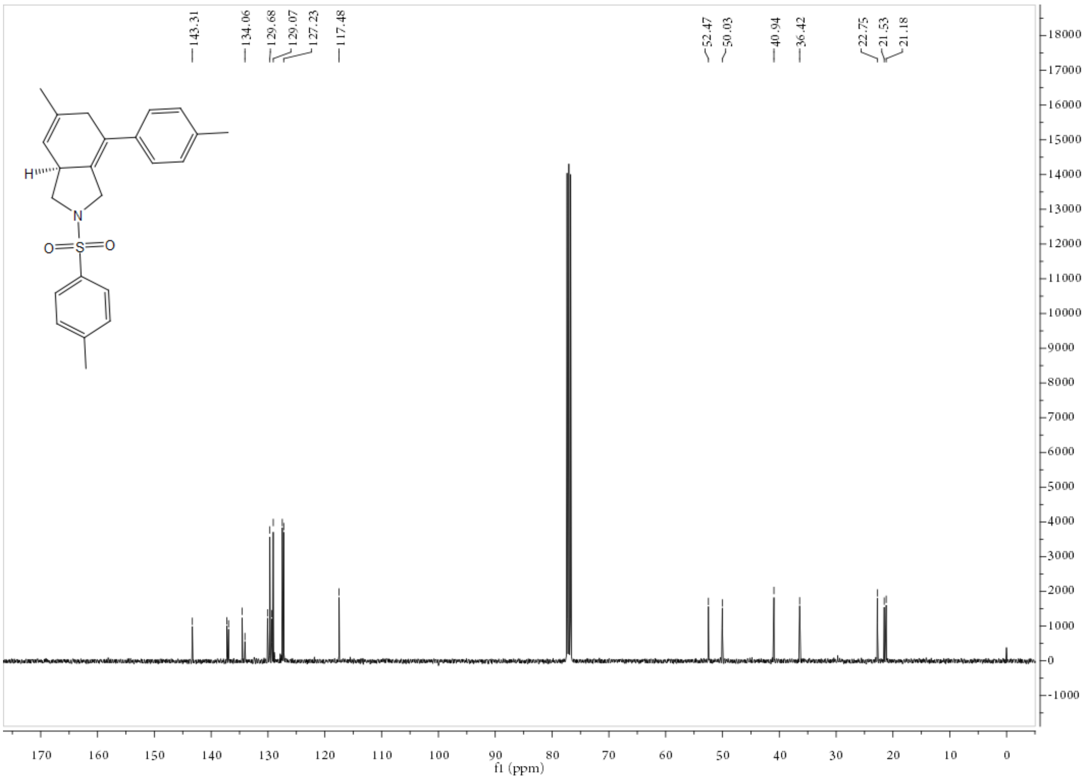

Supplementary Figure 8. ^{1}H and ^{13}C-NMR of **2g**

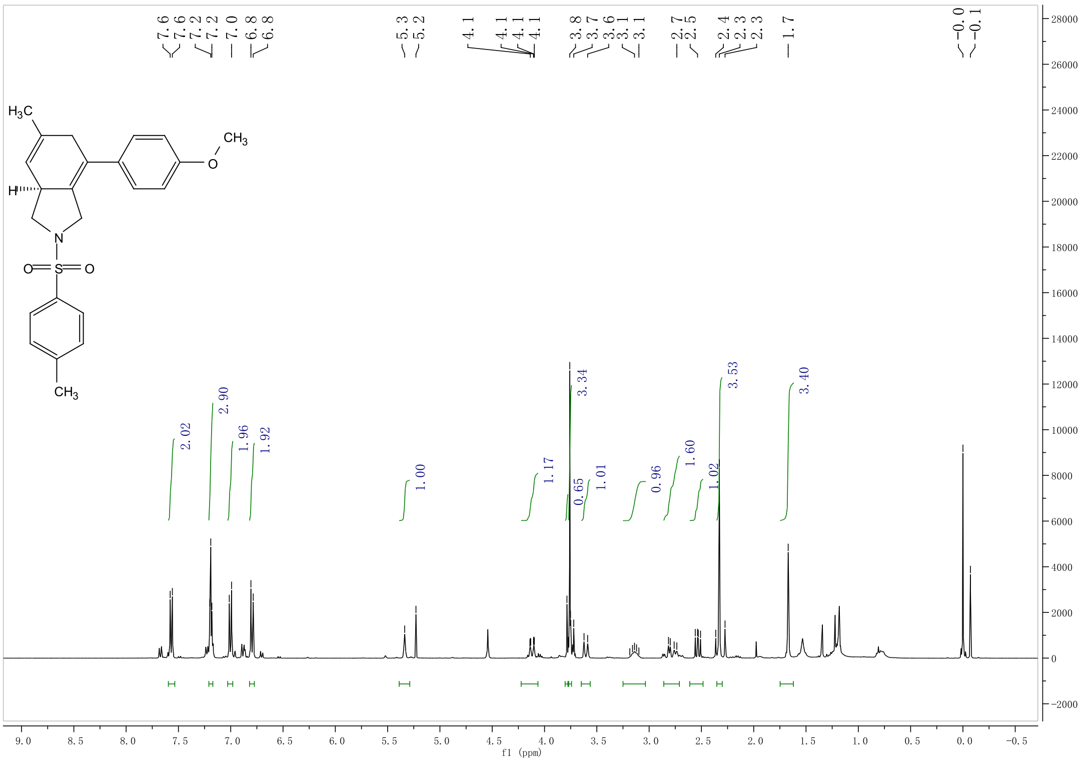

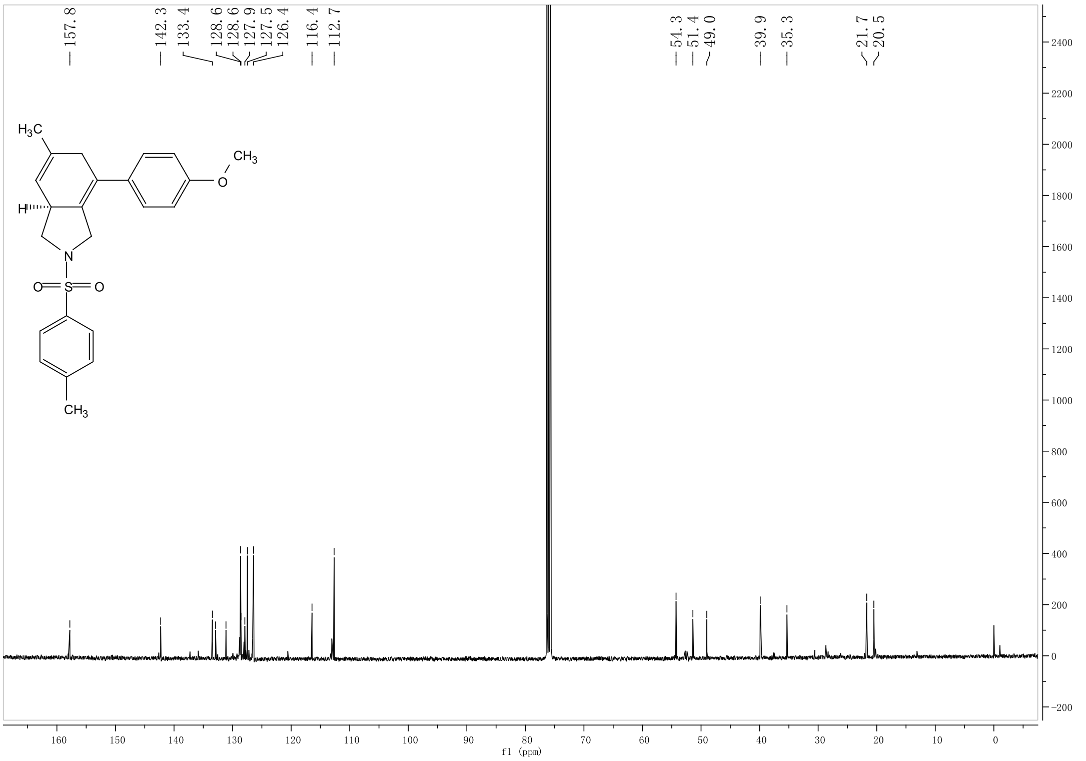

Supplementary Figure 9. ^{1}H and ^{13}C-NMR of **2h**

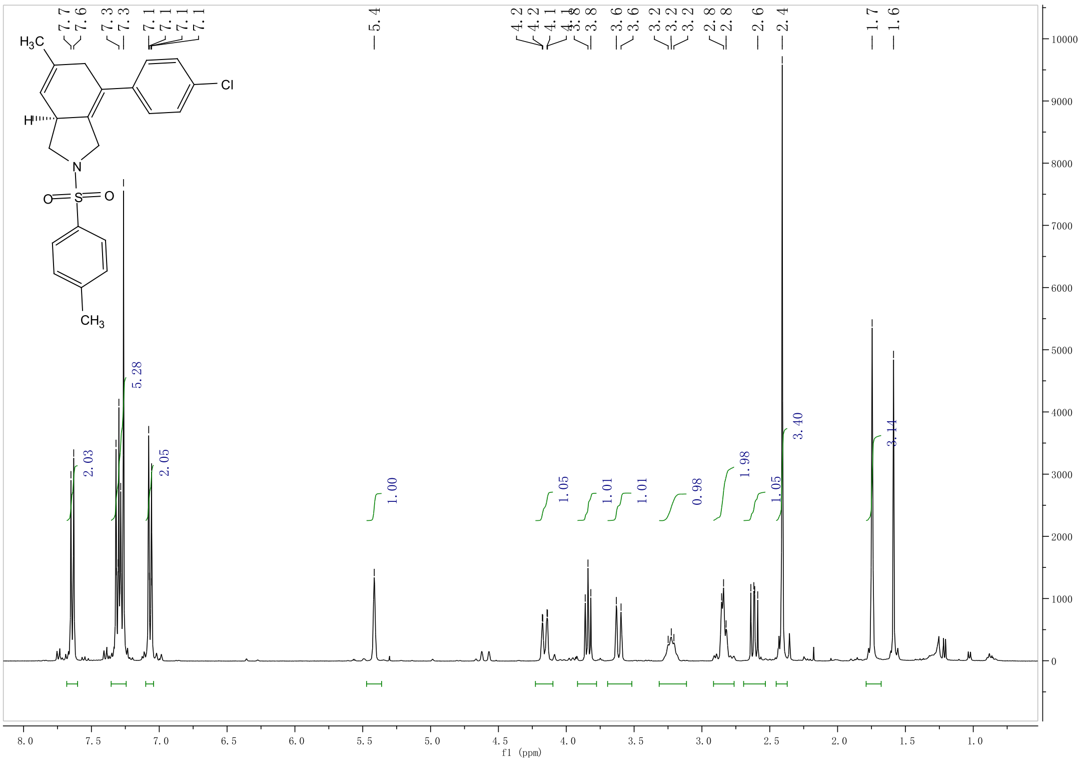

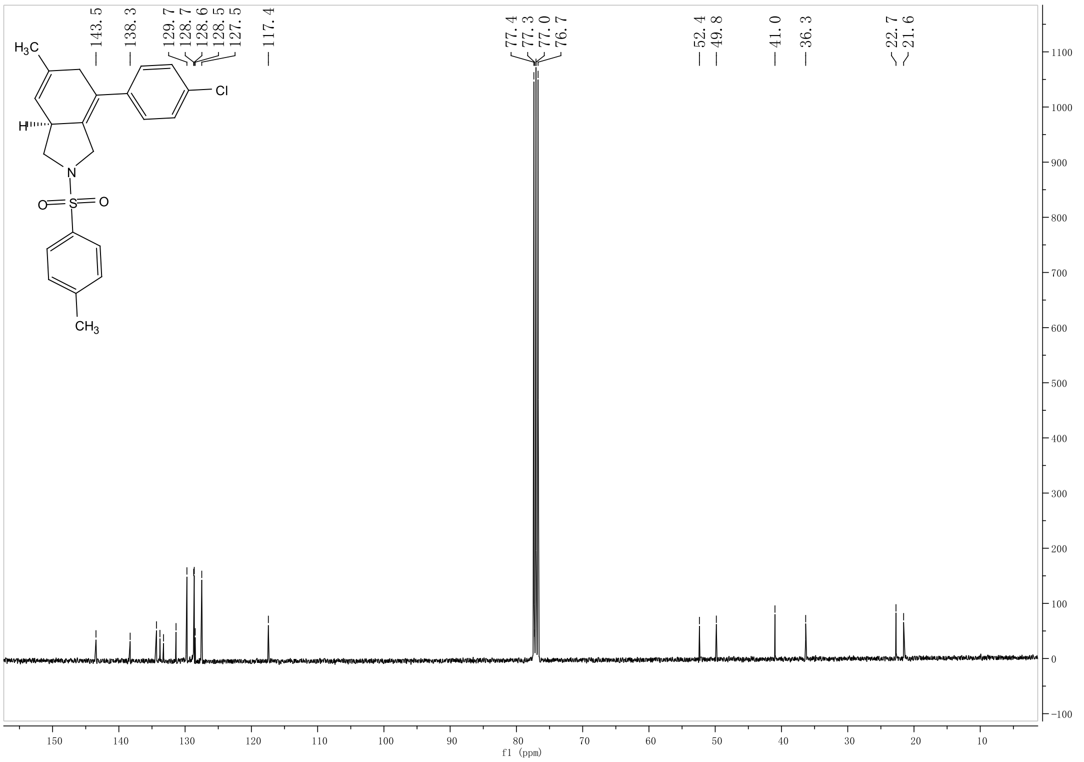

Supplementary Figure 10. ^{1}H and ^{13}C-NMR of **2i**

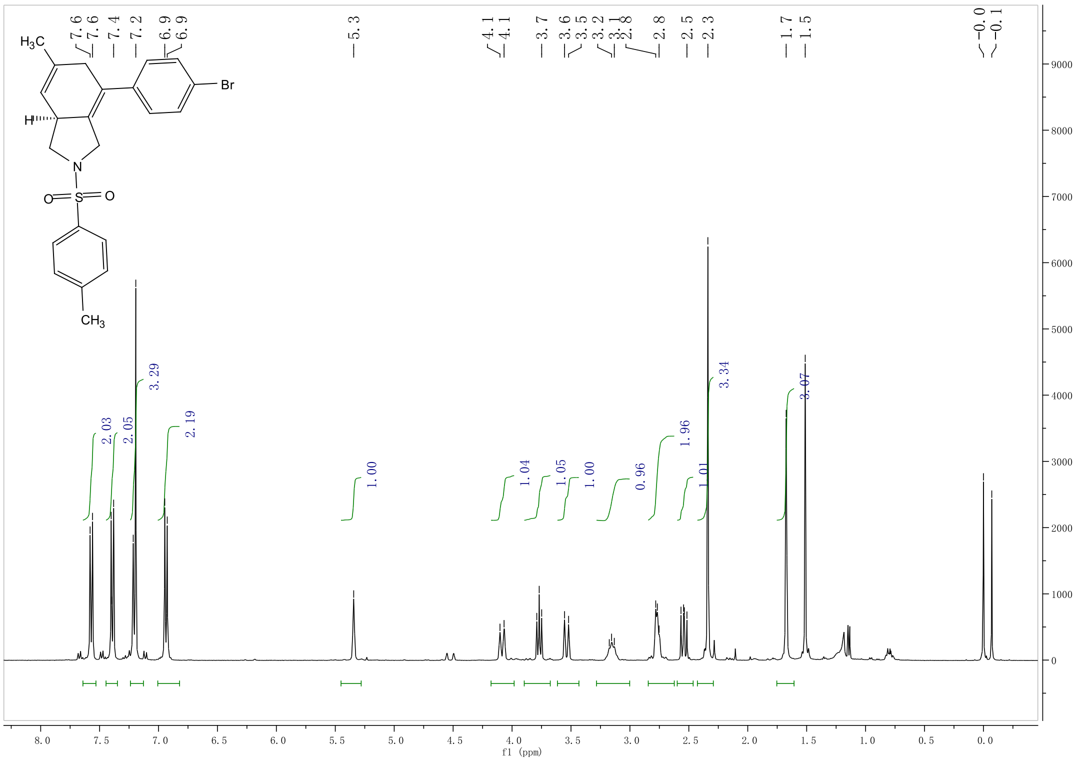

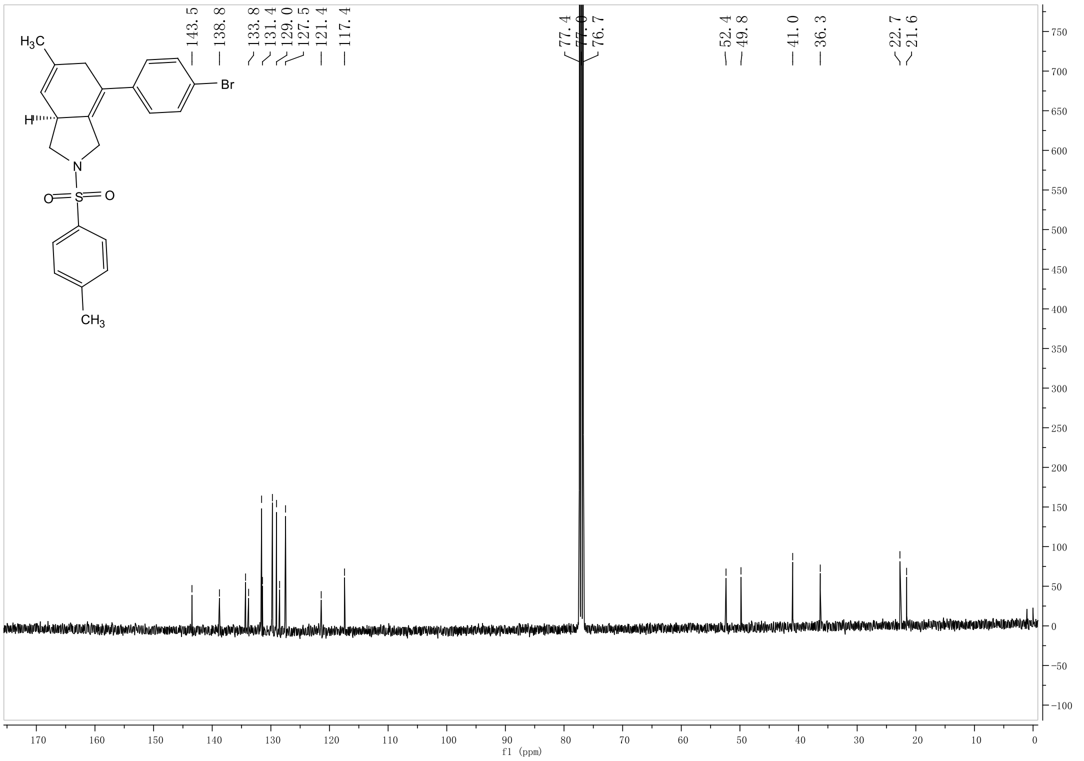

Supplementary Figure 11. ^{1}H and ^{13}C-NMR of **2j**

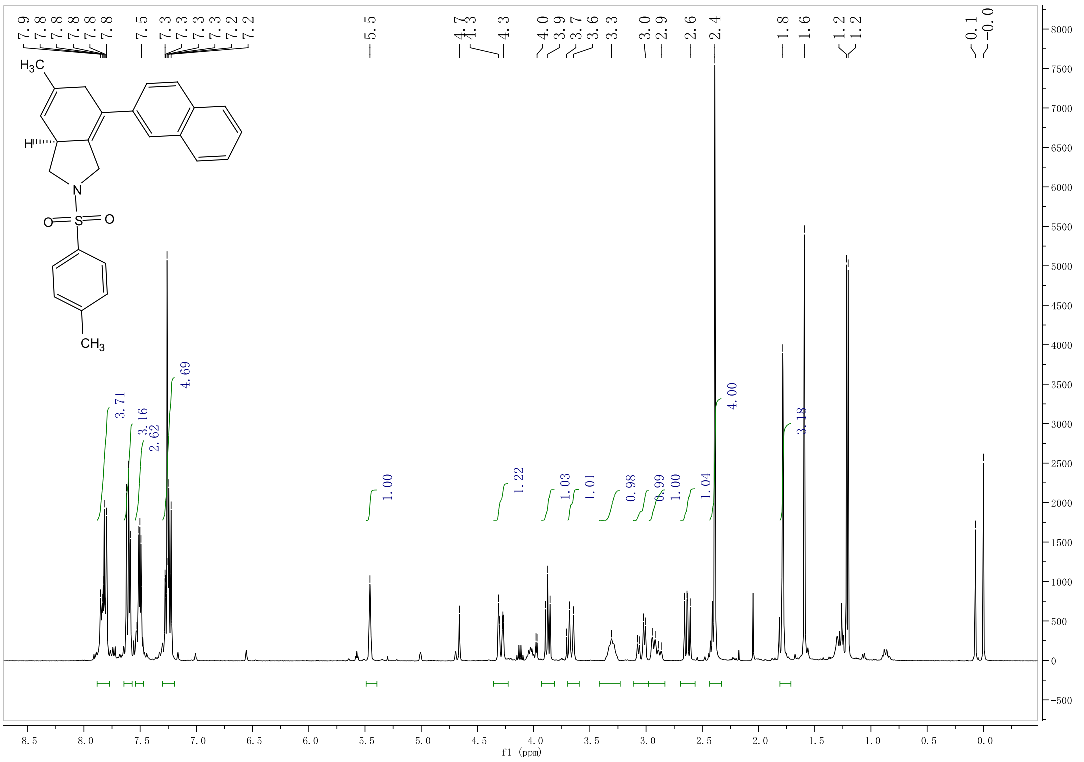

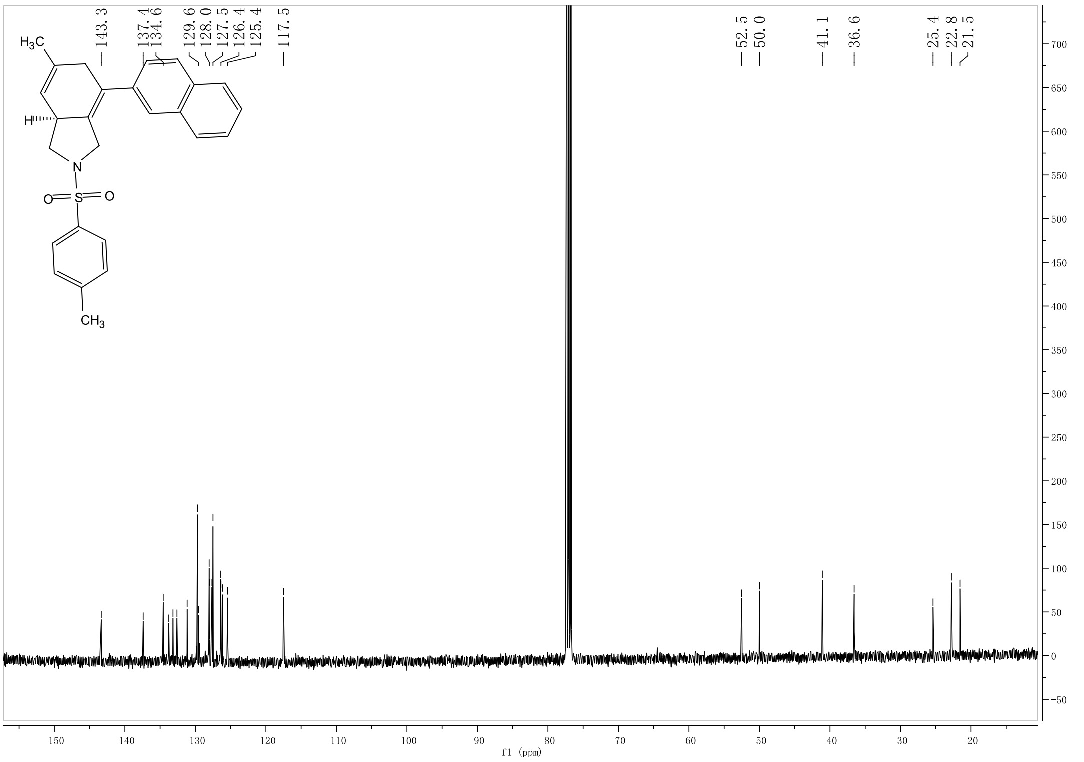

Supplementary Figure 12. ^{1}H and ^{13}C-NMR of **2k**

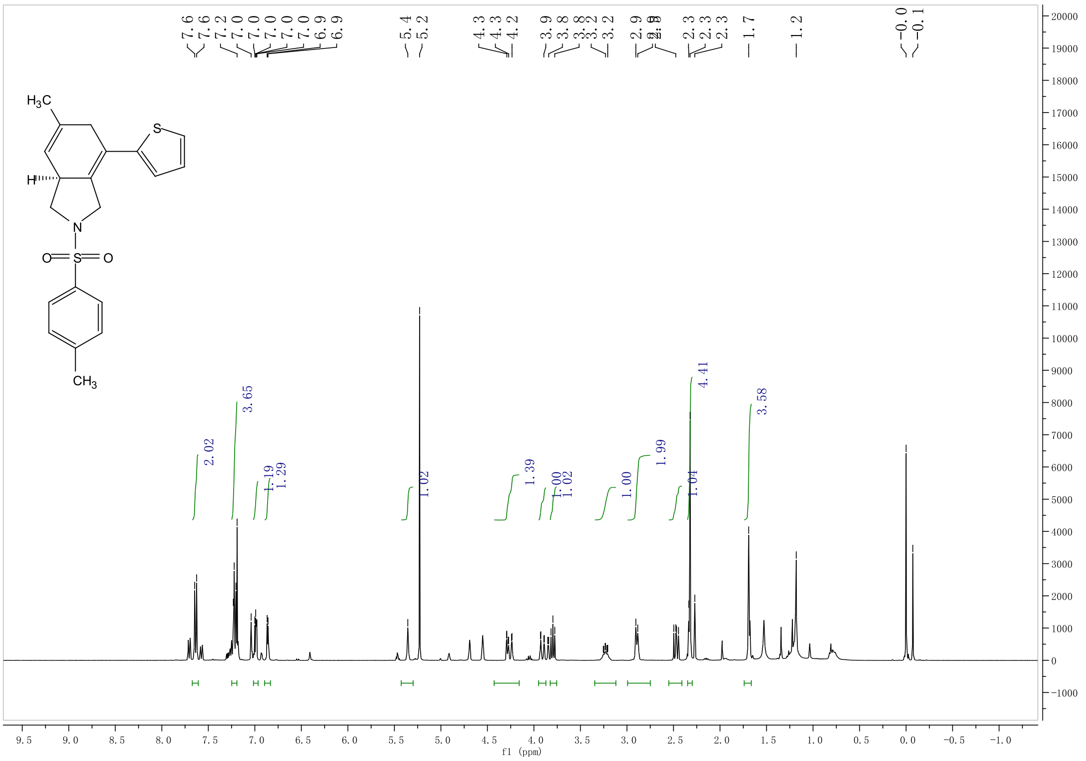

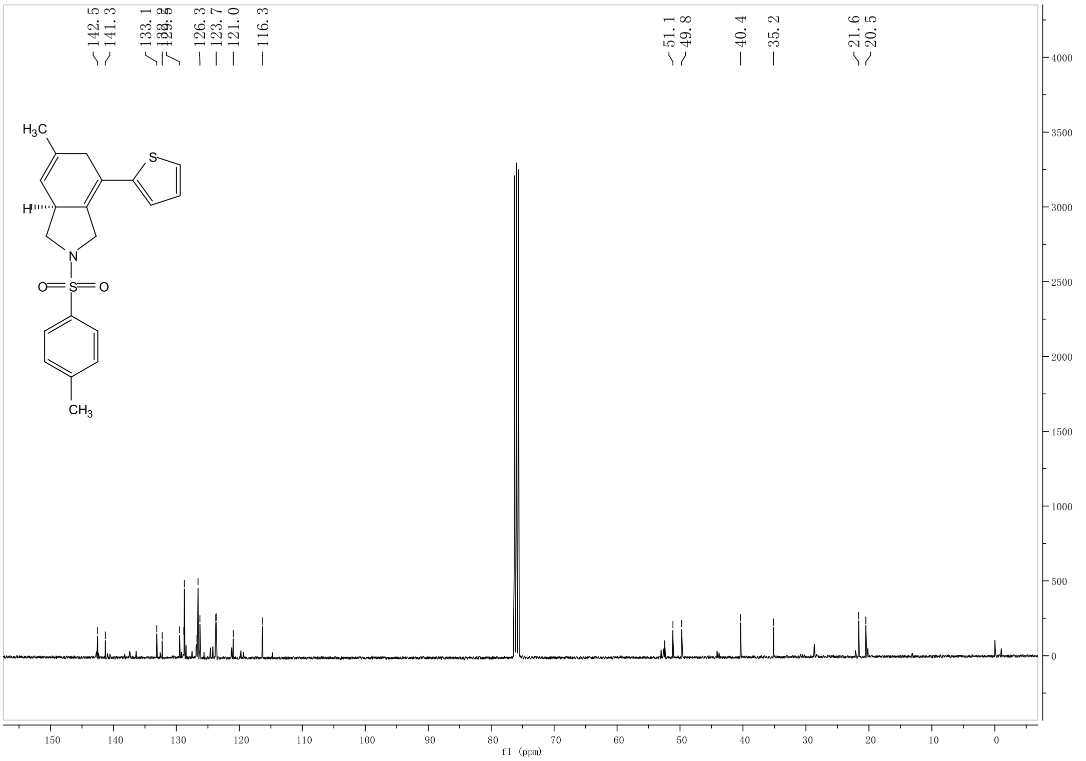

Supplementary Figure 13. ^{1}H and ^{13}C-NMR of **5a**

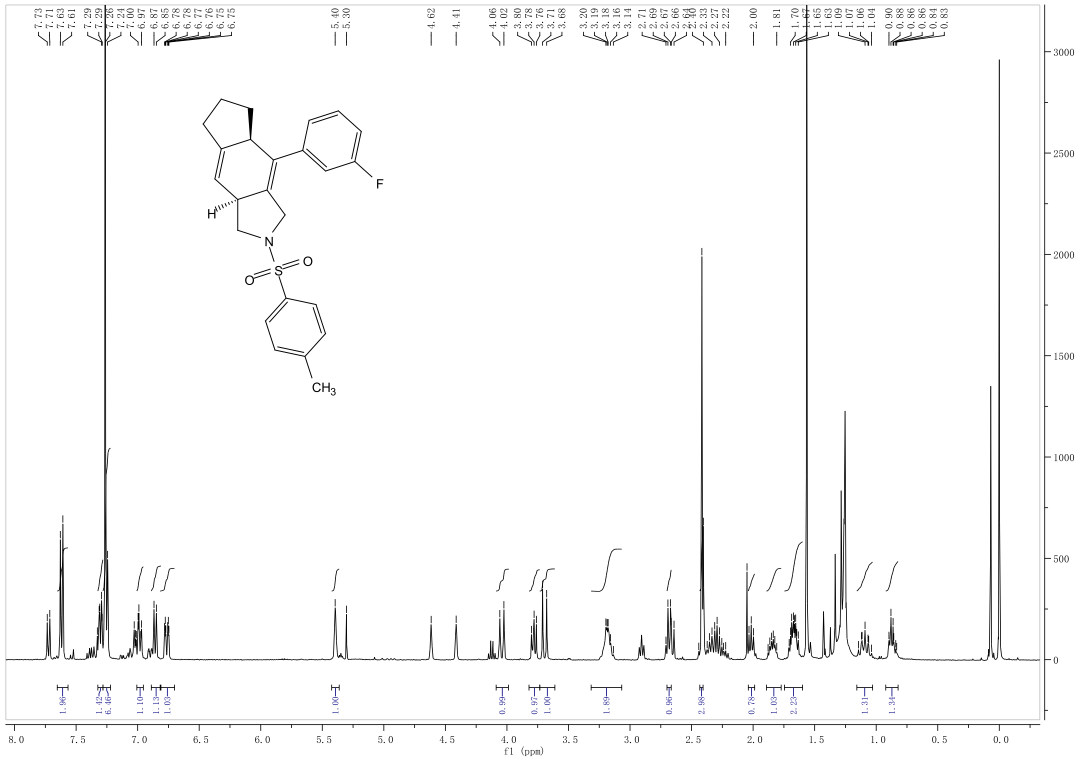

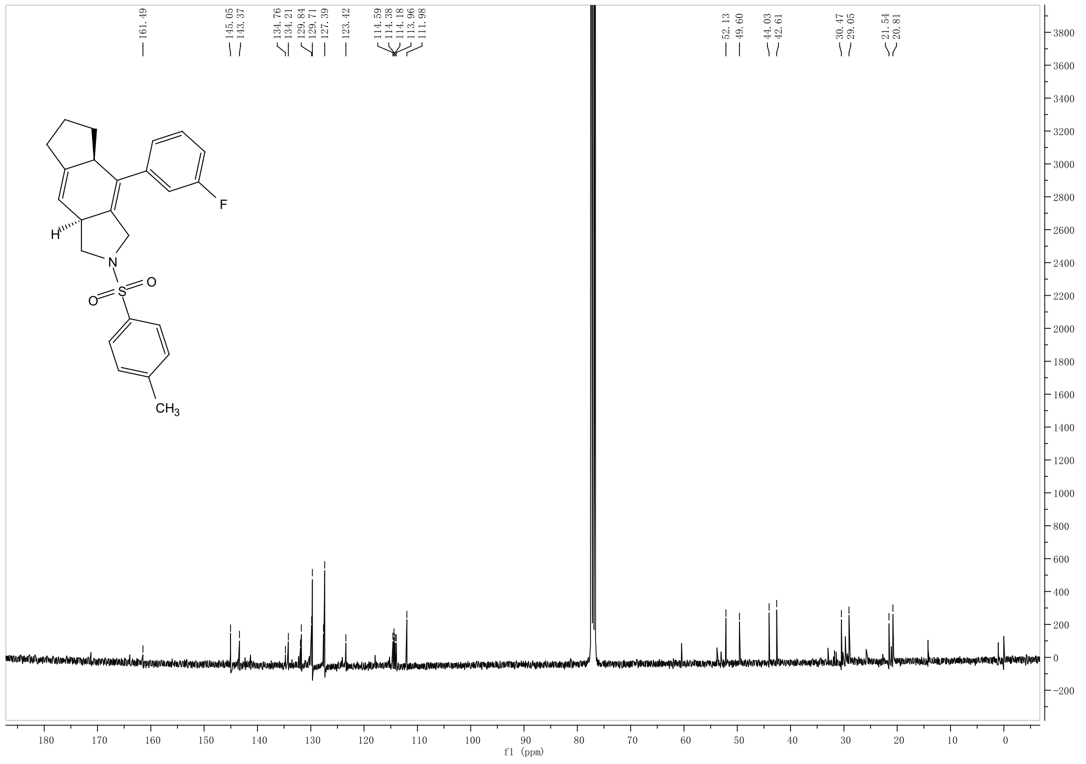

Supplementary Figure 14. ^{1}H and ^{13}C-NMR of **5b**

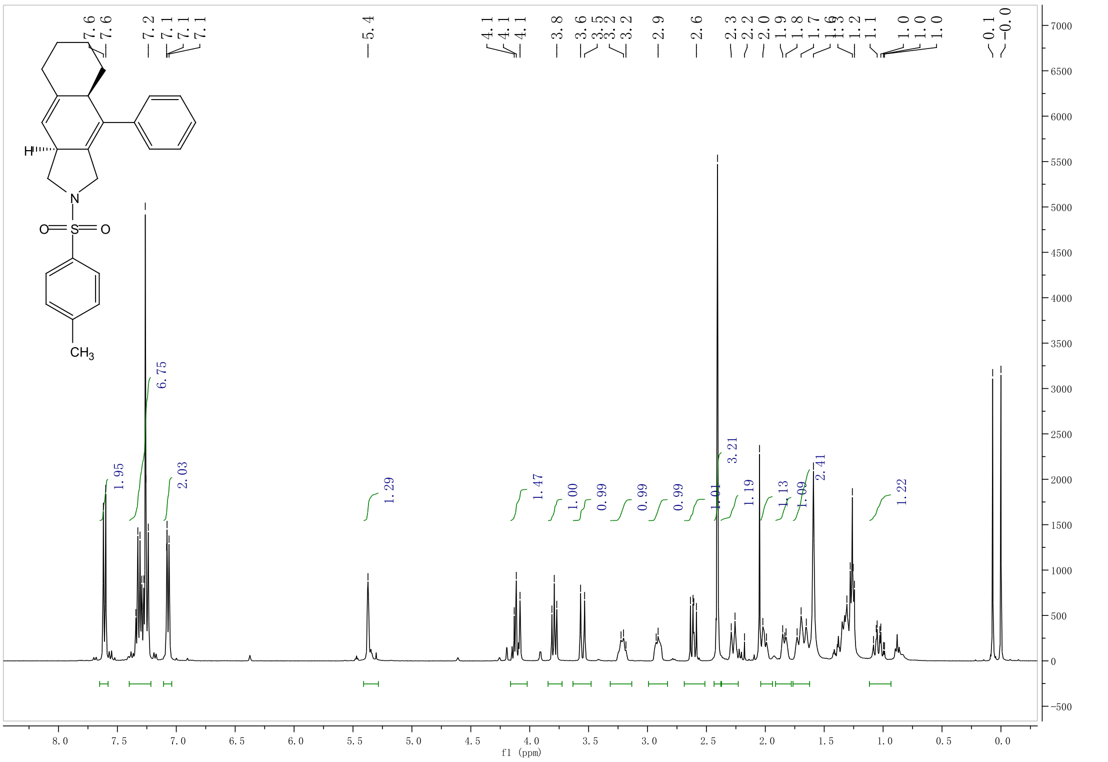

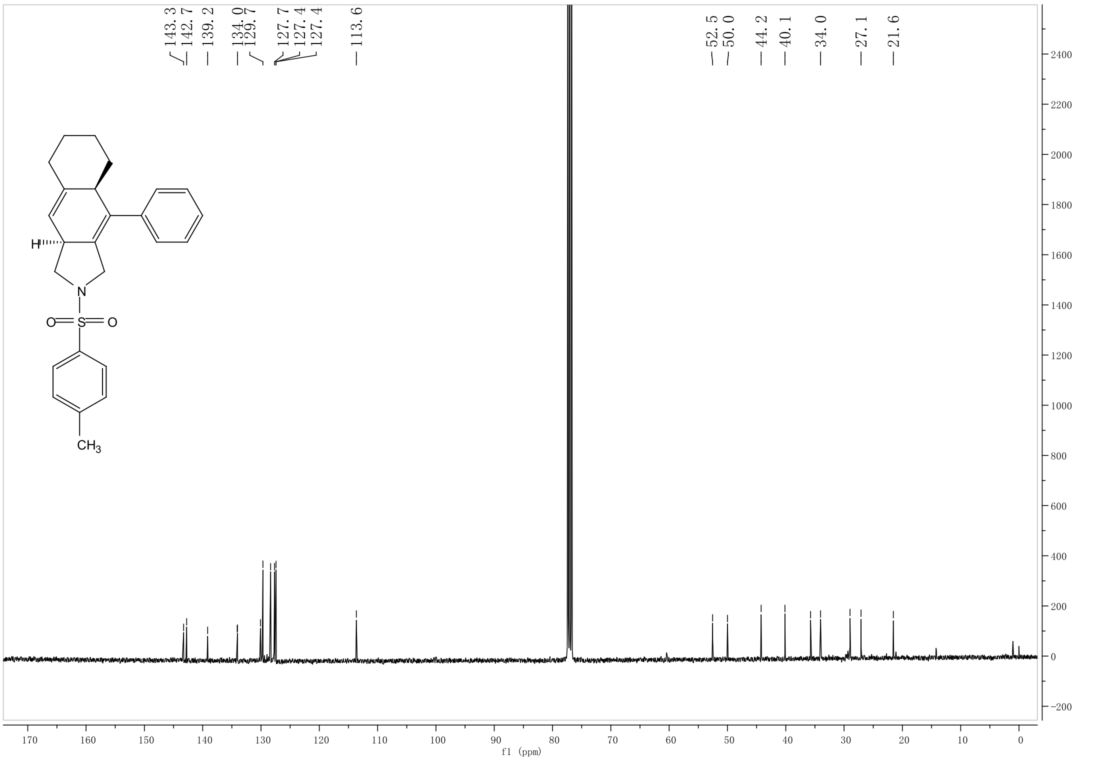

Supplementary Figure 15. ^{1}H and ^{13}C-NMR of **5c**

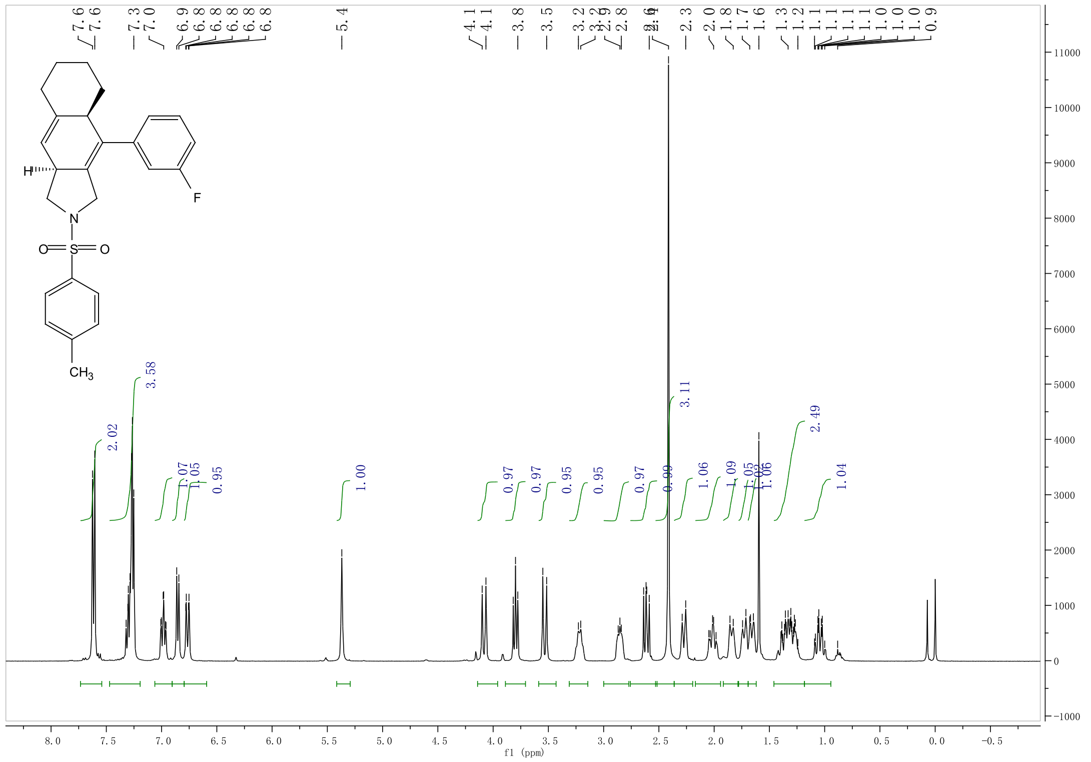

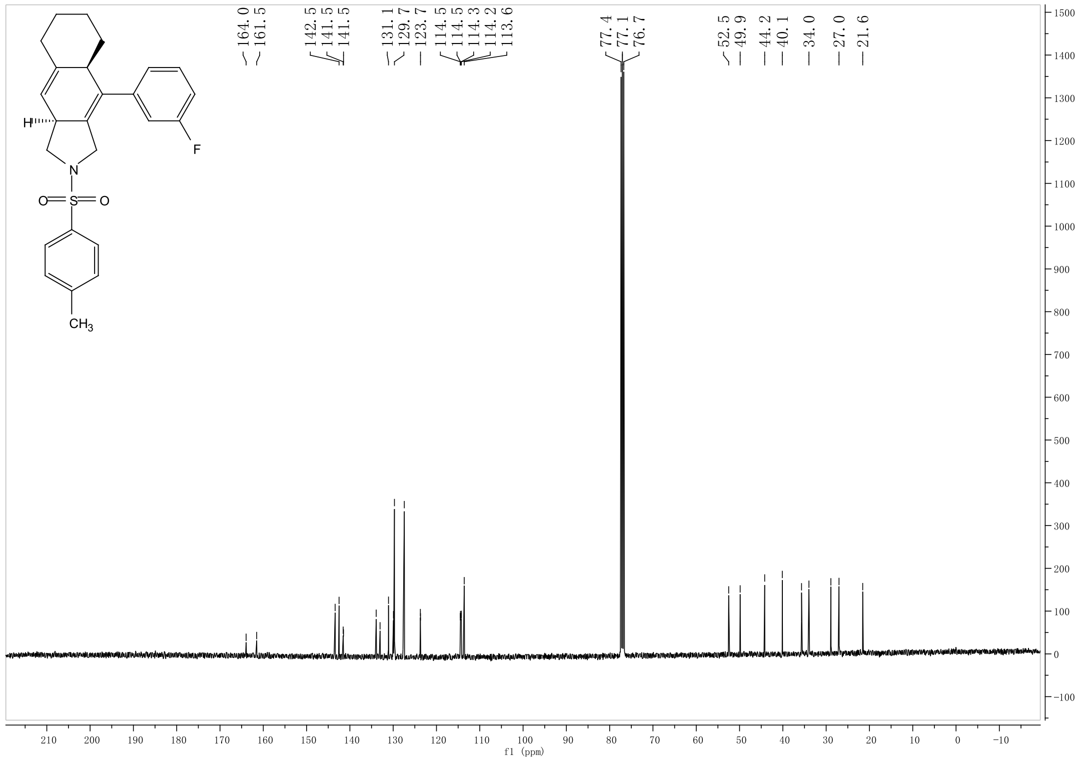

Supplementary Figure 16. NOESY of **5c**

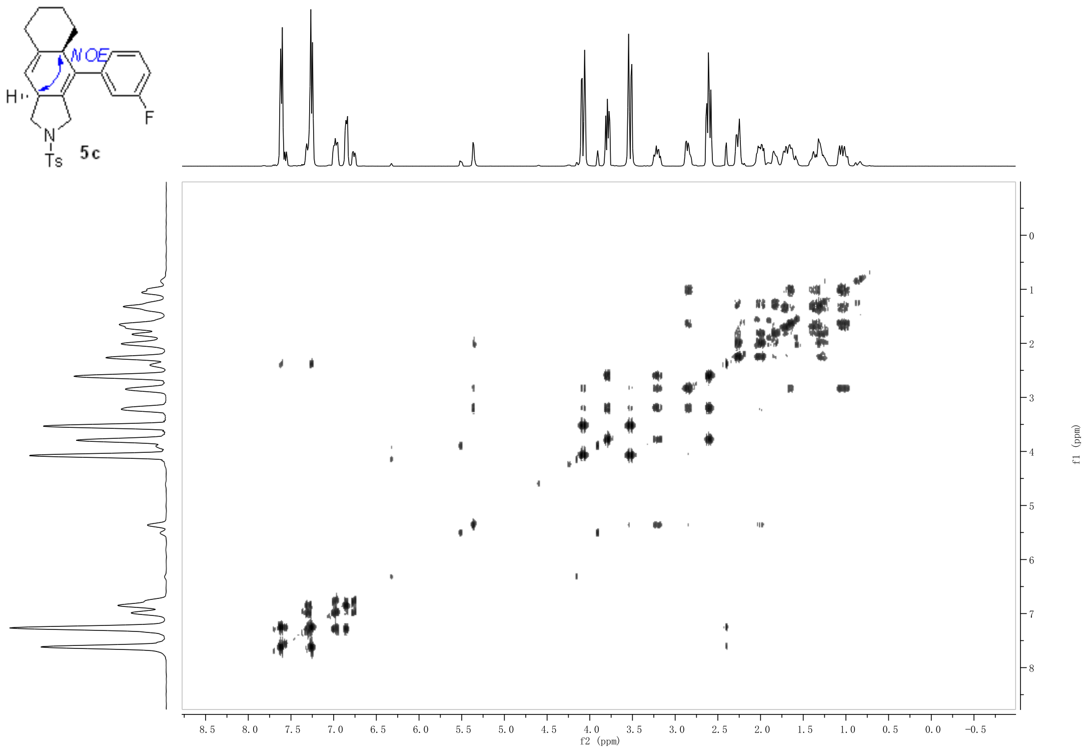

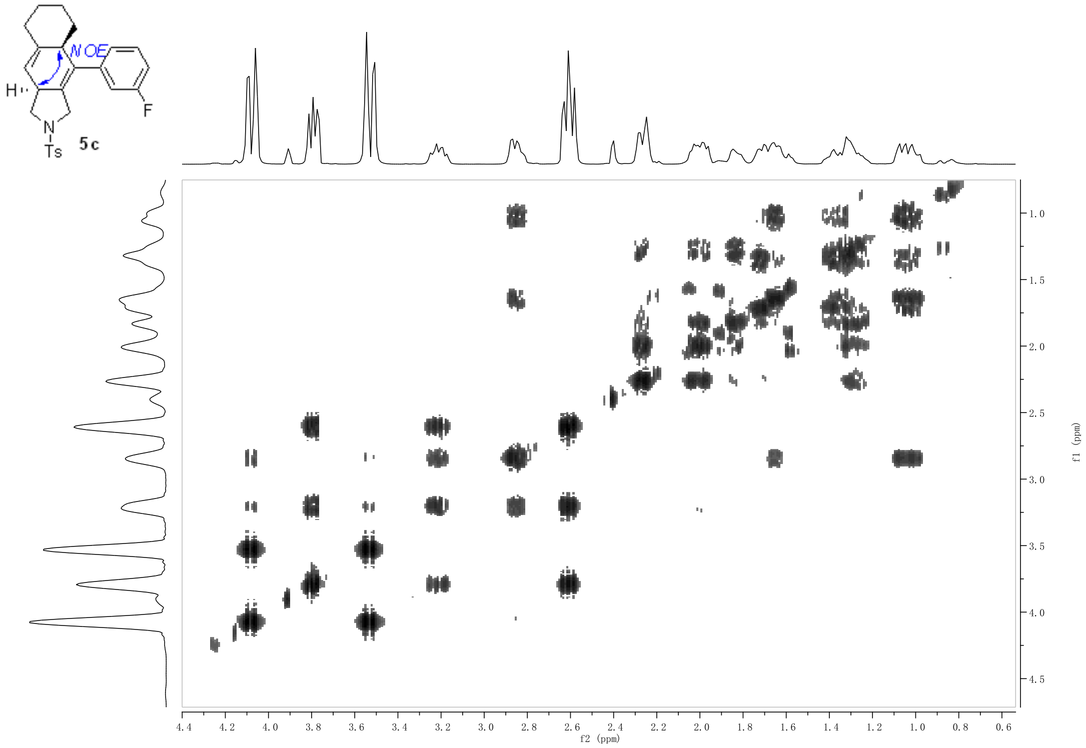

Supplementary Figure 17. ^{1}H and ^{13}C-NMR of **5d**

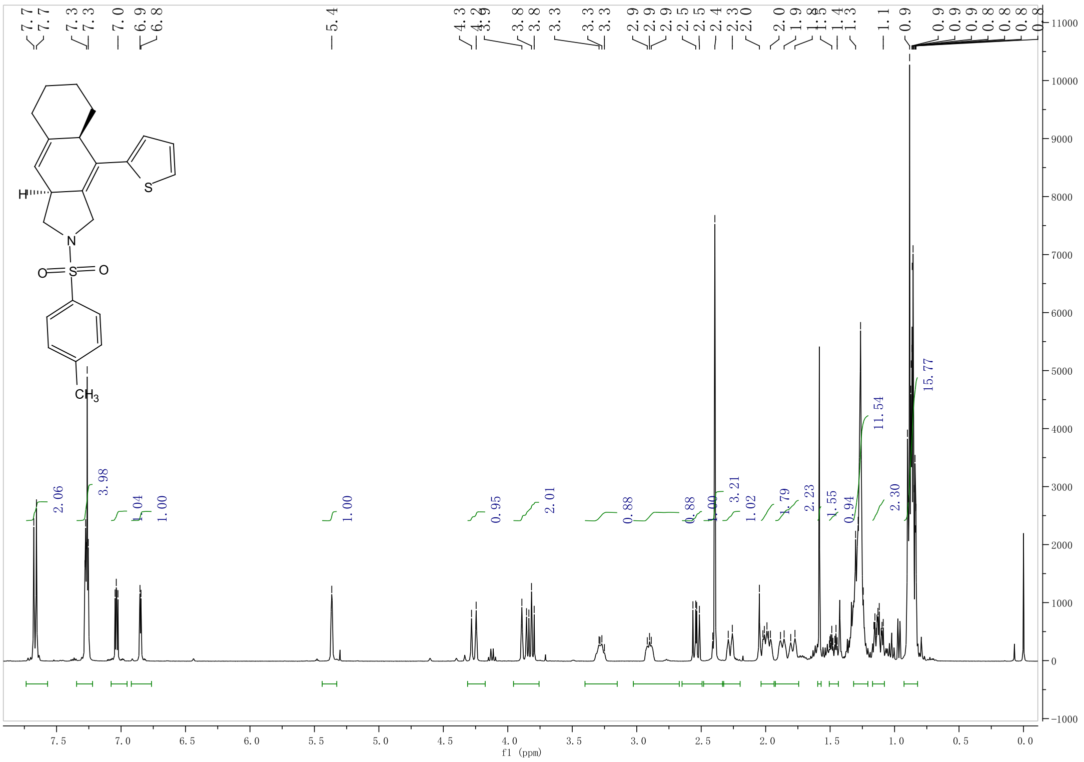

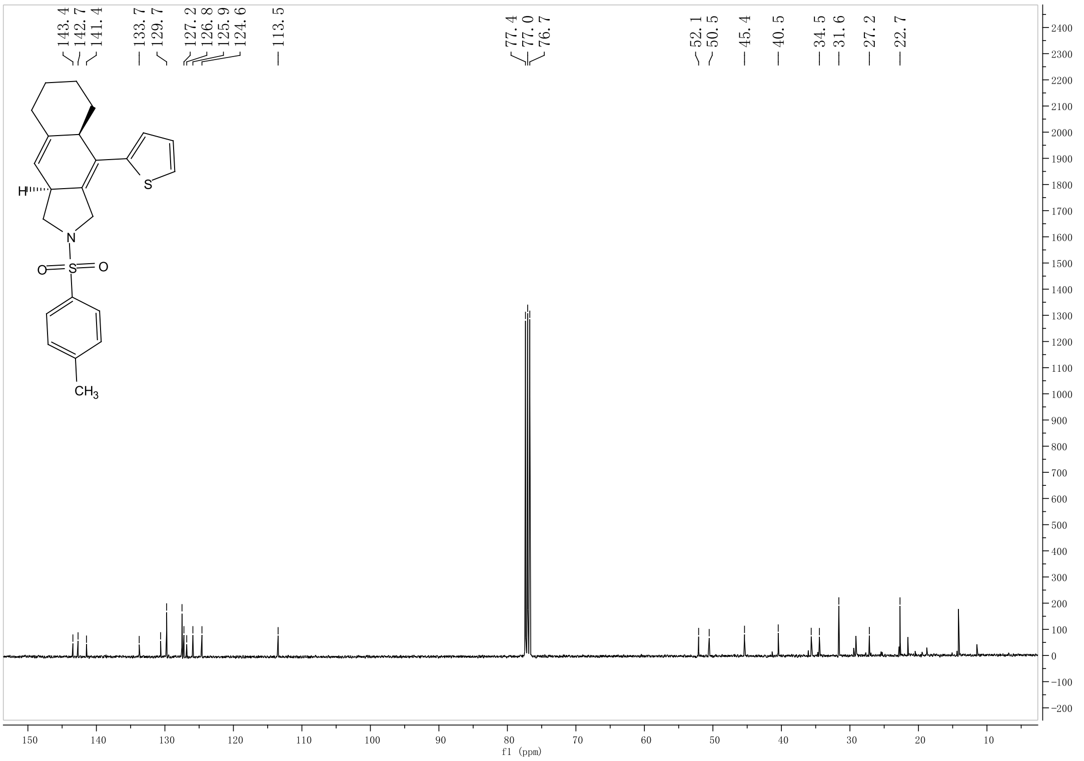

Supplementary Figure 18. ^{1}H and ^{13}C-NMR of **3a**

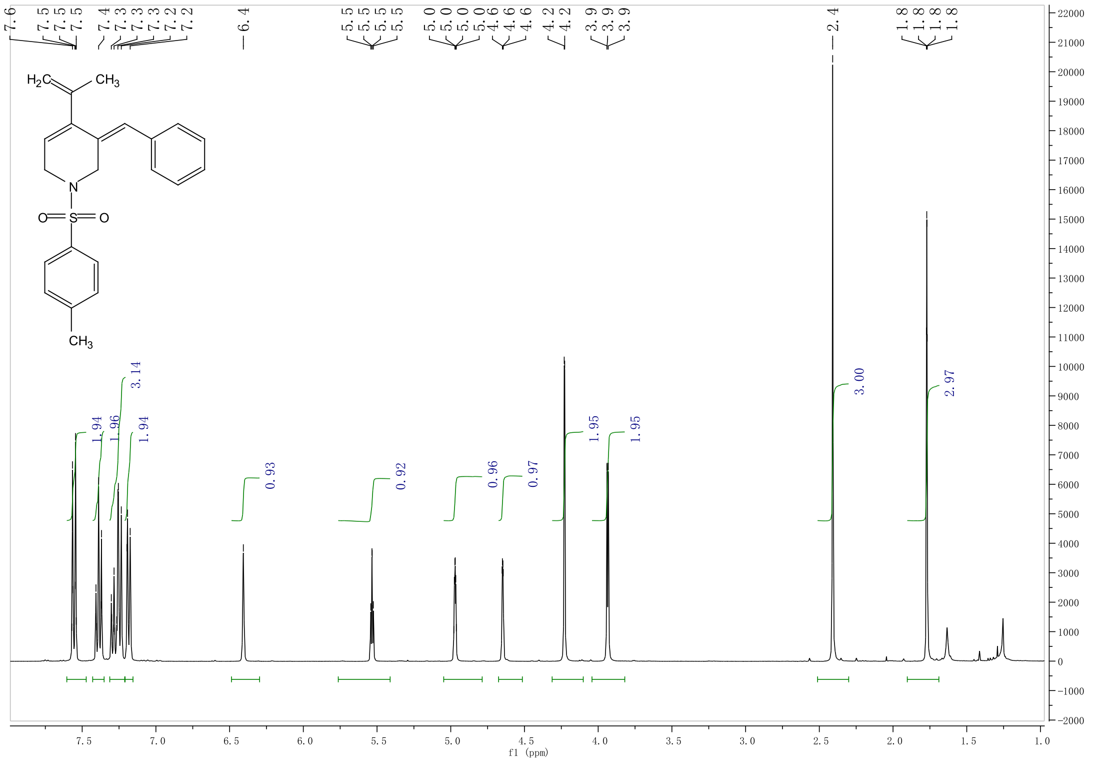

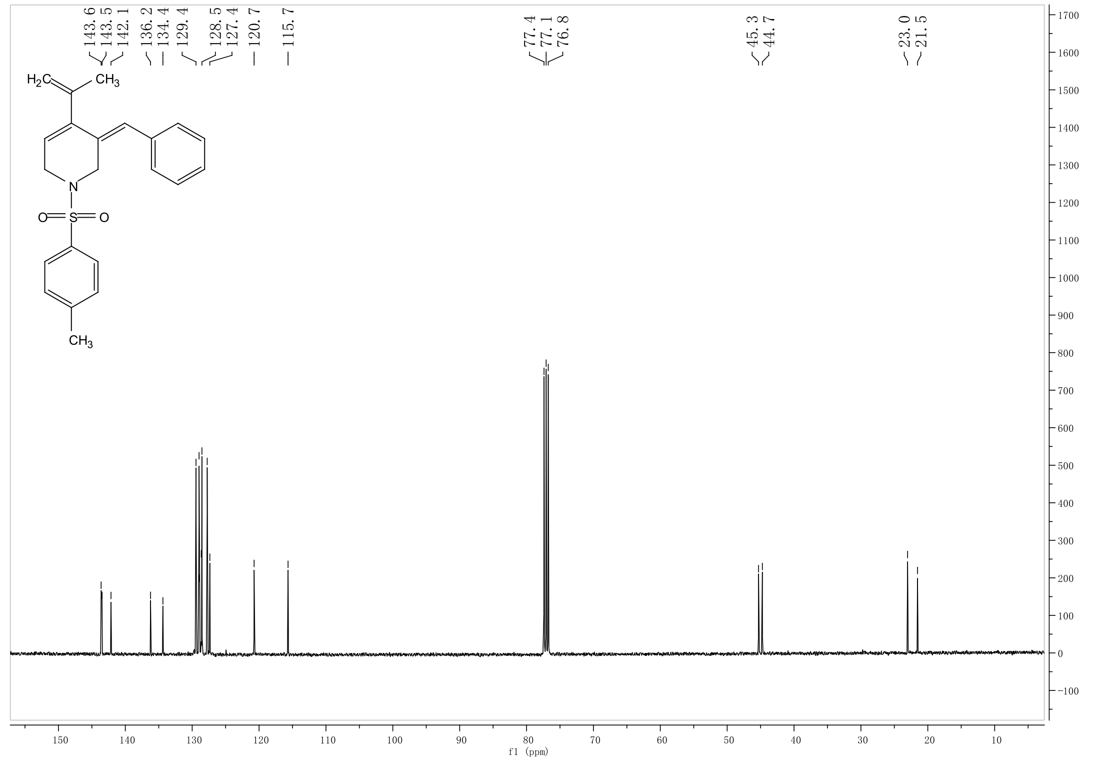

Supplementary Figure 19. ^{1}H and ^{13}C-NMR of **[D$_6$]-3a**

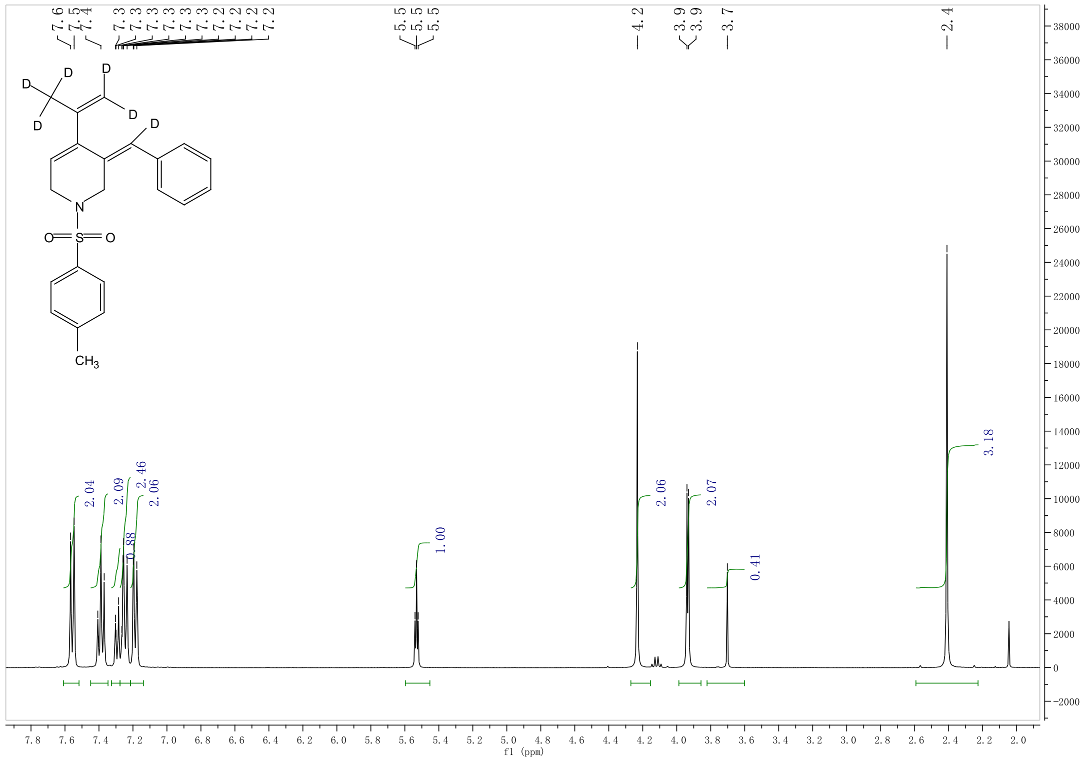

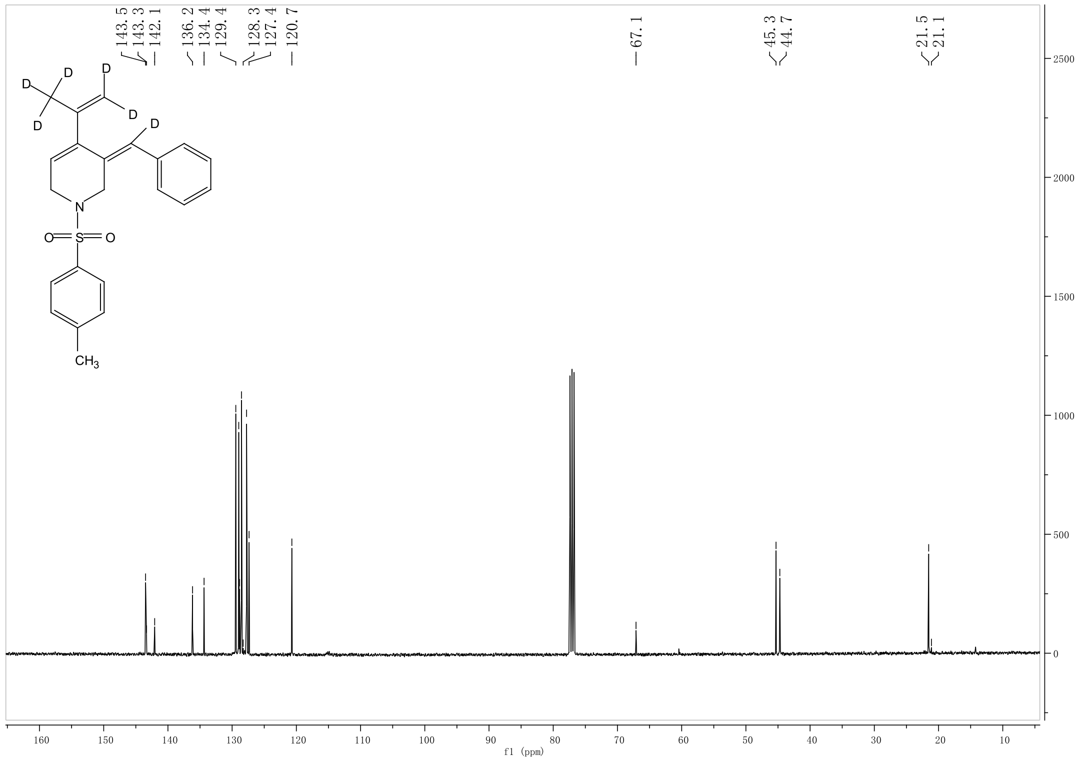

Supplementary Figure 20. ^{1}H and ^{13}C-NMR of **3b**

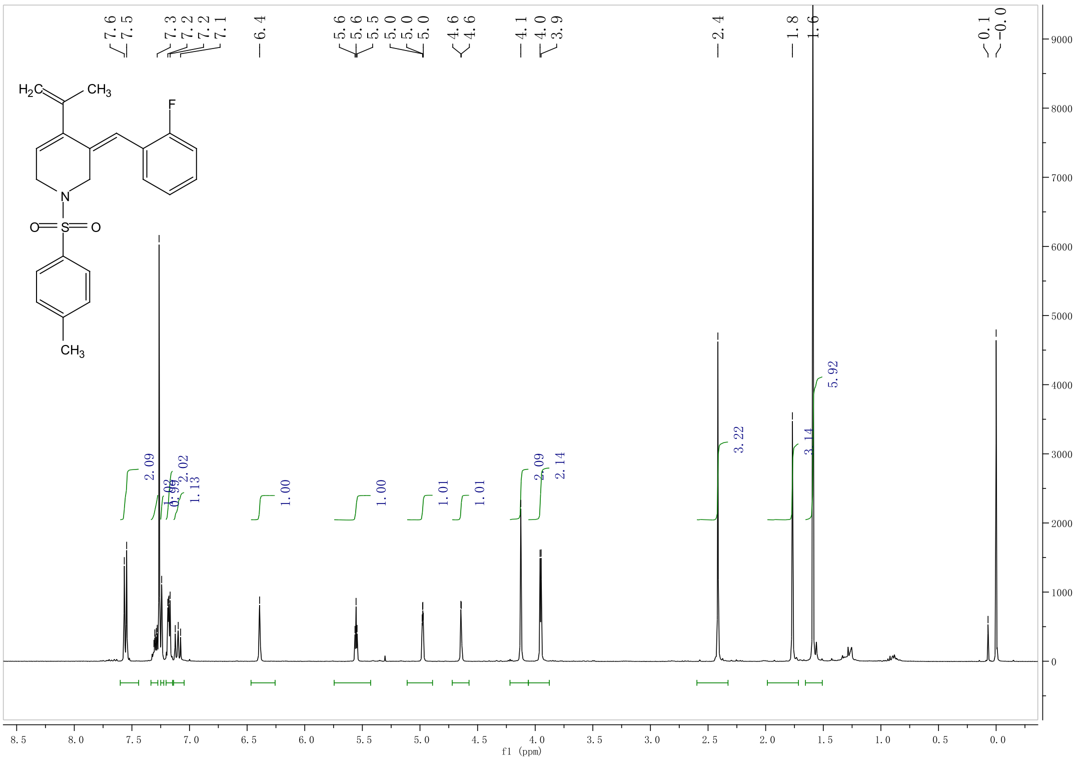

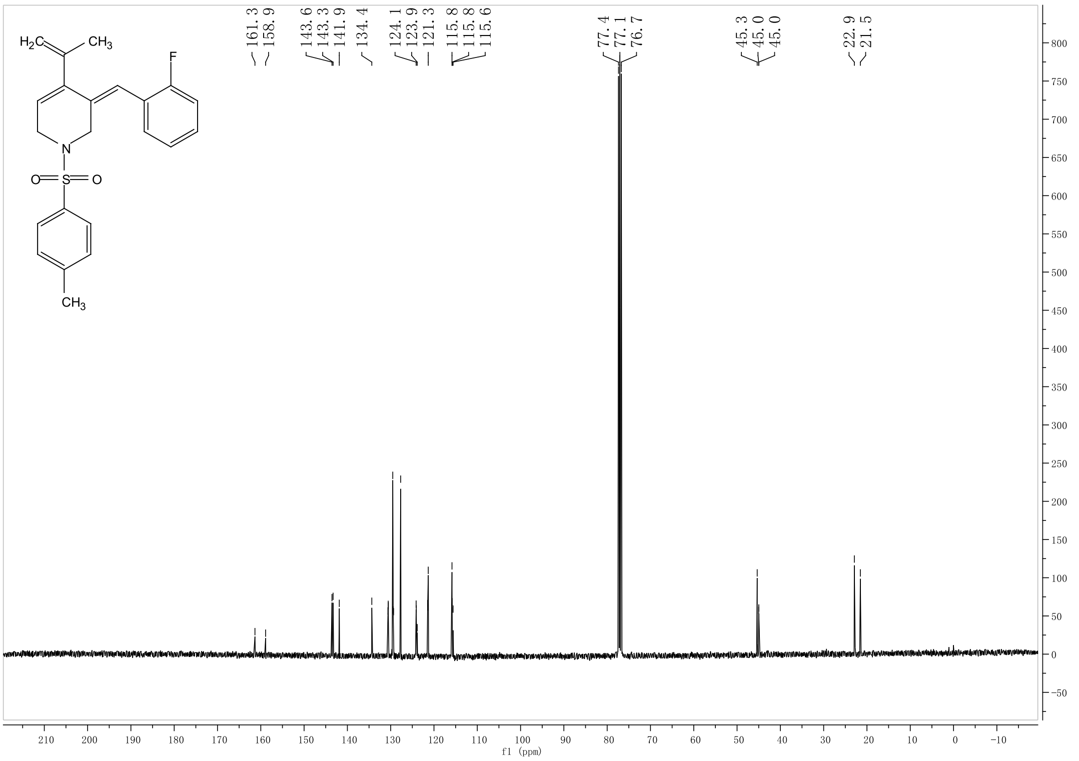

Supplementary Figure 21. ^{1}H and ^{13}C-NMR of **3c**

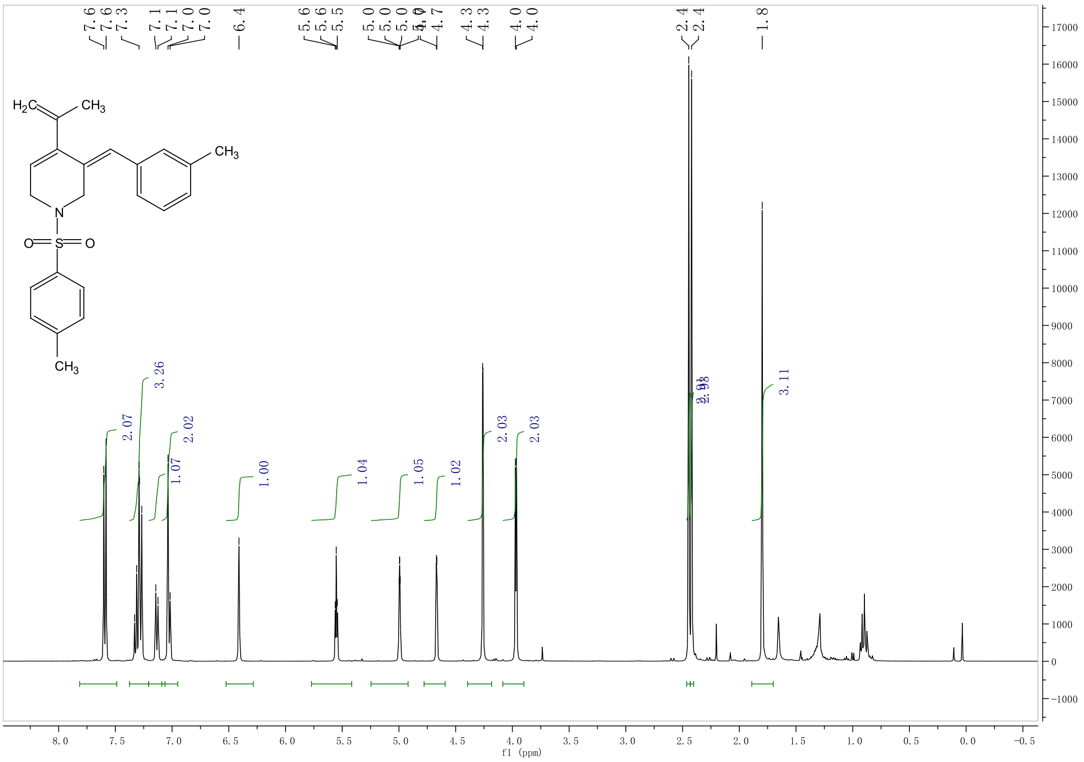

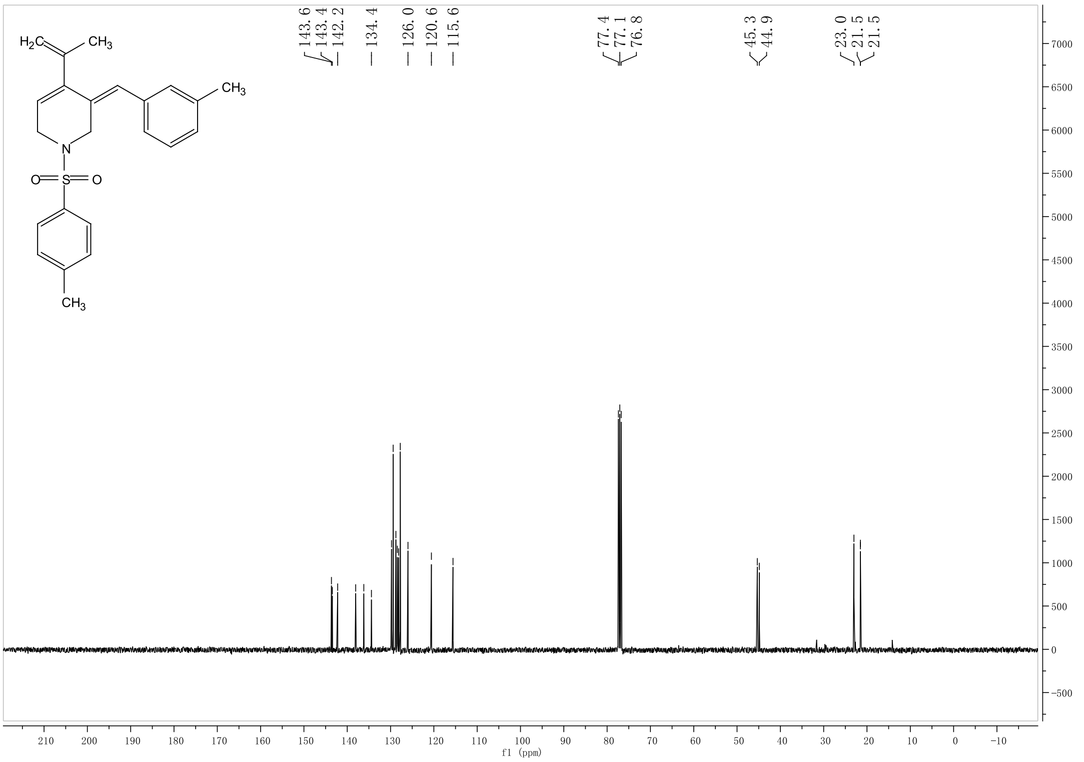

Supplementary Figure 22. ^{1}H and ^{13}C-NMR of **3d**

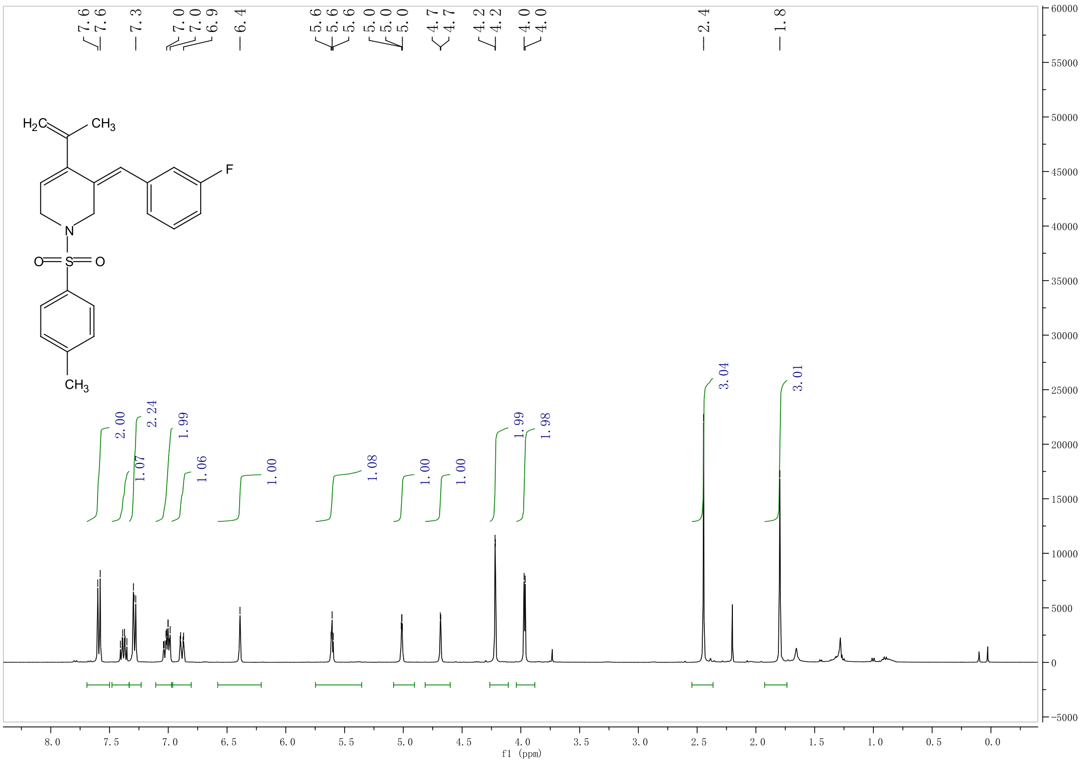

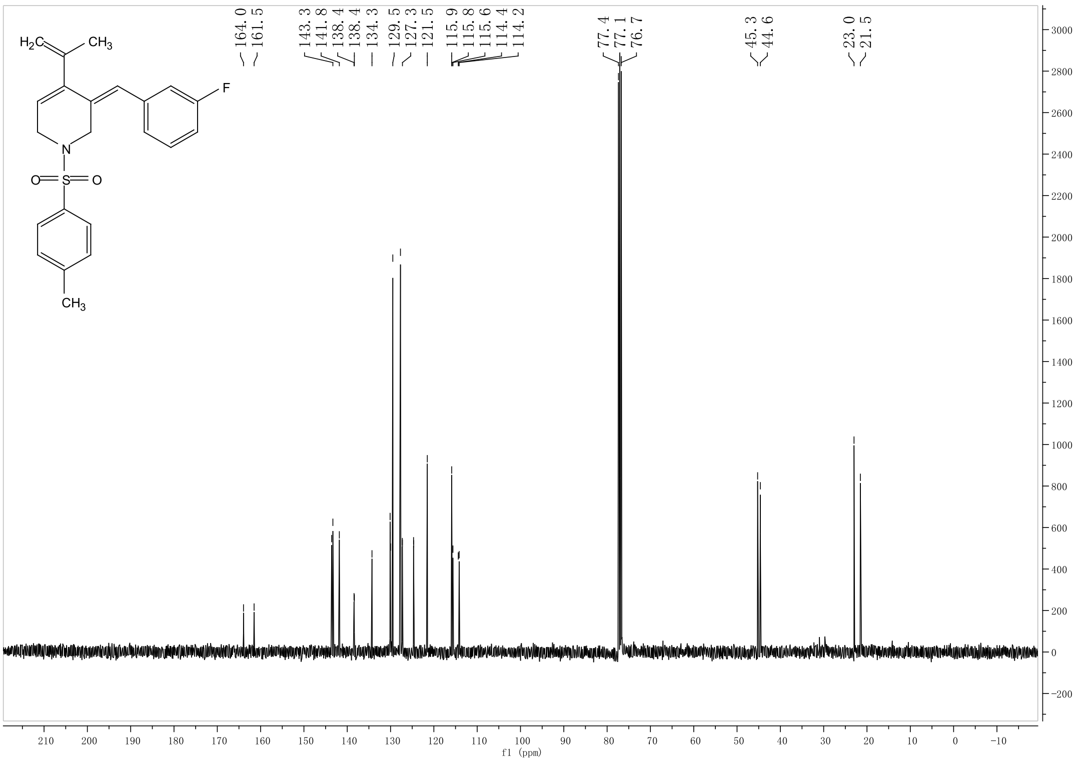

Supplementary Figure 23. ^{1}H and ^{13}C-NMR of **3e**

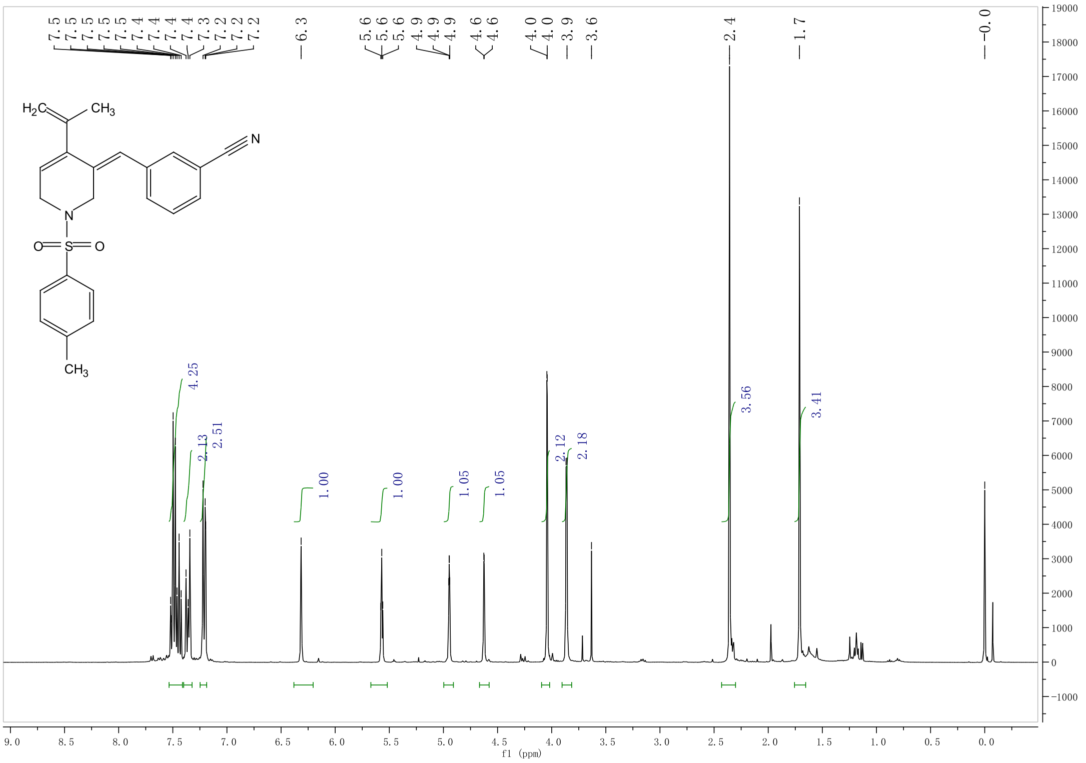

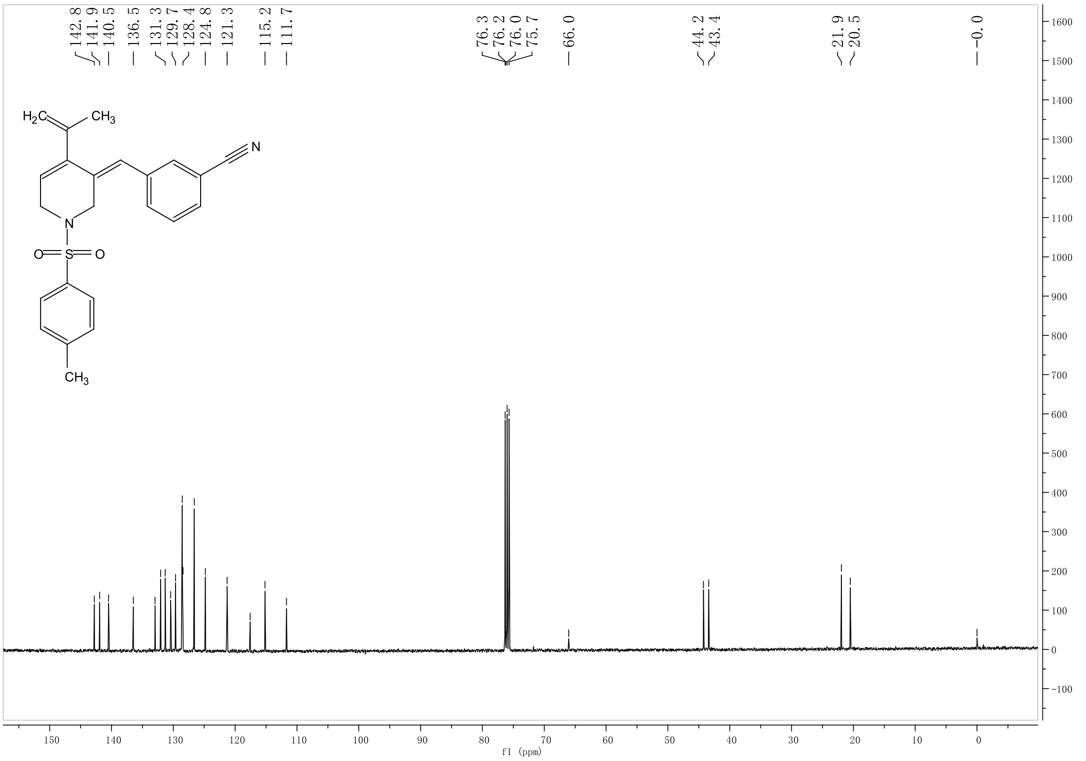

Supplementary Figure 24. ^{1}H and ^{13}C-NMR of **3f**

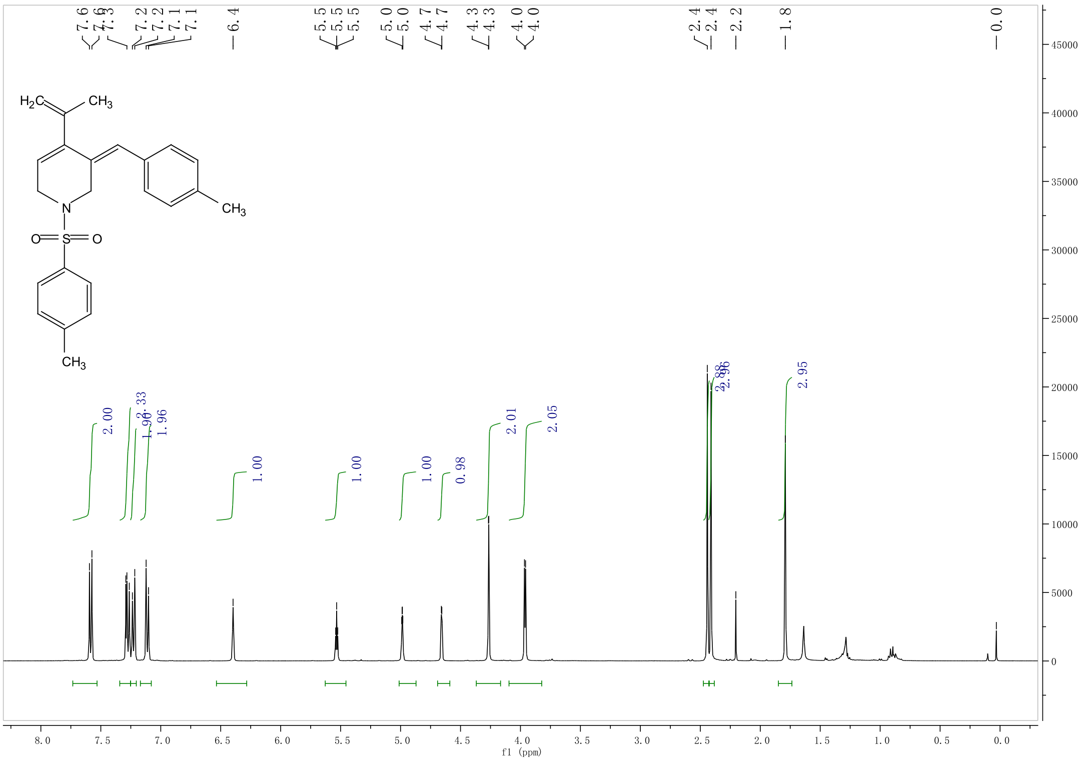

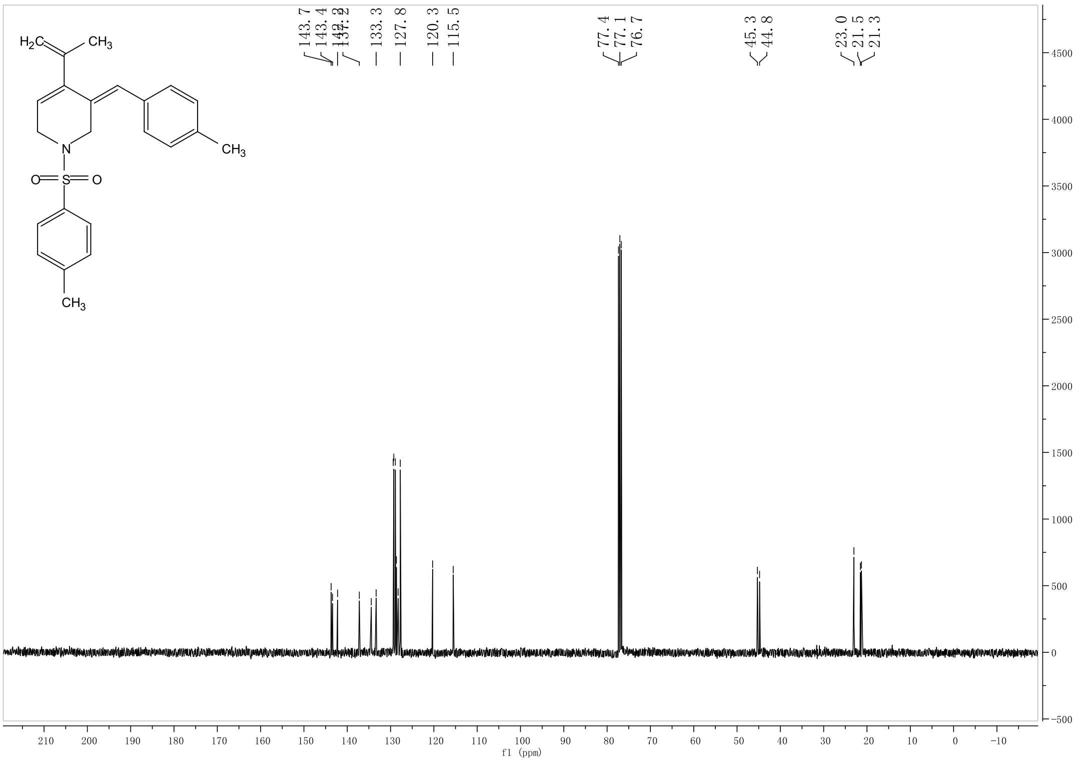

Supplementary Figure 25. ^{1}H and ^{13}C-NMR of **3g**

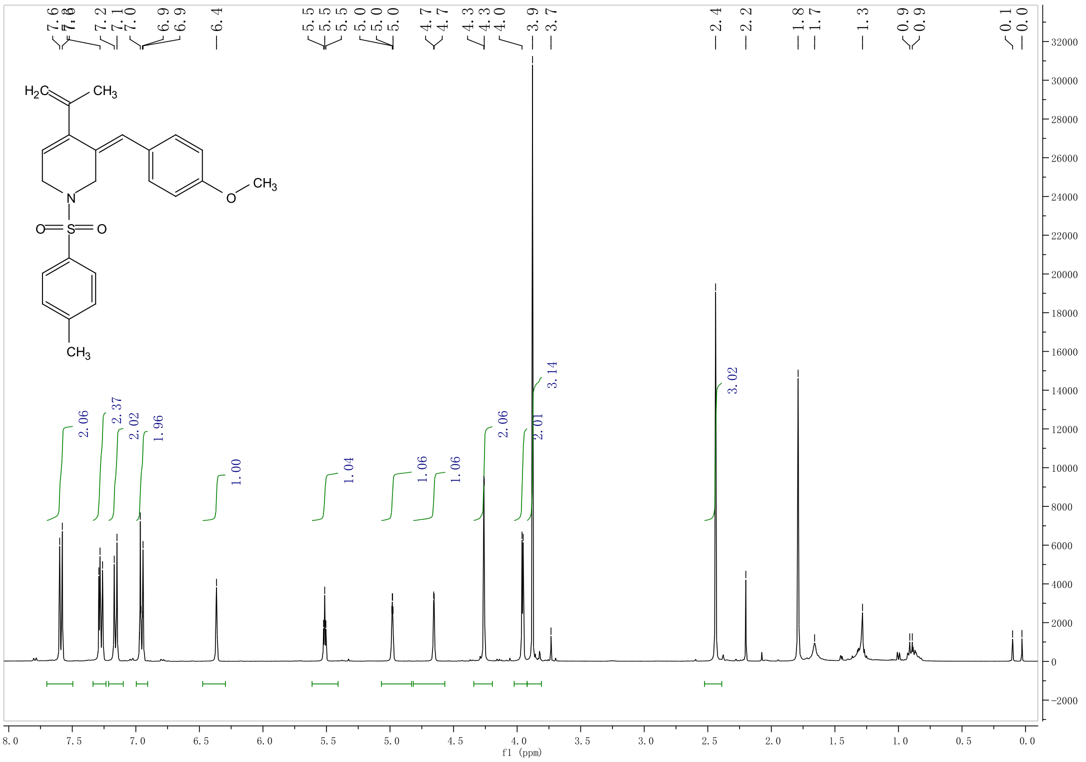

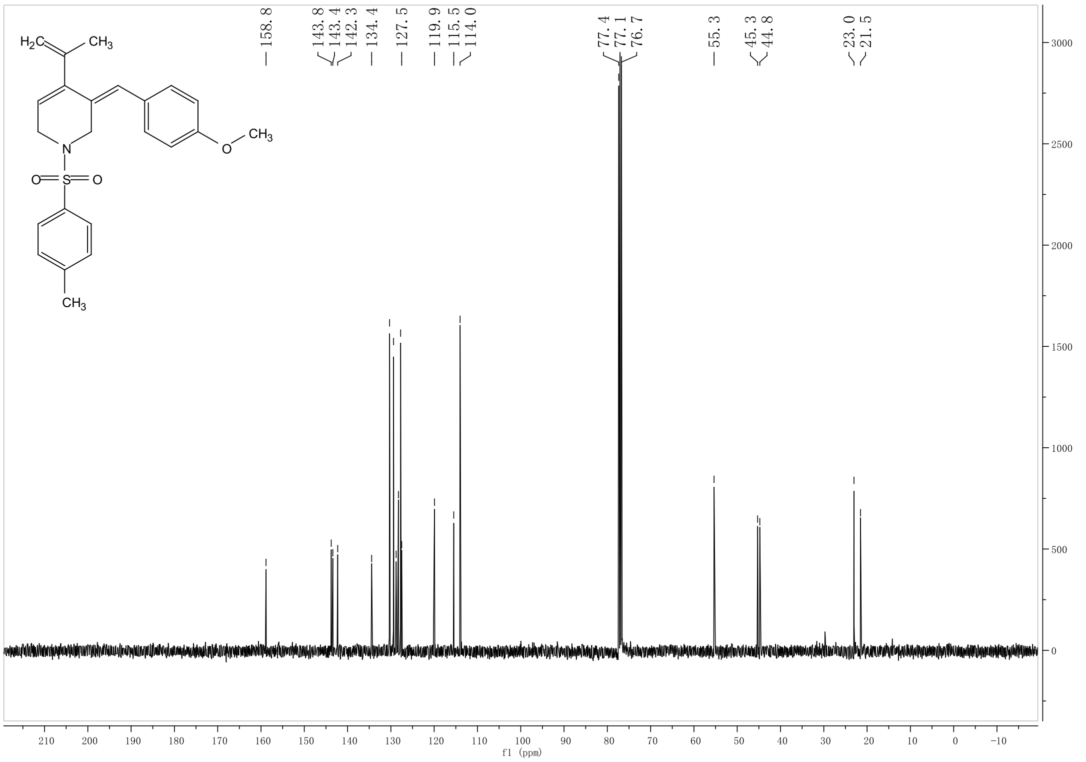

Supplementary Figure 26. ^{1}H and ^{13}C-NMR of **3h**

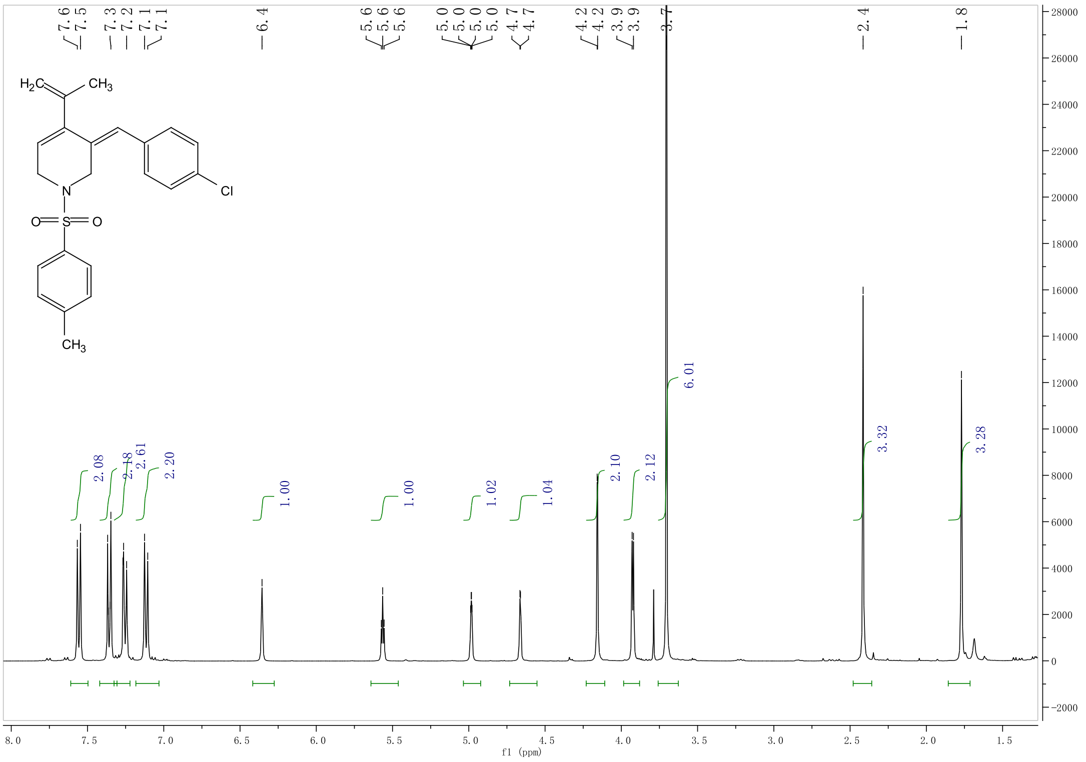

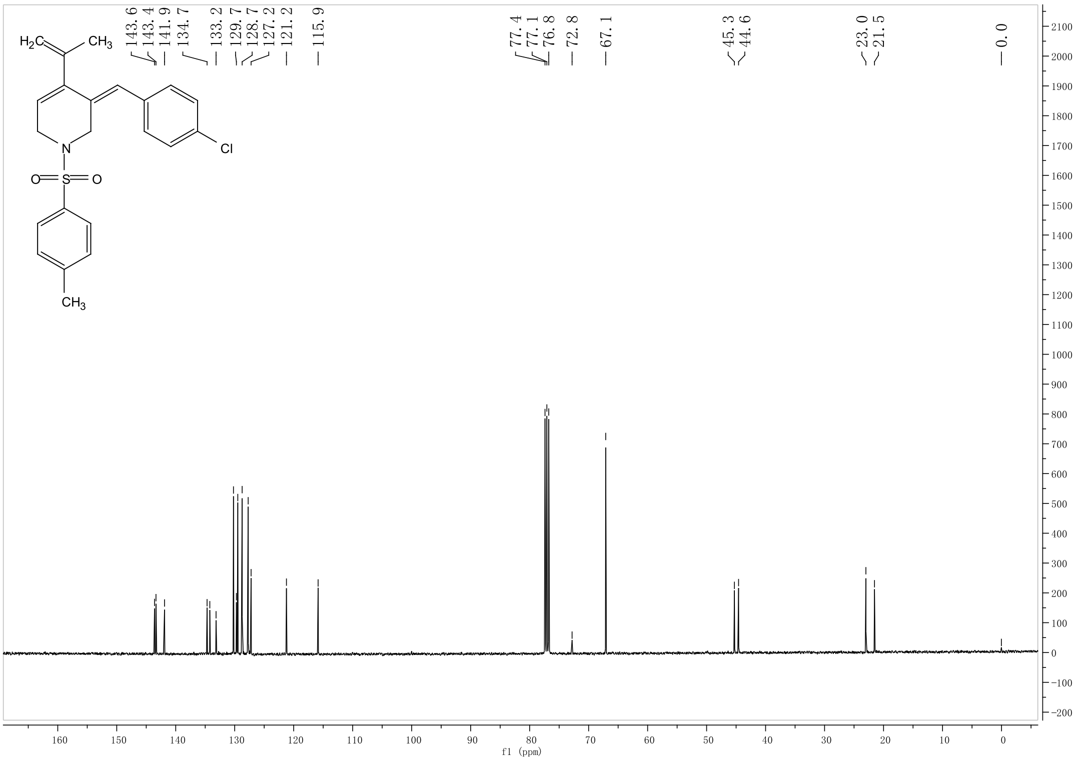

Supplementary Figure 27. ^{1}H and ^{13}C-NMR of **3i**

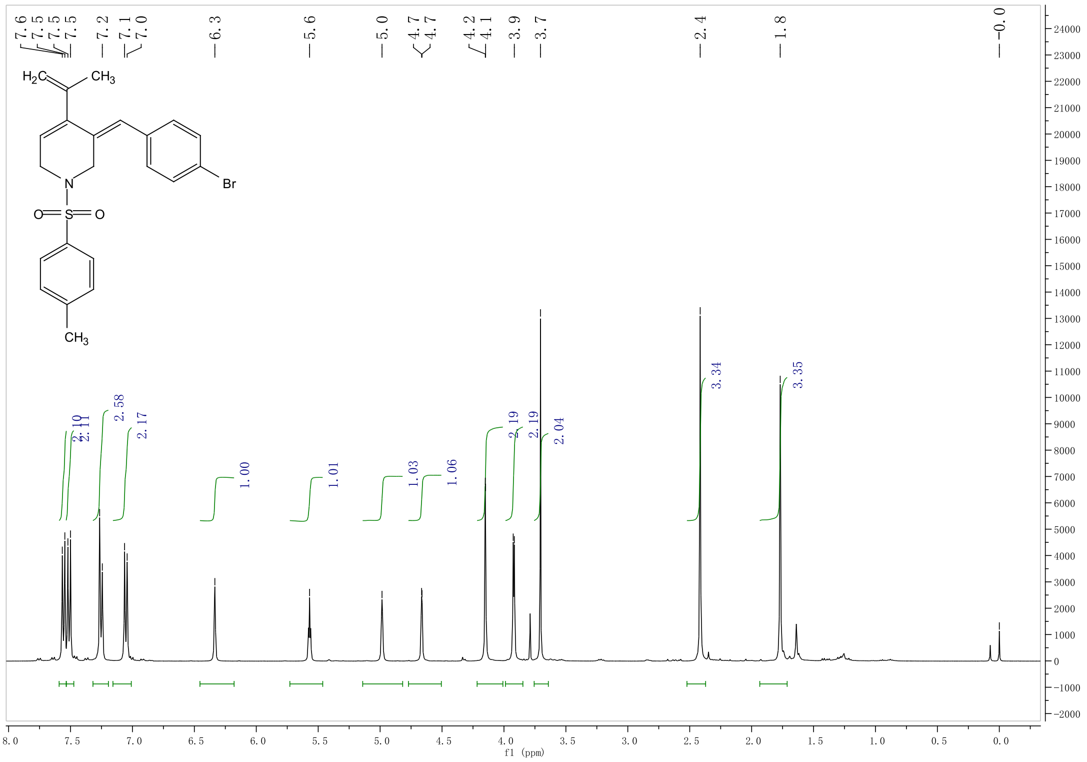

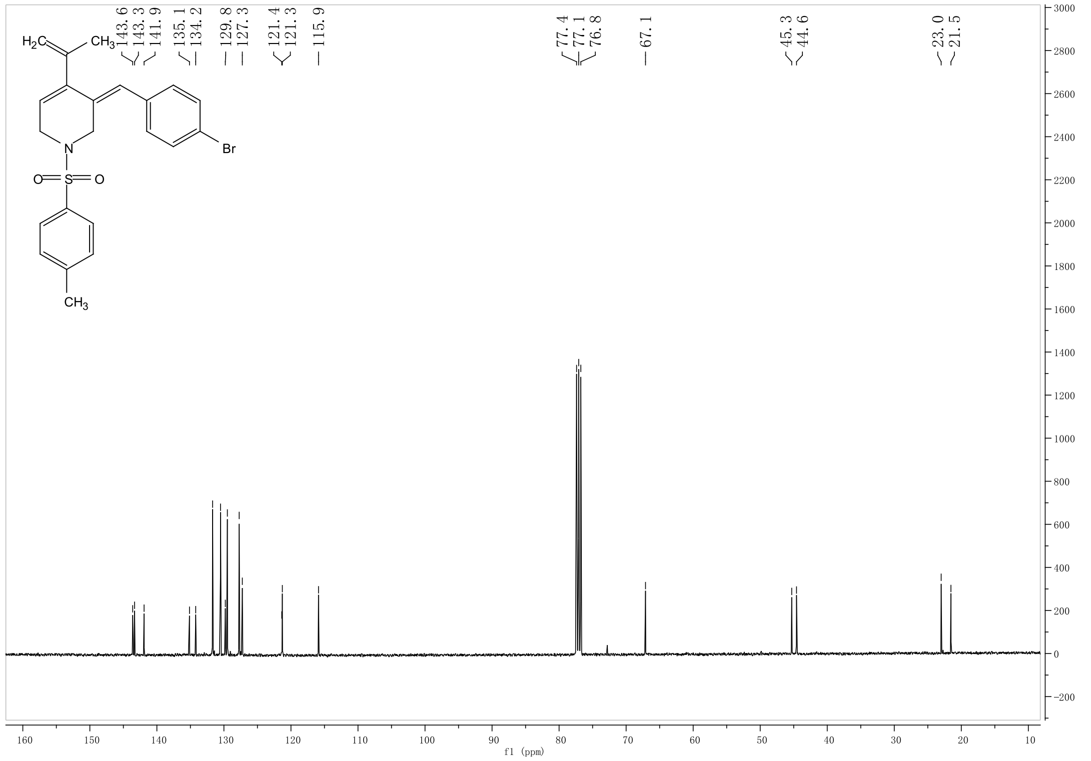

Supplementary Figure 28. ^{1}H and ^{13}C-NMR of **3j**

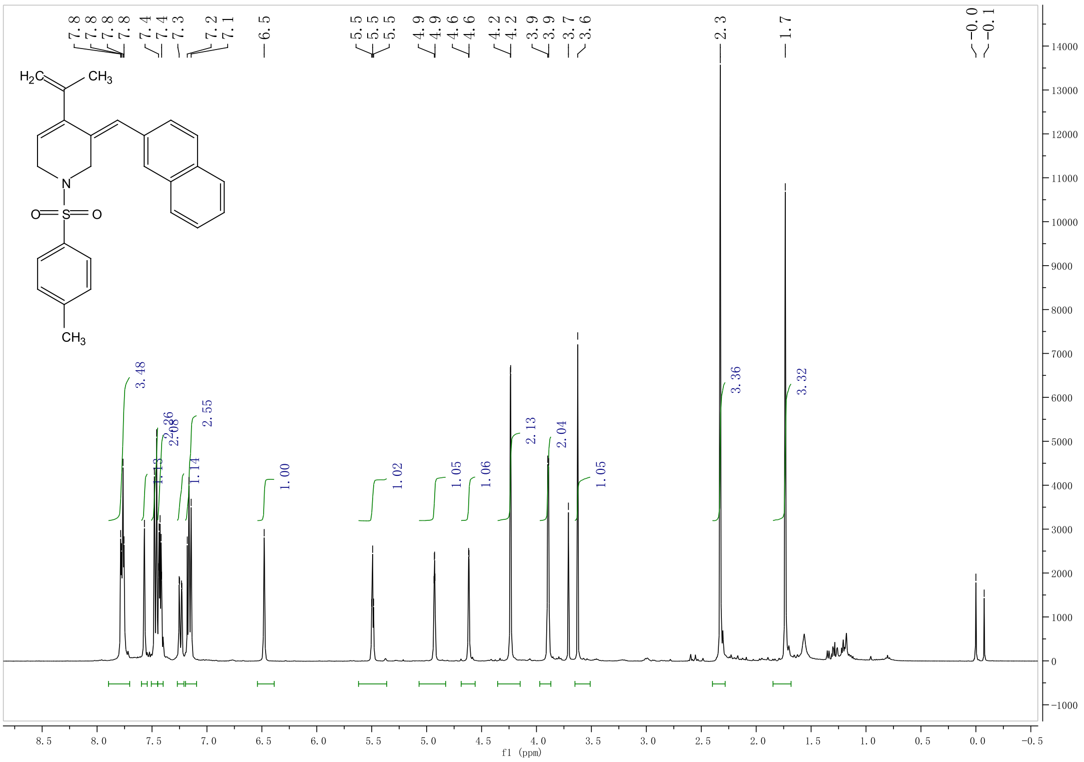

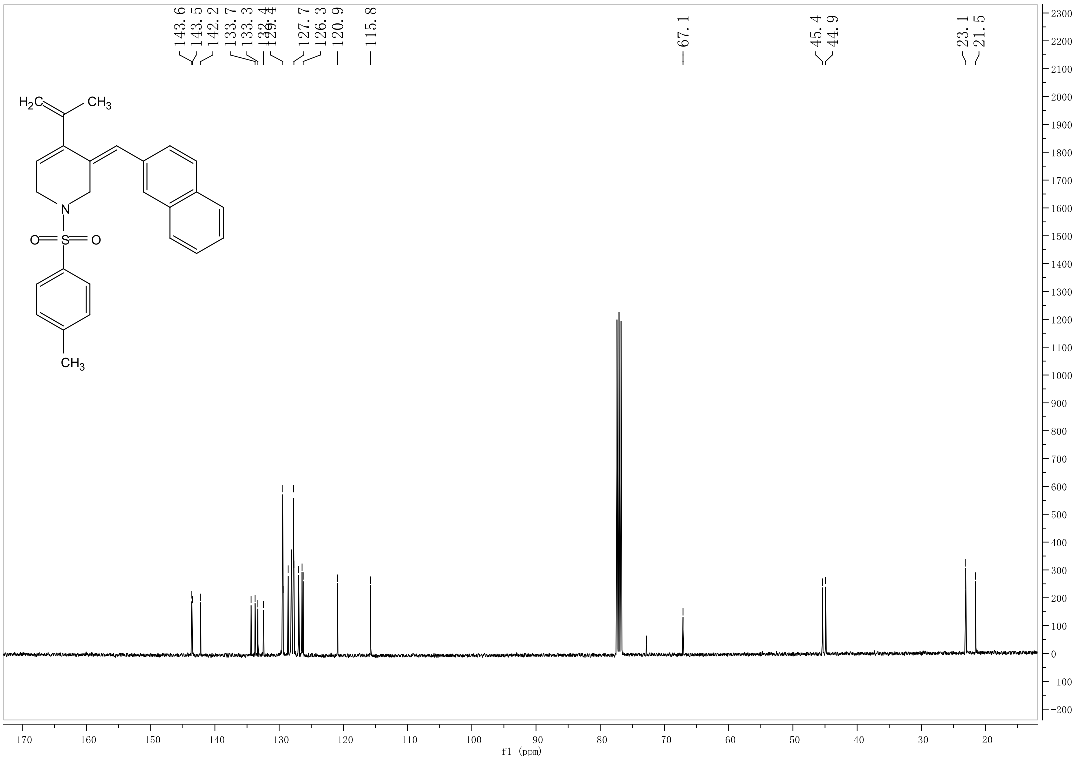

Supplementary Figure 29. ^{1}H and ^{13}C-NMR of **3k**

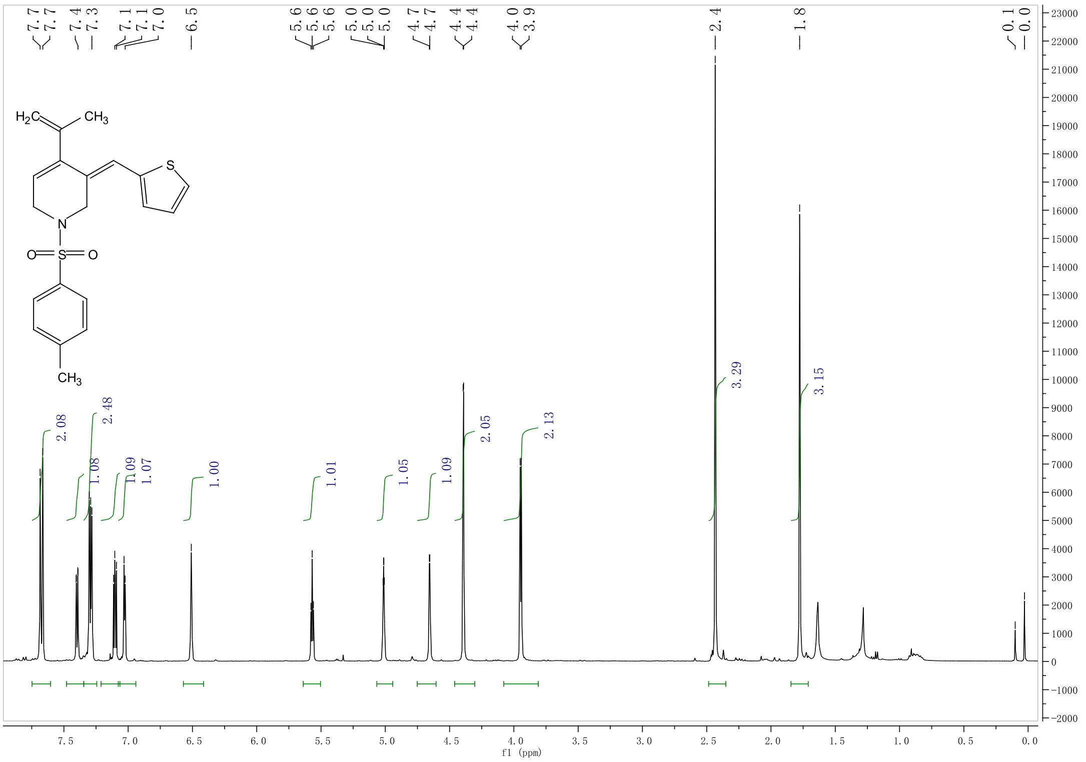

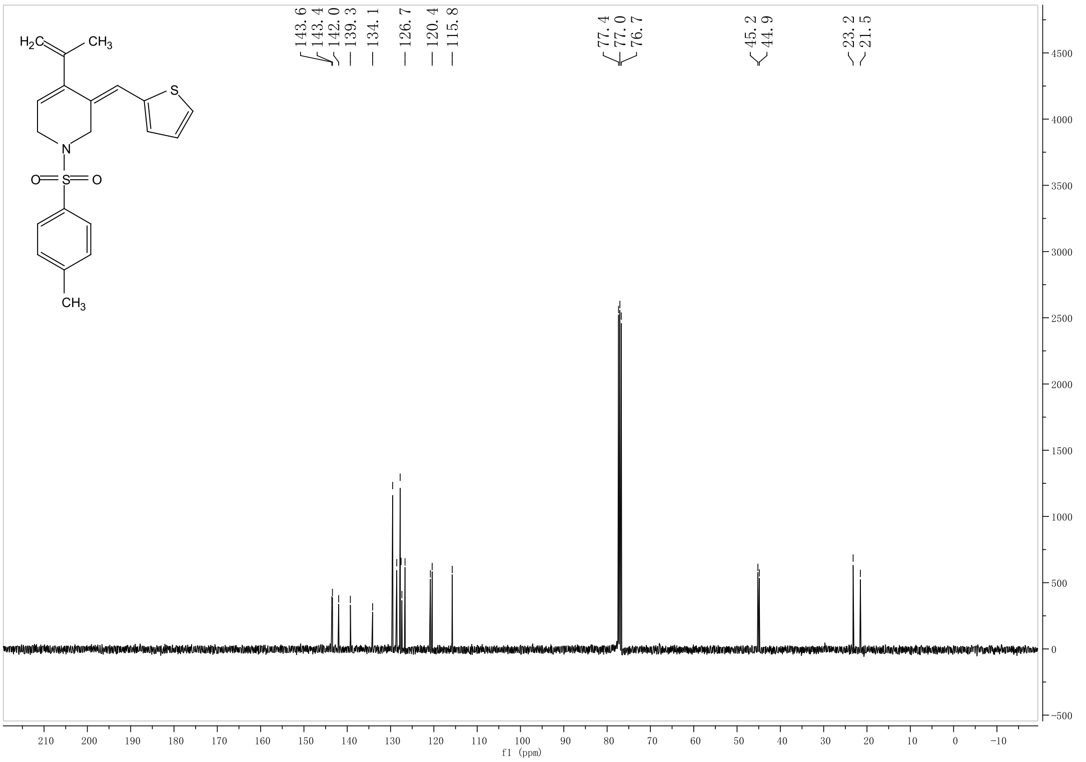

Supplementary Figure 30. ^{1}H and ^{13}C-NMR of **3l**

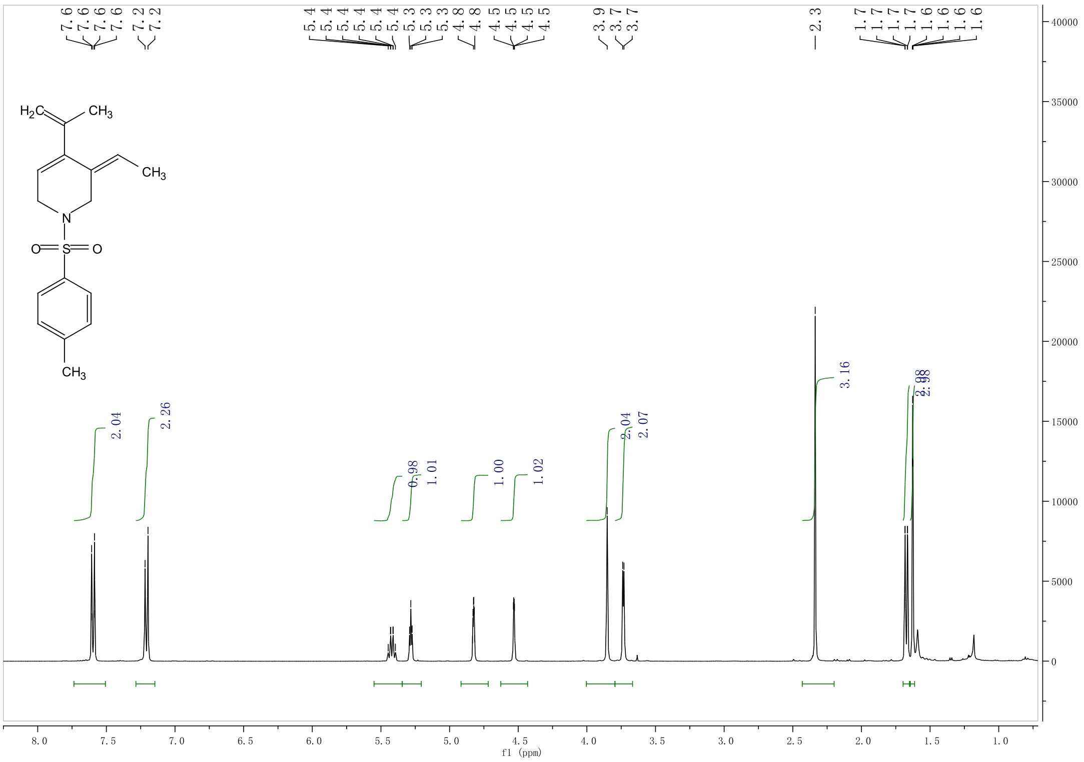

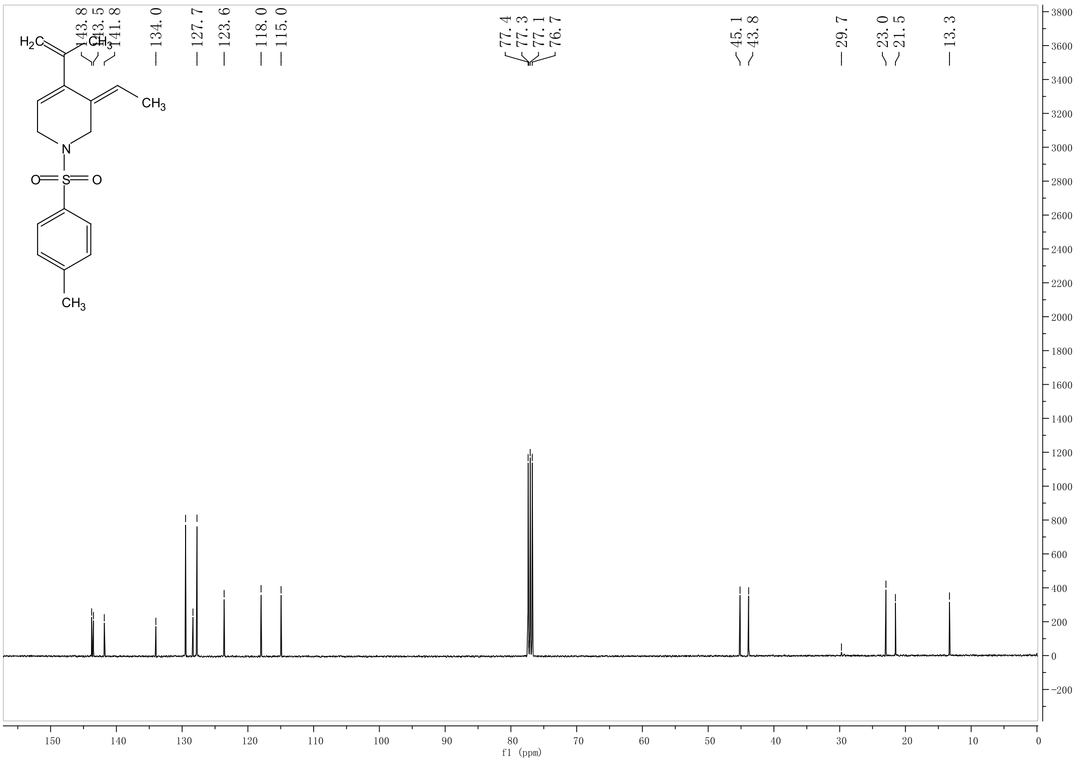

Supplementary Figure 31. ^{1}H and ^{13}C-NMR of **3m**

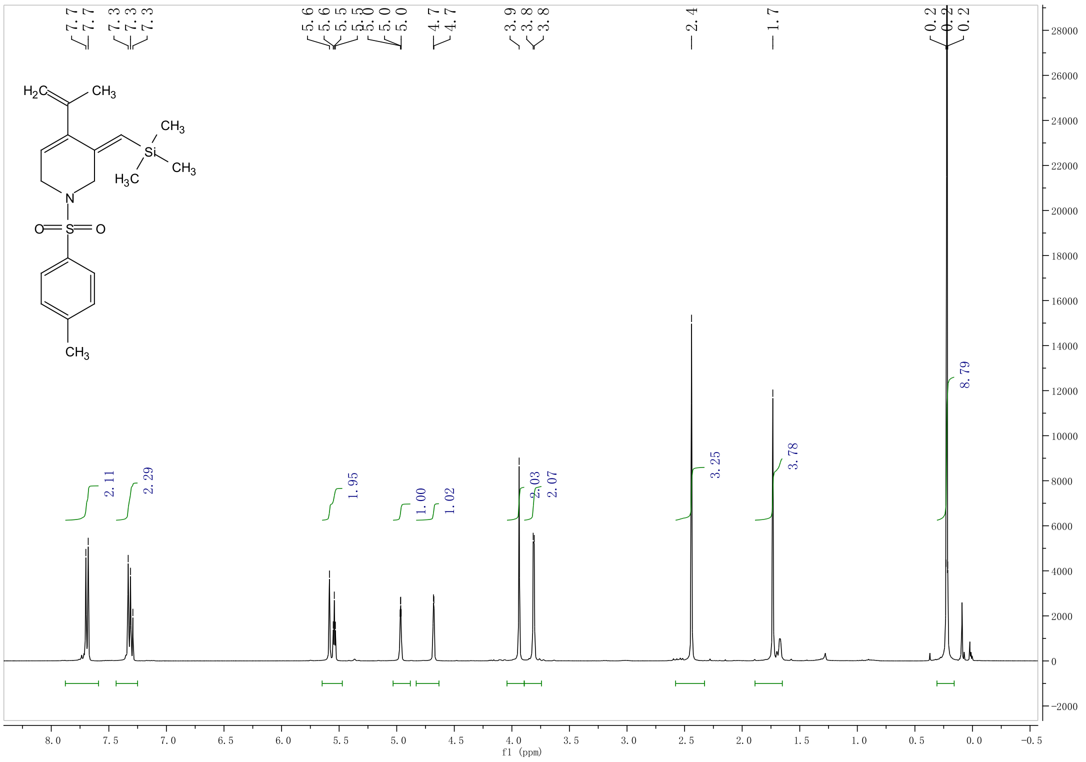

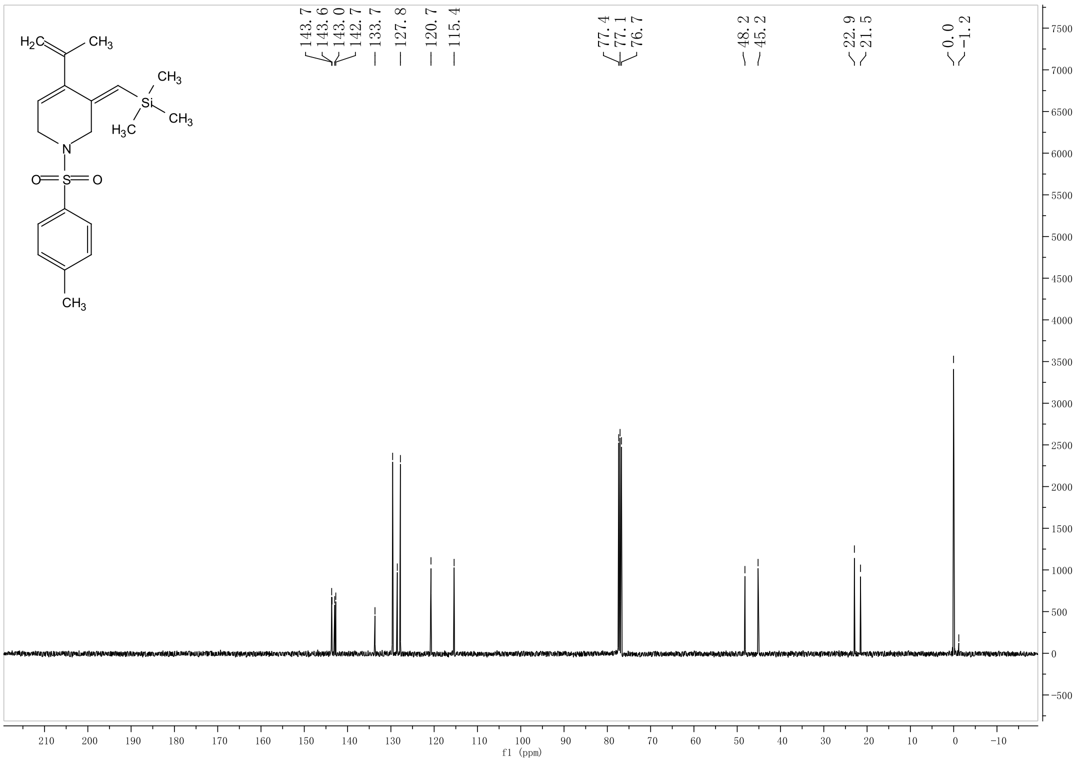

Supplementary Figure 32. ^{1}H and ^{13}C-NMR of **3n**

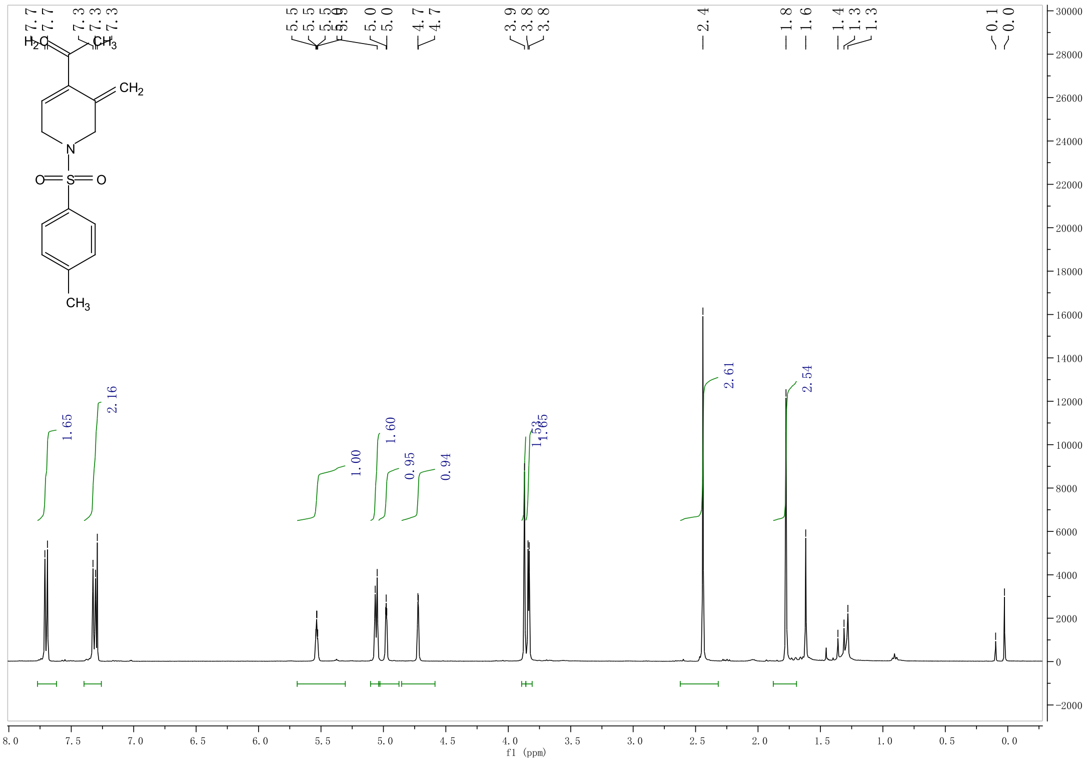

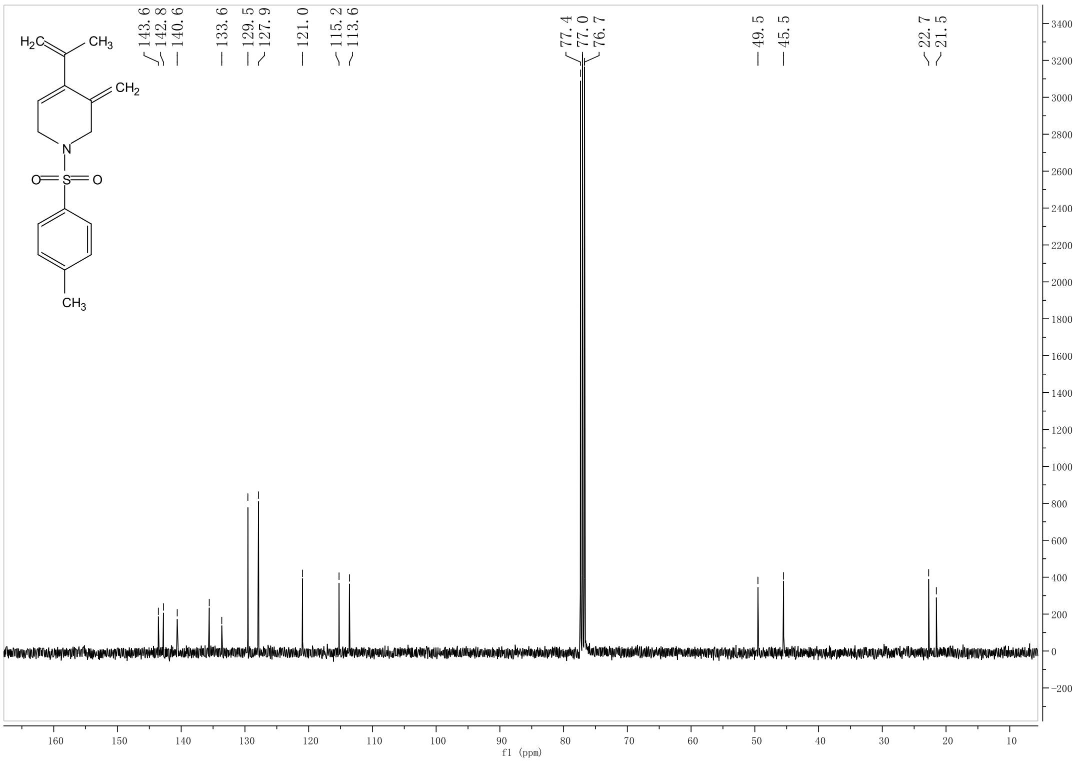

Supplementary Figure 33. ^{1}H and ^{13}C-NMR of **3o**

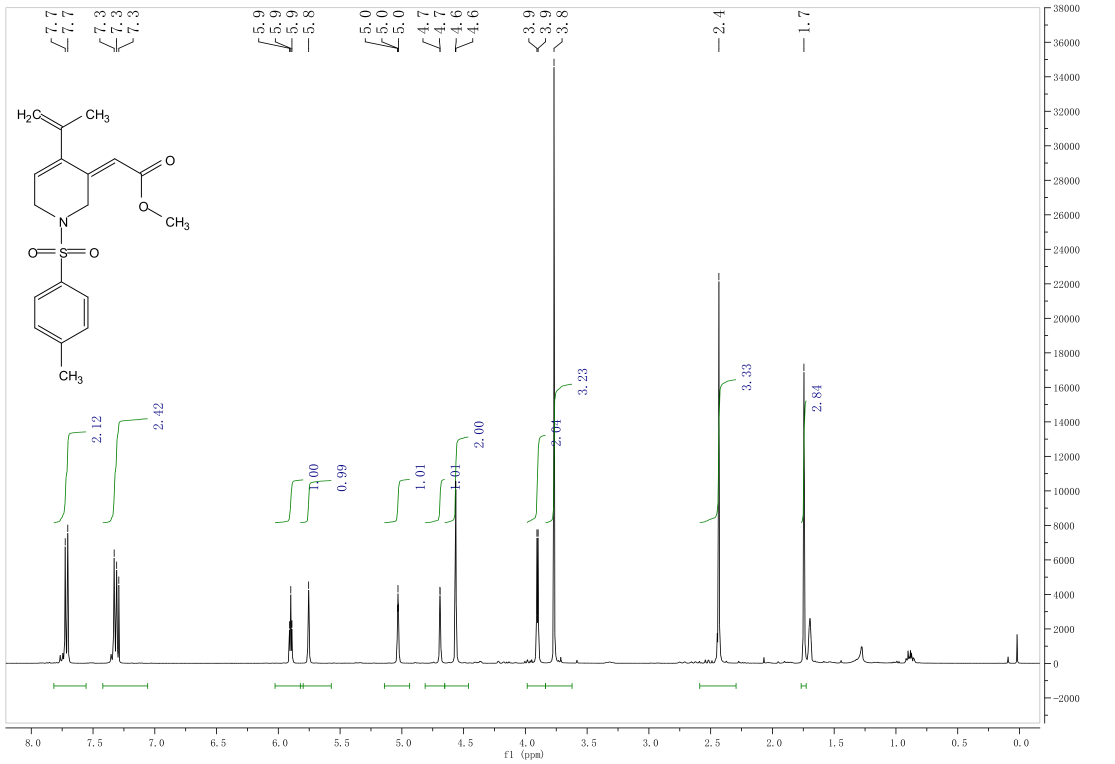

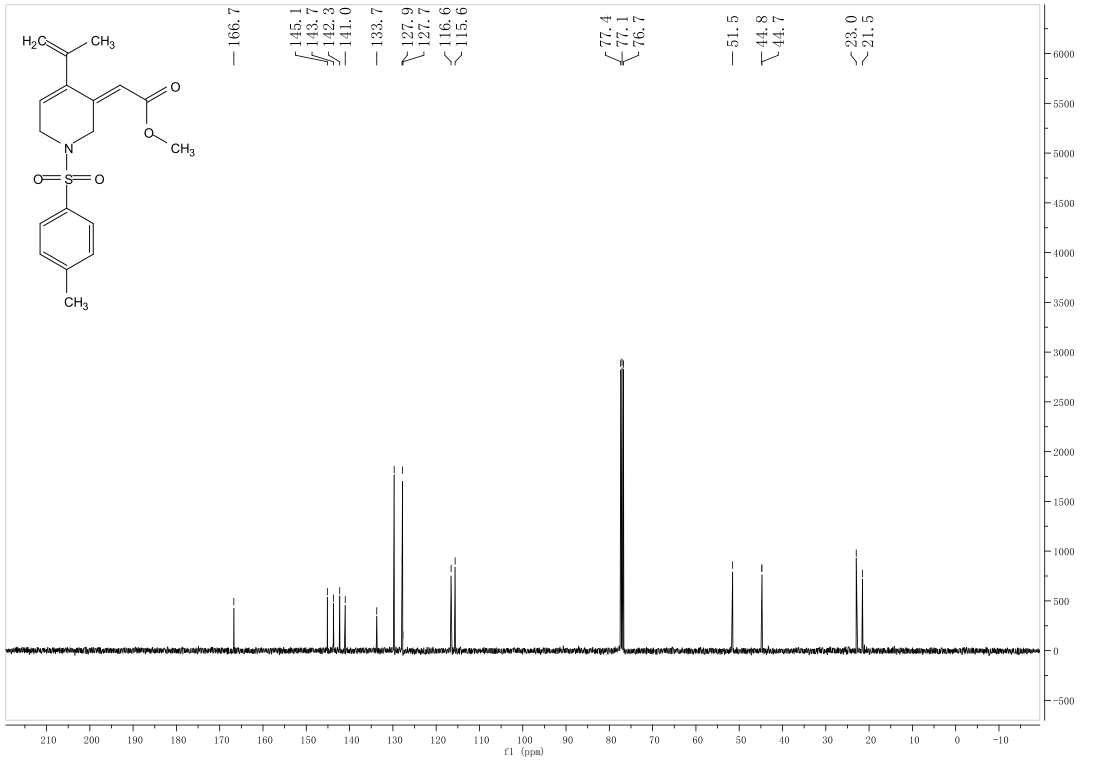

Supplementary Figure 34. ^{1}H and ^{13}C-NMR of **3p**

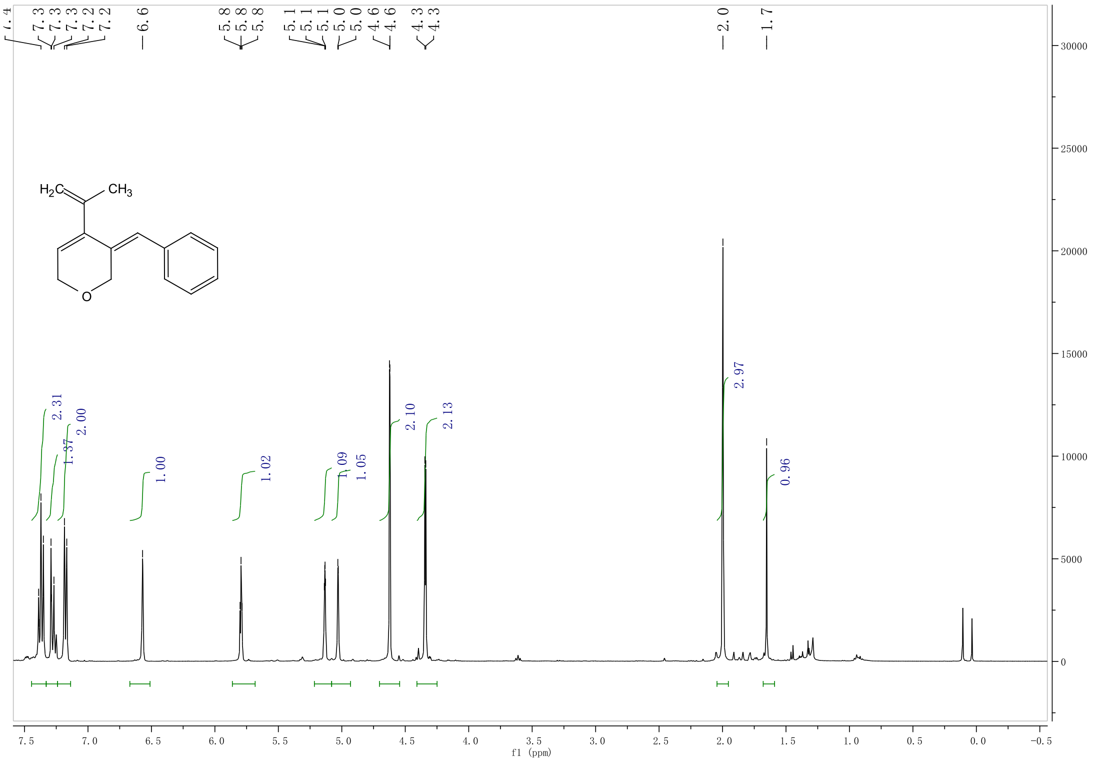

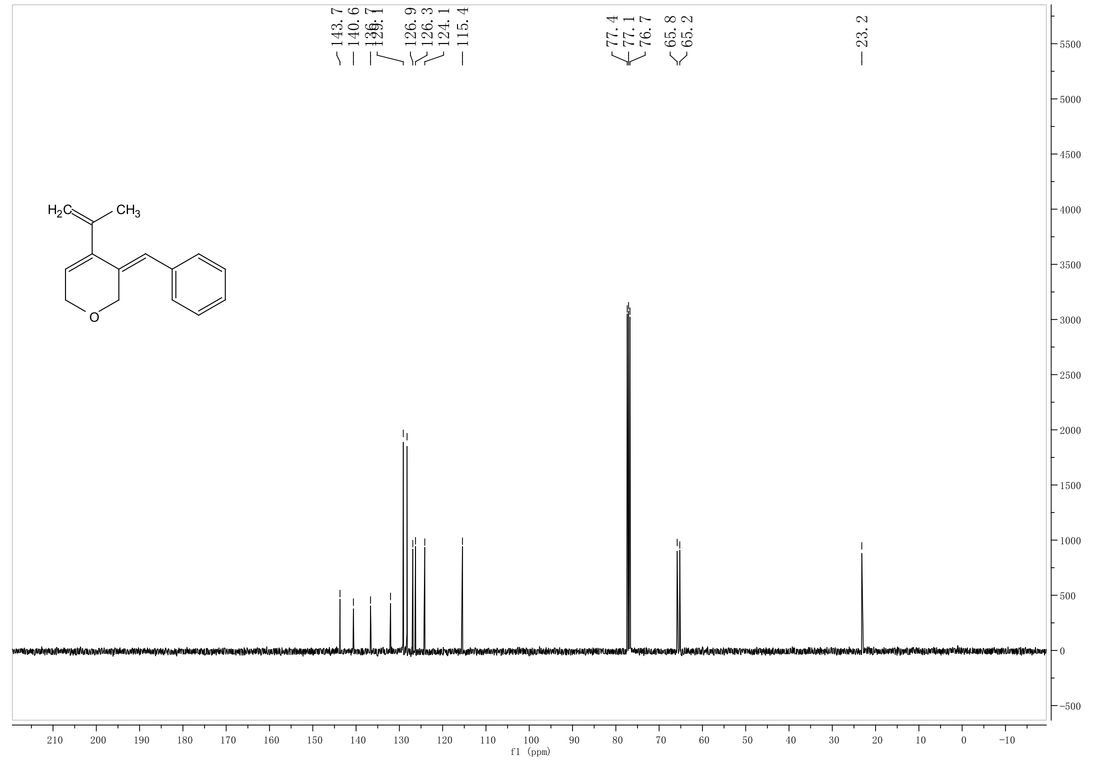

Supplementary Figure 35. ^{1}H and ^{13}C-NMR of **3q**

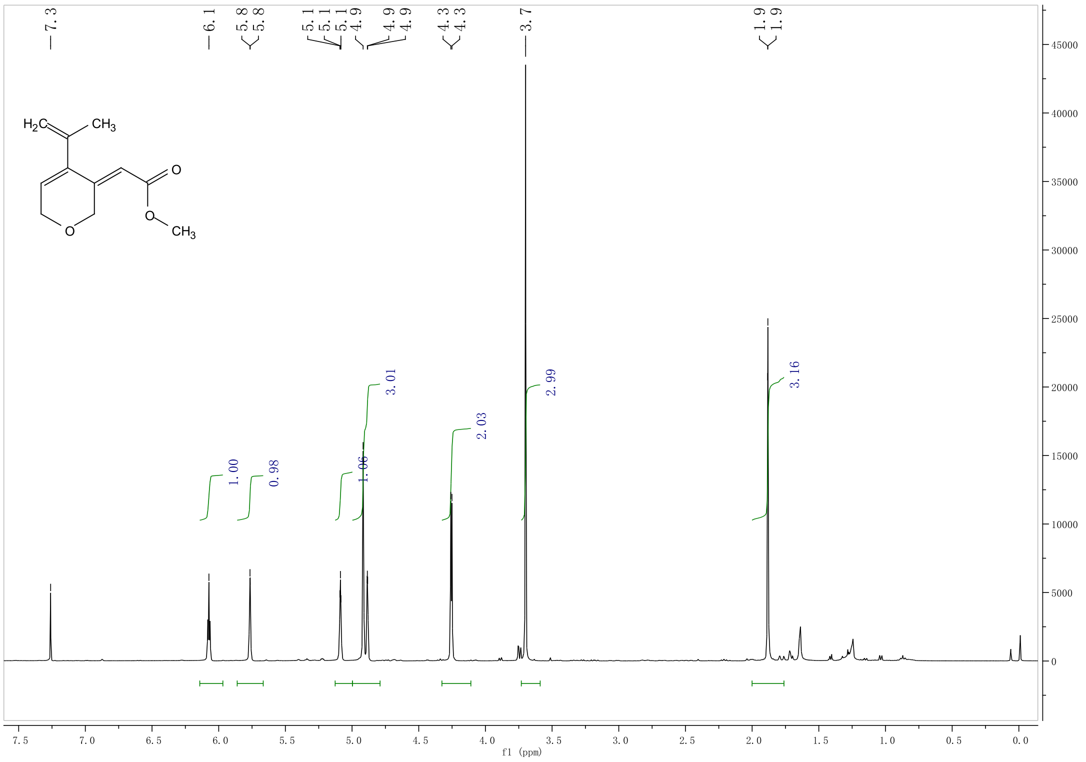

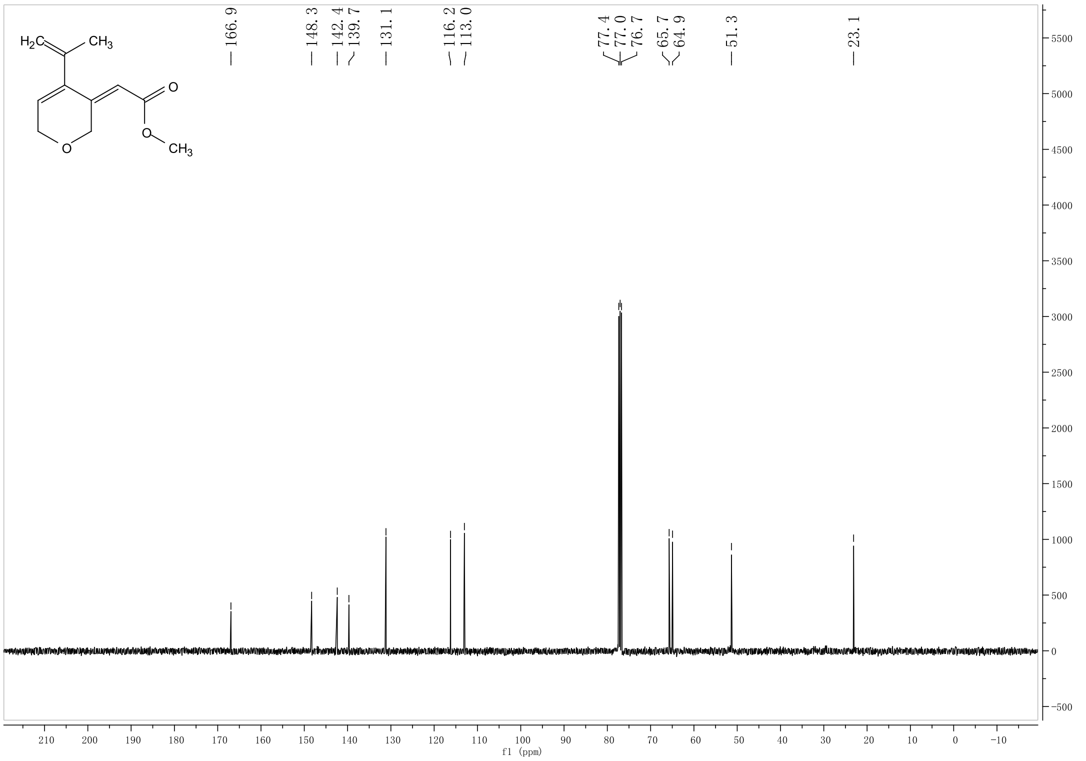

Supplementary Figure 36. ^{1}H and ^{13}C-NMR of **6a**

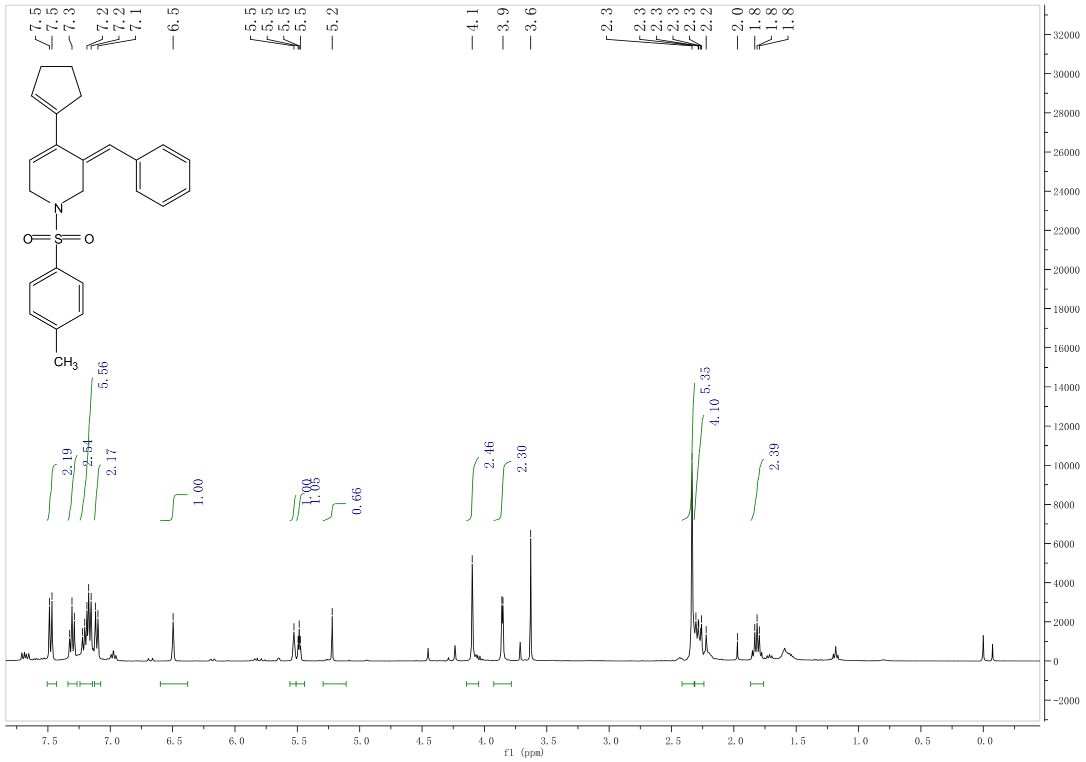

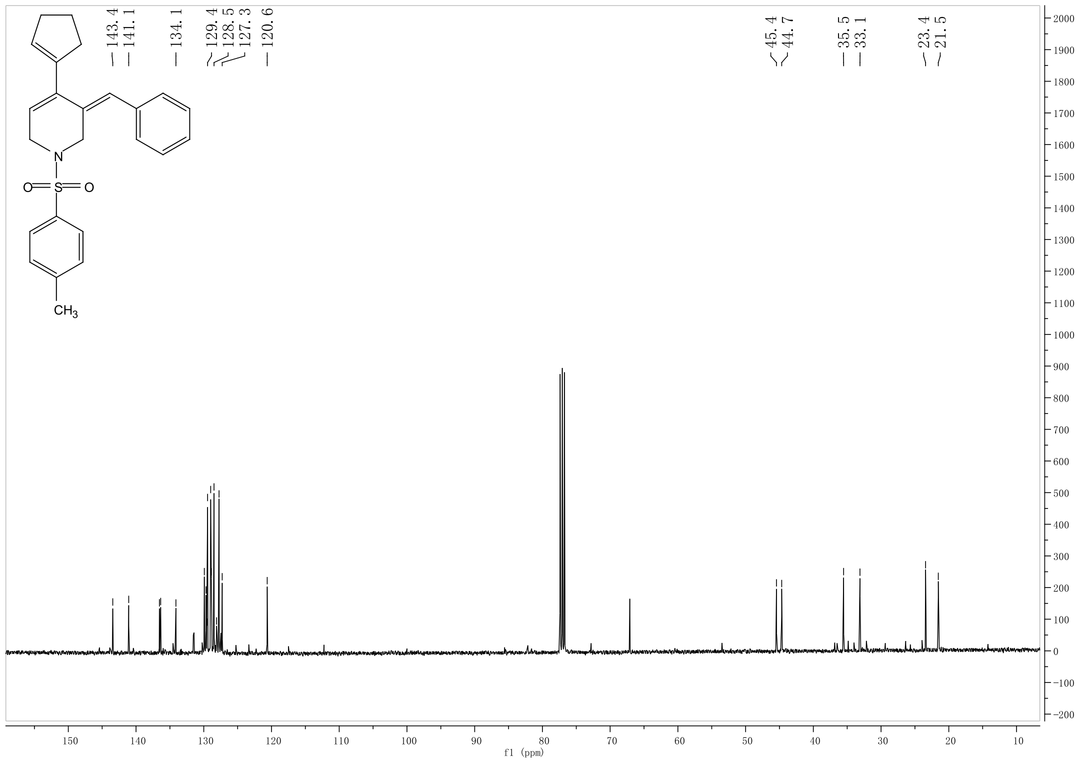

Supplementary Figure 37. ^{1}H and ^{13}C-NMR of **6b**

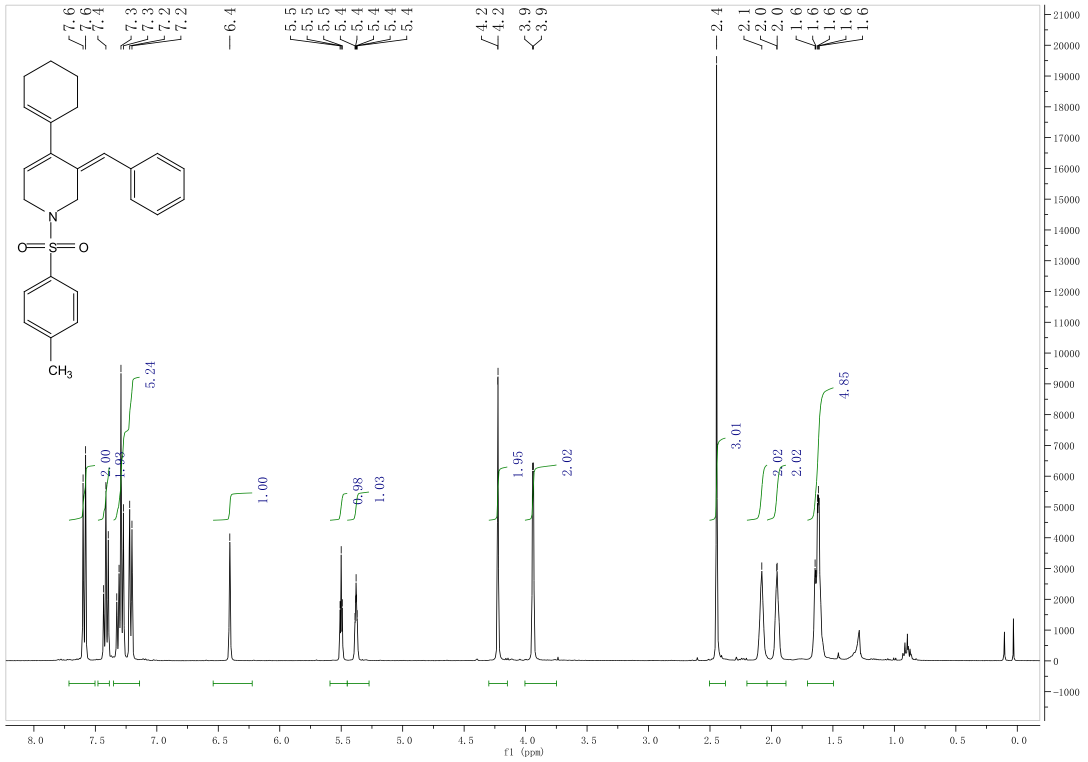

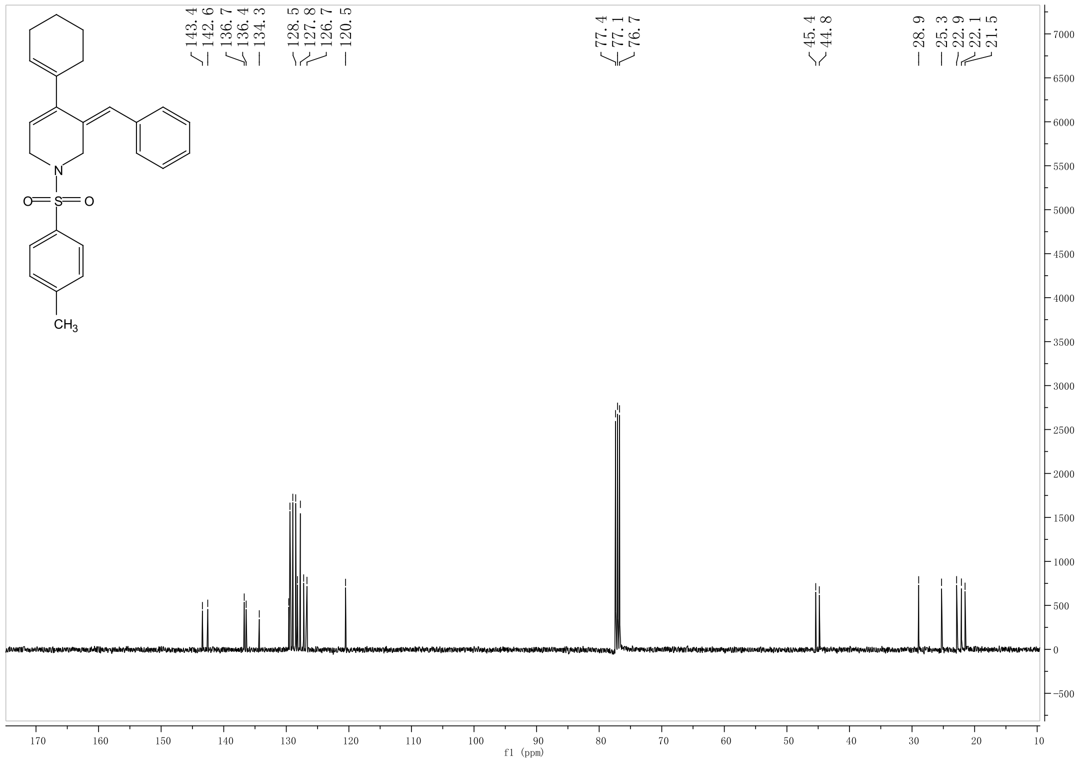

Supplementary Figure 38. ^{1}H and ^{13}C-NMR of **6c**

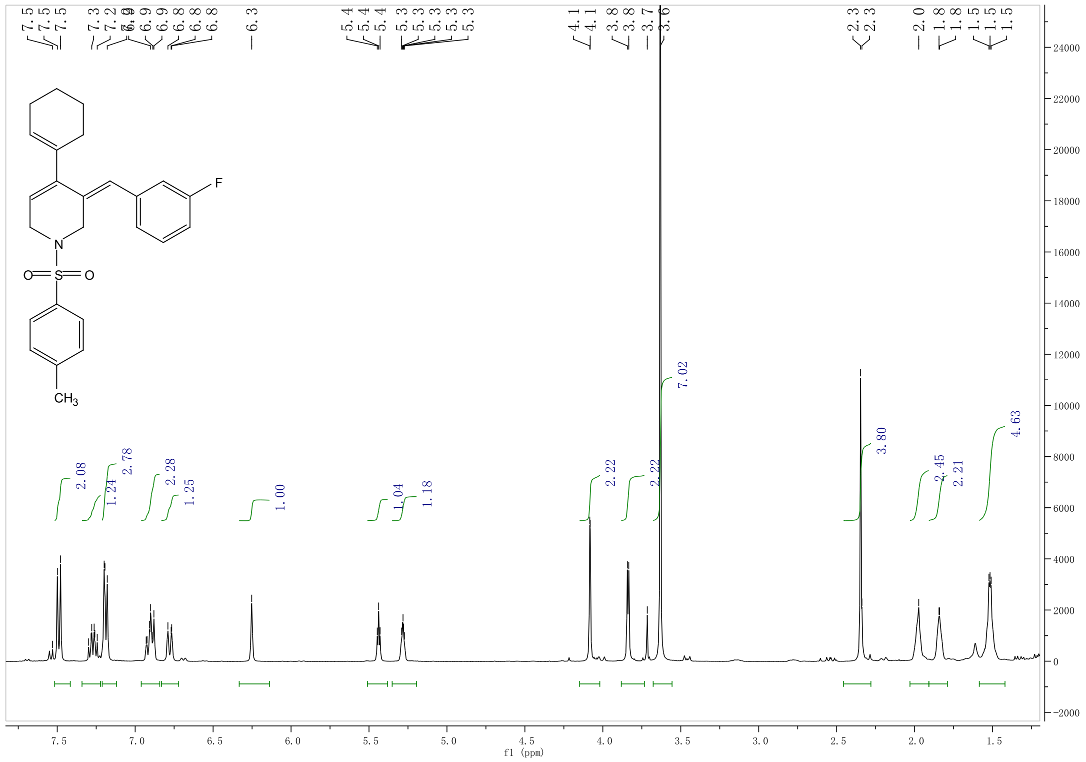

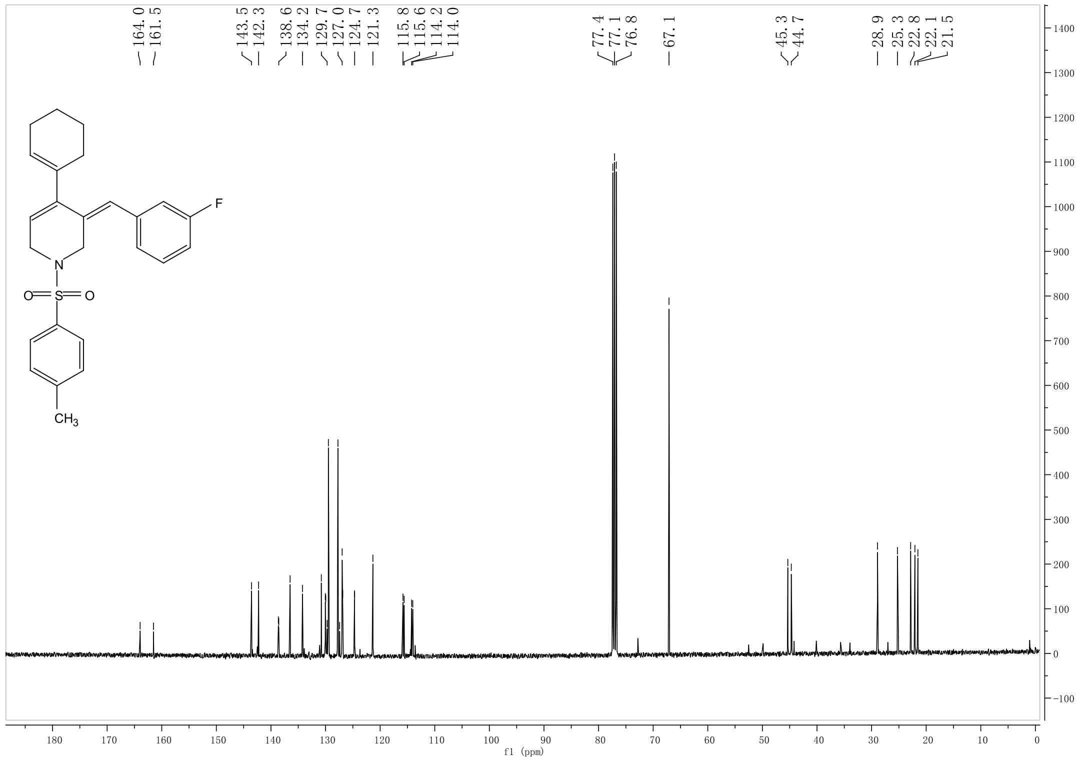

Supplementary Figure 39. ^{1}H and ^{13}C-NMR of **6d**

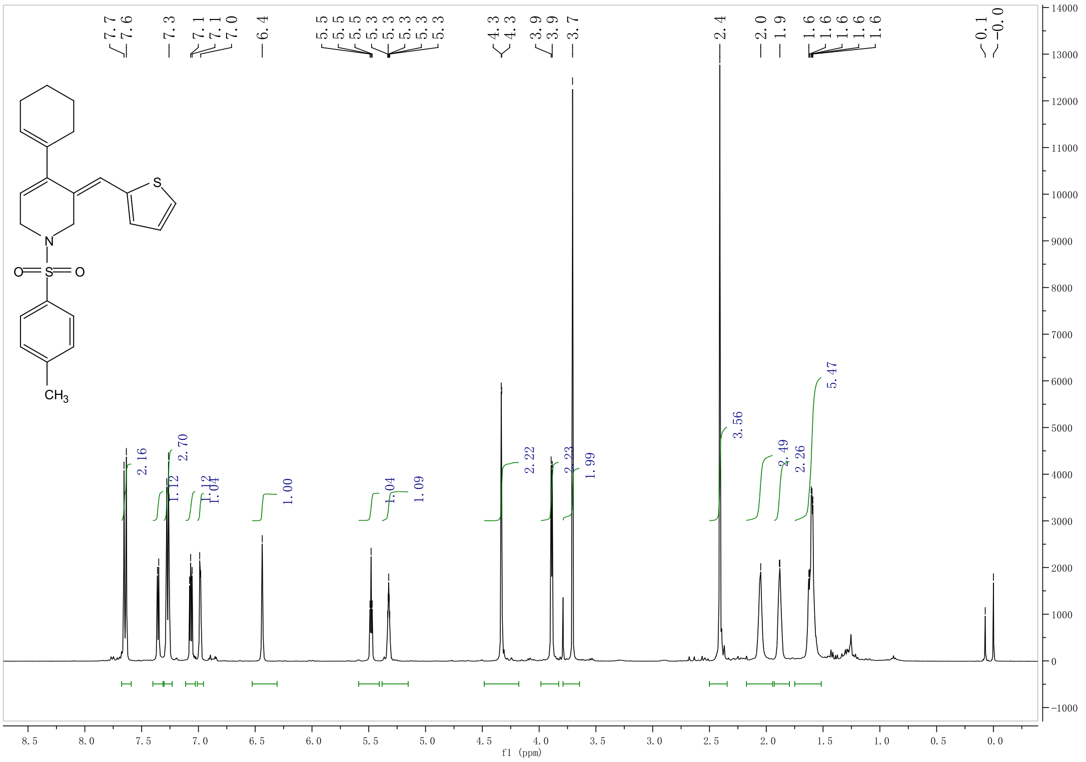

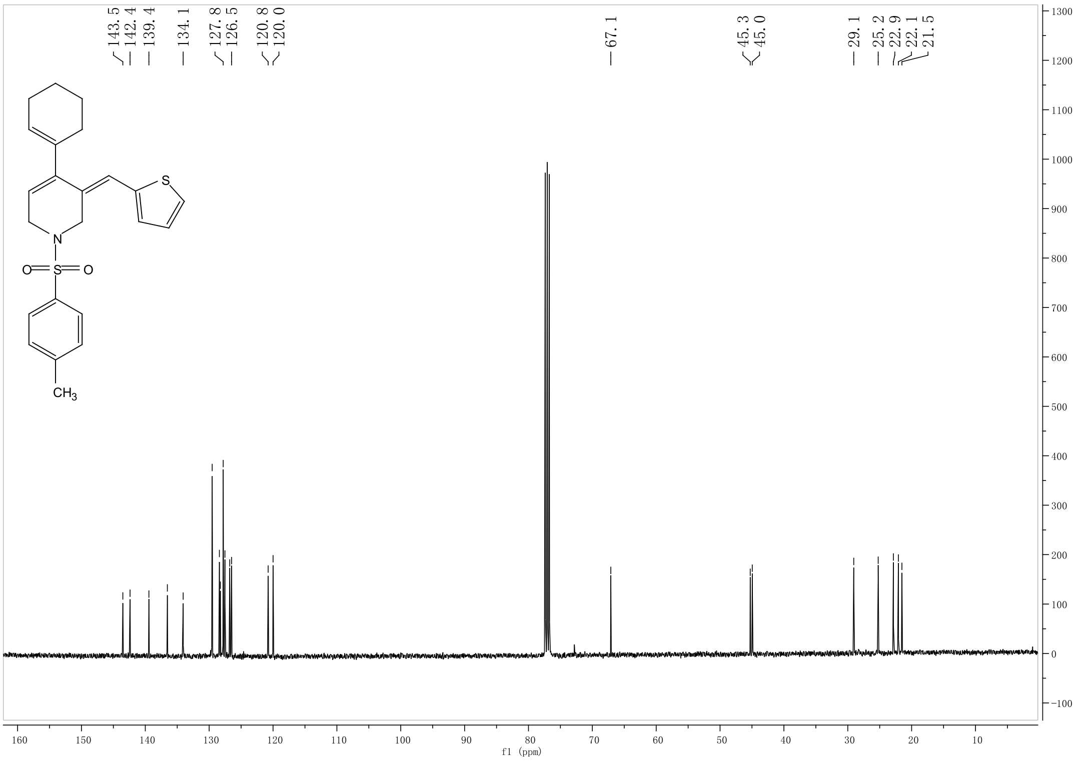

Supplementary Figure 40. Deuterium labeling experiment

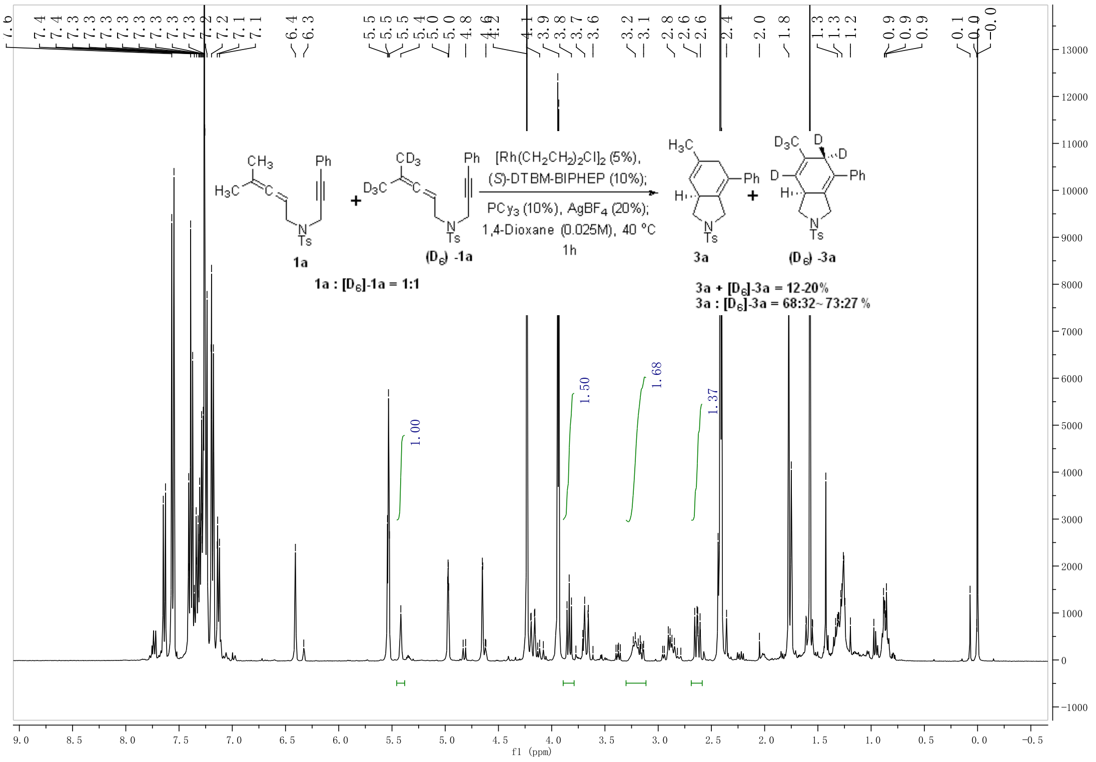

HPLC traces. Racemic and optically active pyrrolidines were analyzed with chiral HPLC to determine retention time and enantiomeric excesses.

Supplementary Figure 41. HPLC (**2a**): Chiracel OD-H column (255 mm), detected at 254 nM; *n*-hexane/*i*-PrOH = 95/5, flow = 0.5 mL/min, Retention time: 34.7 min, 41.9 min (major);

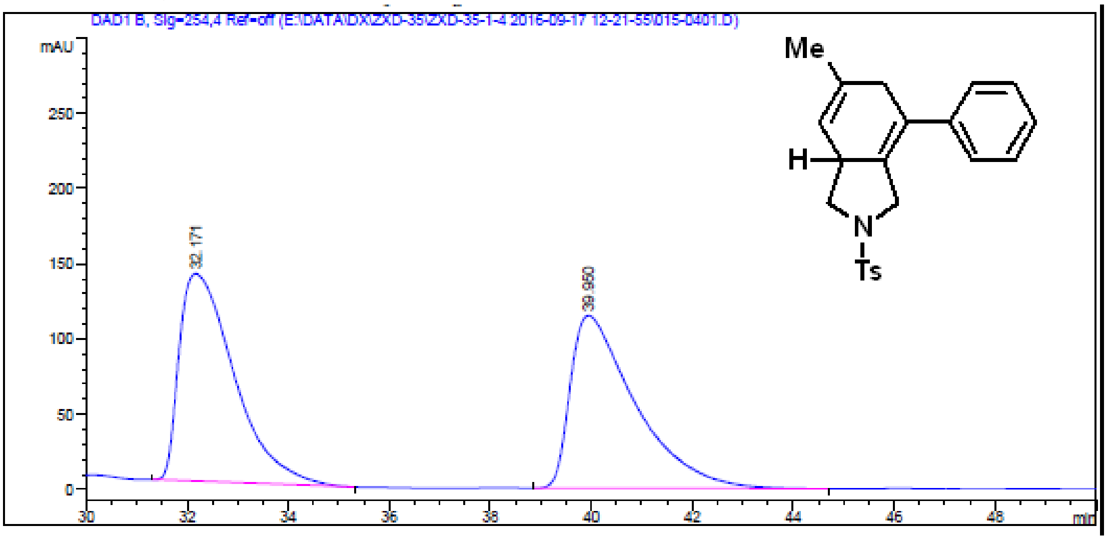

Peak number	Retention time (min)	Area	Height	Area（%）
1	32.1	10439.6	137.7	50.4584
2	39.9	10250.0	114.7	49.5416

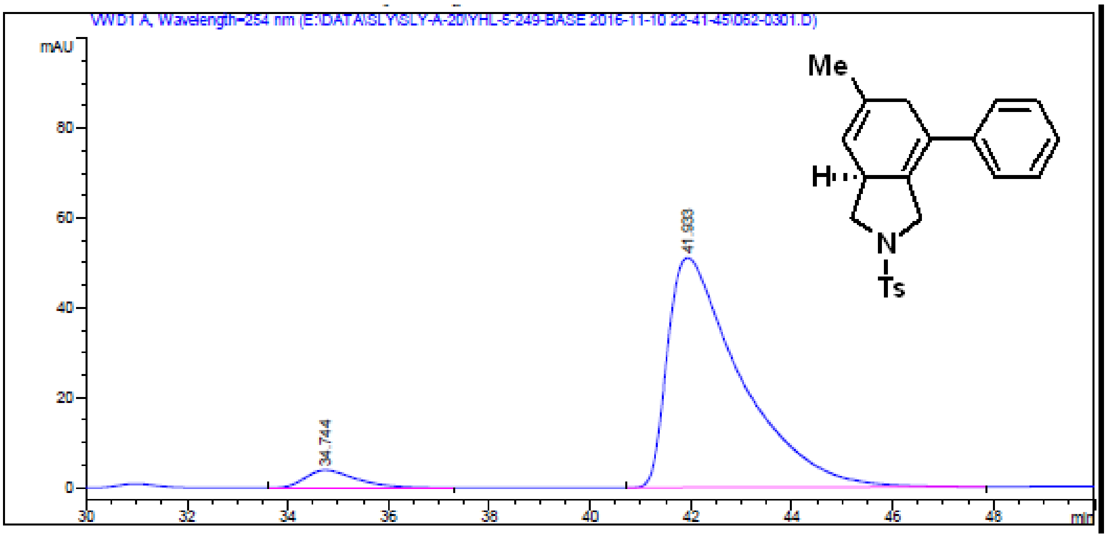

Peak number	Retention time (min)	Area	Height	Area（%）
1	34.7	272.6	3.8	5.0
2	41.9	5159.2	51.1	94.9

Supplementary Figure 42. HPLC (**2b**): Chiracel AD-H column (255 mm), detected at 254 nM; *n*-hexane/*i*-PrOH = 95/5, flow = 0.5 mL/min, Retention time: 46.0 min (major), 47.9 min;

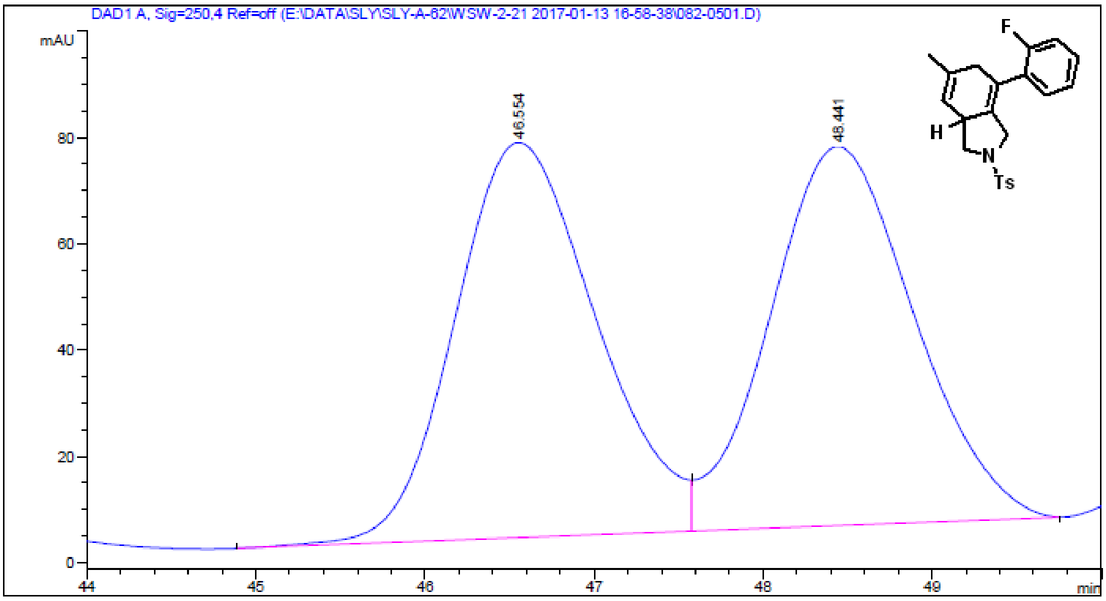

Peak number	Retention time (min)	Area	Height	Area（%）
1	46.5	4311.5	74.3	50.4
2	48.4	4228.8	71.3	49.5

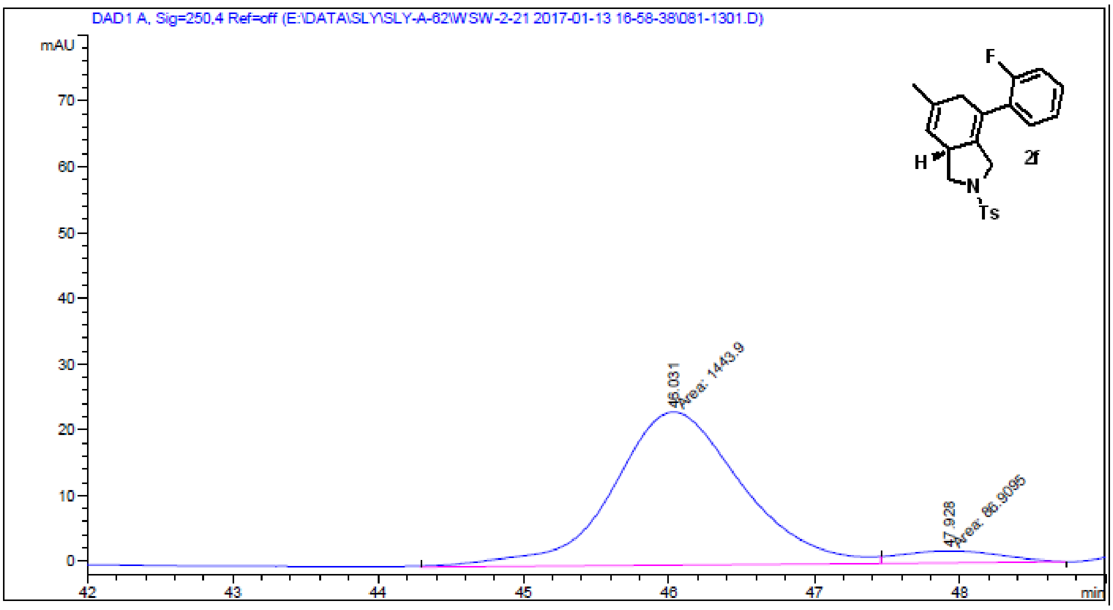

Peak number	Retention time (min)	Area	Height	Area（%）
1	46.0	1443.9	23.2	94.3
2	47.9	86.9	1.8	5.6

Supplementary Figure 43. HPLC (**2c**): Chiracel OD-H column (255 mm), detected at 254 nM; *n*-hexane/*i*-PrOH = 95/5, flow = 0.5 mL/min, Retention time: 27.5 min, 30.6 min (major);

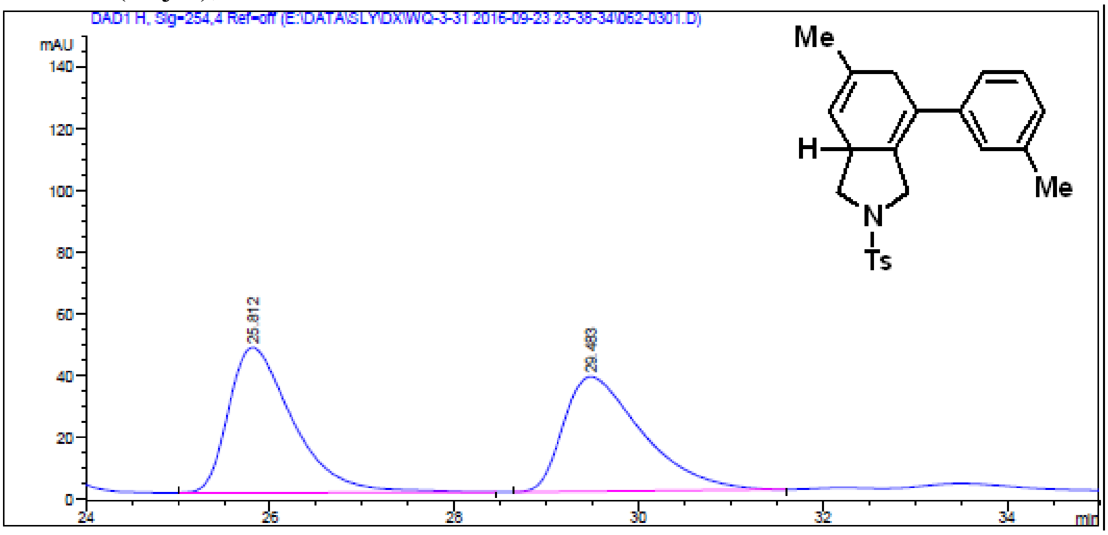

Peak number	Retention time (min)	Area	Height	Area （%）
1	25.8	2290.2	47.0	50.9
2	29.4	2205.0	37.0	49.0

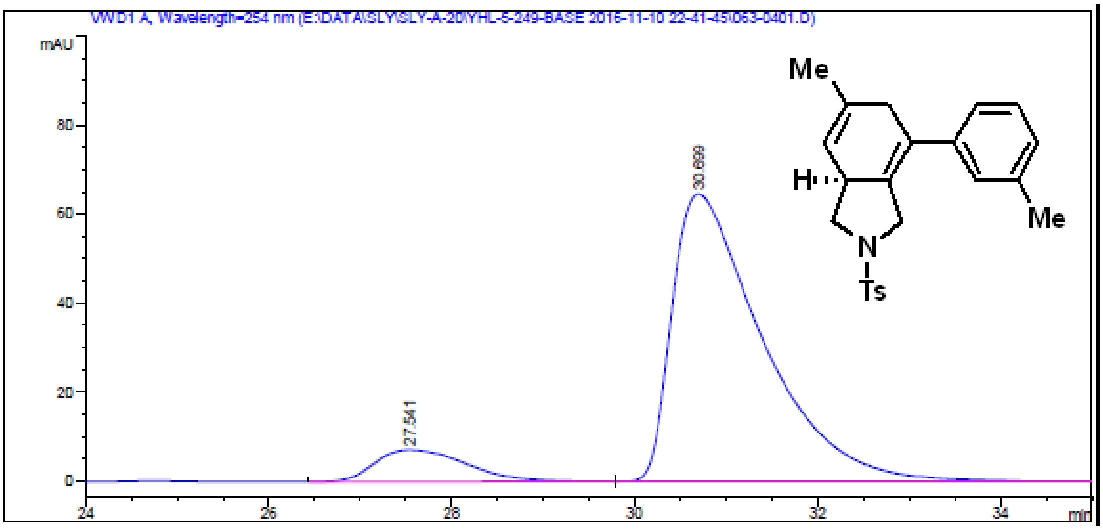

Peak number	Retention time (min)	Area	Height	Area （%）
1	27.5	495.3	7.1	10.0
2	30.6	4446.5	64.6	89.9

Supplementary Figure 44. HPLC (**2d**): Chiracel OD-H column (255 mm), detected at 254 nM; *n*-hexane/*i*-PrOH = 95/5, flow = 0.4 mL/min, Retention time: 44.2 min, 50.6 min (major);

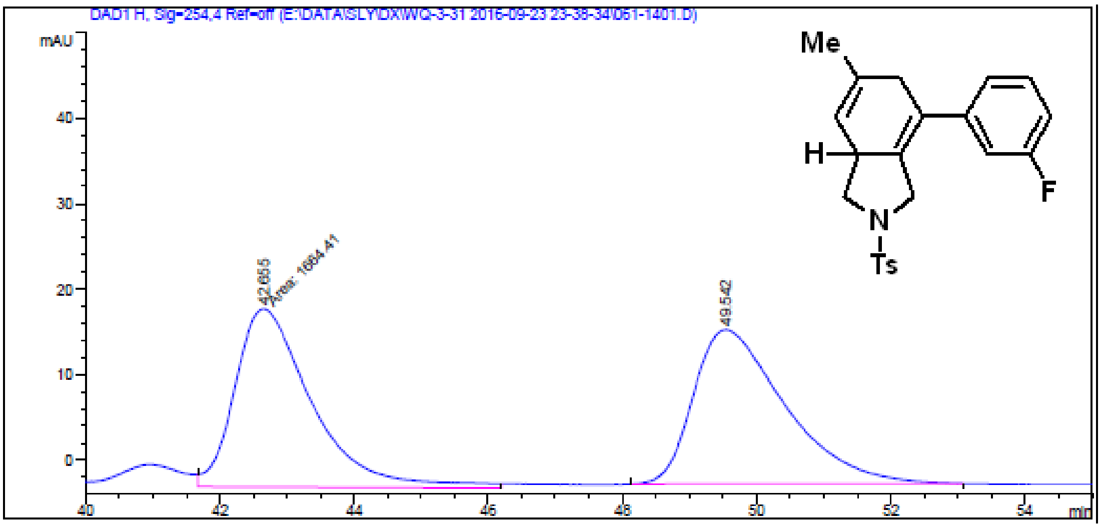

Peak number	Retention time (min)	Area	Height	Area（%）
1	42.6	1664.4	20.8	49.3
2	49.5	1706.6	18.0	50.6

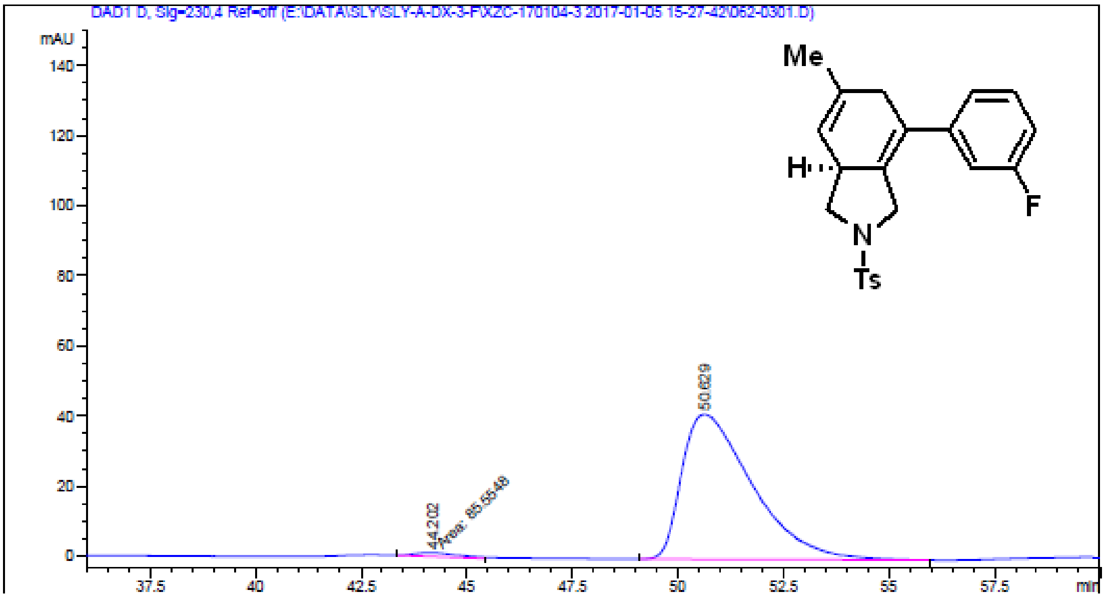

Peak number	Retention time (min)	Area	Height	Area（%）
1	44.2	85.5	1.1	1.7
2	50.6	4807.7	41.3	98.2

Supplementary Figure 45. HPLC (**2e**): Chiracel OD-H column (255 mm), detected at 254 nM; *n*-hexane/*i*-PrOH = 95/5, flow = 0.5 mL/min, Retention time: 41.5 min, 47.7 min (major);

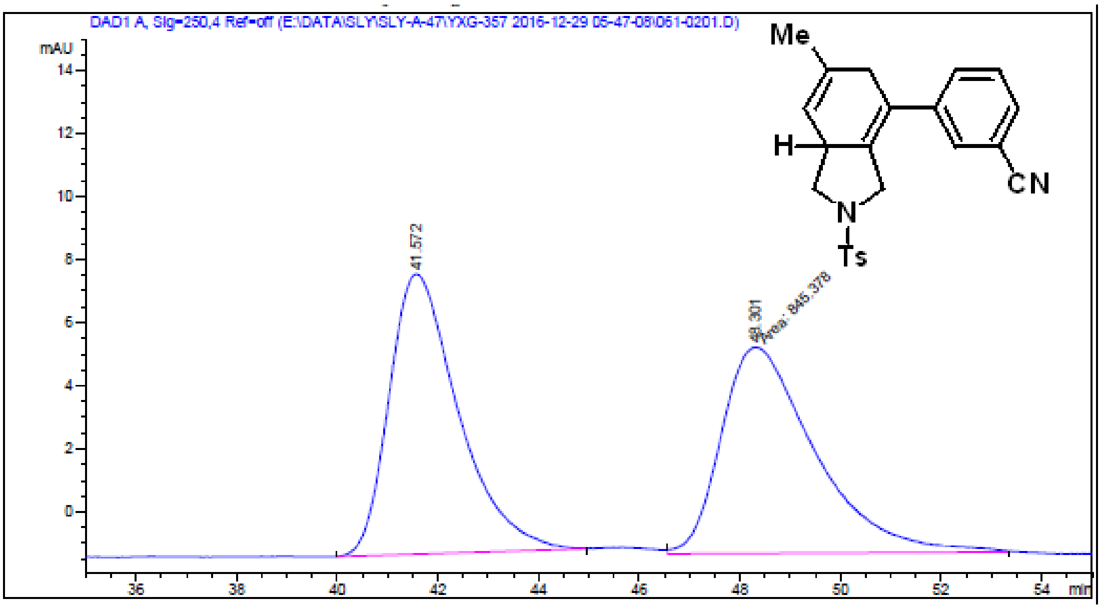

Peak number	Retention time (min)	Area	Height	Area（%）
1	41.5	849.3	8.8	50.1
2	48.3	845.3	6.5	49.8

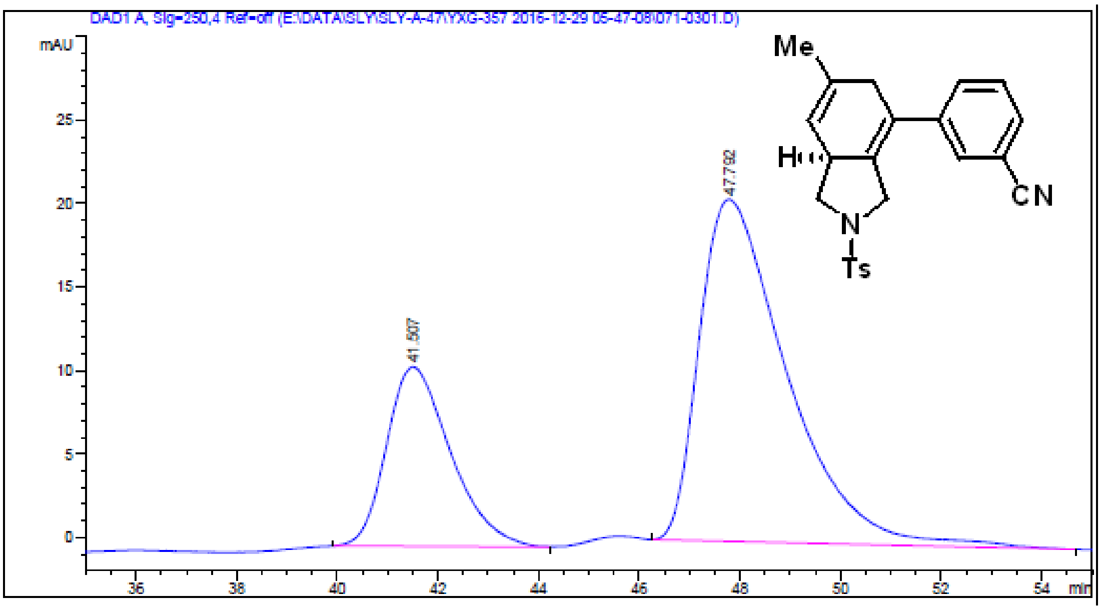

Peak number	Retention time (min)	Area	Height	Area（%）
1	41.5	948.6	10.7	27.6
2	47.7	2481.2	20.4	72.3

Supplementary Figure 46. HPLC (**2f**): Chiracel AD-H column (255 mm), detected at 254 nM; *n*-hexane/*i*-PrOH = 90/10, flow = 0.6 mL/min, Retention time: 23.1 min (major), 24.3 min;

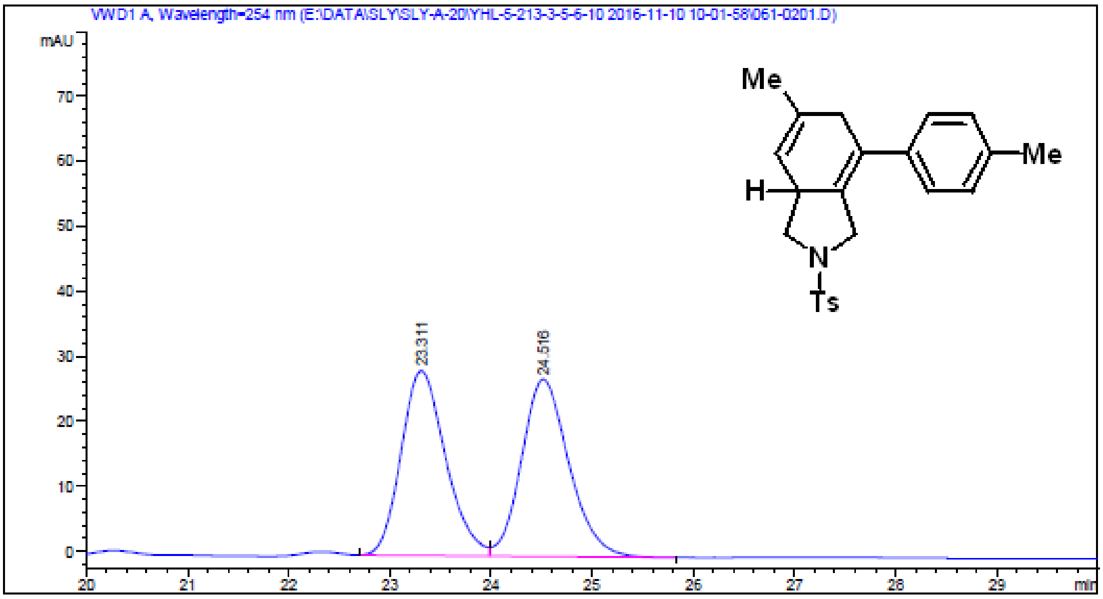

Peak number	Retention time (min)	Area	Height	Area（%）
1	23.3	868.8	28.4	49.4
2	24.5	886.9	27.2	50.5

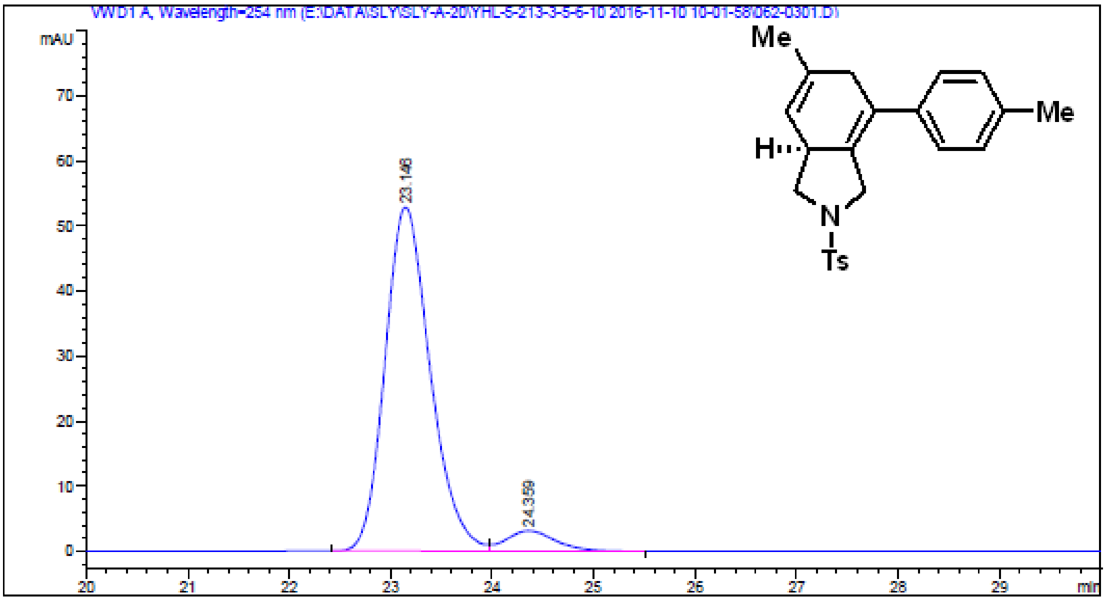

Peak number	Retention time (min)	Area	Height	Area（%）
1	23.1	1636.4	52.8	93.9
2	24.3	105.2	3.1	6.0

Supplementary Figure 47. HPLC (**2g**): Chiracel AS-H column (255 mm), detected at 254 nM; *n*-hexane/*i*-PrOH = 85/15, flow = 0.8 mL/min, Retention time: 53.1 min, 64.3 min (major);

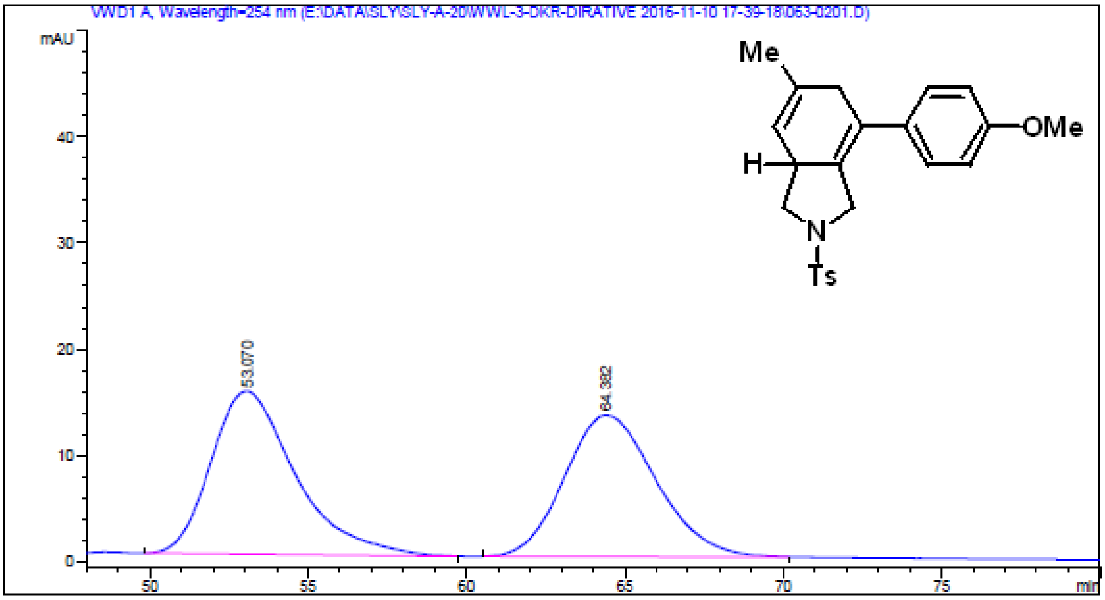

Peak number	Retention time (min)	Area	Height	Area（%）
1	53.0	2840.2	15.3	51.5
2	64.3	2672.8	13.3	48.5

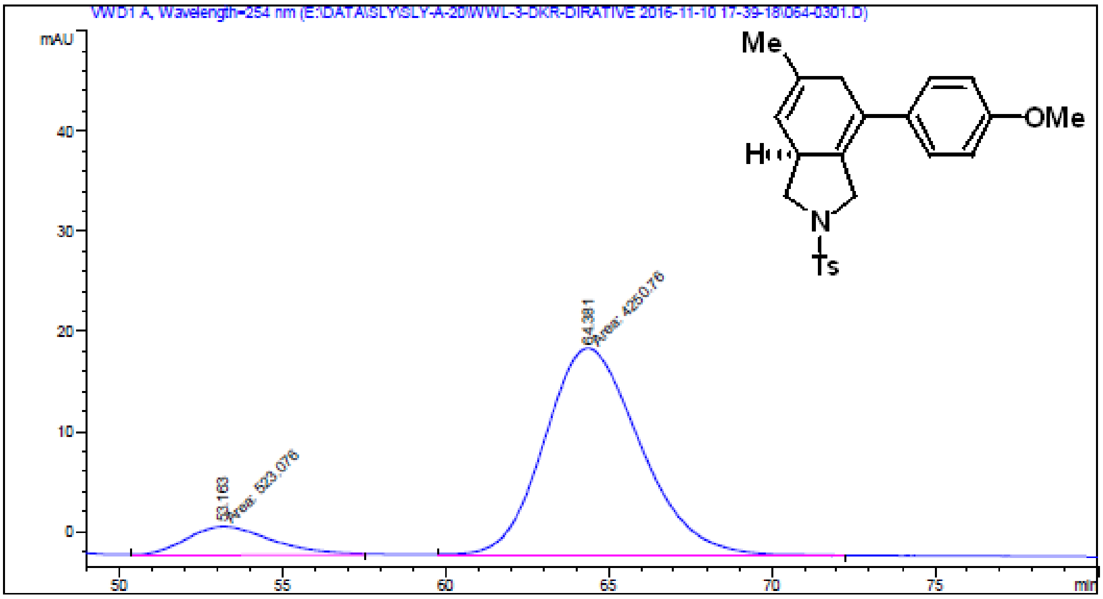

Peak number	Retention time (min)	Area	Height	Area（%）
1	53.1	523.1	2.8	10.9
2	64.3	4250.7	20.7	89.0

Supplementary Figure 48. HPLC (**2h**): Chiracel AD-H column (255 mm), detected at 254 nM; *n*-hexane/*i*-PrOH = 80/20, flow = 0.5 mL/min, Retention time: 22.2 min, 22.7 min (major);

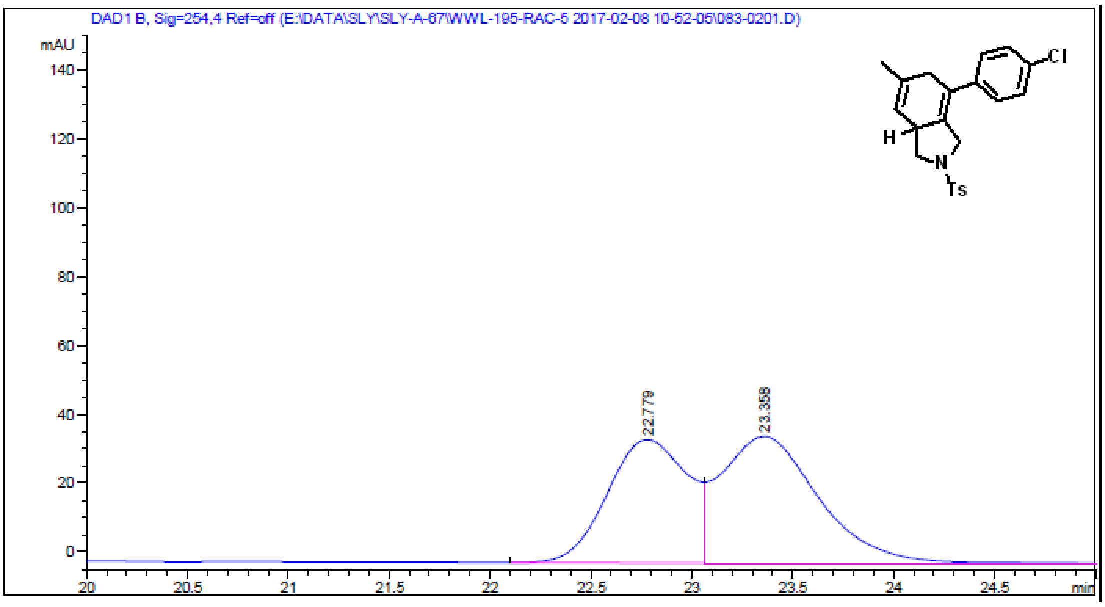

Peak number	Retention time (min)	Area	Height	Area （%）
1	22.7	987.5	35.7	44.4
2	23.3	1238.5	36.7	55.6

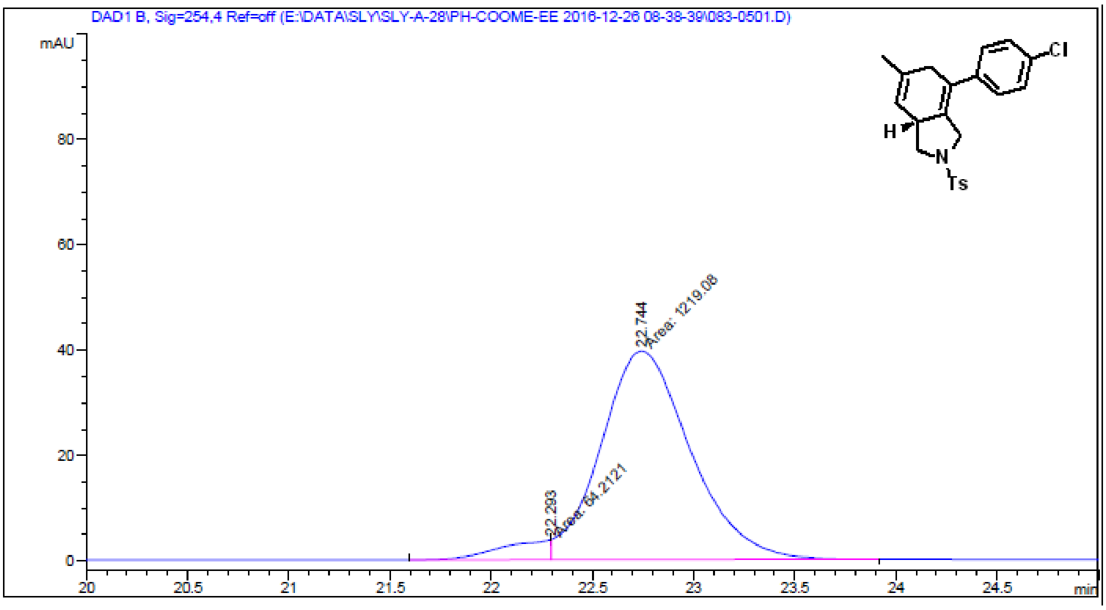

Peak number	Retention time (min)	Area	Height	Area （%）
1	22.2	64.2	3.8	5.0
2	22.7	1219.1	39.6	95.0

Supplementary Figure 49. HPLC (**2i**): Chiracel AD-H column (255 mm), detected at 254 nM; *n*-hexane/*i*-PrOH = 90/10, flow = 0.4 mL/min, Retention time: 48.2 min, 49.5 min (major);

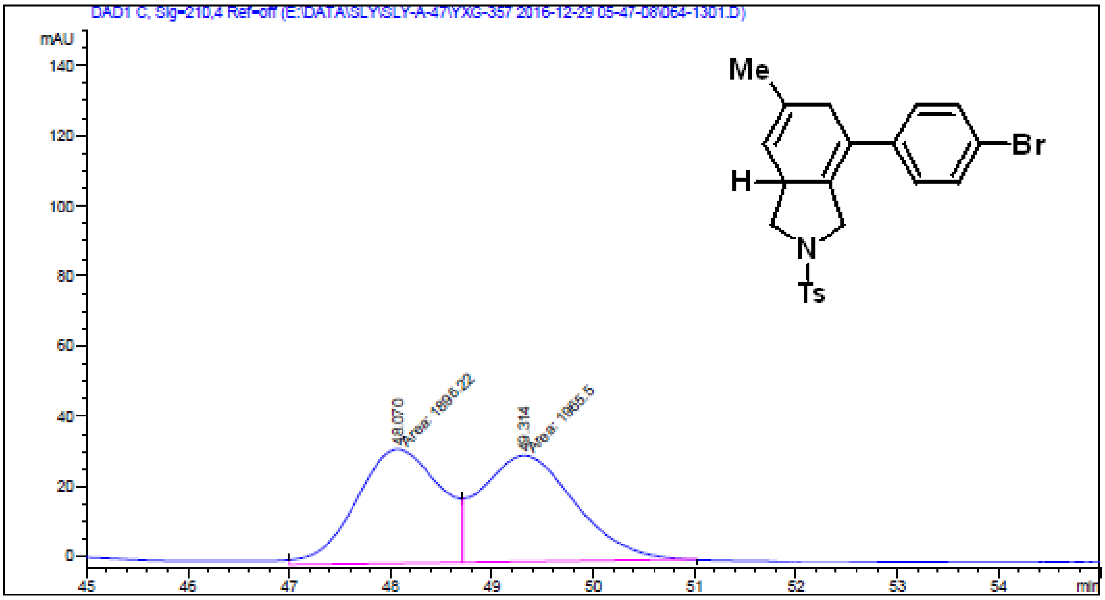

Peak number	Retention time (min)	Area	Height	Area（%）
1	48.0	1896.2	32.5	49.1
2	49.3	1965.5	30.2	50.9

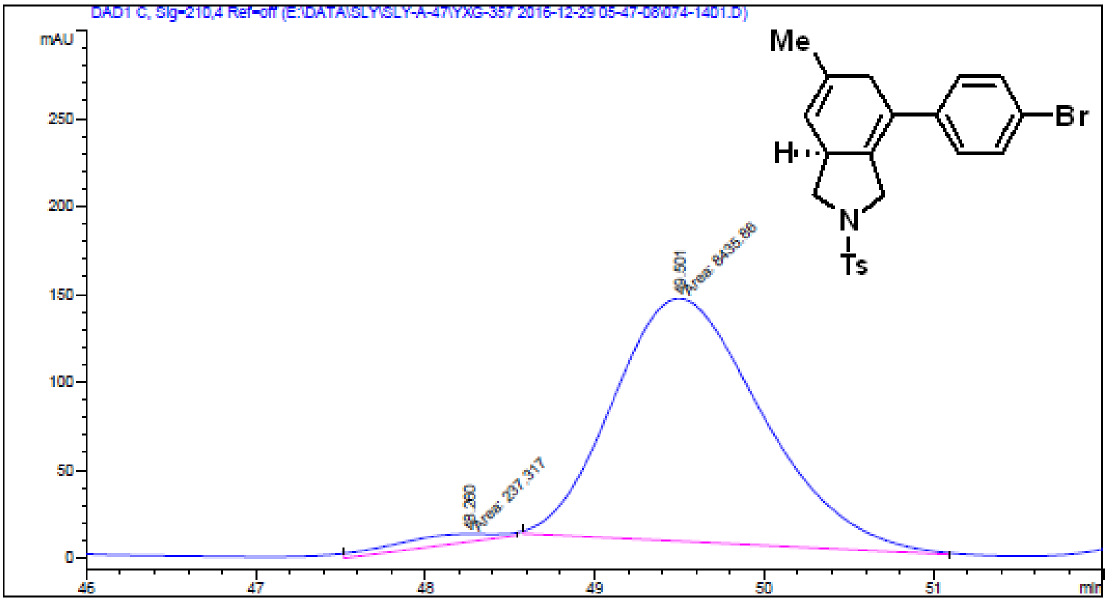

Peak number	Retention time (min)	Area	Height	Area（%）
1	48.2	237.3	4.4	2.7
2	49.5	8435.8	138.1	97.3

Supplementary Figure 50. HPLC (**2j**): Chiracel AD-H column (255 mm), detected at 254 nM; *n*-hexane/*i*-PrOH = 95/5, flow = 0.5 mL/min, Retention time: 68.2 min (major), 79.5 min;

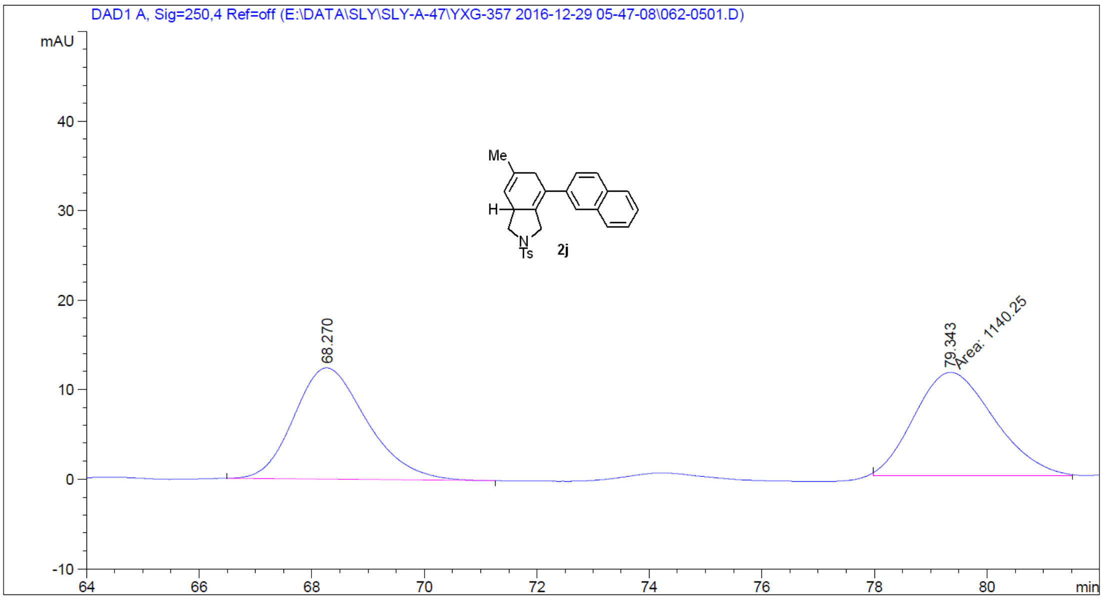

Peak number	Retention time (min)	Area	Height	Area（%）
1	68.2	1120.5	12.4	49.5
2	79.3	1140.2	11.5	50.4

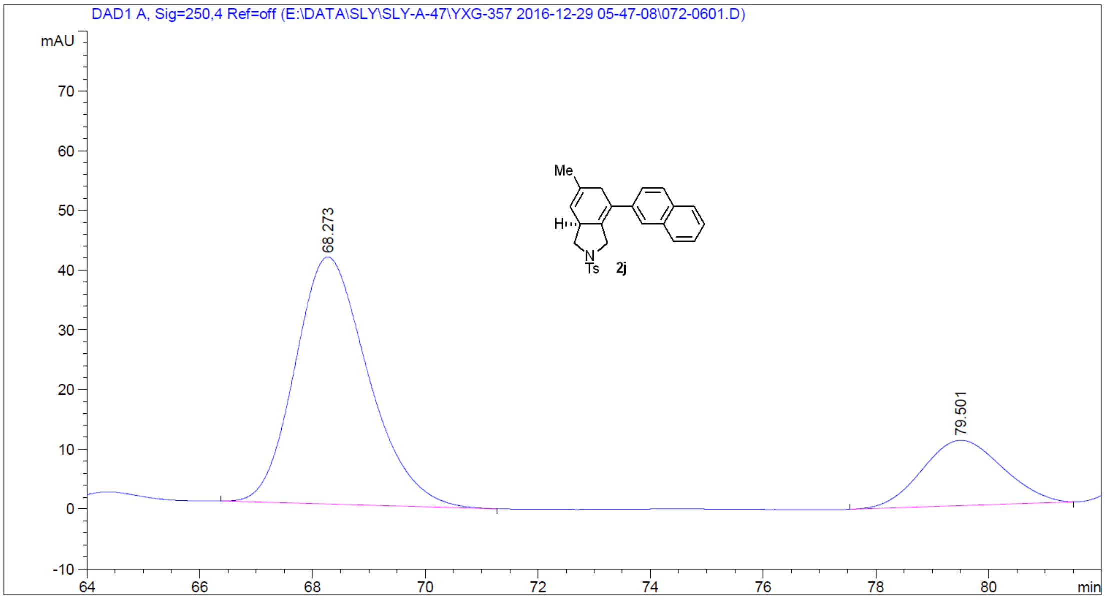

Peak number	Retention time (min)	Area	Height	Area（%）
1	68.2	3736.7	41.3	77.5
2	79.5	1084.4	10.9	22.4

Supplementary Figure 51. HPLC (**2k**): Chiracel IB column (255 mm), detected at 254 nM; *n*-hexane/*i*-PrOH = 90/10, flow = 0.5 mL/min, Retention time: 27.7 min, 29.5 min (major);

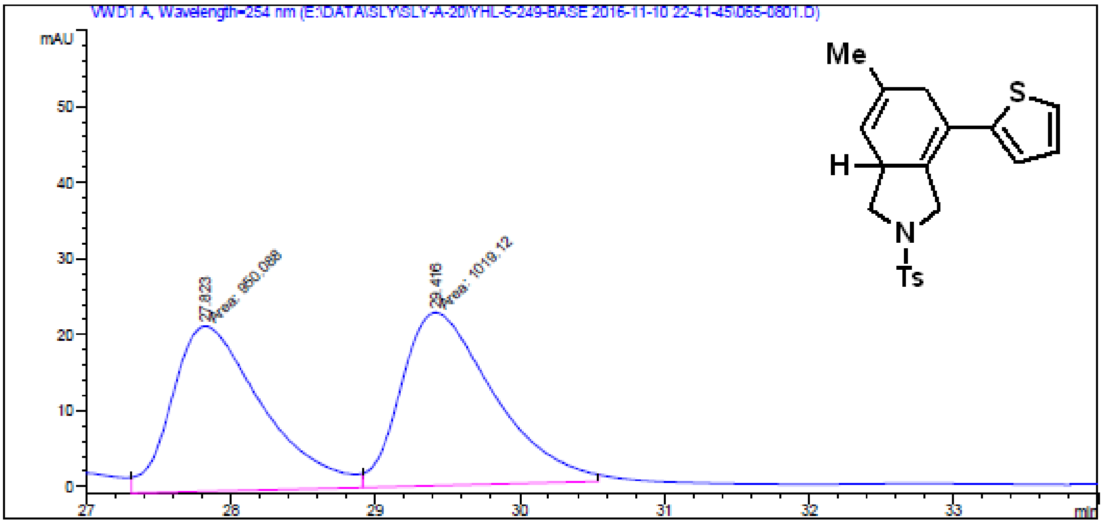

Peak number	Retention time (min)	Area	Height	Area（%）
1	27.8	950.1	21.7	48.2
2	29.4	1019.1	22.7	51.8

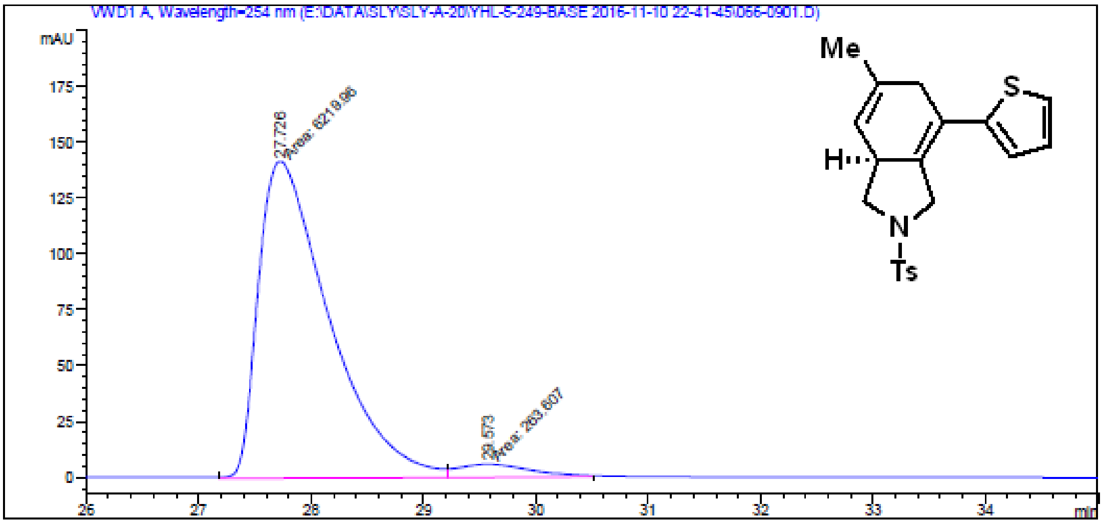

Peak number	Retention time (min)	Area	Height	Area（%）
1	27.7	6219.9	141.7	95.9
2	29.5	263.6	5.8	4.1

Supplementary Figure 52. HPLC (**5a**): Chiracel OD column (255 mm), detected at 254 nM; *n*-hexane/*i*-PrOH = 97/3, flow = 0.5 mL/min, Retention time: 45.7 min (major), 49.8 min;

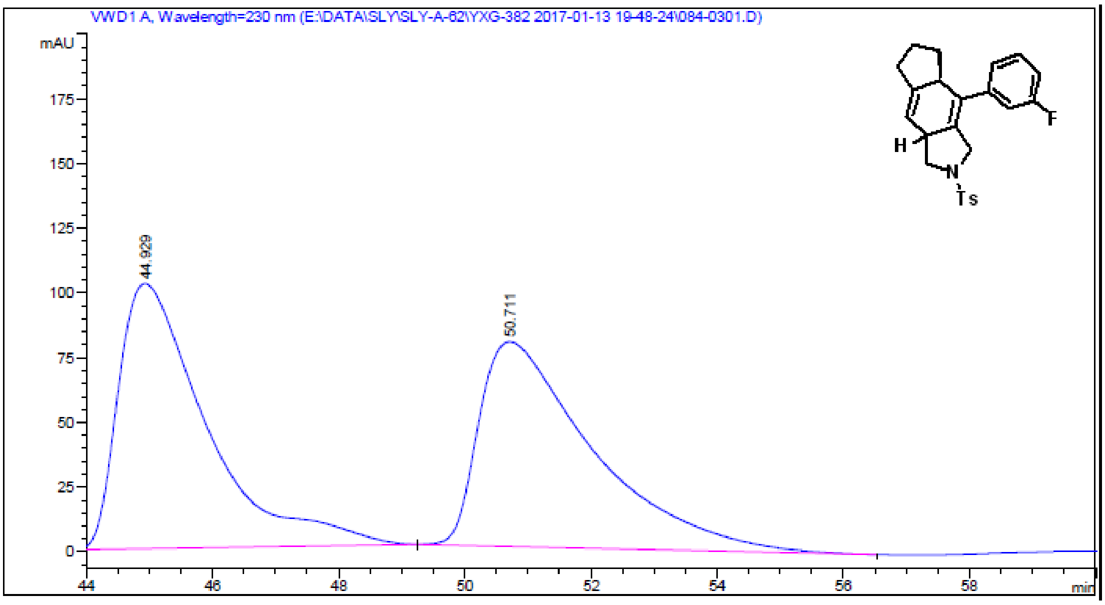

Peak number	Retention time (min)	Area	Height	Area（%）
1	44.9	9948.5	102.4	50.1
2	50.7	9891.2	79.0	49.8

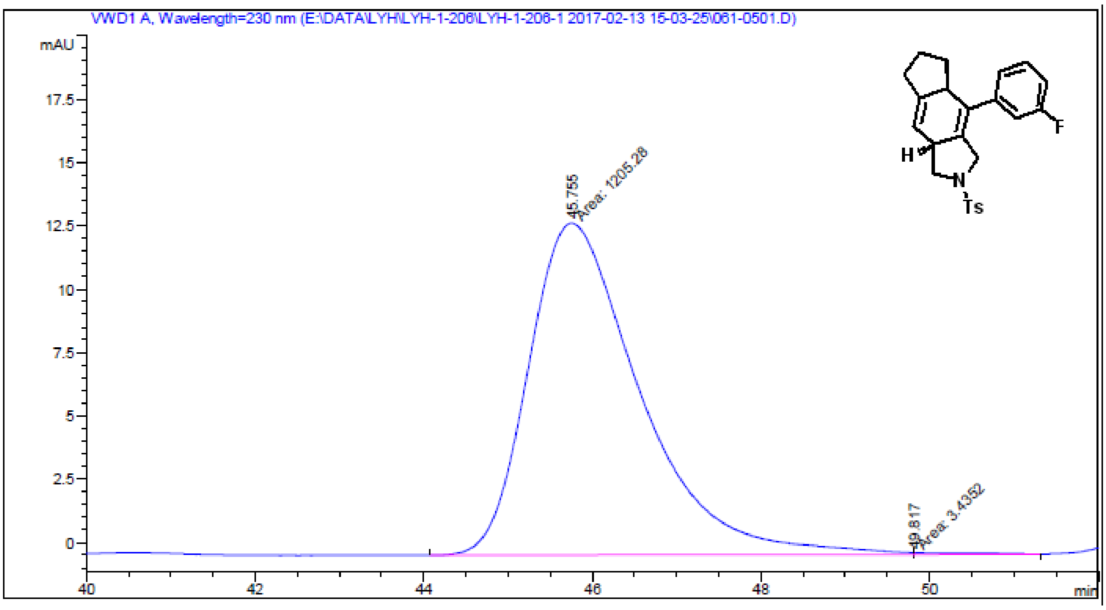

Peak number	Retention time (min)	Area	Height	Area（%）
1	45.7	1205.2	13.1	99.7
2	49.8	3.4	0.065	3.4

Supplementary Figure 53. HPLC (**5b**): Chiracel AD column (255 mm), detected at 254 nM; *n*-hexane/*i*-PrOH = 90/10, flow = 1.0 mL/min, Retention time: 9.1 min, 14.7 min (major);

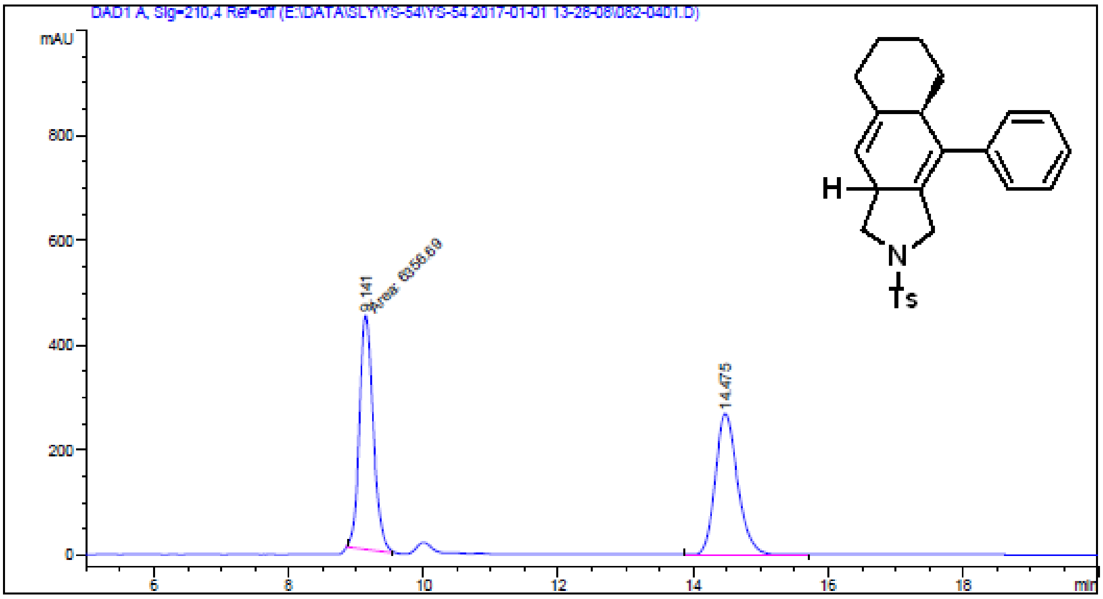

Peak number	Retention time (min)	Area	Height	Area（%）
1	9.1	6356.6	446.1	50.7
2	14.7	6171.1	268.6	49.2

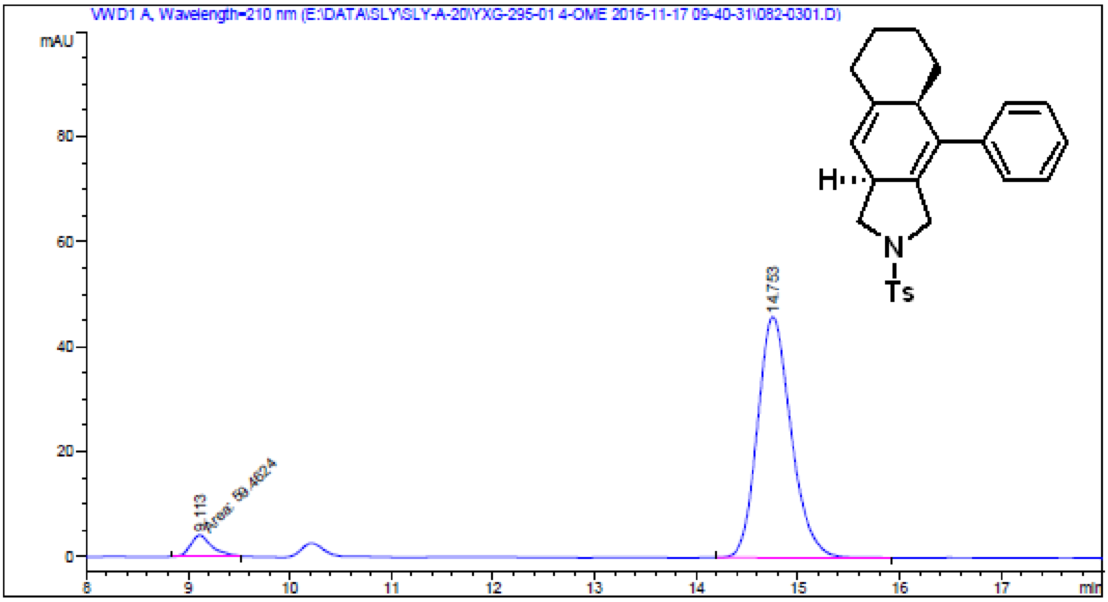

Peak number	Retention time (min)	Area	Height	Area（%）
1	9.1	59.4	4.0	5.3
2	14.7	1055.3	45.8	94.7

Supplementary Figure 54. HPLC (**5c**): Chiracel AD column (255 mm), detected at 254 nM; *n*-hexane/*i*-PrOH = 95/5, flow = 0.5 mL/min, Retention time: 27.3 min, 38.6 min (major);

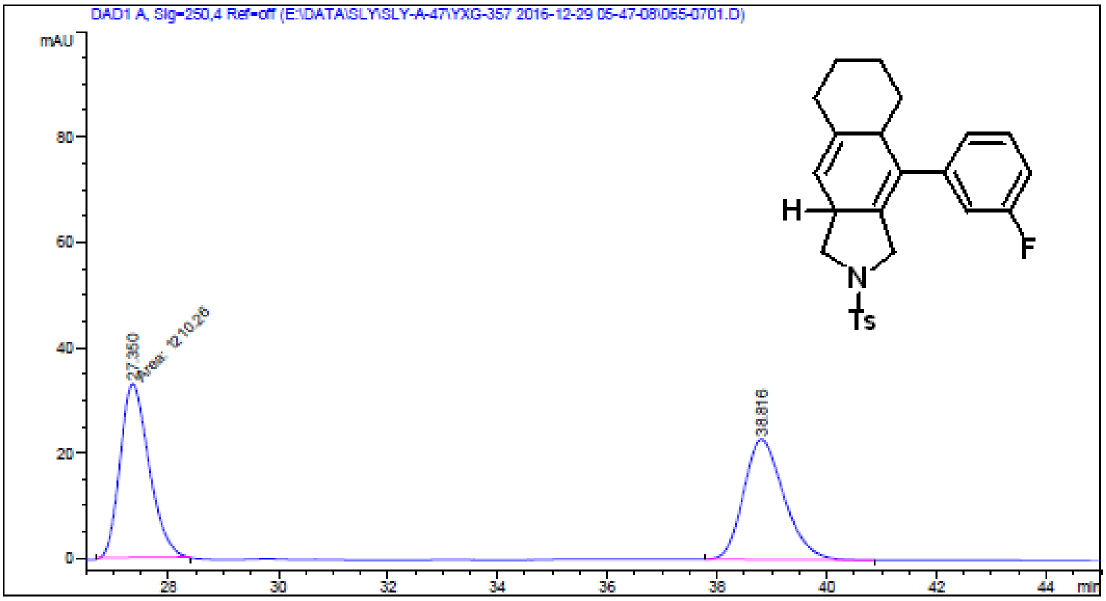

Peak number	Retention time (min)	Area	Height	Area（%）
1	27.3	1210.2	32.9	50.9
2	38.8	1167.9	22.7	49.1

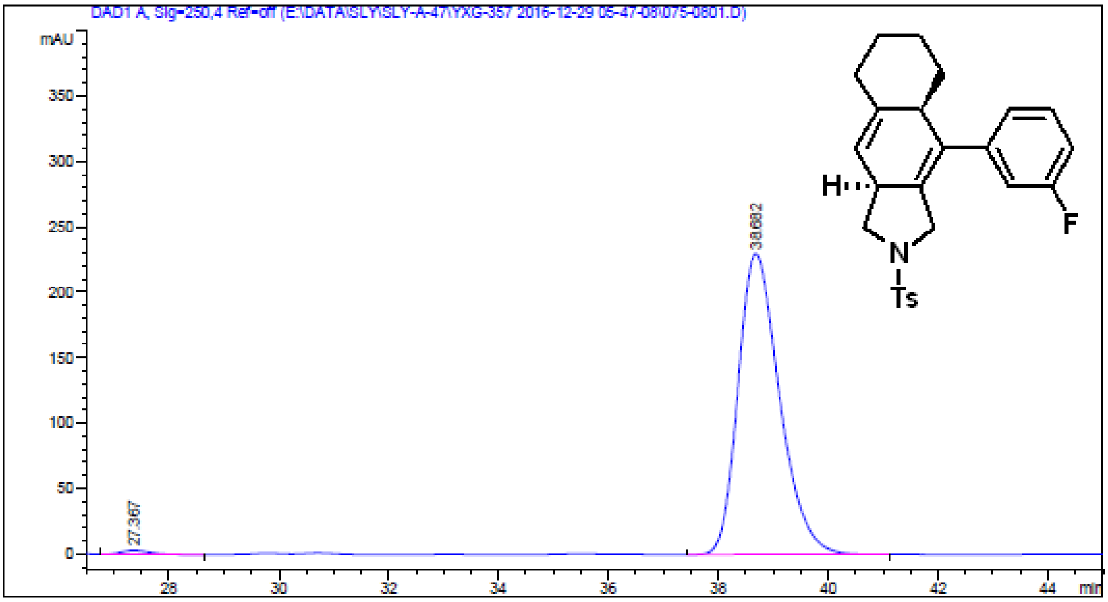

Peak number	Retention time (min)	Area	Height	Area（%）
1	27.3	108.4	3.0	0.8
2	38.6	12029.0	230.0	99.2

Supplementary Figure 55. HPLC (**5d**): Chiracel OD column (255 mm), detected at 254 nM; *n*-hexane/*i*-PrOH = 90/10, flow = 0.5 mL/min, Retention time: 20.5 min, 21.6 min (major);

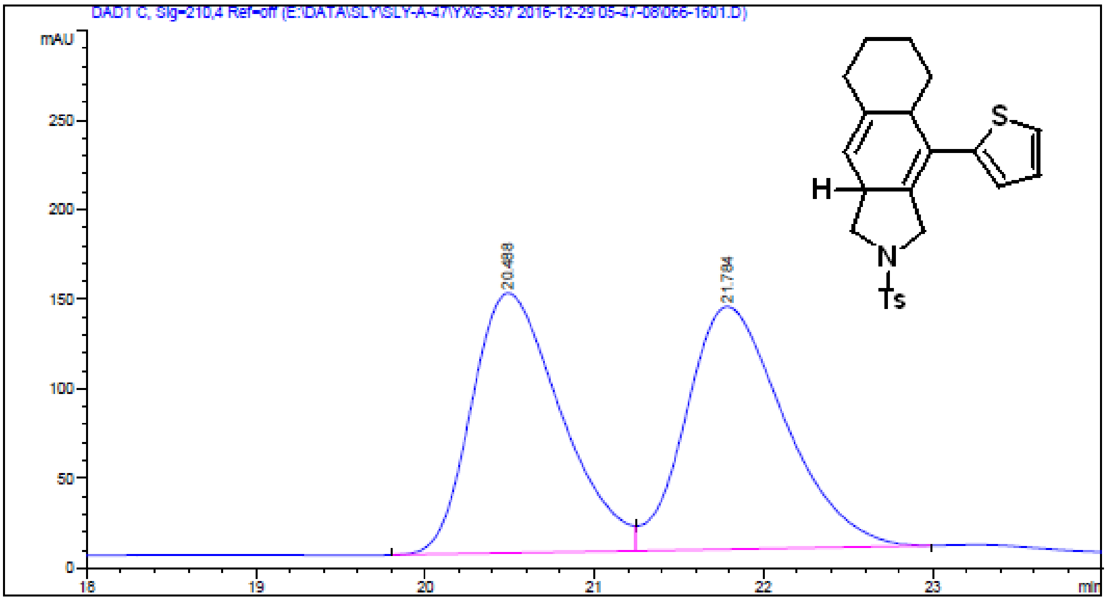

Peak number	Retention time (min)	Area	Height	Area（%）
1	20.4	5318.7	144.8	49.2
2	21.7	5484.1	135.3	50.7

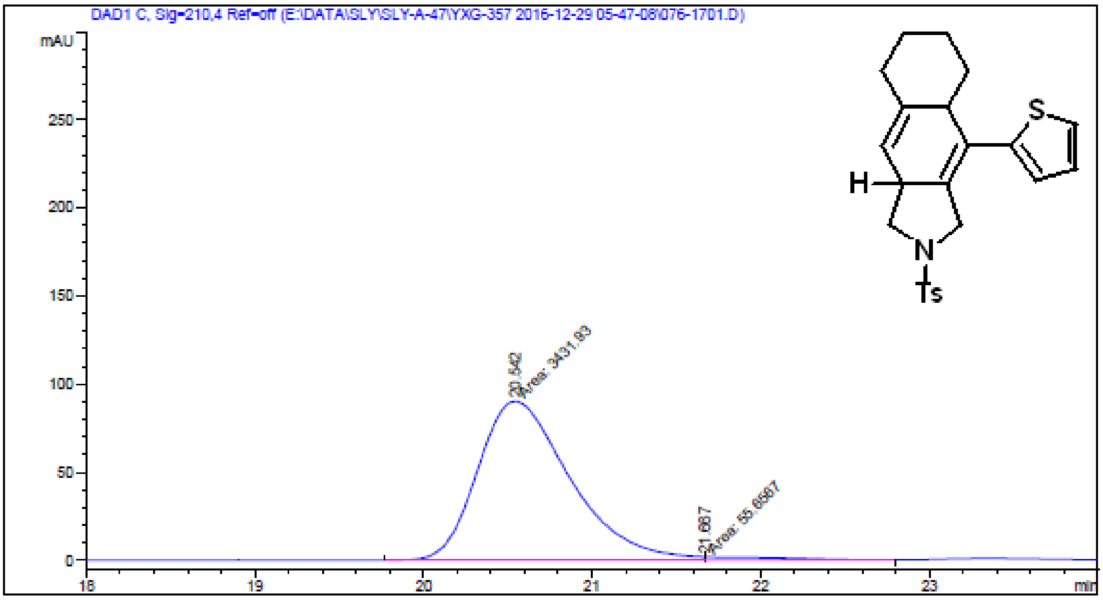

Peak number	Retention time (min)	Area	Height	Area（%）
1	20.5	3431.9	90.0	98.4
2	21.6	55.6	1.7	1.6

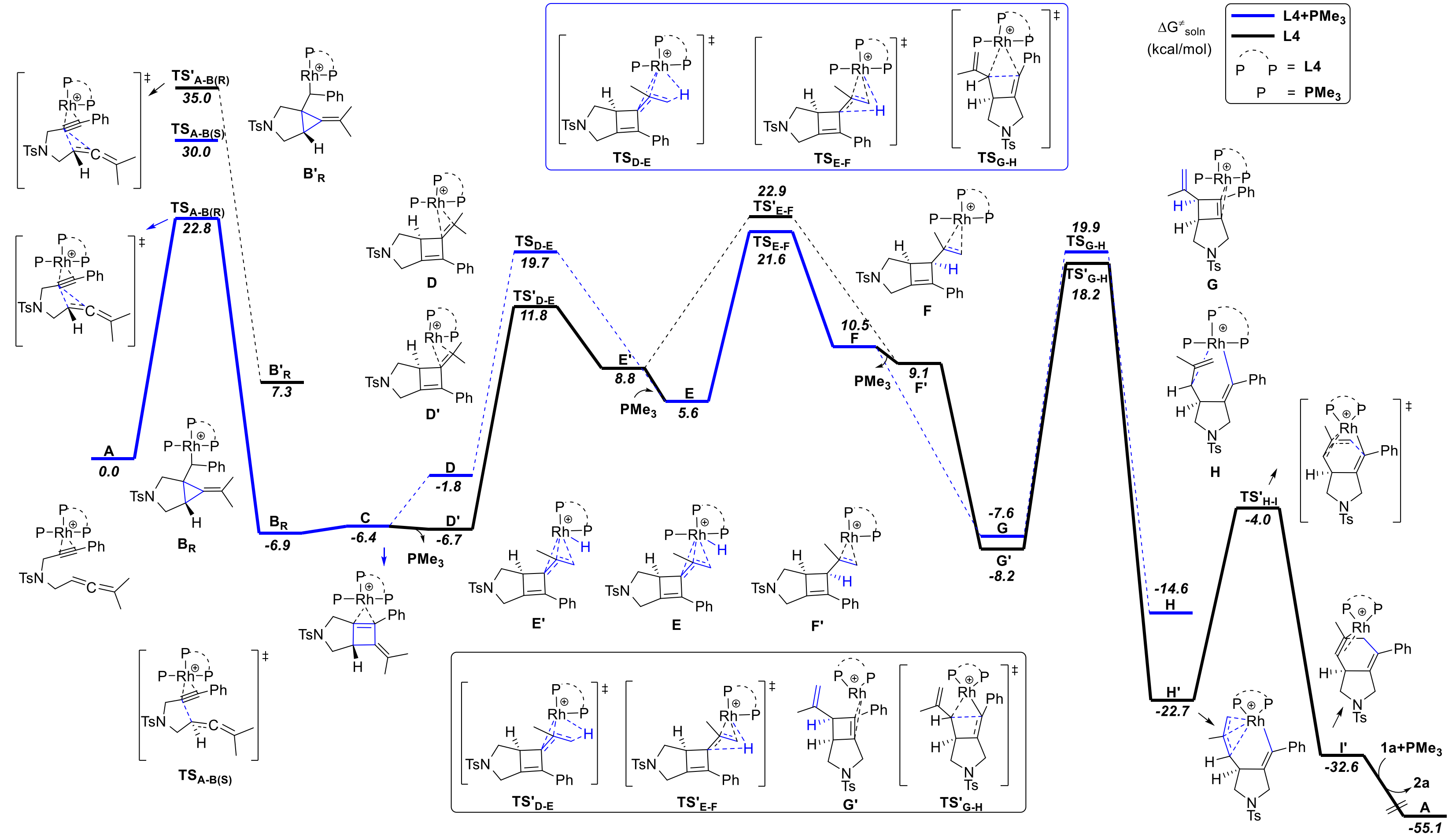

Supplementary Figure 56. The computed free energy profiles for the pathway **i** of the Rh(I)-catalyzed cycloisomerization of 1,6-allenyne (**1a**) in solution by SMD M06-L//M06-L method. PMe_3 ligand was used as the additional ligand.

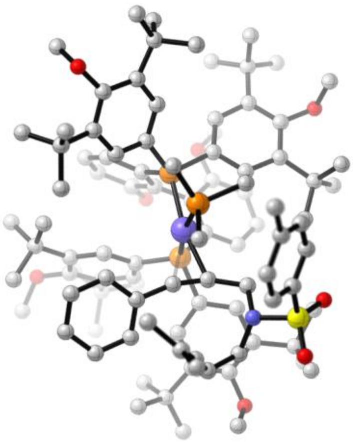

A 0.0

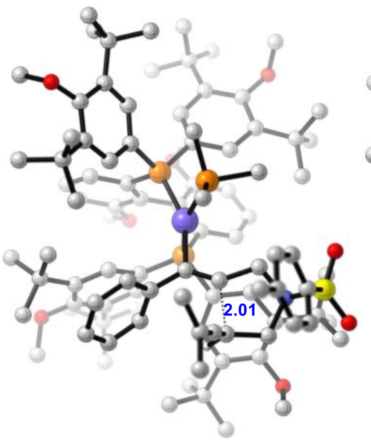

TS$_{A\text{-}B(R)}$ 22.8

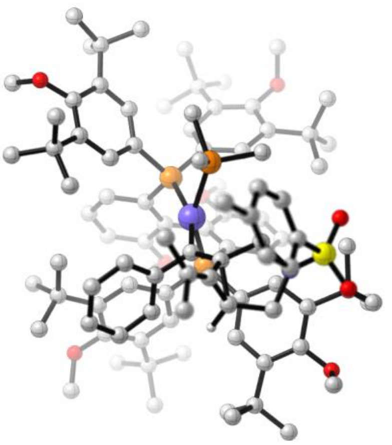

B$_R$ -6.9

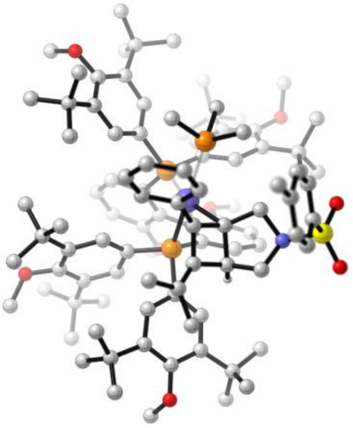

C -6.4

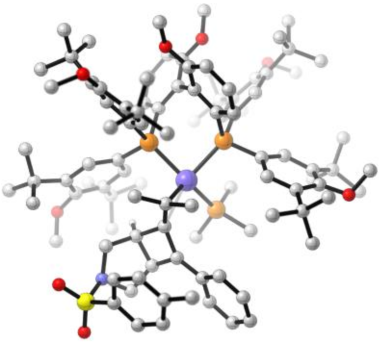

D -1.8

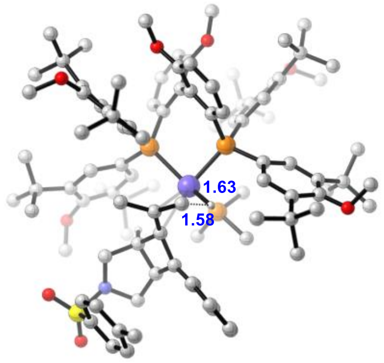

TS$_{D\text{-}E}$ 19.7

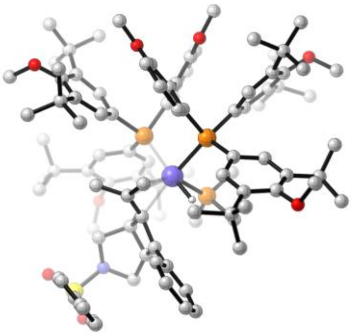

E 5.6

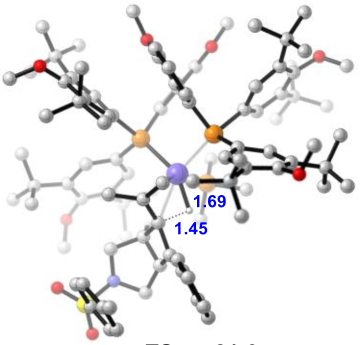

TS$_{E\text{-}F}$ 21.6

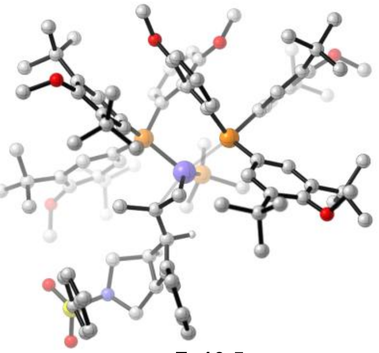

F 10.5

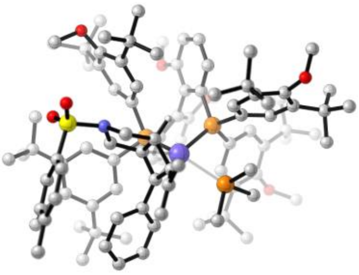

G -7.6

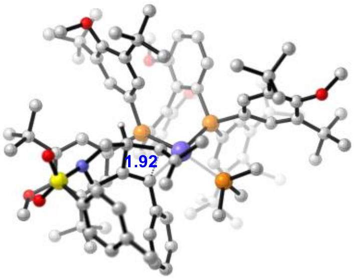

TS$_{G\text{-}H}$ 19.9

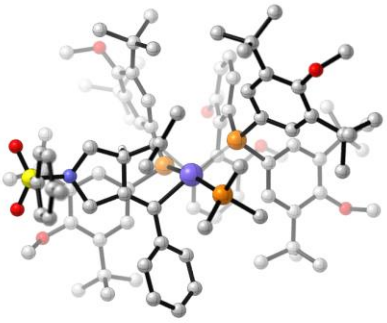

H -14.6

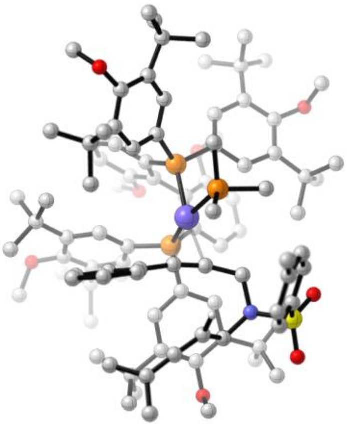

A_S 5.5

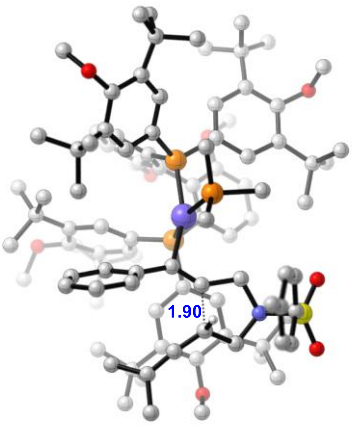

$TS_{A\text{-}B(S)}$ 30.0

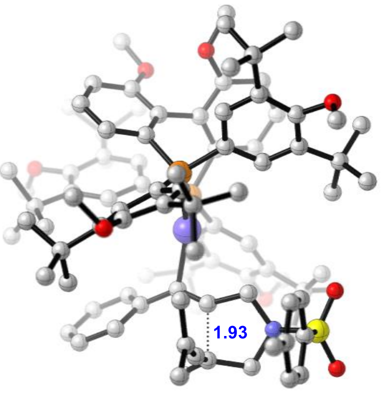

$TS'_{A\text{-}B(R)}$ 35.0

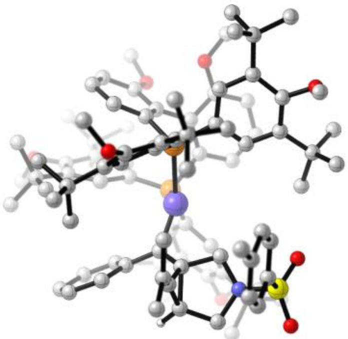

B'_R 7.3

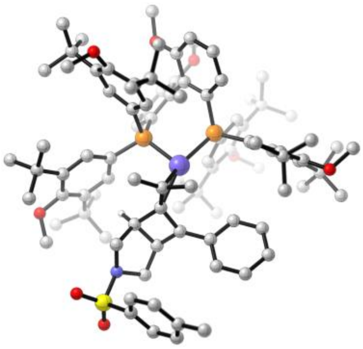

D' -6.7

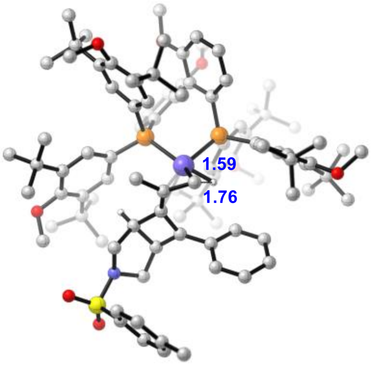

$TS'_{D\text{-}E}$ 11.8

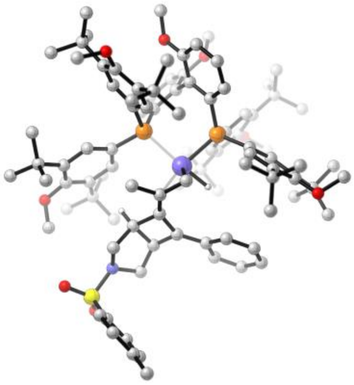

E' 8.8

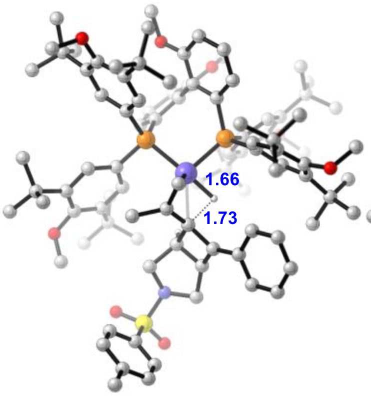

$TS'_{E\text{-}F}$ 22.9

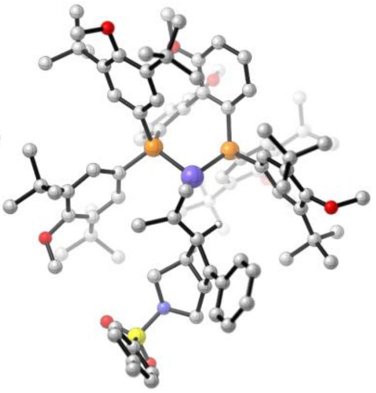

F' 9.1

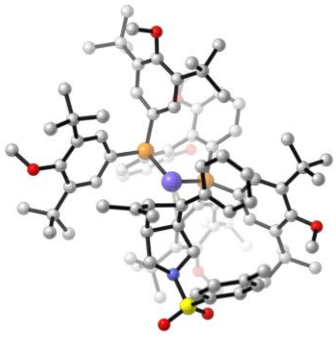

G' -8.2

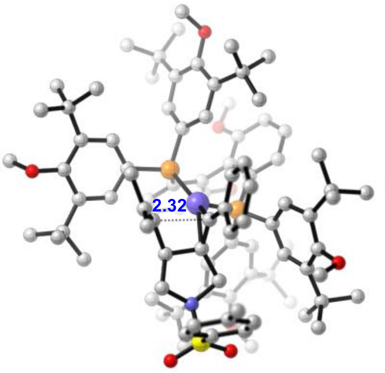

$TS'_{G\text{-}H}$ 18.2

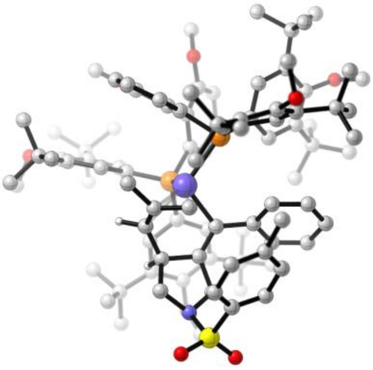

H' -22.7

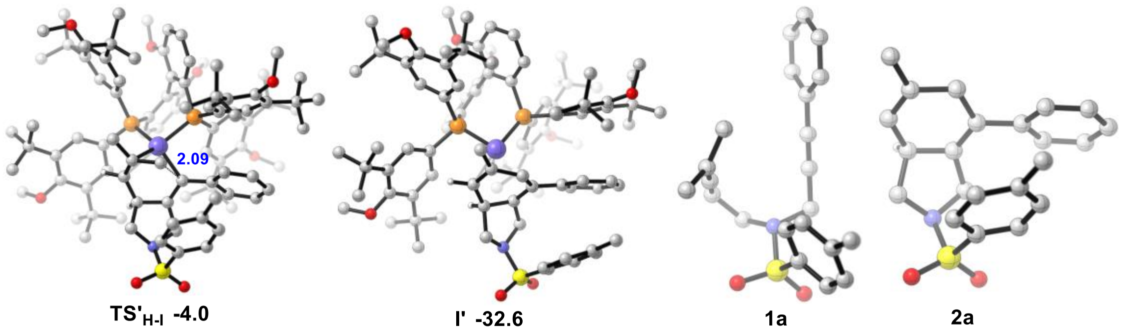

Supplementary Figure 57. Calculated key structural parameters of the intermediates and transition states for the pathway **i** by the M06-L method with the key bond lengths (Ångstroms). Unimportant H atoms are omitted for clarity. Their relative free energy in solution by SMD M06-L//M06-L method are also given.

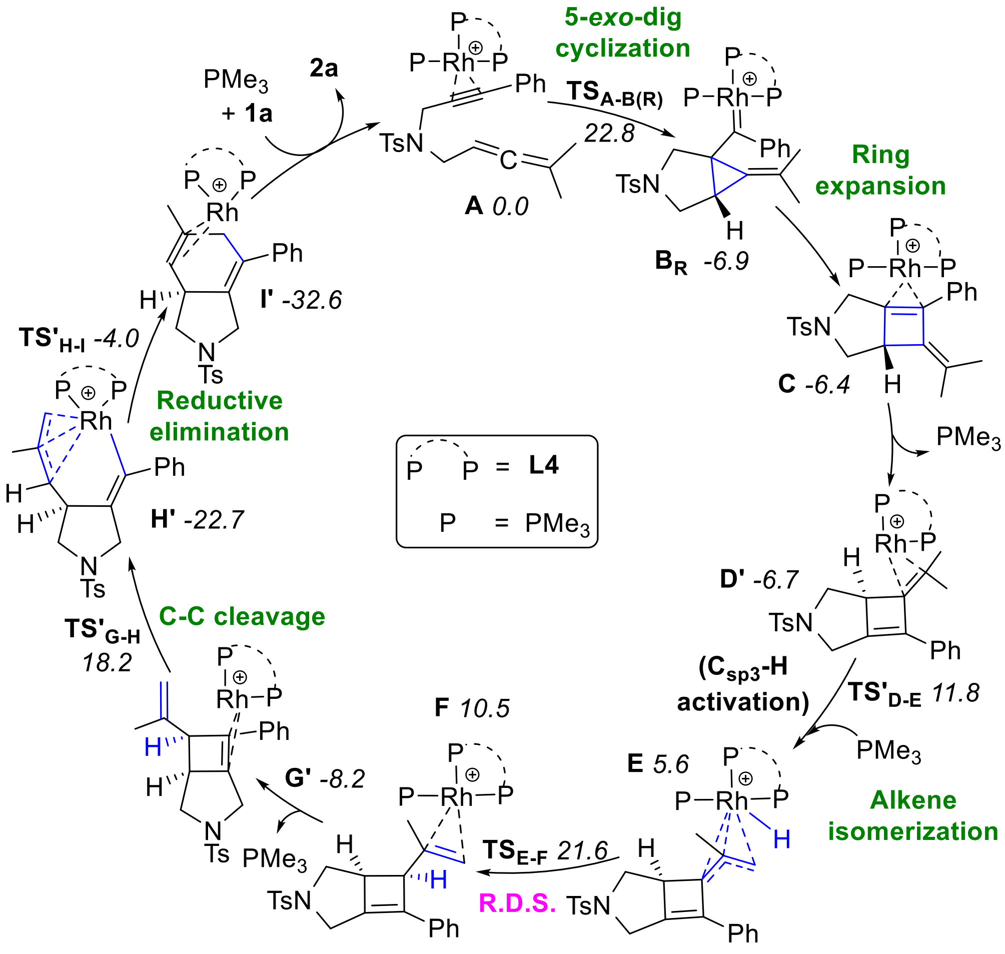

Supplementary Figure 58. Proposed catalytic cycle: Computed free energy profile of the proposed most favorable pathway (**i**) for the novel cationic Rh(I)-catalyzed cycloisomerization of **1a** in solution by the SMD M06-L//M06-L method. PMe_3 ligand was used as the additional ligand. Energetic of the complete pathway **i** is shown in Supplementary Figure S56.

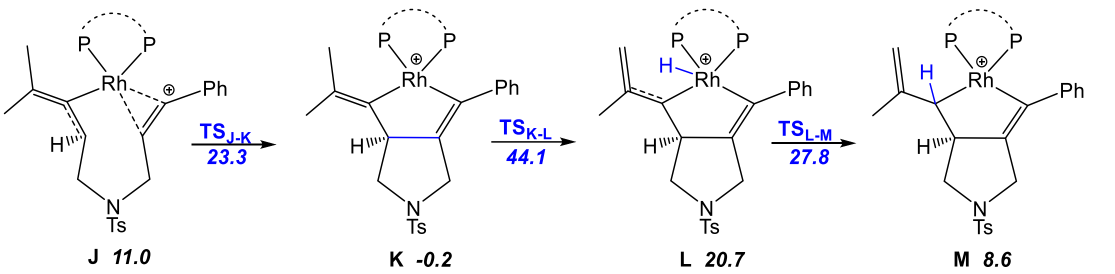

Supplementary Figure 59. The relative free energy profiles of the pathway **ii** for the Rh(I)-catalyzed cycloisomerization of a 1,6-allenyne (**1a**) in solution by the SMD M06-L//M06-L method.

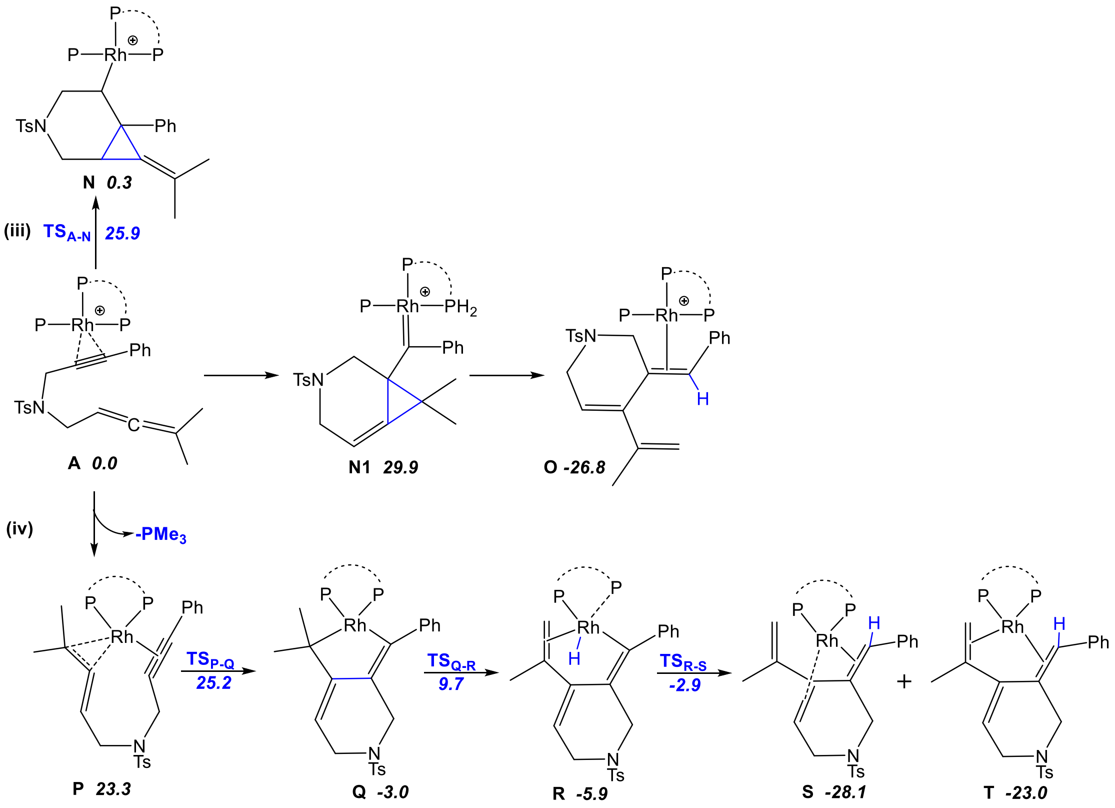

Supplementary Figure 60. The relative free energy profiles of the pathways **iii** and **iv** for the Rh(I)-catalyzed cycloisomerization of a 1,6-allenyne (**1a**) in solution by the SMD M06-L//M06-L method. PMe_3 ligand was used as the additional ligand.

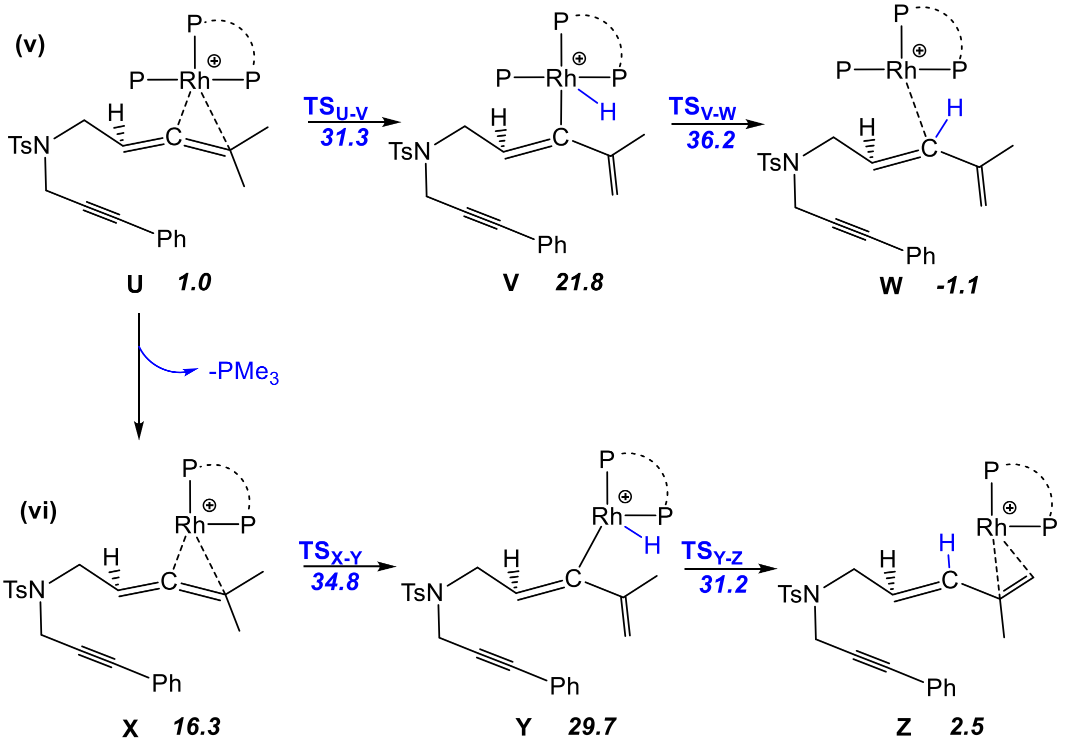

Supplementary Figure 61. The relative free energy profiles of the pathways **v** and **vi** for the Rh(I)-catalyzed cycloisomerization of a 1,6-allenyne (**1a**) in solution by the SMD M06-L//M06-L method. PMe_3 ligand was used as the additional ligand.

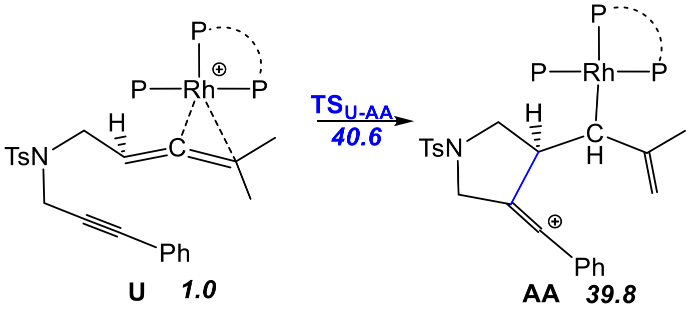

Supplementary Figure 62. The relative free energy profiles of the pathway **vii** for the Rh(I)-catalyzed cycloisomerization of a 1,6-allenyne (**1a**) in solution by the SMD M06-L//M06-L method. PMe_3 ligand was used as the additional ligand.

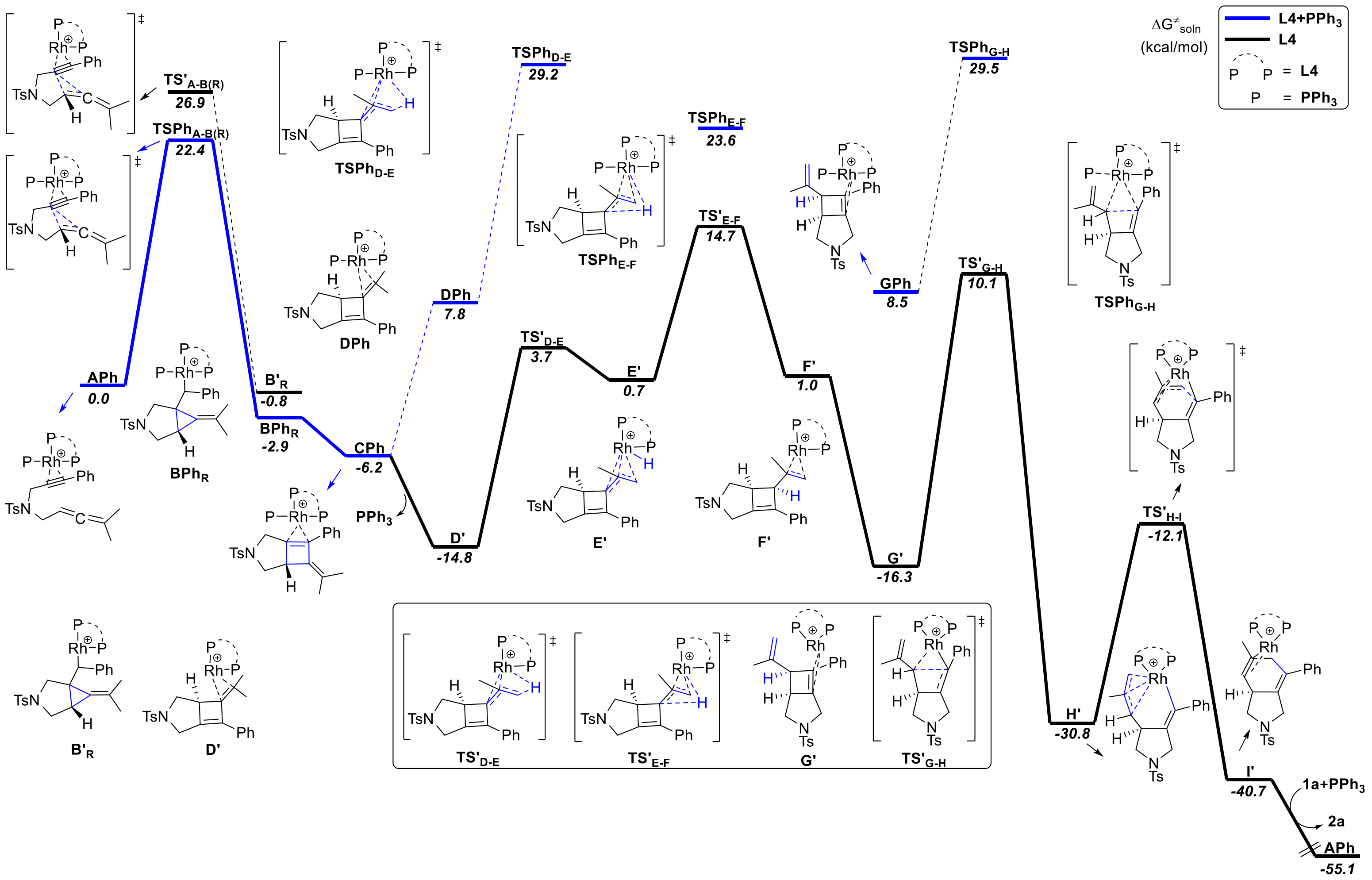

Supplementary Figure 63. The computed free energy profiles for the key geometries in the pathway **i-Ph** of the Rh(I)-catalyzed cycloisomerization of 1,6-allenyne (**1a**) in solution by SMD M06-L//M06-L method. PPh_3 was used as the additional ligand.

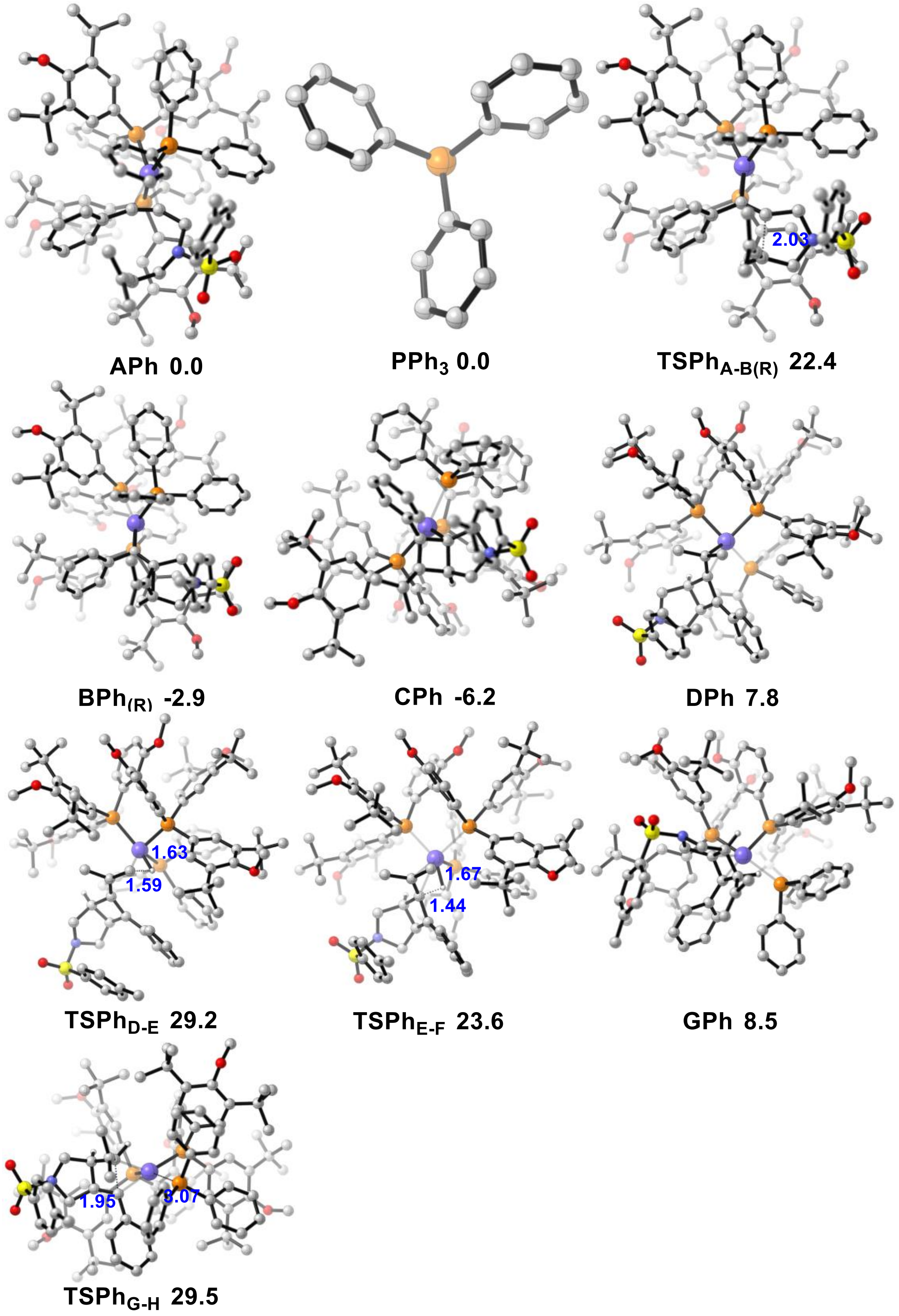

Supplementary Figure 64. Calculated key structural parameters of the intermediates and transition states for the pathway **i-Ph** by the M06-L method with the key bond lengths (Ångstroms). Unimportant H atoms are omitted for clarity. Their relative free energy in solution by SMD M06-L//M06-L method are also given. PPh_3 was used as the additional ligand
