# Supplementary material for: Enantioselective Rhodium-Catalyzed Cycloisomerization of 1,6-Allenynes to access 5/6-Fused Bicycle[4.3.0]nonadienes
Source: Nat Commun. 2019 Feb 27;10:949. doi: 10.1038/s41467-019-08900-z (PMC6393573; doi:10.1038/s41467-019-08900-z)
Supplement: Supplementary file 2 — Description of Additional Supplementary Files [file 41467_2019_8900_MOESM2_ESM.pdf]

Description of Additional Supplementary Files

File Name: Supplementary Data 1

Description: Cartesian coordinates of all optimized structures
